# 5-Hydroxy-2(5*H*)-furanone (HFO) – a versatile and bio-based platform chemical

**DOI:** 10.1039/d6cs00842a

**Published:** 2026-10-26

**Authors:** Jelmer T. Meijer, Magdalena M. Kleybolte, Andries Jensma, Jeya P. Kaniraj, Thomas Freese, Ben L. Feringa

**Affiliations:** a Stratingh Institute for Chemistry, University of Groningen Nijenborgh 3 9747 AG Groningen The Netherlands t.freese@rug.nl t.freese@circolide.com b.l.feringa@rug.nl; b R&D Department Circolide, Circolide Technologies B.V Groningen The Netherlands

## Abstract

The transition toward sustainable chemistry requires a fundamental rethinking of chemical building blocks beyond fossil-derived systems. While conventional petrochemical platform chemicals are industrially indispensable, they suffer from reliance on finite resources, environmental persistence, and restricted structural diversity. In contrast, bio-based and renewable platform chemicals offer major new opportunities, particularly when exploiting the inherent functionality and structural diversity of natural metabolites through bottom-up design strategies. This review highlights 5-hydroxy-2(5*H*)-furanone (5-hydroxybutenolide, HFO), a chiral maleic anhydride analogue, as a promising yet underexplored bio-based platform chemical. It critically assesses its synthetic accessibility from renewable furanic feedstocks and examines key transformation strategies that enable the generation of structurally diverse and enantioenriched derivatives. Emphasis is placed on its multifunctional reactivity, which underpins its application as versatile building block in asymmetric synthesis, fine chemicals, and materials science. Additionally, its potential as a precursor to industrially relevant C4 compounds under mild and sustainable conditions is evaluated. Its broader sustainability and industrial profile are also considered, including renewability, process efficiency, scalability, techno-economic considerations, and commercial relevance. Overall, this review demonstrates HFO not merely as another bio-based intermediate but positions it as strategically valuable platform molecule whose multifunctionality, accessibility, and adaptability enable innovative chemical design. Consequently, it represents a compelling candidate for bridging the gap between renewable resources and high-value applications, supporting the development of a more sustainable and forward-looking chemical industry.

## Abstract 2

5-Hydroxy-2(5*H*)-furanone (HFO) emerges as a versatile bio-based platform chemical, combining renewable accessibility, rich multifunctional reactivity, asymmetric synthesis potential, and routes to high-value chemicals and sustainable materials alike.

## Graphical abstract

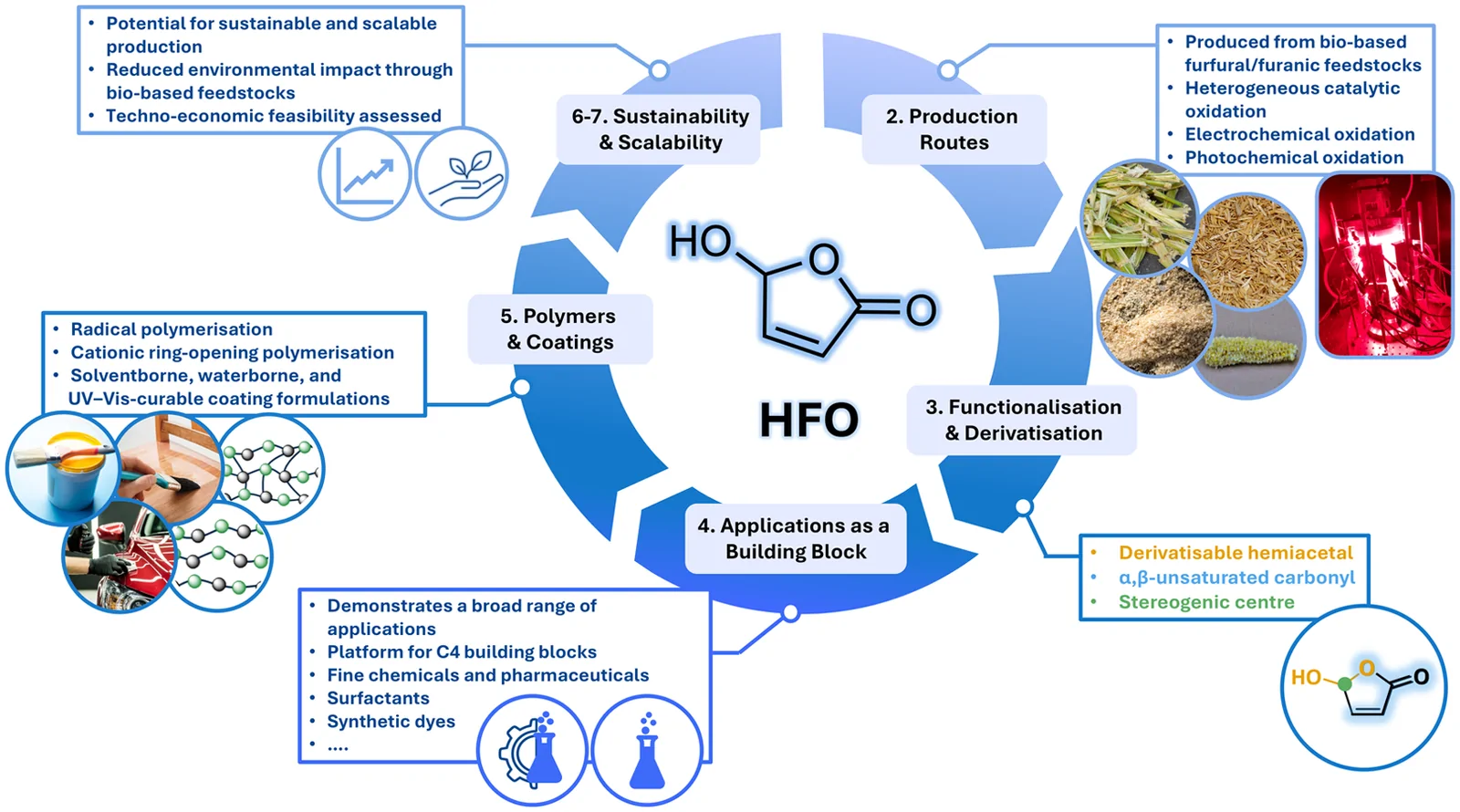

## 1. Introduction – sustainable platform chemicals

“Chemistry itself can be neither ‘good’ nor ‘bad’ in a moral sense […]. Chemists, however, possess the knowledge and skills to make decisions […] that can result in immense benefit to society or cause harm to life and living systems.”^1^ “It is the chemist who has the responsibility […].”^1^ With these opening statements, John Warner and Paul Anastas introduced in 1998 the 12 Principles of Green Chemistry, upon which the foundations of sustainable chemistry stand today.^1–5^ Despite improved understanding and engagement in the field of Green Chemistry, particularly within academia, the incorporation of such practices within the real world remains insufficient. As the global population continues to grow, increasing the demand for chemicals across almost every sector, the planet is facing an escalating climate crisis. Chemistry is frequently associated with high energy consumption, waste generation, greenhouse gas emissions, and health risks, contributing to a negative public perception of the field. As such, industry remains after the transportation and energy sectors one of the largest contributors to global greenhouse gas emissions.^6^ Additionally, the to date irreversible pollution of our ecosystems with so-called ‘forever chemicals’, microplastics, pharmaceuticals, and other chemical pollutants only intensifies the criticism of chemistry.^7–9^

Although these concerns are understandable, chemistry should not be viewed solely as a source of environmental burden, but rather providing opportunities towards a sustainable manufacturing industry. Novel chemicals and materials have drastically improved modern living standards and continue to play a crucial role in addressing global challenges. For instance, advances in chemical research enable the development of more affordable medicines, the production of modern cars and airplanes, help address global food security by improving crop yields and pest control, and support the transition to sustainable energy through the design and synthesis of materials for renewable energy technologies.^10^ In this way, chemistry itself provides many of the tools_-_ needed to overcome the very challenges it is often associated with. Implementing the 12 Principles of Green Chemistry should therefore neither discourage new developments nor hinder innovation.

Consequently, the central question is not “How can we reduce the role of chemistry in our lives?”, but rather “How can we make chemistry itself greener and more sustainable, while promoting innovations needed for a brighter future?”.

### 1.1. Current petrochemical platform chemicals and their limitations

A promising approach to improving the sustainability of chemical production is the use of sustainable platform chemicals. A platform chemical is defined as a versatile intermediate that serves as a foundational building block for producing a wide range of chemicals and materials. These chemicals streamline processes, improve resource and cost efficiency, and facilitate infrastructure. If carefully selected, they can reduce environmental impact significantly.^11,12^

The ‘big five’ petroleum-based platform chemicals are ethylene, propylene, benzene, toluene, and xylenes (BTX).^13^ They emerged as such due to the high-volume production of a few dominating polymers, including polyethylene (PE), polypropylene (PP), polyvinyl chloride (PVC), polyethylene terephthalate (PET), polyurethane (PU), and polystyrene (PS) (see Fig. 1). Globally, plastics production alone exceeds 400 Mt per year, which represents up to 80% of all organic chemicals produced when excluding fuels.^14–16^ Ethylene and propylene, the world's two leading petrochemicals and the simplest olefins, are primarily produced *via* steam cracking and are widely used in the production of PE and PP, respectively. Benzene is mainly used to synthesize products such as styrene (used as monomer for PS), phenols, and nylon intermediates. Toluene and xylenes, collectively known as BTX, are widely used as solvents and as precursors for materials such as PET.^15,17^

**Fig. 1:**
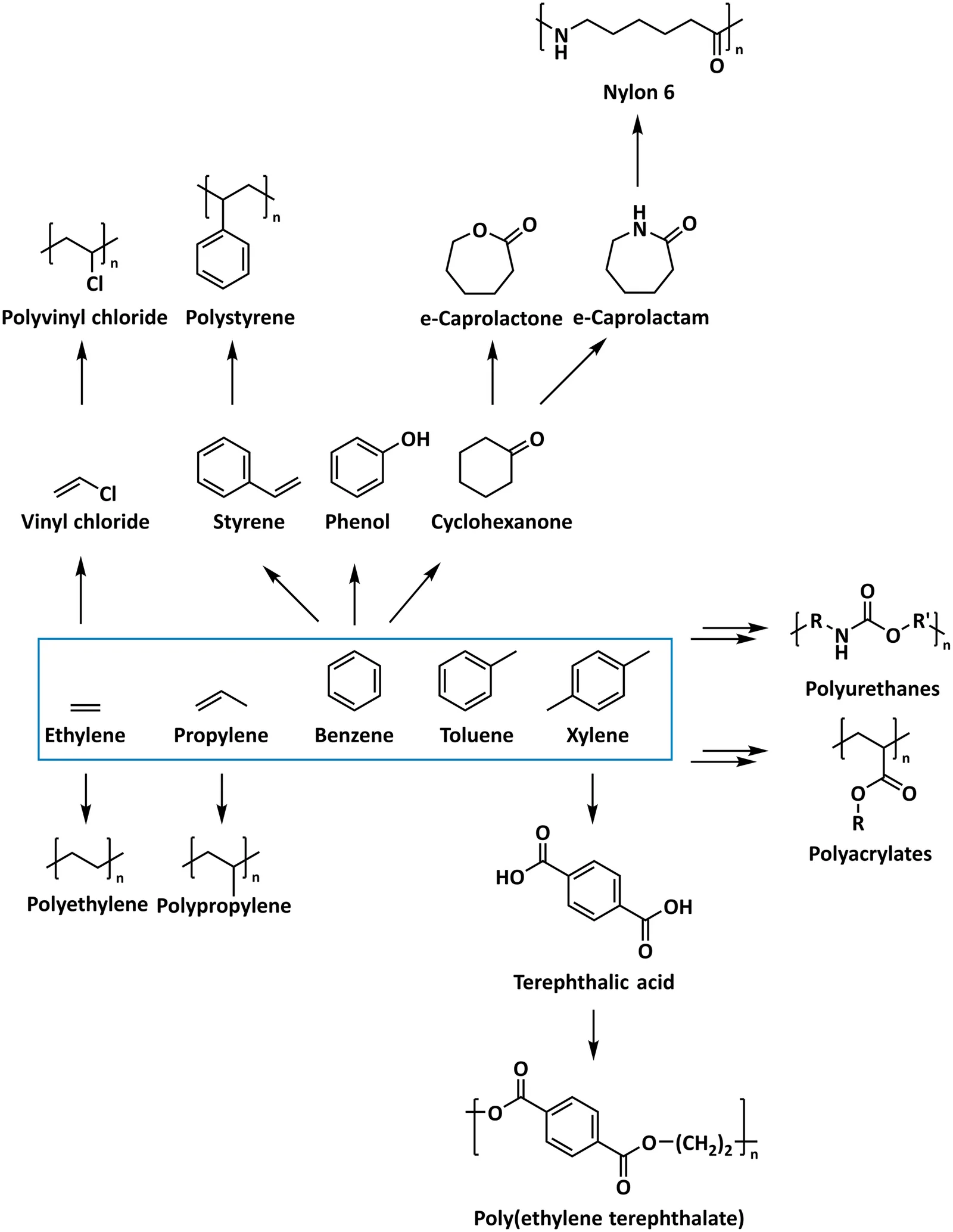
The “big five” petrochemical platform chemicals (blue), derivates and their highest-volume materials.^13^

Understandably, these building blocks are considered vital for the chemical industry, highlighting our strong dependence on these platform chemicals. However, despite their industrial importance, these petrochemicals face structural, technological and environmental limitations that must be addressed to ensure future ecological and economic sustainability.^16^ Ecological limitations arise due to their toxicity, the high CO_2_ emissions during production, as well as their dependence on finite crude oil resources.^14,16,17^ Furthermore, many petrochemical-derived materials and their degradation products are inherently persistent, due to their limited biodegradability resulting in long-term environmental accumulation.^9^ Beyond sustainability, there are several additional challenges associated with crude-oil based platform chemicals and the infrastructure built around them. As their production relies on highly centralized, capital- and energy-intensive processes, such as steam cracking, flexibility and regional adaptability are limited. This makes the system less resilient to supply chain disruptions. Additionally, while their simplicity enables efficient large-scale production, it limits their versatility in synthesising more complex and specialised molecules. This reinforces a production paradigm in which materials are designed to meet immediate quantitative demands rather than considering evolving/future functionality requirements.^18^ Consequently, producing highly functional materials often necessitates multiple energy- and resource-intensive transformation steps to introduce functional groups or stereochemical complexity. High selectivity and well-defined chemical complexity are often limited by current synthetic capabilities and may not always be possible.^16^ Altogether, these factors highlight not only the environmental but also the structural and technological constraints of current petrochemical platform chemicals rendering the need for more versatile and sustainable alternatives inevitable.^19^

Building on these limitations of current petrochemical platform chemicals, increasing attention is being directed toward bio-based, structurally diverse, and sustainable alternatives, reflecting the notion that “nature provides more efficient and green solutions.”

### 1.2. Opportunity from nature: bio-based platform chemicals

As discussed in Section 1.1, platform chemicals are of immense importance to the chemical industry, and their relevance becomes even greater in the context of sustainability. Many desirable attributes of sustainable platform chemicals – bio-based origin, structural diversity, and sustainability – can be found in plant-derived feedstocks. However, before discussing these feedstocks in more detail, the terms “bio-based” and “sustainable” should be defined first.

Contrary to a common misconception, “bio-based” does not necessarily imply “sustainable”, and *vice versa*. Bio-based chemicals are defined as compounds derived from biological resources. These resources may include renewable waste biomass streams from agriculture, forestry, and food processing, but can also originate directly from living organisms.^20^ Consequently, the term ‘bio-based’ refers only to the origin of a material and does not necessarily reflect its environmental performance. In contrast, sustainable feedstocks are materials whose production and use maintain ecological balance, rely on renewable resources that minimize competition with food production, and reduce environmental impact.^21^ Among bio-based resources, plant-derived feedstocks represent an important and widely used subset. These materials are commonly categorized according to their biological roles into primary and secondary metabolites. Primary metabolites are essential for plant growth, their development, and processes like photosynthesis, while secondary metabolites mainly contribute to defence mechanisms and environmental adaptation. These compounds can be grouped according to their chemical structure into eight overarching classes: nucleic acids, proteins, carbohydrates, lipids, nitrogen-containing and non-nitrogen-containing secondary metabolites, terpenoids/isoprenoids, and phenolics.^22–25^ Each of these classes can be further subdivided based on structure and function, as depicted in Fig. 2. Carbohydrates, for example, can either have a structural function as found in cellulose, hemicellulose, and pectin, or a storage function, *i.e.* starch and simple sugars.^22^ It is important to note however that this categorization of biomass is not strict and hierarchical because of their interconnected nature. Depending on their application, the bio-based chemicals are either directly obtained from the plant-based feedstock *via* extraction or distillation or through bioprocesses such as fermentation, biocatalysis, and metabolism in microorganisms.^12,26,27^

**Fig. 2:**
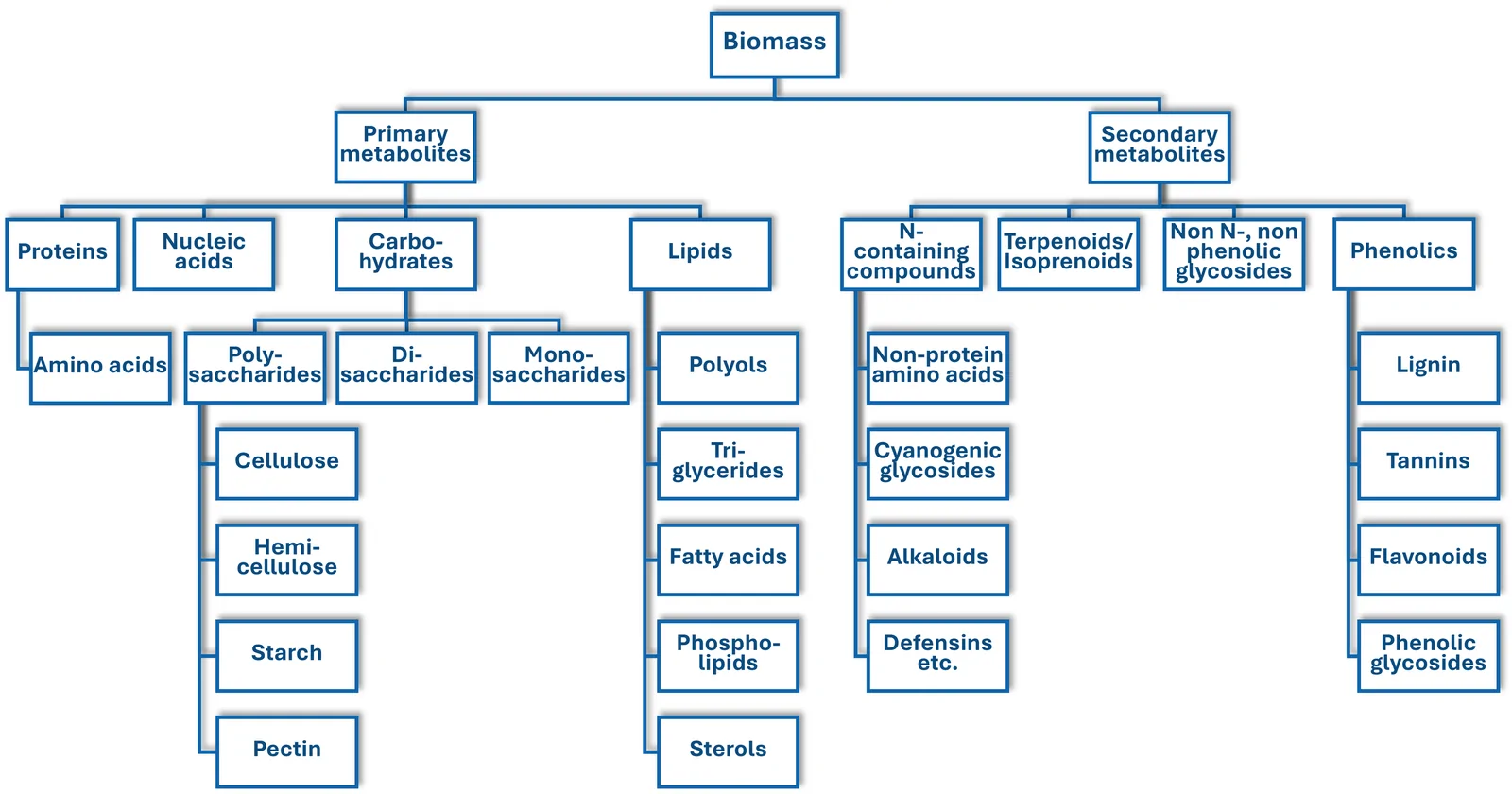
General categorisation of plant-derived biomass and its major chemical constituents. Typical biomass composition (dry basis) is commonly reported as: cellulose (∼35–50%), hemicellulose (∼20–35%), lignin (∼10–30%), extractives (∼2–10%), proteins (∼1–10%), lipids (∼1–5%).^22,23^

As highlighted before, natural metabolites frequently contain multiple functional groups, heteroatoms, and stereogenic centres that can serve as valuable handles for further chemical transformations and value-added products. This inherent molecular complexity can reduce the number of synthetic steps required to access functional materials and intermediates. As a result, bio-based molecules offer unique opportunities to develop structurally diverse platform chemicals that would be difficult or inefficient to obtain from conventional petrochemical building blocks.

#### 1.2.1. Approaches to sustainable platform chemicals

The development of sustainable platform chemicals can generally follow two main approaches. The most common strategy is the so-called drop-in approach in which only the origin of the chemical is replaced without changing its structure or functionality.^28^ As a result, existing infrastructure, established risk assessments, integrated processes, and downstream product streams can be directly utilised. Although this approach promises to be fast and streamlined, it fails to meet the increasingly sophisticated needs of modern society, which often require ever more structurally complex chemicals. The second strategy, referred to as the bottom-up approach, utilizes naturally occurring compounds that resemble petroleum-derived molecules but differ from them structurally and functionally. These platform chemicals benefit from the diverse and often hard-to-synthesize functionalities already present in biomolecules, rendering them suitable for both existing and novel applications. However, extensive data on their toxicological profiles, performance, and life-cycle analysis are often still required for their commercial adoption.^29^

Both the drop-in and bottom-up approach offer distinct advantages and limitations. However, the simple replacement of existing petrochemical platform chemicals alone is unlikely to drive substantial progress toward sustainable chemical production.

Sustainability in chemistry not only involves replacing conventional, non-green processes but also requires the development of novel, innovative chemical solutions that address present and future societal needs. While drop-in substitutions may improve the sustainability profile of certain products, they often remain in direct competition with well-established and cost-efficient petrochemical analogues. Ultimately, the future impact of bio-based chemistry will not be defined by how well it imitates petrochemicals, but by how valuably it differentiates from them. By creating alternative processes and platform molecules with new structural features and thus functionalities, the field can surpass direct competition and unlock entirely novel, economically viable application areas.

Accordingly, this review focuses on bottom-up platform chemicals, specifically HFO, emphasizing their design, development, and potential applications.

#### 1.2.2. Established bio-based bottom-up platform chemicals

Several bio-based platform chemicals have already been established (Fig. 3). These examples demonstrate that bottom-up strategies are not only conceptual but have already gained industrial relevance.

**Fig. 3:**
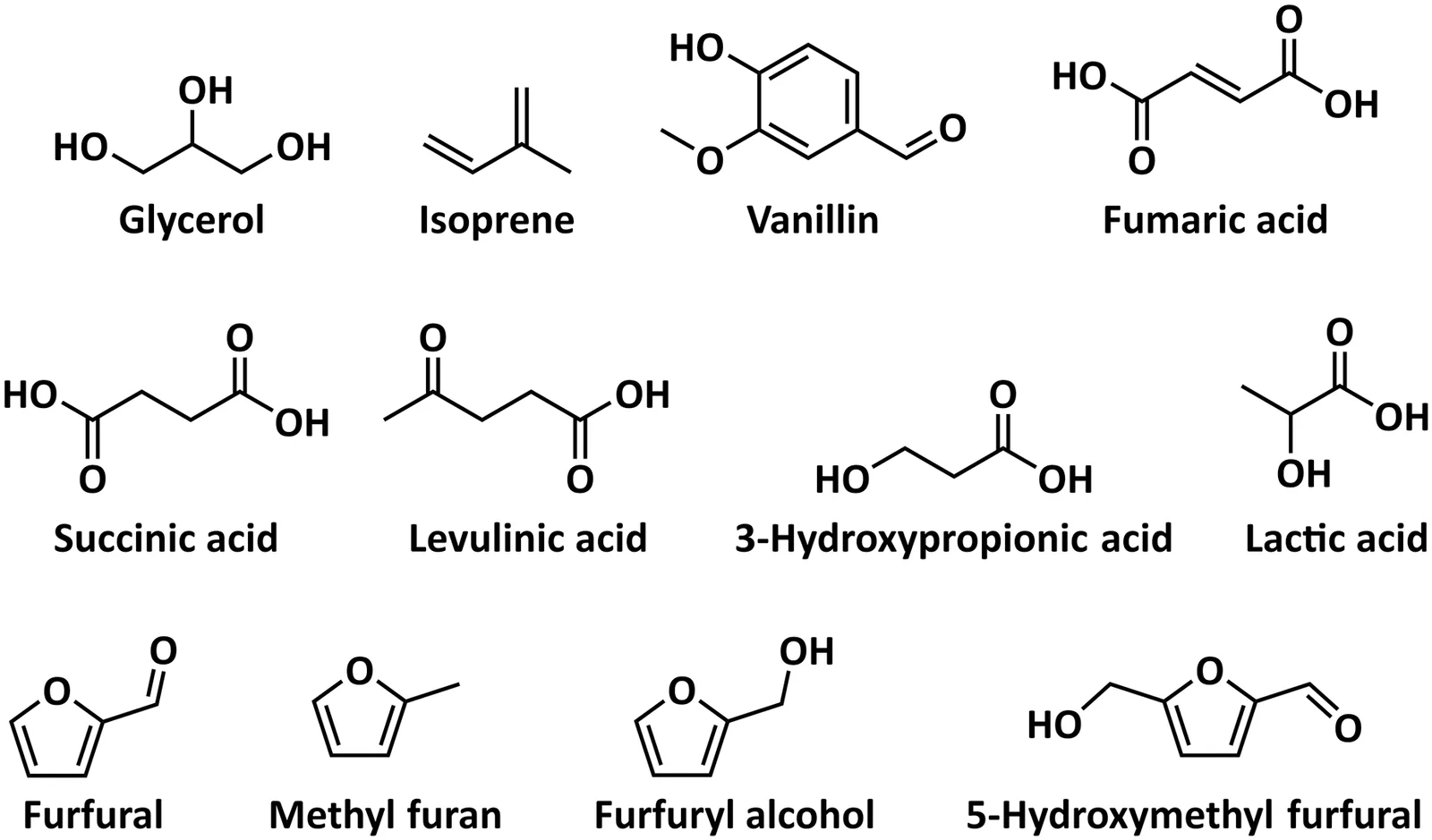
Exemplary bio-based molecules already established as sustainable platform chemicals.

Among the most established examples is succinic acid, which is produced *via* microbial fermentation.^27^ Owing to its versatility as a precursor for biodegradable polymers (*e.g.* polyester), resins, and solvents, it is considered a viable substitute for several petroleum-derived intermediates in polymer chemistry.^27^ Similarly, lactic acid represents one of the most mature bio-based chemicals and is widely produced at industrial scale. It serves as the monomer for polylactic acid, which has rapidly growing applications in packaging and biomedical fields.^26,30,31^ Another key molecule is 3-hydroxypropionic acid, which can be derived from glycerol and subsequently converted into acrylic acid and other valuable derivatives, making it a promising platform molecule, used in coatings, adhesives, and superabsorbent polymers.^32,33^ Meanwhile, levulinic acid, which is usually obtained through the acid-catalysed degradation of cellulose, is a prime example of the exploitation of plant-based biomass. It serves as a versatile precursor for fuel additives, plasticisers and speciality chemicals.^34,35^ Terpenes are also frequently considered platform chemicals due to their highly complex structures based on isoprene. They are widely utilised in fragrances, pharmaceuticals, and as renewable feedstocks for bio-based chemicals and advanced materials.^36,37^ Finally, furfural, one of the earliest industrial bio-based chemicals, is derived from hemicellulose. It is highly relevant due to its role in furan-based chemistry and serves as a precursor to numerous derivatives, which are widely used in resins, solvents, and emerging bio-based materials.^38,39^

Given the importance of furfural derivatives, increasing attention has been directed toward structurally related oxygen-containing heterocycles. Among these, γ-butenolides (also known as furan-2(5*H*)-ones) stand out as a promising class of platform molecules. As their name suggests, they can be synthesised from furan or its substituted derivatives,^40–42^ but they also occur naturally in various biological systems, including terpenoids.^43^

### 1.3. Butenolides – naturally available and synthetically accessible platform chemicals

γ-Butenolides (furan-2(5*H*-ones) are an important class of 5-membered α,β-unsaturated lactones, often occurring as enantioenriched structural framework embedded in numerous natural products. These compounds display a wide range of biological activities. Well-known examples include ascorbic acid (commonly known as Vitamin *C*), an important antioxidant in the human body; (+)-strigol, a terpenoid phytohormone known to trigger germination of parasitic plants and inhibit plant shoot branching;^44,45^ and *Asteriscunolide D*, showing significant anticancer activity towards different cell lines.^46^ Other prominent examples are *Avenolide*, a streptomyces hormone, involved in antibiotic production in *Streptomyces avermitilis*^47^ and protoanemonin, a lactone found in *ranunculaceae* plants showing antimicrobial activity.^48^ It is therefore unsurprising that this class of compounds has attracted the interest of many researchers, prompting extensive synthetic efforts to access molecules containing this motif.^49–56^ As a result, they have found their way into reports covering their synthesis, transformations, and applications.^40,56–89^ Several naturally occurring molecules containing the γ-butenolide motif are shown in Fig. 4.

**Fig. 4:**
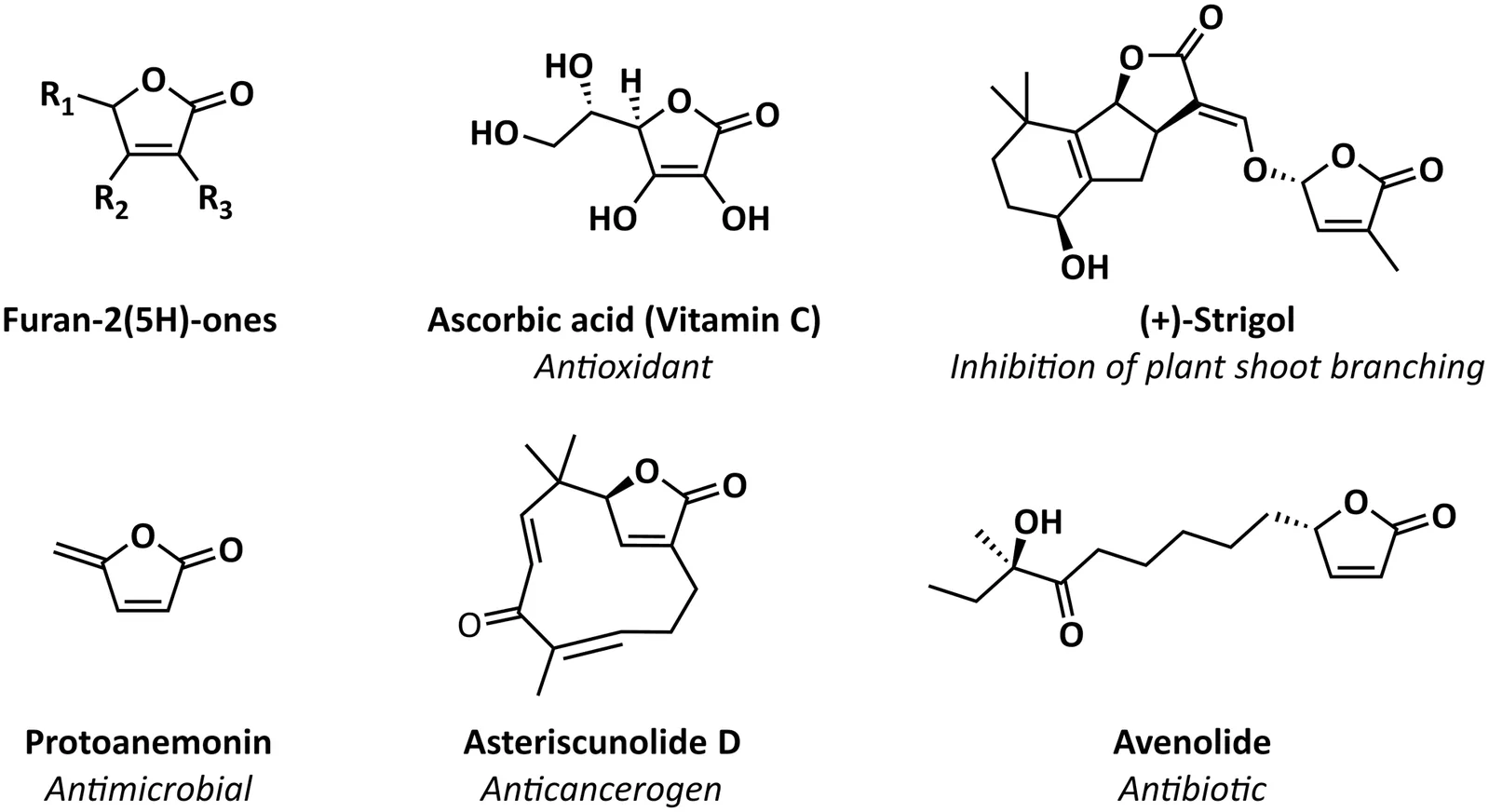
Naturally occurring products containing the γ-butenolide motif.

Due to their high reactivity, butenolides serve as versatile building blocks for the synthesis of numerous reagents and biologically active compounds. The simplest butenolide, 2-furanone (see Fig. 5), is utilised extensively in the synthesis of pharmaceutical products, natural products and fine chemicals.^73^ It is industrially produced *via* deoxygenation of substituted butyrate, hydrolysis of substituted furan, or cyclocarbonylation of terminal alkynols.^77,90^ More environmentally friendly approaches focus on the reaction of furfural with hydrogen peroxide (H_2_O_2_).^79,91^ In a related context, *Angelica lactone* is readily attainable from the platform chemical levulinic acid, and has emerged as a valuable building block, finding applications in natural product synthesis, asymmetric catalysis and the transformation into value-added products such as γ-valerolactone, polymers and biofuels.^18,84^ 5-Hydroxymethyl-furanone is another prominent biologically sourced furanone, obtained by reacting levoglucosenone with H_2_O_2_, providing a chiral compound with a double bond, lactone ring and primary alcohol as a playground for synthetic chemists.^65^

**Fig. 5:**
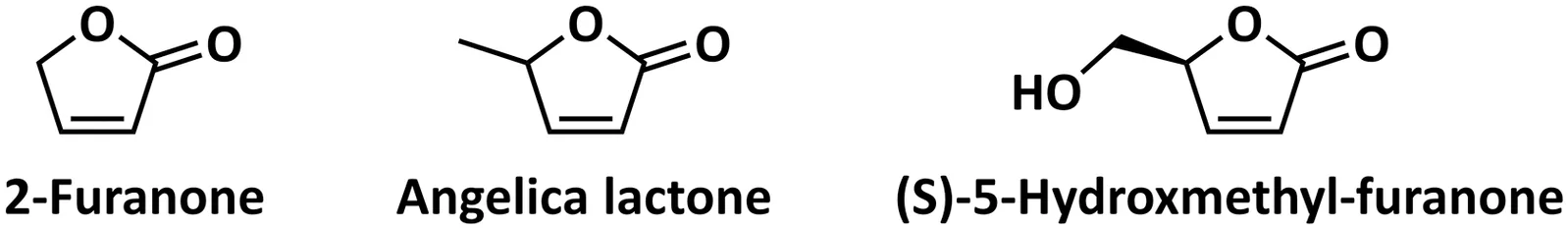
Accessible butenolide building blocks used in synthesis.

In this context, this review aims to highlight the potential of 5-hydroxy-2(5*H*)-furanone 1 (Scheme 1), hereafter referred to as 5-hydroxybutenolide or HFO, as a bio-based platform chemical. It can be readily obtained from bio-based furanic compounds, such as furfural, which will be discussed in greater detail in Section 2. Its synthesis was first reported in 1913 by Ciamician and Silber^92^ through photooxidation of furoic acid, and its derivatization has been studied intensively from the 1980s onwards. HFO is a highly versatile building block and can be regarded as a chiral, hydroxylated analogue of maleic anhydride, bearing a lactone ring, an olefinic bond, and a hemiacetal that together offer broad opportunities for further functionalisation. Its ability to be converted into either the R- or S-configured chiral derivatives through relatively straightforward and dynamic transformations highlights its exceptional utility in asymmetric synthesis. Compared to structurally related compounds, which lack comparable chiral versatility, HFO offers a distinct advantage for the development of bio-based chiral molecules.^49,93,94^

**Scheme 1:**
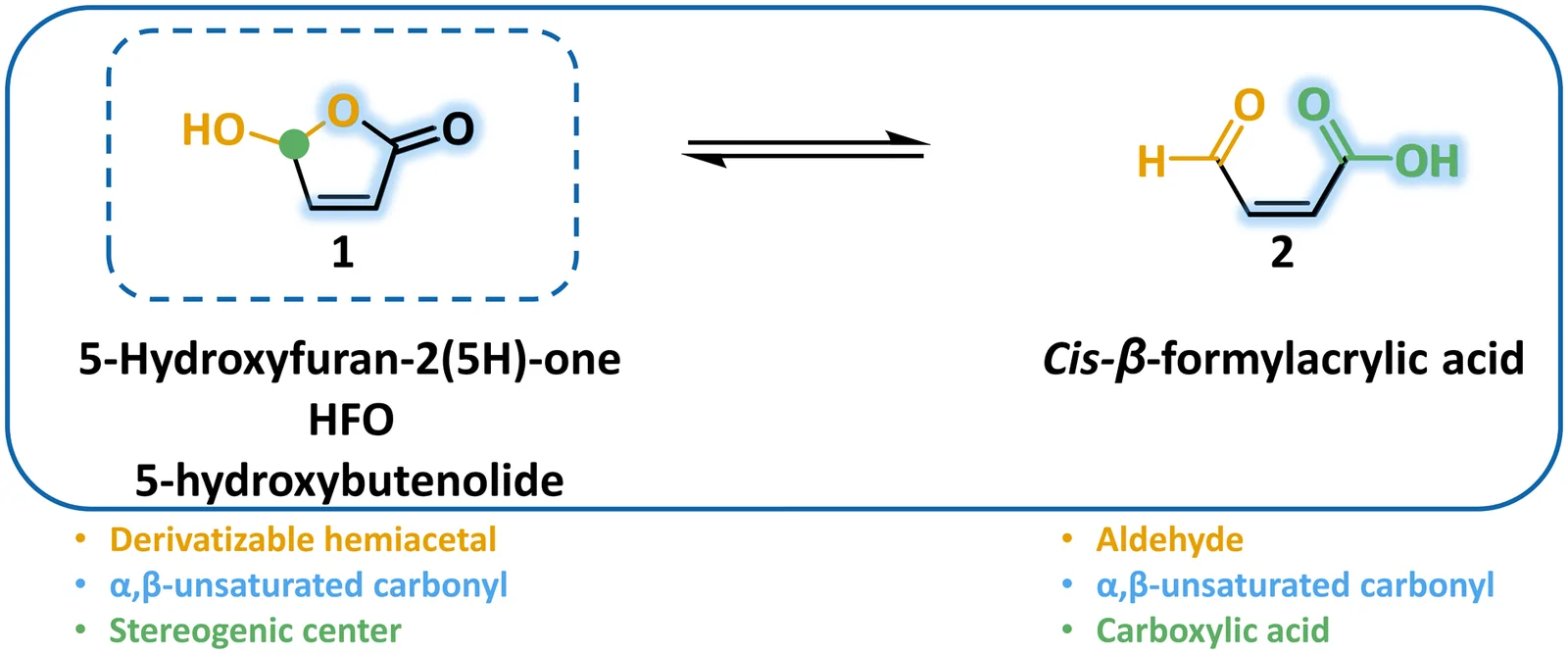
Tautomeric equilibrium between HFO and *cis*-β-formylacrylic acid.

It exists in equilibrium with its ring-opened form, *cis*-β-formylacrylic 2 (Scheme 1), but is typically observed as the closed form in organic media or upon isolation. In aqueous media, however, tautomeric transformations to *cis*-β-formylacrylic acid were reported.^95,96^

It is important to emphasize that HFO contains a stereogenic centre and is therefore typically obtained as a racemic mixture upon production. This chirality is a key feature of the molecule as it enables access to enantioenriched derivatives present in numerous natural products and biologically active compounds. Racemic HFO has been enantioenriched through chiral auxiliary-based resolution, where diastereomerically pure 5-menthyloxybutenolides 3a and 4a (Fig. 6) are accessed through acetalization with d- or l-menthol.^56^ The diastereomers are then separated by crystallization due to their difference in physical properties. Other methods to obtain enantiomerically pure butenolides are *via* dynamic kinetic asymmetric transformation (DYKAT) or enzymatic catalysis.^94,97,98^ These strategies will be discussed more in depth in Section 3. More recently HFO has been highlighted as a promising C_4_ platform chemical for the synthesis of several industrially established chemicals like maleic acid, γ-butyrolactone, 1,4-butanediol, tetrahydrofuran (THF) and pyrrolidines *via* heterogeneous catalysis under mild conditions.^99^

**Fig. 6:**
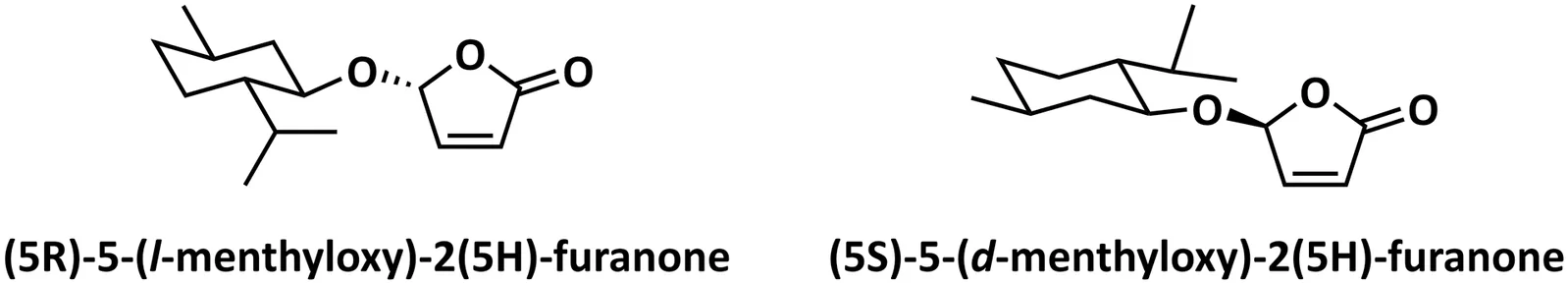
Diastereomerically pure 5-menthyloxybutenolides used for the synthesis of chiral building blocks.

We showed that butenolides, bearing a cyclic acrylate motif, are highly attractive for applications in polymers and materials science, including coatings.^100–103^ The α,β-unsaturated double bond is able to take part in radical polymerisations in the presence of several comonomers like vinyl ethers, vinyl esters and vinyl lactams. 5-Alkoxy-2(5*H*)-furanones (5-alkoxybutenolides) have been further incorporated in polymers with oxiranes and vinyl ethers, providing hydrolysable acetal moieties in the main chain.^104^

In summary, HFO is a versatile and accessible bio-based platform chemical that can be transformed under mild conditions into a wide range of high-value products. Its high reactivity, combined with its chirality, enables applications from asymmetric synthesis to materials science, highlighting its considerable potential for future developments.

By drawing inspiration from nature, bottom-up bio-based platform chemicals can provide access to inherently structurally complex molecules, whose defined steric environments and diverse functional groups enhance synthetic efficiency and position them as attractive alternatives to petrochemical counterparts. Such next-generation platform materials will be essential for developing an innovative and sustainable chemical industry. Within this context, HFO emerges as a particularly promising, yet still underrepresented, candidate. Its unique combination of structural features, reactivity, and accessibility from renewable resources shows its potential as a next-generation platform chemical, highlighting its attractiveness for applications in pharmaceuticals, specialty coatings, agrochemicals, and other bio-based materials. Despite a growing body of literature and major recent progress, a comprehensive overview of its chemistry and applications is lacking, motivating the need for this review. In the following section, we will further outline the scope and structure of this review.

### 1.4. Scope and structure of this review

This review aims to provide both a comprehensive overview of HFO as a new platform chemical and a critical reflection on its current development, potential and context regarding sustainability and Green Chemistry. Rather than viewing this shift as merely a substitution issue, we recognise it as an opportunity to develop molecules that offer inherent functional advantages and expand the scope of chemical design and flexibility towards several applications.

Based on this, we present butenolides as a particularly promising class of compounds. Their structural diversity and reactivity position them between simple petrochemical building blocks and highly complex bio-derived molecules. Well-established compounds such as 2-furanone and *angelica lactone* have already demonstrated the value of this scaffold in synthetic and materials chemistry. Within this family, HFO stands out by exhibiting multifunctionality with tuneable reactivity.

The main objective of this review is to establish HFO as a true sustainable platform chemical. To achieve this, the review is comprehensively structured from its synthesis to application (illustrated in Fig. 7). First, the synthetic accessibility of HFO and its derivatization pathways are discussed. Next, its function as a bio-based building block across different sectors, with particular emphasis on polymer chemistry and advanced materials in comparison to conventional petrochemical analogues, is discussed. Subsequently, we aim to evaluate its sustainability profile, including environmental impact, biodegradability, and scalability. Finally, future research directions that support its role as a platform chemical for sustainable innovation in chemistry and materials science are outlined.

**Fig. 7:**
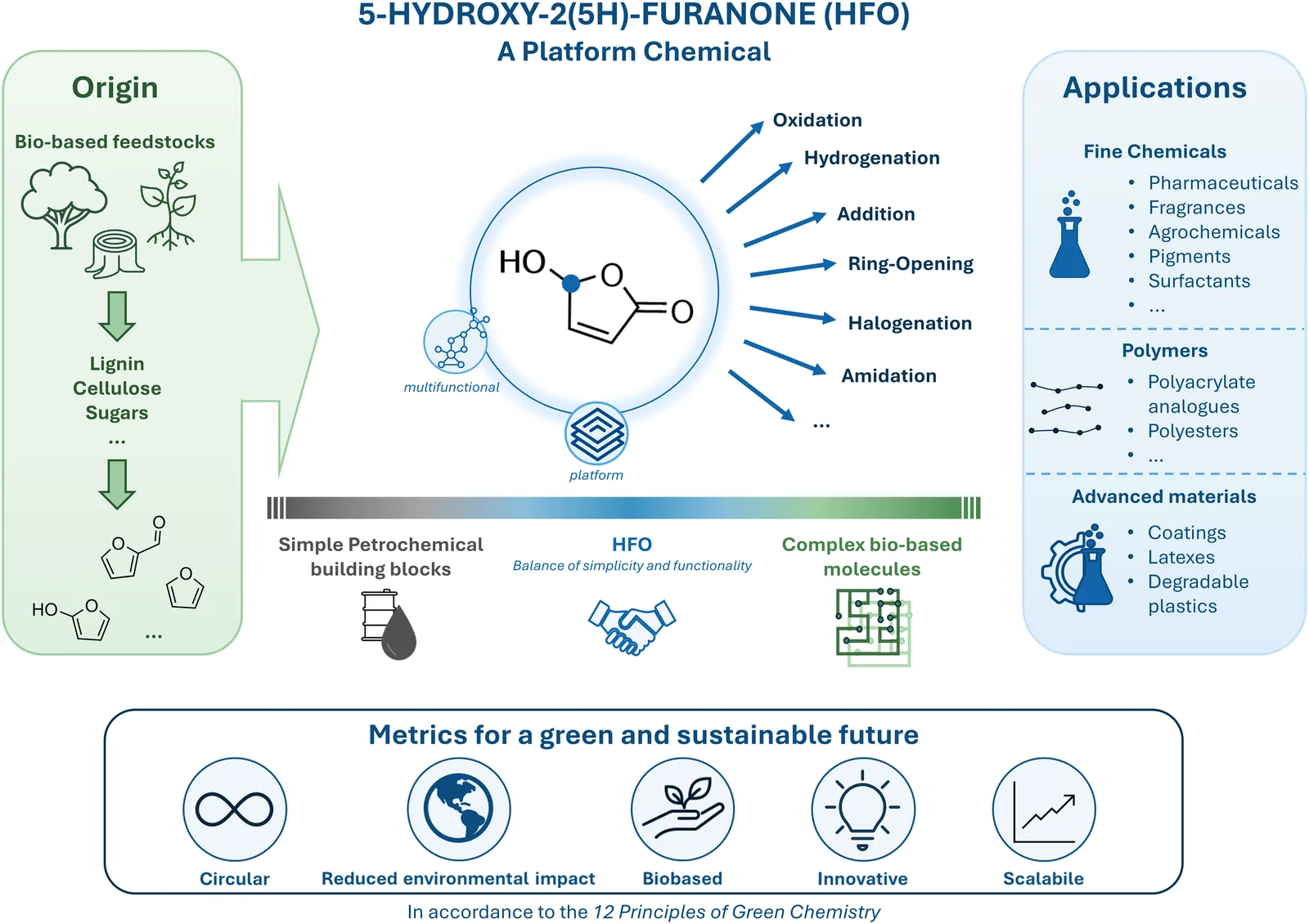
Descriptive illustration of the scope and structure of this review. 5-Hydroxy-2(5*H*)-furanone (5-hydroxybutenolide, HFO) is depicted as a bridge between simple petrochemical and complex bio-based building blocks. The origin of HFO is shown on the left, its derivatisation into other valuable molecules is presented in the centre, and their applications are illustrated on the right. As the review concludes with an evaluation of HFO regarding its suitability as a versatile and sustainable platform chemical, the figure also highlights the properties of HFO that, in light of the 12 Principles of Green Chemistry, support its potential as a sustainable platform chemical.

## 2. Production routes toward 5-hydroxy-2(5*H*)-furanone

The importance of 5-hydroxy-2(5*H*)-furanone (5-hydroxybutenolide, HFO), is reflected in the variety of synthetic methods reported in the literature. These include photocatalytic, thermocatalytic, and electrocatalytic approaches, as well as oxidation reactions of furan and its derivatives.

### 2.1. Photocatalytic oxidation

#### 2.1.1. Batch photooxidations

The earliest and most widely reported method for obtaining HFO is the photooxidation of furfural 3 with singlet oxygen (^1^O_2_) in alcoholic solvents. In this process, photosensitizers such as methylene blue or rose bengal are excited by visible (sun)light and subsequently transfer energy to molecular oxygen (O_2_) through triplet–triplet energy transfer, generating ^1^O_2_.^40–42,85^^1^O_2_ then undergoes a [4+2] cycloaddition with furfural 3 to form unstable endoperoxide intermediate 4. Subsequent nucleophilic attack of the solvent methanol results in cleavage of the endoperoxide, affording HFO 1 with elimination of methyl formate 5 (Scheme 2A).^40–42,85^

**Scheme 2:**
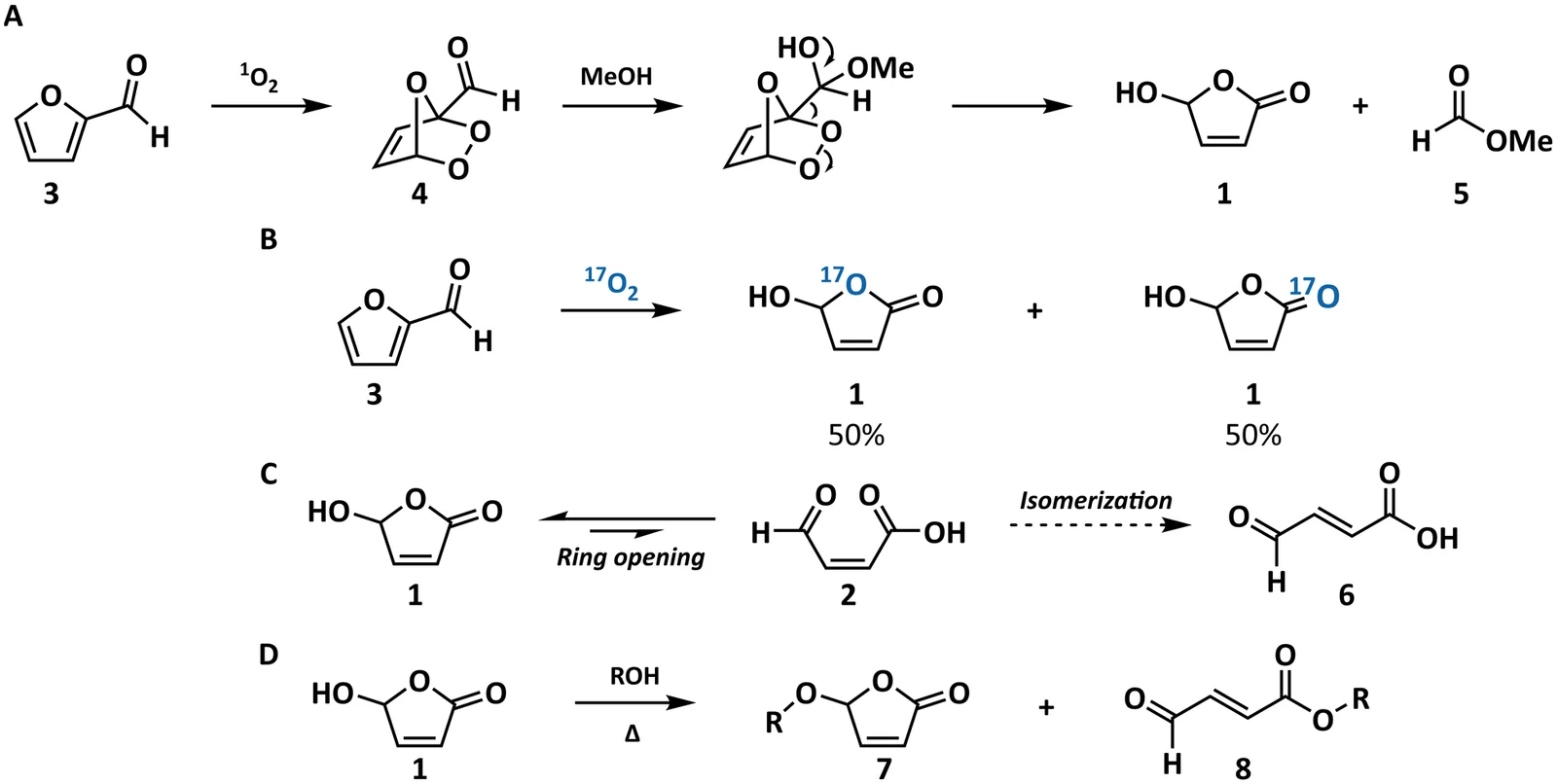
(A) Mechanism for photooxidation of furfural with singlet oxygen in methanol, (B) ^17^O_2_ isotopic labelling studies confirm the transient opening of the lactone ring, (C) transient ring-opening to *cis*-β-formylacrylic acid, which can isomerize to *trans*-β-formylacrylic acid, (D) alkoxybutenolide and ring-opened side products formed when work-up or photooxidation is performed above 40 °C.^40–42,85,105,106^

Mechanistic studies using ^17^O_2_ showed isotopic labelling of both the intracyclic and carbonyl oxygen atoms, confirming that the two isotopomers are in equilibrium *via* transient ring-opening of the lactone (Scheme 2B and C).^105^ Heat generated by the lamps or introduced during workup can promote side reactions, leading to formation of 5-alkoxybutenolides 7 through condensation with the alcoholic solvent, or to *trans*-β-formylacrylic esters 8*via* ring-opening and *cis*–*trans* isomerization (Scheme 2C and D).^85,105,106^ These side reactions can largely be avoided by maintaining the reaction and work-up temperatures below 40 °C.^40–42,85^

In addition to furfural,^54,107^ HFO can also be synthesized from other furan derivatives, including furan,^108,109^ 2-furoic acid,^110,111^ and furfuryl alcohol,^112^ using similar photooxidation conditions. Reactions starting from furfural, 2-furoic acid, or furfuryl alcohol typically provide quantitative yields towards HFO, whereas the reaction with furan is less selective and affords lower reported yields (35–80%). Utilising furfural, accessible from lignocellulosic biomass *via* acid-mediated dehydration,^25^ as a starting material is generally preferred, as both furfuryl alcohol and furoic acid are derived from it. Furfuryl alcohol is obtained *via* hydrogenation of furfural, while furoic acid is produced through oxidation.^43^

#### 2.1.2. Heterogeneous photosensitizers

A drawback of the photocatalytic pathway is the poor recyclability of organic photosensitizers as they are typically homogeneously dissolved in the reaction mixture and therefore require additional separation steps during work-up. To address this limitation, efforts have been made to develop heterogeneous photosensitizers, that can be readily separated from the reaction mixture *via* centrifugation or filtration.

Mesoporous carbon nitride (SGCN) has been reported as an efficient polymeric organic semiconductor photocatalyst, promoting the photooxidation of furfural under simulated solar light to yield maleic anhydride (MAnh, 42%) and HFO (33%) at furfural conversions exceeding 95%.^113^ Photooxidation of furfural has also been demonstrated using sulphated graphitic carbon nitride (g-C_3_N_4_), where product selectivity strongly depended on the solvent, oxidant, and light source. Under optimized conditions, HFO was obtained in 39% yield, accompanied by 61% furan.^114^

Another photooxidation approach employs amphiphilic star block copolymers with a Pd-contained core synthesized *via* atom transfer radical polymerisation (ATRP). These materials were found to be highly efficient sensitizers, achieving 90% conversion of furfural to HFO in methanol.^115^ Poly(*N*-isopropylacrylamide (PNIPAM)-based microgels with covalently attached rose bengal as the photosensitizer enabled over 99% conversion of furoic acid to HFO in aqueous media.^116^ Furthermore, a CdS/MOF photocatalyst (CdS 2.5%/NH_2_-MIL-125) has been used to oxidize furfural with O_2_ under sunlight, achieving 92% conversion with product selectivity of 52% toward HFO and 40% toward maleic acid (MA).^117^

Although these heterogeneous photocatalysts generally show lower selectivity toward HFO than homogeneous organic photosensitizers, they offer advantages in catalyst recovery and typically maintain high furfural conversions.

#### 2.1.3. Improving photooxidation by transition from batch to flow

Most studied described previously were conducted in batch reactors.^85,105,106,108–117^ Photooxidations in batch are inherently limited by poor light penetration and inefficient gas–liquid mass transfer.^118^ To overcome these limitations, photo-flow reactors have been developed that enforce short optical pathlengths and improved O_2_–liquid contact.^119^

Using a modified rotary evaporator (PhotoVap) George and coworkers demonstrated semi-continuous photooxidation of furfural to HFO in 2024 (Fig. 8A).^120,121^ In this setup, rotation of the reaction flask generates a thin film, creating a large surface area that facilitates both light penetration and O_2_ transfer into the solution. O_2_ was constantly bubbled through the reaction mixture while irradiation is provided by 360 W LEDs. Within 20 min, 92% conversion to HFO was achieved, after which a computer-controlled pump automatically empties and refills the reactor with new starting material. Using this system, semi-continuous production rates of 28 mmol h^−1^ were obtained.^120^

**Fig. 8:**
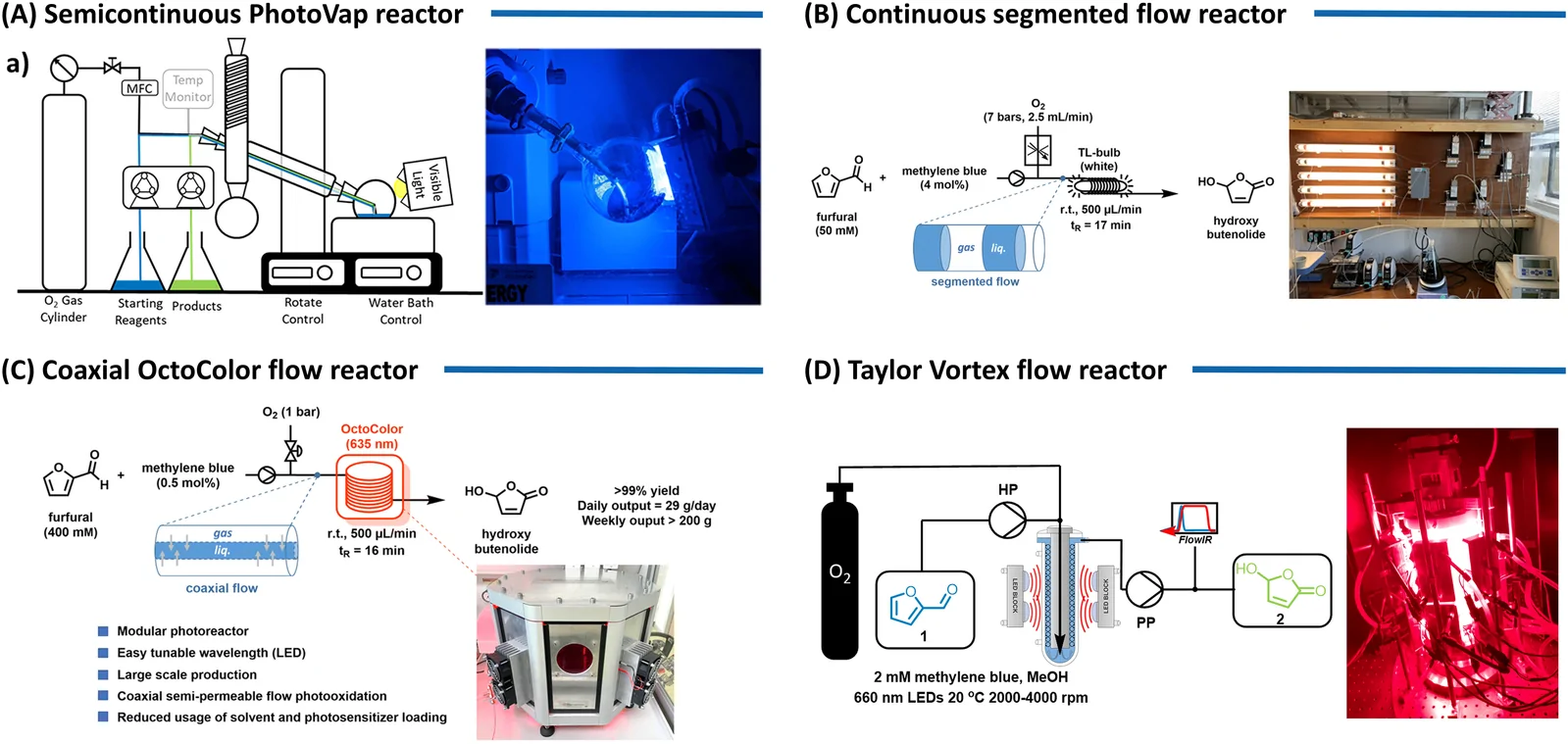
(Semi)continuous reactors designed for homogeneous photooxidation of furfural to HFO, (A) semi-continuous reactor design using a modified rotary evaporator,^120^ (B) continuous segmented flow reactor design,^107^ (C) continuous coaxial flow reactor design,^119^ (D) Taylor vortex flow reactor design.^120^ Adapted from ref. 107, 119, and 120 under the respective licences: CC BY-NC 4.0 (AAAS, © 2020), CC BY-NC 3.0 (RSC, © 2022), and CC BY 4.0 (ACS, © 2024).

The Feringa group designed a segmented flow reactor for continuous HFO production in 2020 (Fig. 8B). In this design, fluorinated ethylene propylene (FEP) tubing was coiled around an 18 W TL bulb. Mixing of the O_2_ stream and substrate solution in a T-piece generated a segmented gas–liquid flow. By operating five reactors in parallel, they were able to continuously produce HFO with a productivity of 7.5 mmol h^−1^.^107^ In a subsequent study (2021), our group reported a coaxial flow reactor that provided a constant O_2_ supply through a semi-permeable inner tube containing the flowing substrate solution (Fig. 8C). This configuration increased the productivity to 12 mmol h^−1^ and enabled quantitative production of more than 200 g of HFO per week.^119^

A notable advance was later reported by George and coworkers^120^ in 2024 through the deployment of Taylor Vortex flow reactors for the continuous photooxidation of furfural (Fig. 8D). These reactors employ a rotor-stator design consisting of a rotating inner cylinder and a stationary outer tube, generating Taylor vortices that significantly enhance micromixing and gas–liquid contact. This work enabled the first kilogram-per-day photochemical production of HFO, achieving yields of approximately 85% and space-time yield of ∼6 g day^−1^ mL^−1^.^120^

A drawback of the photocatalytic pathway is, however, the poor recyclability of the homogeneous photosensitizers and their difficult separation from the product after reaction. Efforts have therefore been directed toward the development of flow reactors employing heterogeneous photocatalysts. In 2019, Valverde *et al.*^122^ immobilized rose bengal on a polymeric material containing imidazolium fragments. These materials efficiently catalysed the photooxidation of 2-furoic acid to HFO and were implemented in a continuous mini-flow photocatalytic process.^122^ More recently in 2026, He *et al.*^93^ packed tubular photoreactors with PCN-222, a MOF-based photocatalyst, achieving near-quantitative conversion of 2-furoic acid into HFO with space time yield of 0.15 g day^−1^ mL^−1^.^93^

### 2.2. Electrocatalytic oxidation

Electrocatalytic oxidation of furfural to HFO has received increasing attention as a potentially sustainable approach, as these reactions can operate under relatively mild conditions and use water (H_2_O) as the oxygen source. However, achieving selective formation of HFO *via* electrooxidation remains challenging. In many reports, it appears only as an intermediate *en route* to the more oxidized product MA^123,124^ or the reaction is directed towards 2-furoic acid.

The first report describing selective electrooxidation of furfural to HFO was in 2019 by Wu *et al.*^125^ Using CuS nanosheets as the electrocatalyst in a ternary electrolyte consisting of triethylammonium nitrate ([Et_3_NH]NO_3_), acetonitrile (MeCN) and H_2_O, they achieved a HFO selectivity of 84% at a furfural conversion of 70%. The reaction proceeds through the generation of reactive hydroxyl species (OH˙) from H_2_O, which oxidize furfural *via* surface-mediated pathways involving C–C bond cleavage, ring-opening, intramolecular rearrangement, and isomerization (Scheme 3).^125^ From this mechanism it becomes evident why selectivity is difficult: HFO is a highly reactive intermediate that can readily undergo further oxidation to MA under strongly oxidative conditions.

**Scheme 3:**
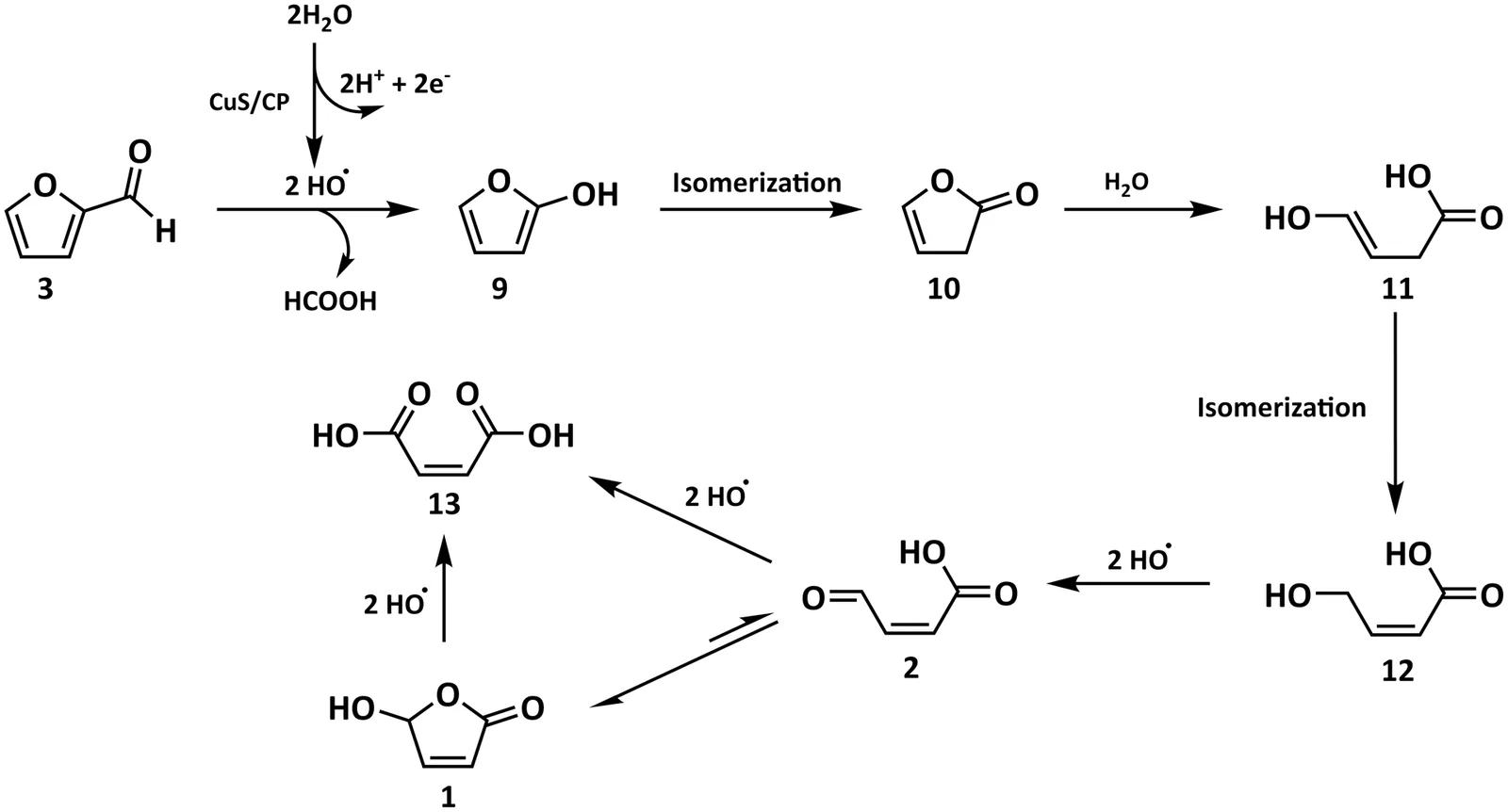
Proposed reaction mechanism for electrochemical oxidation of furfural to HFO over the CuS/CP electrode in the [Et_3_NH]NO_3_–MeCN–H_2_O anolyte.^125^

In the same year, the Holewinski group investigated the electrooxidation of furfural on carbon-supported platinum electrodes in 0.25 M HClO_4_. Their study provided valuable insight into the influence of the applied potential on product distribution. At lower potentials (0.9–1.0 V *vs*. RHE), partial oxidation products such as 2-furoic acid and 5-hydrofuroic acid dominated. Increasing the potential (≥1.2 V *vs*. RHE) shifted the selectivity toward HFO, accompanied by the formation of MA, while potentials above 1.3 V resulted in overoxidation to CO_2_.^126^ These findings highlight that careful control of the applied potential can aid in tuning the selectivity; however, the strong adsorption and high catalytic activity of Pt surfaces promote continuous oxidation, leading to poor selectivity toward HFO.^126^

Subsequent studies therefore focused on tuning catalyst activity through compositional and structural modification. For example, Bi-doped PbO_*x*_ catalysts were reported to suppress highly oxidative Pb^4+^ species and improve carbon efficiency, although MA remained the dominant product.^123^ Similarly, MOF-derived MnCeO_*x*_ catalysts exploit oxygen vacancies to enhance adsorption and catalytic activity, achieving MA yields of 67%.^124^ In parallel, bimetallic oxide systems such as Mn_*x*_Zn_1−*x*_O were investigated to balance catalytic activity and oxidation control by modifying the electronic structure of the catalyst surface, thereby reducing excessive oxidation and CO_2_ formation.^127^ Despite these advances, most of these systems still favour further oxidation of HFO rather than its selective accumulation.

More recently, in 2025 Zhu *et al.*^128^ reported highly efficient electrooxidation of furfural to HFO using metal-free mesoporous polymer catalysts. The best performance was obtained with mesoporous polydopamine (*meso*-PDA), synthesized through a block-copolymer-templated self-assembly strategy using dopamine as monomer. Under optimized conditions, the system achieved a faradaic efficiency of 93%, HFO selectivity of 96%, and furfural conversion of 80%. In this case, an adsorption-controlled mechanism was proposed, in which balanced adsorption of both furfural and hydroxyl species enhanced their interaction and promotes selective HFO formation.^128^ This shift from activity-driven to adsorption-controlled catalysis represents a significant advancement in the field.

A broader overview of catalysts reported for electrochemical furfural oxidation is summarized in Table 1. Overall, these studies demonstrate a gradual transition from activity-driven catalytic systems toward more selective and controlled approaches. Nevertheless, two key challenges remain. First, many catalysts either promote excessive oxidation or lack sufficient control over intermediate stability, preventing the selective accumulation of HFO (Scheme 4). Second, the wide diversity of catalyst systems reported in the literature indicates that a clear understanding of catalyst design principles for selective HFO formation is still lacking. Furthermore, long-term stability studies of several catalyst systems, particularly polymer-based materials, remain scarce.

**Table 1:** Examples of different methods of producing 5-hydroxy-2(5*H*)-furanone within literature

Catalyst	Substrate	Method	Solvent	Conditions	C [%]/Y^a^ [%]	Ref.
Methylene blue	Furfural	Photocatalysis	MeOH	Light, O_2_	Quantitative	54
Rose bengal	2-Furoic acid	Photocatalysis	MeOH	Light, O_2_, 25 °C	Quantitative	111
Rose bengal	Furfuryl alcohol	Photocatalysis	MeCN	Light, O_2_, 0–5 °C	Quantitative	112
CdS 2.5%/NH_2_-MIL-125	Furfural	Heterogeneous photocatalysis	MeCN/H_2_O	Sunlight, O_2_	92/52	117
Methylene blue	Furfural	Flow photocatalysis	MeOH	Light, O_2_	85%/6 g day^−1^ mL^−1^	120
PCN-222 (MOF)	Furoic acid	Heterogeneous flow photocatalysis	2-MeTHF	Light, O_2_, 500 mM	Quantitative/0.15 g day^−1^ mL^−1^	93
CuS	Furfural	Electrocatalysis	MeCN/H_2_O	[Et_3_NH]NO_3_ at 1.5–1.6 V *vs*. Ag/Ag^+^, 25 °C	70/59	125
Meso-PDA	Furfural	Electrocatalysis	MeCN/H_2_O	BmimBF4, 1.6–1.9 V *vs*. Ag/Ag^+^	80/77	128
Vanadium 2-oxonaphtenate	Furfural	Homogeneous catalysis	H_2_O/acetone	Molar ratio of furfural : H_2_O_2_ : catalyst : hydroquinone = 1 : 3 : 0.003 : 0.0003, 6 h, 55–60 °C	N.D./90	129
TS-1	Furan	Heterogeneous catalysis	MeCN	H_2_O_2_ (2.4 eq.), 8 h, 0 °C-RT	Quantitative	130
TS-1	Furfural	Heterogeneous catalysis	H_2_O	H_2_O_2_ (16 eq.), 8 h, RT	Quantitative	99
MOF	Furfural	Heterogeneous catalysis	MeCN/H_2_O	H_2_O_2_	99/53	131
—	Furan	Stoichiometric oxidation	H_2_O	Oxone® (1.25 eq.), 8 h 0 °C, then 8 h RT	N.D./75	132
—	Furfural	Ultrasound irradiation	H_2_O	Ultrasound irradiation: 75 kHz, 80 W, 42 °C	54/37	133

a STY given for flow systems.

**Scheme 4:**
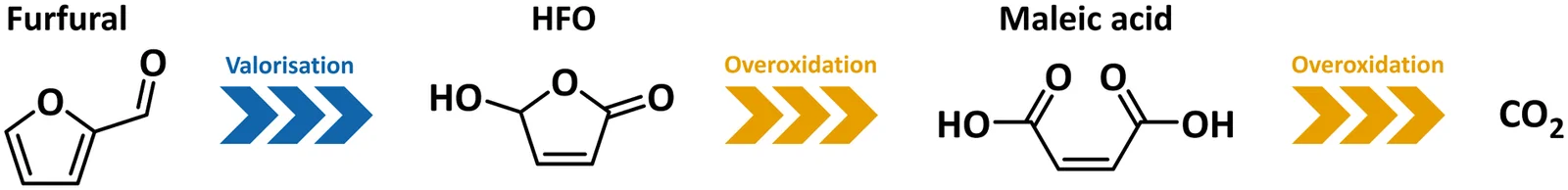
Valorisation of furfural to 5-hydroxybutenolide (HFO) and common overoxidation products.

### 2.3. Homogeneous catalysis

Great efforts toward homogeneous catalytic oxidation of furfural and furan with H_2_O_2_ have been reported by the Poskonin group.^129,134–143^ In this system (Scheme 5), first an unstable peroxide intermediate 14 is generated, which undergoes a Baeyer–Villiger rearrangement to afford 2-formyloxyfuran 15. Hydrolysis of compound 15 releases acetic acid and hydroxyfuran 9, which is not isolated due to its rapid tautomerization into lactones 10 and 16. Formation of these lactones competes with the formation of HFO 1*via* hydroperoxide 17.^129,144^

**Scheme 5:**
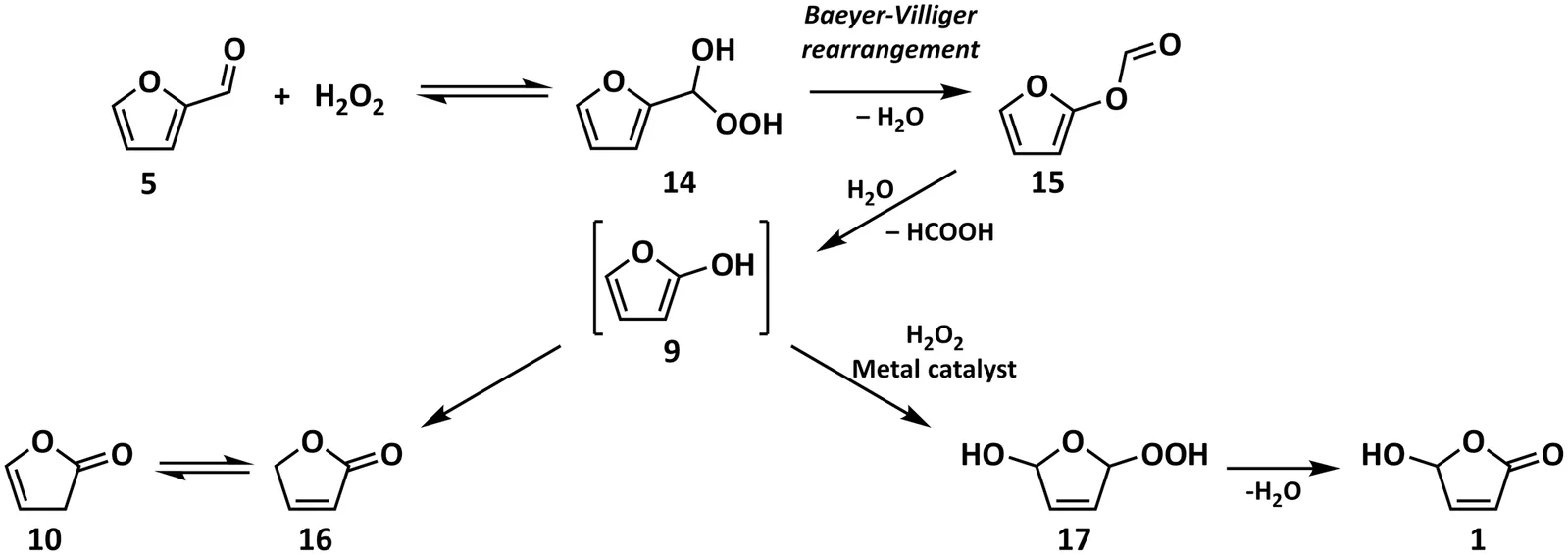
Oxidation of furfural with H_2_O_2_. Metal catalysts like vanadium 2-oxonaphtene promote the pathway towards HFO, where catalyst-free systems favour the formation of lactones 10 and 16.^129^

In catalyst-free systems, the conversion of 9 into lactones 10 and 16 is favoured, and HFO 1 is only obtained as a minor product with yields less than 4%.^144^ However, in the presence of catalysts such as vanadium(iv), vanadium(v), molybdenum(vi), or SeO_2_, hydroxyfuran 9 is rapidly oxidized to HFO 1, thereby outcompeting its tautomerization into lactones 10 and 16. These catalysts form peroxo-species with H_2_O_2_ that are significantly stronger oxidants than H_2_O_2_ itself.^144^ Under optimized conditions, oxidation of furfural with 30% H_2_O_2_ in the presence of catalytic amounts of vanadium 2-oxonaphtenate and hydroquinone in a H_2_O-acetone mixture (molar ratio of furfural : H_2_O_2_ : catalyst : hydroquinone = 1 : 3 : 0.003 : 0.0003) afforded HFO in yields up to 90%. The reactions were typically performed at 55–60 °C for 6 h, after which the reaction mixture was neutralized, concentrated, and extracted with diethyl ether (Et_2_O) to isolate HFO 1.^129^

### 2.4. Heterogeneous catalysis

#### 2.4.1. TS-1

Thermocatalytic oxidation of furanic compounds using H_2_O_2_ and titanium silicate-1 (TS-1) has emerged as a heterogeneous strategy for accessing oxygenated platform molecules like HFO.

Kumar *et al.*^130^ first reported this transformation, preparing HFO 1 from furan 18 using H_2_O_2_ (2.4 eq.) and TS-1 in MeCN at 0 °C for 8 h, after which the reaction mixture was allowed to warm to room temperature (Scheme 6A). Under these conditions, an isolated yield of 98% was obtained. The authors hypothesized that ^1^O_2_ is generated through hydroperoxo- or peroxo-titanium species, leading to endoperoxide formation on the furan ring and ultimately yielding HFO.^130^

**Scheme 6:**
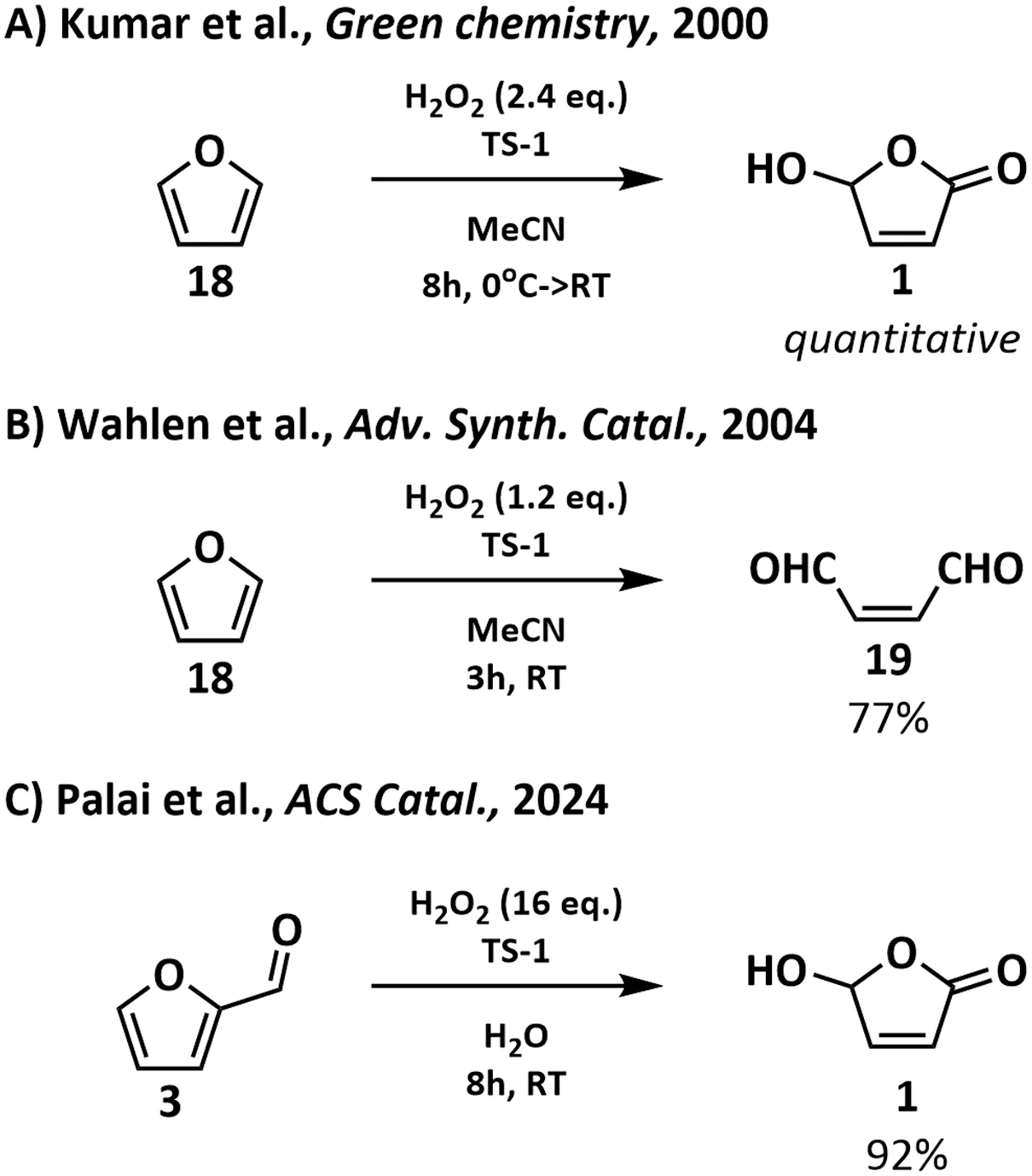
Thermocatalytic oxidation of furan and furfural using hydrogen peroxide and TS-1, (A) furan oxidation to hydroxybutenolide,^130^ (B) furan oxidation to maleic dialdehyde,^145^ (C) furfural oxidation to HFO in water.^99^

This procedure was later reproduced by Chavan *et al.*^146^ on a 25 g scale, affording HFO in 76% isolated yield. Under similar conditions but without cooling the reaction mixture to 0 °C prior to H_2_O_2_ addition, Wahlen *et al.*^145^ instead reported formation of maleic dialdehyde 19 with 77% selectivity (Scheme 6B).^145^ These results highlight the strong influence of reaction conditions, particularly temperature, on the final product distribution.

HFO 1 synthesis from furfural 3 using TS-1 and H_2_O_2_ was later reported by Palai *et al.*^99^ in aqueous media. In this study, an excess of H_2_O_2_ (16 equivalents) was used to accelerate the reaction toward HFO formation, of which 3.1 equivalents were consumed during the reaction. Under these conditions, quantitative conversion of furfural was reported, with 92% yield of HFO together with formation of 93% formic acid (Scheme 6C). The proposed mechanism is shown in Scheme 7.^99^ TS-1 contains isolated tetrahedral Ti sites capable of activating aqueous H_2_O_2_. The Ti(iv) centres form electrophilic hydroperoxo- or peroxo-species that efficiently epoxidize electron-rich C=C bonds. Applied to furanic substrates, this pathway proceeds *via* epoxidation of the furan ring, followed by rearrangement to (*Z*)-4-oxopent-2-enedial (4O2EL, 20) that subsequently converts to HFO 1 through non-catalytic Baeyer–Villiger oxidation. This process requires ambient temperature, since its further oxidation towards MA is known to be promoted at more elevated temperatures.^147^ This was also found in furfural oxidation over perovskites at 150 °C and 30 bar in O_2_ atmosphere, where HFO was only observed as an intermediate towards MA.^148^

**Scheme 7:**
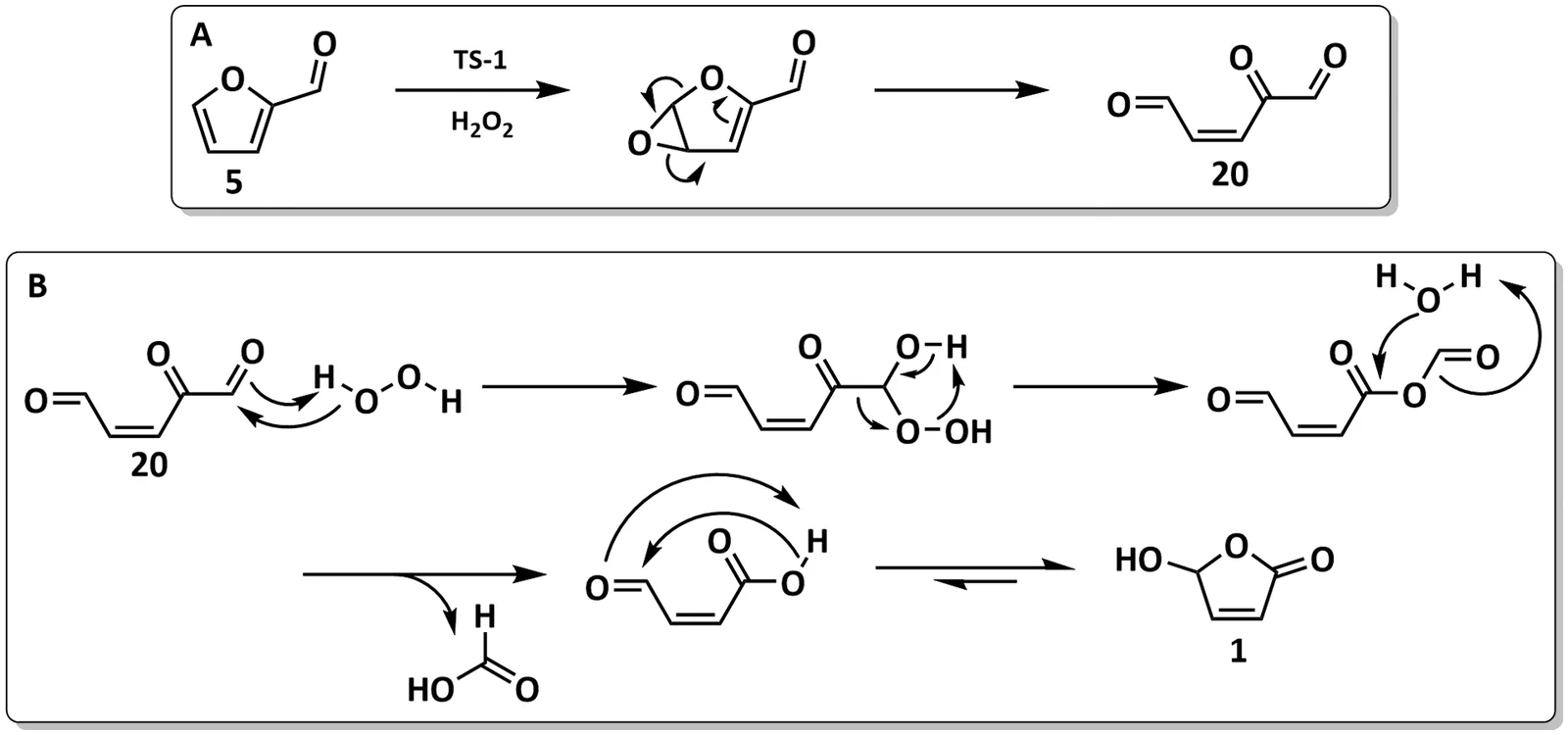
Mechanism for HFO from furfural in water with hydrogen peroxide and TS-1, (A) reaction of furfural with activated H_2_O_2_ on TS-1 surface, (B) Baeyer–Villiger oxidation with free H_2_O_2_ in solvent.^99^

#### 2.4.2. MOFs

More recently, Li and coworkers reported the oxidation of furfural to HFO using a dual-coordination-sphere porphyrin-based covalent organic framework.^131^ The catalyst was synthesized through solvothermal condensation of 5,10,15,20-tetrakis(4-aminophenyl) porphyrin and thieno[3,2-*b*]thiophene-2,5-dicarbaldehyde, followed by iron metalation. In this system, the iron-porphyrin centre acts as the primary active site, while the sulphur-rich linkers form a biomimetic catalytic pocket that functions as the second coordination sphere. Using H_2_O_2_ as the oxidant in a H_2_O–MeCN mixture, the system achieved 99% conversion of furfural with a HFO yield of 53%. The catalytic pocket synergistically promotes the activation of H_2_O_2_ to generate hydroxyl radicals while simultaneously stabilizing reactive intermediates, thereby enhancing catalytic efficiency.^131^

### 2.5. Stoichiometric oxidation

In 1997 Boukouvalas *et al.* reported a for the direct synthesis of HFO from 2-furanone 16 over 2-trialkylsilyloxyfurans 21 (Scheme 8).^149^ Their approach was based on the capacity of siloxyfurans to undergo rapid oxidative cleavage upon treatment with dimethyldioxirane, yielding (*Z*)-4-oxo-2-alkenoates 22. Subsequent hydrolysis affords HFO 1 in high yield.^149^

**Scheme 8:**
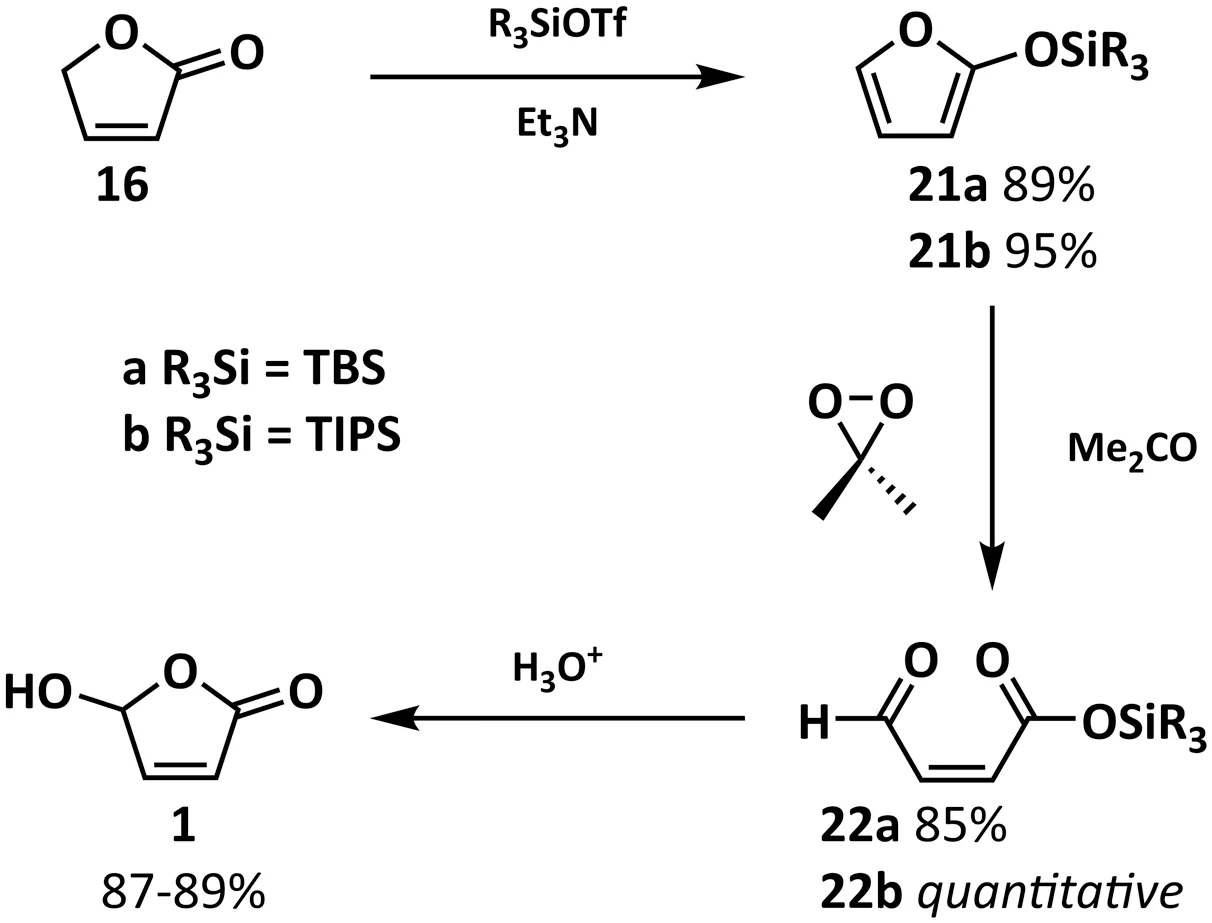
Oxidative cleavage of siloxyfurans 21 upon treatment with dimethyldioxirane and subsequent hydrolysis to HFO 1 (TBS = *tert*-butyldimethylsilyl, TIPS = triisopropylsilyl).^149^

In 2021 Chavan *et al.*^132^ continued the research on stoichiometric oxidations by using Oxone® as the oxidant in H_2_O (Scheme 9). When the reaction on furan 18 was performed on a 50 g scale, an isolated yield of 75% was obtained. Due to the low cost of Oxone® and the use of H_2_O as the reaction medium, the authors proposed this method as an industrially relevant, catalyst free, and environmentally benign protocol for the production of HFO.^132^ However, Oxone® must be used in stoichiometric quantities, which leads to the formation of large amounts of inorganic sulfate salts. As a result, the process generates significant waste relative to the amount of product formed.

**Scheme 9:**
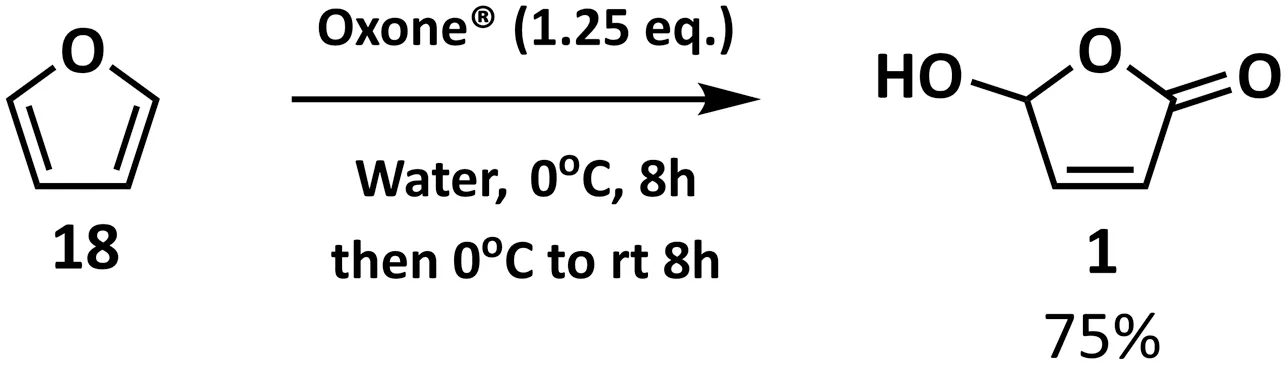
Stoichiometric oxidation of furan using Oxone® in water.^132^

Interestingly, Ayoub *et al.*^133^ reported in 2022 on catalyst-free oxidation of furfural to MA using high-frequency ultrasonication.^133^ Ultrasound irradiation can generate highly reactive radical species, such as hydroxyl radicals, through localized high temperatures and pressures created during cavitation bubble collapse in solution. Under these conditions, a biphasic mixture of furfural and aqueous H_2_O_2_ could be oxidized to MA on gram scale with good energy efficiency. In this study, HFO was identified as an intermediate. However, when operating at lower ultrasound frequency (50 kHz) and 80 W power, the authors reported a selectivity of 68% toward HFO at a furfural conversion of 54% (Scheme 10).^133^

**Scheme 10:**
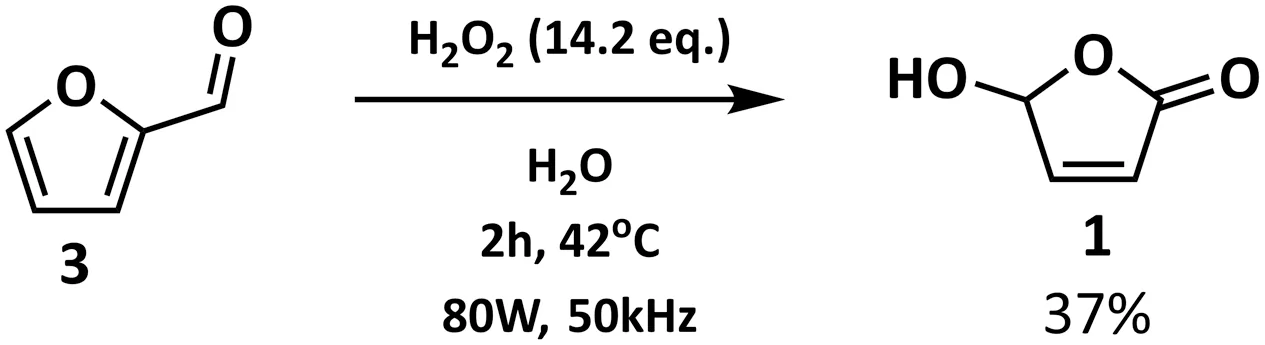
Ultrasound irradiation for the oxidation of furfural to HFO with H_2_O_2_.^133^

### 2.6. Concluding remarks on the production of HFO

The oxidation of bio-based furanic compounds into value-added products has attracted significant interest over the past decades. A variety of catalytic and non-catalytic approaches have been explored for the synthesis of HFO, each with distinct advantages and limitations. The main strategies discussed in this section, including photocatalytic, electrocatalytic, homogeneous, heterogeneous, and catalyst-free approaches, are summarized and compared in Table 1.

Among the various strategies reported for the synthesis of HFO, photocatalytic oxidation remains the most widely studied approach. In these systems, organic photosensitizers activate molecular O_2_ to generate ^1^O_2_, enabling efficient oxidation of furanic substrates under relatively mild conditions. These reactions often proceed with high selectivity and yields when overheating caused by irradiation is avoided. Advances in reactor design, particularly the development of continuous photo-flow systems, have further improved these processes by overcoming limitations related to light penetration and O_2_ solubility. Despite these improvements, their homogeneous nature causes challenges related to photosensitizer recovery and recyclability. Immobilized photosensitizers and heterogeneous photocatalysts have therefore been explored as alternatives, although these systems often show lower productivity.

Electrocatalytic oxidation represents another promising strategy, as it uses H_2_O as oxygen source and allows oxidation strength to be tuned through the applied potential. However, controlling overoxidation remains difficult due to the high reactivity of intermediate species, and current systems often suffer from limited selectivity or moderate substrate conversion. Homogeneous catalytic oxidation using H_2_O_2_ has also proven effective for HFO production, particularly in the presence of transition-metal catalysts such as vanadium complexes. Although these systems can achieve high yields, the need for catalyst separation and recovery remains a practical limitation. Heterogeneous catalytic systems, such as TS-1, offer advantages in catalyst stability and recyclability. In these systems, H_2_O_2_ is activated at isolated metal sites to promote selective oxidation of furanic substrates. TS-1-based systems have demonstrated promising efficiency for HFO formation and may represent a viable pathway toward industrial implementation, although most studies have so far been limited to laboratory or gram-scale reactions.

Overall, the selective oxidation of furanic compounds to HFO requires careful control of oxidation strength and reaction conditions to prevent overoxidation toward MA or other by-products. Continued progress in catalyst design, reaction engineering, and scalable reactor technologies will therefore be essential for developing economically viable and sustainable processes for HFO production.

## 3. Functionalisation and derivatisation of 5-hydroxy-2(5*H*)-furanone (HFO)

Over the years a wide variety of transformations have been reported on 5-hydroxy-2(5*H*)-furanone (HFO, 5-hydroxybutenolide, 1). HFO is a highly reactive chiral compound bearing various functional groups such as a hemiacetal-type hydroxyl group and α,β-unsaturated olefinic double bond. This structural arrangement allows for various transformations of the molecule *via e.g.*, oxidations, ring-opening reactions, ether- and esterifications, aminations and asymmetric transformations. These transformations are covered in detail in this section and illustrated in Fig. 9.

**Fig. 9:**
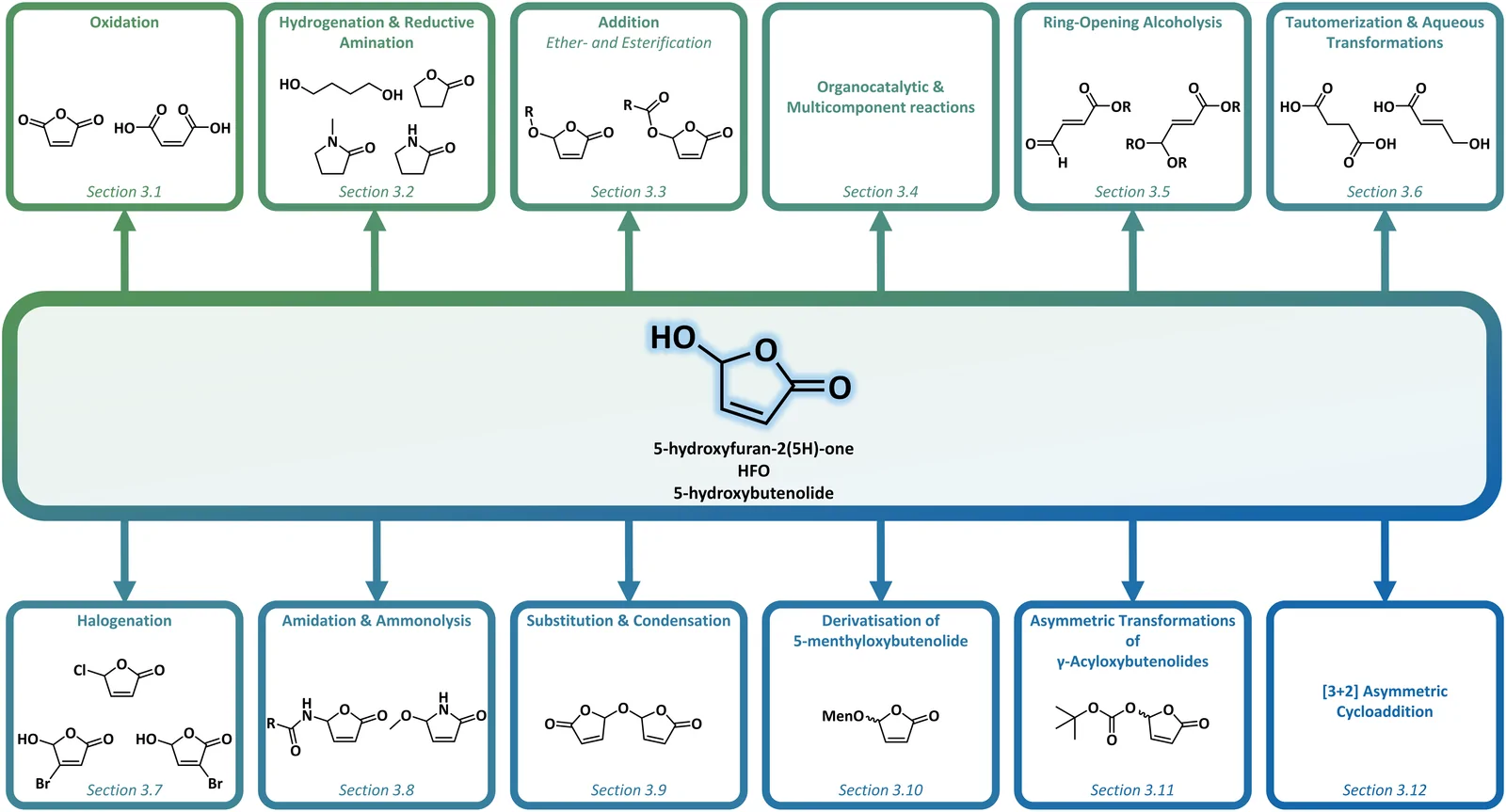
Transformations of HFO covered in the different sections of this section.

### 3.1. Oxidation of 5-hydroxybutenolide (HFO)

The development of green and efficient strategies for the conversion of HFO into value-added products, such as maleic anhydride (MAnh, 23) and maleic acid (MA, 13), is highly desirable from a sustainability perspective. MAnh is a versatile platform molecule widely used due to its high reactivity in Diels–Alder reactions, photochemical [2+2]-cycloadditions, and as a polymer building block. MA is primarily used for the production of fumaric acid, unsaturated polyester resins, surface coatings, plasticizers, copolymers and agricultural chemicals.^150^

A significant advance in this field was reported in 2022 by the Feringa group, who developed a TEMPO-mediated aerobic oxidation of HFO using an earth-abundant and cost-effective iron salt catalyst (10 mol%) in combination with TEMPO as a mediator (Scheme 11). Under mild conditions (O_2_ atmosphere in MeCN), full conversion to MAnh 23 was achieved with 98% selectivity at room temperature.^151^ This methodology provides a convenient and sustainable route to MAnh from renewable feedstocks, highlighting the potential of HFO as a green platform molecule.

**Scheme 11:**
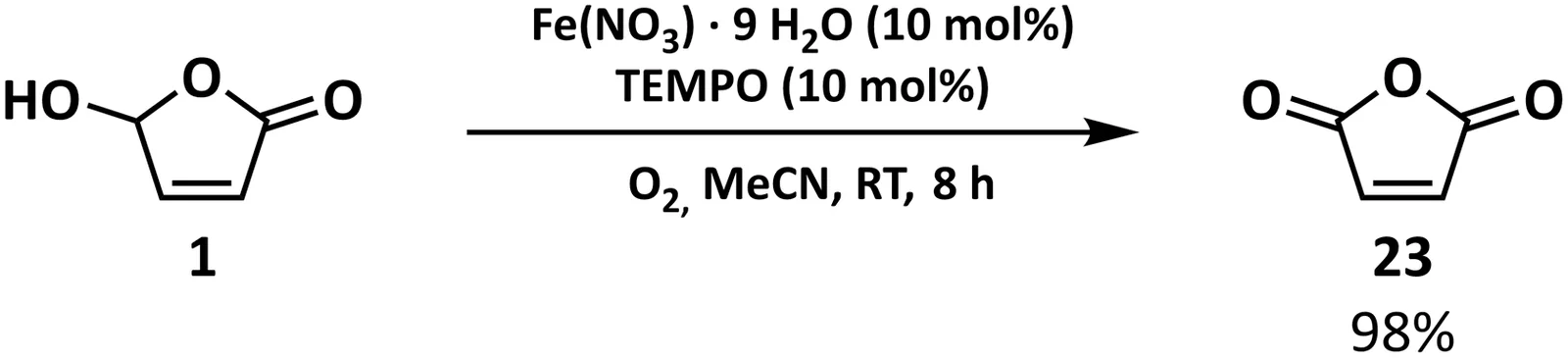
Batch aerobic oxidation of HFO to MAnh using the TEMPO/Fe(NO_3_)_3_ system under O_2_, as reported by the Feringa group (2022).^151^

Building on this work, Mortier *et al.*^152^ (2025) revisited this catalytic system under batch conditions using ethyl acetate as a greener solvent. At 50 °C and 7.5 mol% catalyst and mediator loading under 1 atm O_2_, high conversion (98%) was achieved (Scheme 12).^152^ Increasing the temperature to 70 °C resulted in a decrease in yield (90%), which was attributed to limitations in O_2_ mass transfer. Specifically, the reduced solubility of O_2_ at elevated temperatures, combined with limited gas–liquid interfacial area in batch reactors, hampers efficient oxidation. This study highlights the importance of controlling O_2_ availability in aerobic oxidation processes.

**Scheme 12:**
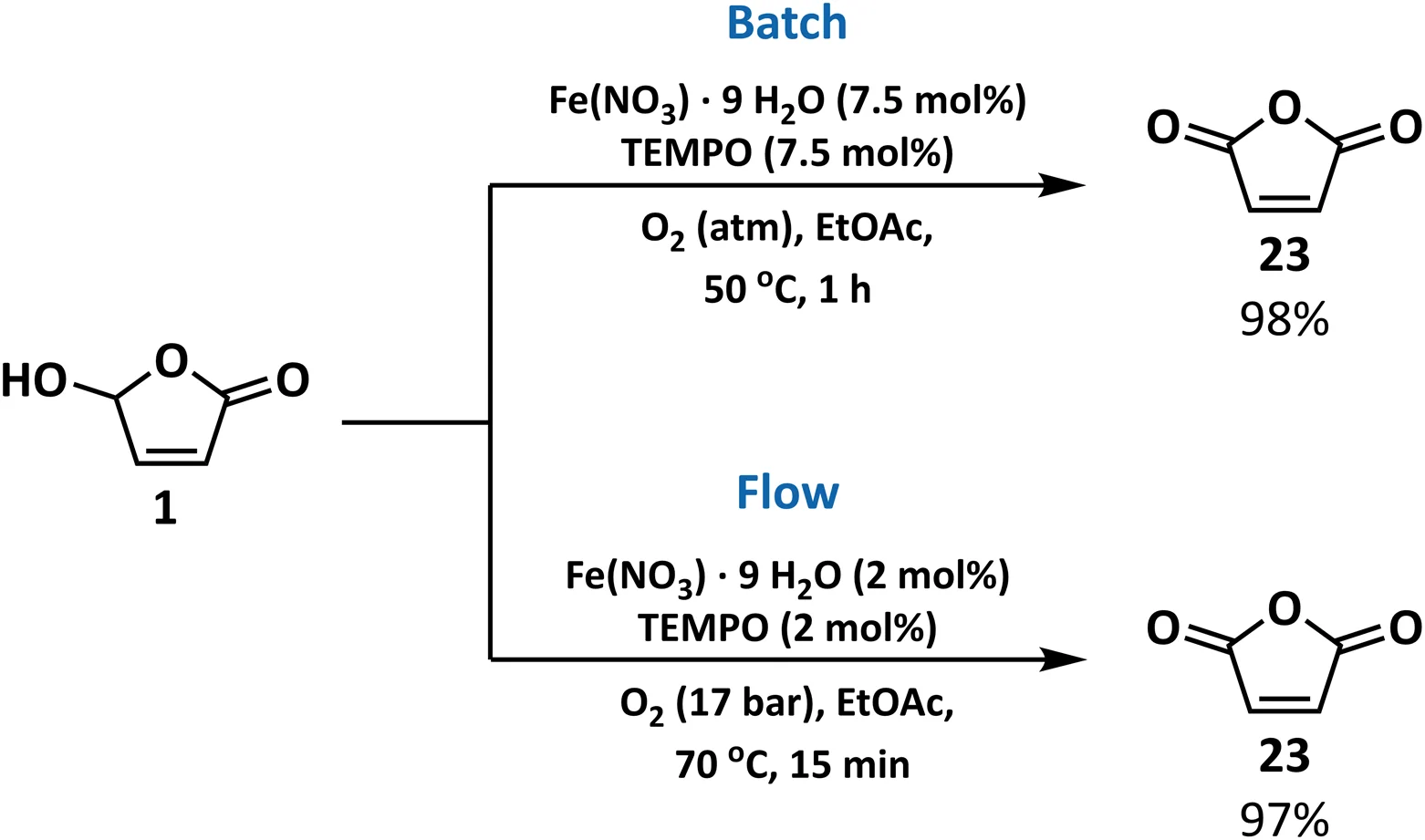
Batch *vs.* continuous-flow aerobic oxidation of HFO to MAnh, based on the study of Mortier *et al.* (2025).^152^

To address these limitations, the authors developed a continuous-flow strategy (Scheme 12).^152^ Under Taylor flow conditions, efficient gas–liquid mixing ensures a constant O_2_ supply due to the high interfacial surface area. In addition, the application of back pressure allows operation above the solvent's boiling point while maintaining the liquid phase, thereby enhancing both reaction rate and safety. Under optimized conditions (0.2 M substrate, 70 °C), complete conversion of HFO to MAnh was achieved within 15 min.

Notably, crude, photochemically generated HFO could be directly used without purification, affording MAnh in up to 98% yield.^152^ This telescoped process represents a highly efficient and scalable route for the valorisation of renewable feedstocks. Importantly, the authors stress that complete removal of residual methanol is essential prior to oxidation when HFO is used directly from the photochemical reaction, as even trace amounts inhibit the process.^152^

#### 3.1.1. Heterogeneous and alternative oxidation systems

Despite the advantages of homogeneous catalytic systems, the development of heterogeneous catalysts for HFO oxidation remains relatively underexplored. Catalyst recyclability and reusability are key factors for achieving high turnover numbers (TON) and turnover frequencies (TOF), making heterogeneous systems particularly attractive.

In 2024, Palai *et al.*^99^ demonstrated that oxidation of HFO using H_2_O_2_ over a TS-1 catalyst at 80 °C affords MAnh 23 and MA 13 (quantified together as MA) in up to 93% yield within 2 h (Scheme 13). Other Lewis acidic zeolites, including Nb-Beta, Ti-Beta, and Zr-Beta, also exhibited moderate to good activity (28–86% yields).^99^ In these systems, oxidation is proposed to proceed *via* metal-peroxo species formed *in situ* on the catalyst surface. Interestingly, the reaction was found to be relatively insensitive to the exact structure of the peroxo species, allowing the use of molecular O_2_ as an alternative oxidant (Scheme 13). For example, Pt/C catalysts operating at 100 °C under 0.9 MPa O_2_ afforded MA in 81% yield after 12 h, demonstrating the versatility of both peroxide- and oxygen-based oxidation pathways.^99^

**Scheme 13:**
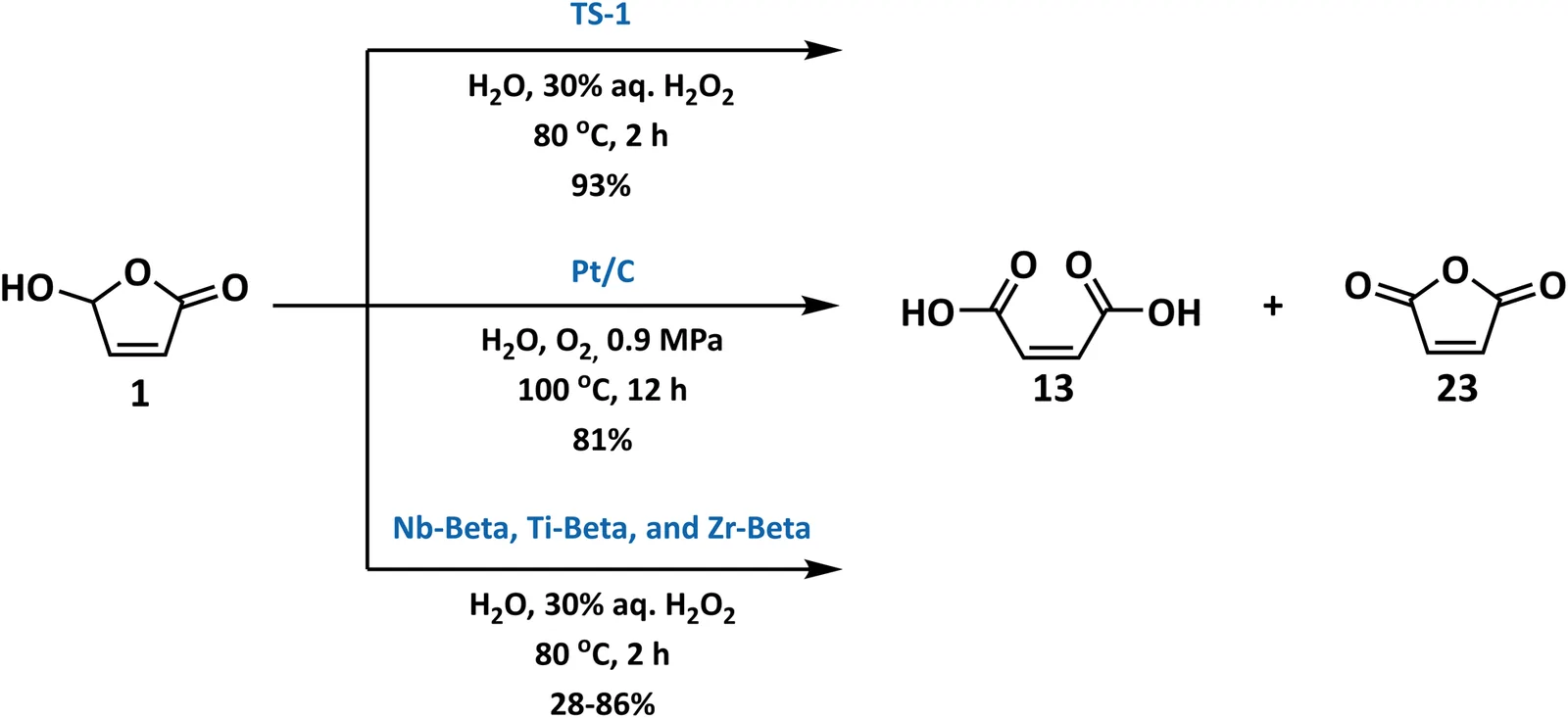
Heterogeneous mixed metal-oxide and Pt catalyst for HFO oxidation (Palai *et al.*, 2024).^99^

#### 3.1.2. Enzymatic and electrochemical oxidation

Biocatalytic approaches offer an attractive alternative due to their mild reaction conditions and high selectivity. In 2020, Thiyagarajan *et al.*^153^ reported that a laccase-TEMPO system enables highly selective aerobic oxidation of HFO to MA (Scheme 14). Under mild aqueous conditions (pH 4.5, 25 °C), quantitative conversion (99%) was achieved within 24 h without detectable byproducts.^153^

**Scheme 14:**
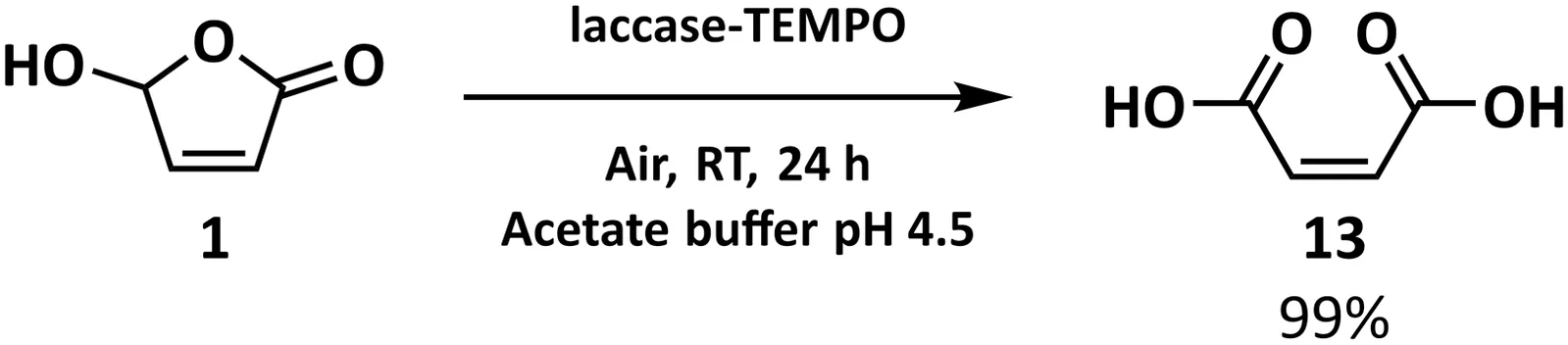
Enzymatic aerobic oxidation of HFO to MA (Thiyagarajan *et al.*, 2020).^153^

The laccase-TEMPO system involves the action of the copper-dependent oxidative enzyme (laccase) and the mediator TEMPO, which enables the oxidation of substrates that are not directly oxidized by the enzyme. TEMPO is first oxidized by laccase to its oxoammonium ion (TEMPO^+^). Subsequently, TEMPO^+^ oxidizes HFO to yield MA 13 and the hydroxylamine species (TEMPOH). TEMPO is then regenerated by comproportionation between TEMPOH and TEMPO^+^. The reduced laccase is reoxidised by molecular O_2_, enabling it to oxidize the TEMPO free radical, thereby closing the catalytic cycle.^153^

Thiyagarajan *et al.*^153^ also investigated electrocatalytic oxidation as a complementary strategy. In this approach, nitroxyl radicals such as 4-Acetamido-TEMPO (ACT) are electrochemically oxidized at a reticulated vitreous carbon (RVC) anode to generate the active oxoammonium species, which subsequently oxidizes HFO (Scheme 15).^153^ The ACT-mediated system demonstrated excellent performance, achieving 99% conversion and 90% yield of MA 13 at pH 10, although minor formation (7%) of fumaric acid 24 was observed, complicating product separation.

**Scheme 15:**
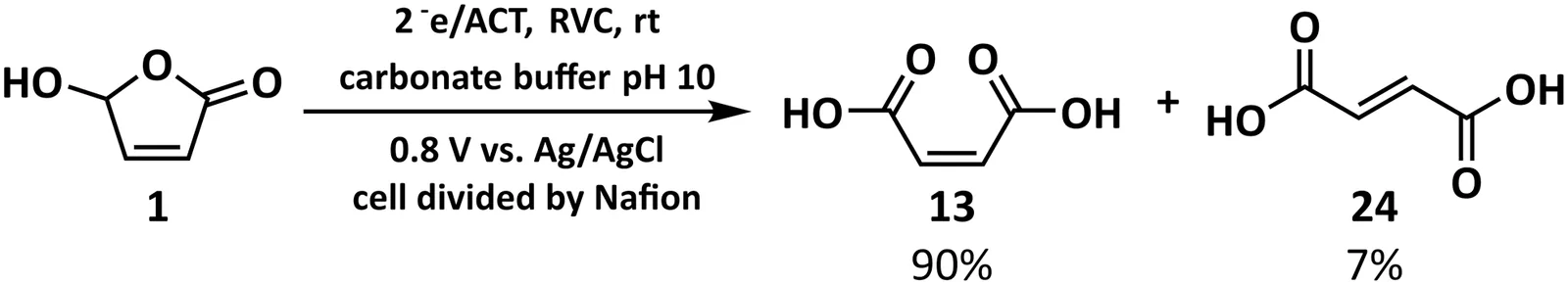
Enzymatic aerobic oxidation of HFO to MA.^153^

To improve selectivity and avoid additional purification steps, Lu *et al.*^154^ (2023) investigated the origin of fumaric acid formation and attributed it to base-catalysed isomerization of *cis*-intermediates at high pH (≈10). By lowering the pH to 8.5, fumaric acid formation was completely suppressed, and MA 13 was obtained in 90% yield with high selectivity (Scheme 16).^154^

**Scheme 16:**
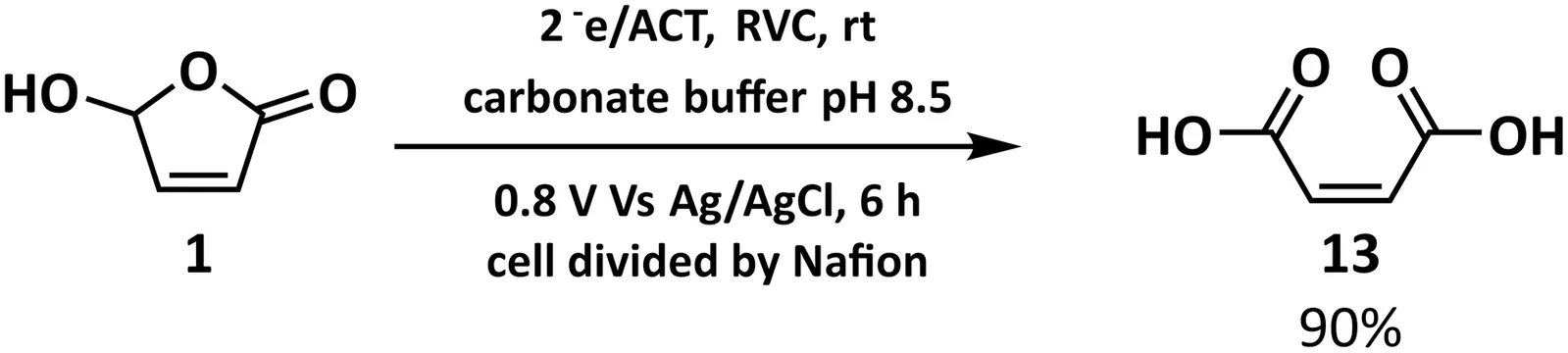
Electrochemical aerobic oxidation of HFO to MA (3).^154^

Earlier studies by the Poskonin group (2021) demonstrated that anodic oxidation of HFO in the presence of H_2_O_2_ affords MA 13 in moderate yield (63%), further highlighting the feasibility of electrochemical-peroxide hybrid systems (Scheme 17).^79^

**Scheme 17:**
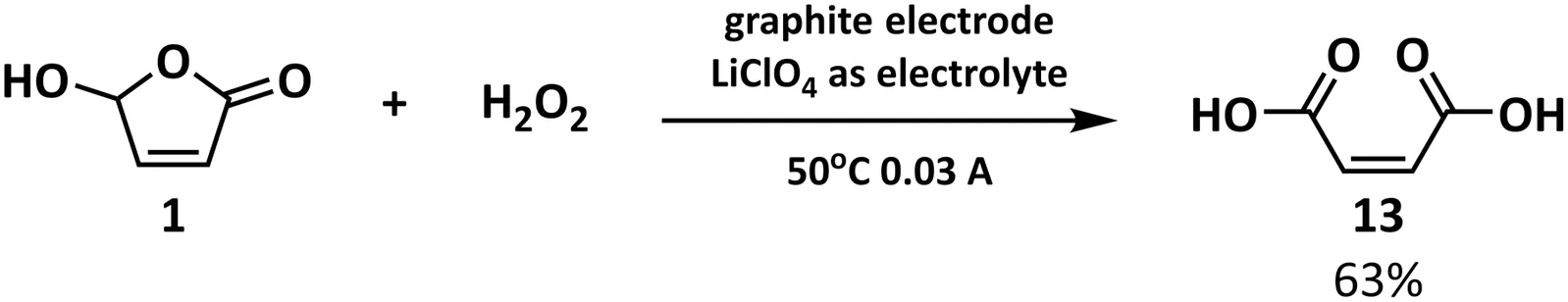
Electrochemical aerobic oxidation of HFO to MA (3).^79^

#### 3.1.3. Selectivity control and emerging strategies

More recently, Warthegau *et al.* (2026)^155^ demonstrated that product selectivity in peroxide-driven HFO oxidation can be effectively tuned between MA 13 and fumaric acid 24 through the use of quaternary ammonium-based salt additives (Scheme 18). For example, the use of aqueous betaine hydrochloride afforded MA in 96% yield with minimal formation of fumaric acid (4%). In contrast, deep eutectic solvent (DES) systems based on choline chloride (ChCl) and organic acids (OA) shifted the selectivity, yielding MA 13 (64%) alongside increased amounts of fumaric acid 24 (up to 36%).

**Scheme 18:**
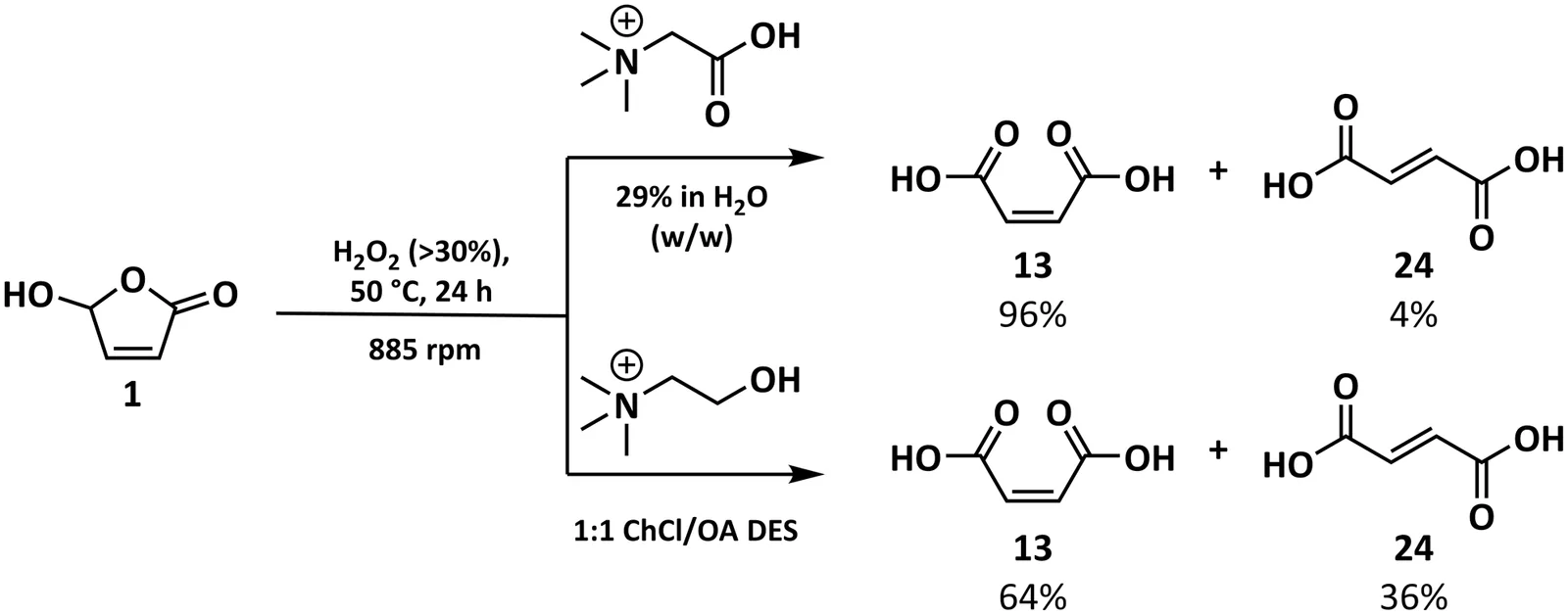
Effects of quaternary ammonium additives and Brønsted acids on product selectivity in HFO oxidation.^155^

Mechanistic investigations suggest that these additives stabilize zwitterionic intermediates *via* hydrogen-bonding networks, thereby facilitating bond rotation and influencing the distribution of geometric isomers. Importantly, time-resolved nuclear magnetic resonance (NMR) studies revealed that fumaric acid is not formed solely through isomerization of MA 13, but likely originates from ring-opened intermediates derived directly from HFO.^155^

### 3.2. Hydrogenation of 5-hydroxybutenolide (HFO)

In 2024, Palai *et al.*^99^ reported the selective hydrogenation of HFO over carbon-supported metal catalysts toward industrial C4 chemicals like γ-butyrolactone (GBL, 26) or 1,4-butanediol (BDO, 27), widely used as precursors for polymers (Scheme 19).^99^ Product selectivity was found to be strongly dependent on both the catalyst type and reaction conditions. Using Pt/C, selective hydrogenation of the C=C bond afforded 5-hydroxy- γ-butyrolactone (HGBL, 25) in 89% yield at 100 °C (Scheme 19A). In contrast, Ru/C enabled deeper hydrogenation, producing GBL 26 in 93% yield without detection of the HGBL 25 intermediate (Scheme 19B).^99^

**Scheme 19:**
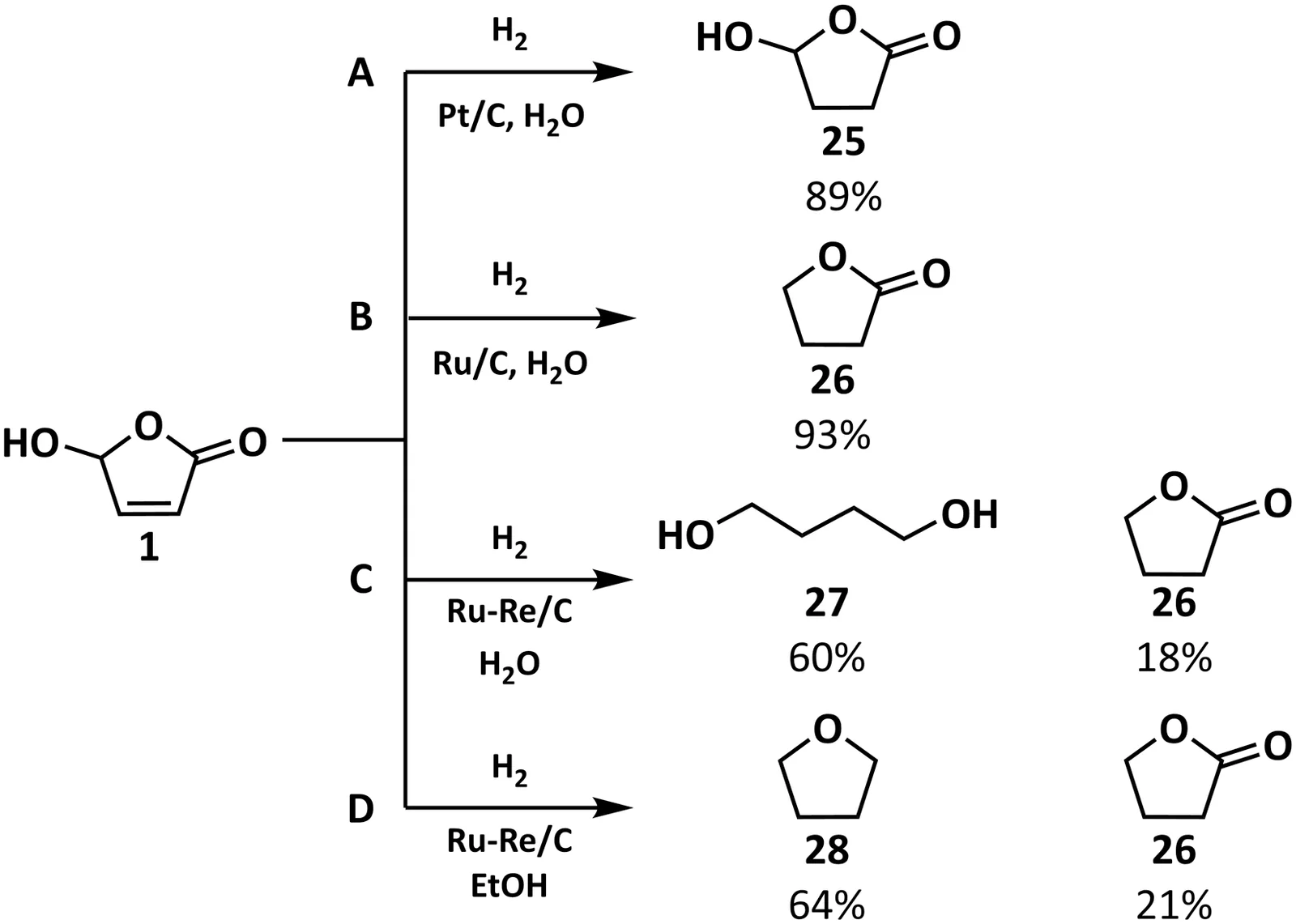
Hydrogenation of HFO leading to different products depending on the reaction conditions used.^99^

Further transformation to downstream products was achieved using a bimetallic Ru–Re/C catalyst. At 150 °C, BDO 27 was obtained in 60% yield after 24 h *via* hydrogenation of GBL, followed by dehydration to tetrahydrofuran (THF) (Scheme 19C). Monometallic Re/C showed no activity towards BDO 27, highlighting the synergistic role of Ru and Re.

Hydrogen temperature-programmed reduction (H_2_-TPR) analysis indicated that Ru promotes the reduction of rhenium oxide, generating active Re species. These species exhibit both acidity and oxophilicity, enabling hydrogenation *via* heterolytic H_2_ activation. Solvent effects were also significant: performing the reaction in ethanol promoted dehydration of BDO 27, yielding THF 28 in 64% along with GBL 26 (21%) and small quantities of ether byproducts (Scheme 19D).^99^

#### 3.2.1. Hydrogenation and reductive amination

Beyond oxygen-containing products, nitrogen-containing C4 compounds can be accessed *via* reductive aminolysis. In the presence of H_2_ and NH_3_, HFO undergoes ring-opening over Ru/C to yield γ-aminobutanoic acid (GABA, 28) and 2-pyrrolidone 29 (Scheme 20). After 1 h at 110 °C, GABA 28 was obtained in 71% yield, whereas prolonged reaction at 130 °C for 18 h afforded 2-pyrrolidone 29 in 67% yield.^99^

**Scheme 20:**
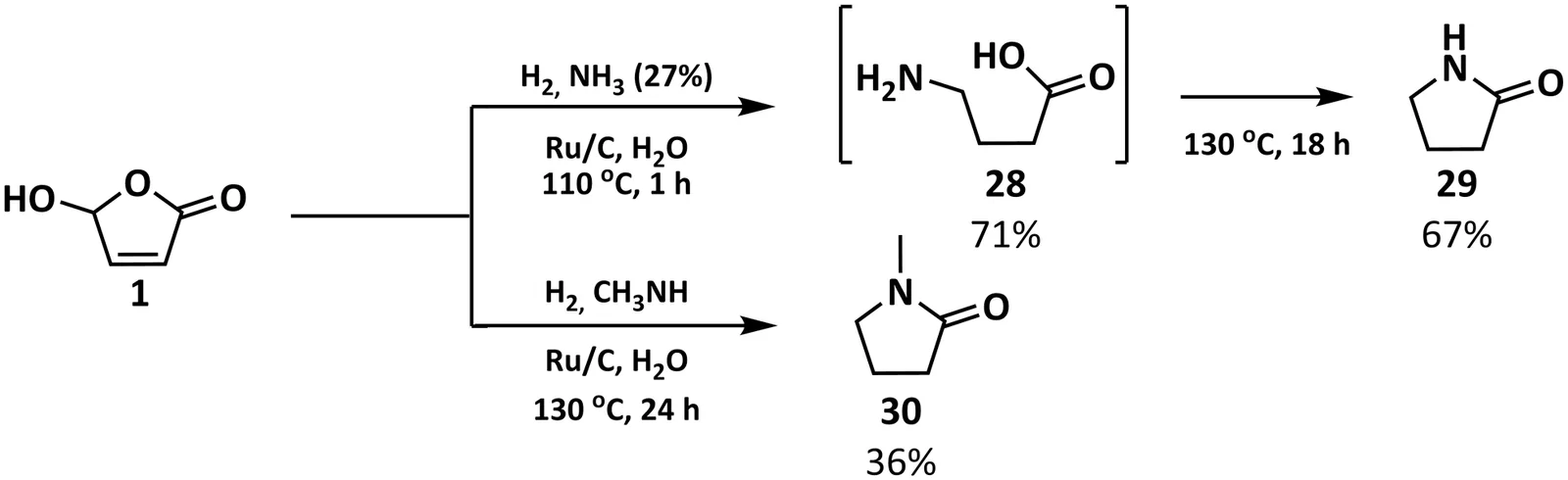
Reductive aminolysis of HFO to γ-aminobutanoic acid and 2-pyrrolidone over RU/C.^99^

Substitution of ammonia with methylamine redirects the reaction toward *N*-methylpyrrolidone (NMP, 30) as the main product. Although HFO is fully consumed within 30 min, NMP 30 formation proceeds more slowly, reaching 36% yield after 24 h (Scheme 20). The absence of detectable intermediates suggests the involvement of oligomeric imines species that are gradually converted into NMP 30.^99^

Industrially, 2-pyrrolidone 29 and NMP 30 are typically produced from petroleum-derived GBL 26 under harsh conditions (250–400 °C, 6–12 MPa). In contrast, the direct aminolysis of HFO proceeds under substantially milder conditions, highlighting its potential as a more sustainable and energy-efficient alternative to conventional petroleum-based routes to these products, which typically require harsh temperatures and pressures.^99^

#### 3.2.2. Hydrodeoxygenation

Selective hydrodeoxygenation of HFO 1 to GBL 26 provides a sustainable alternative to conventional petroleum-based production routes. In 2017, Chen *et al.*^156^ reported that HFO can be efficiently converted to GBL 26*via* hydrodeoxygenation over noble-metal nanoparticles supported on mesoporous Nb–Zr mixed oxides (Scheme 21).^156^

**Scheme 21:**
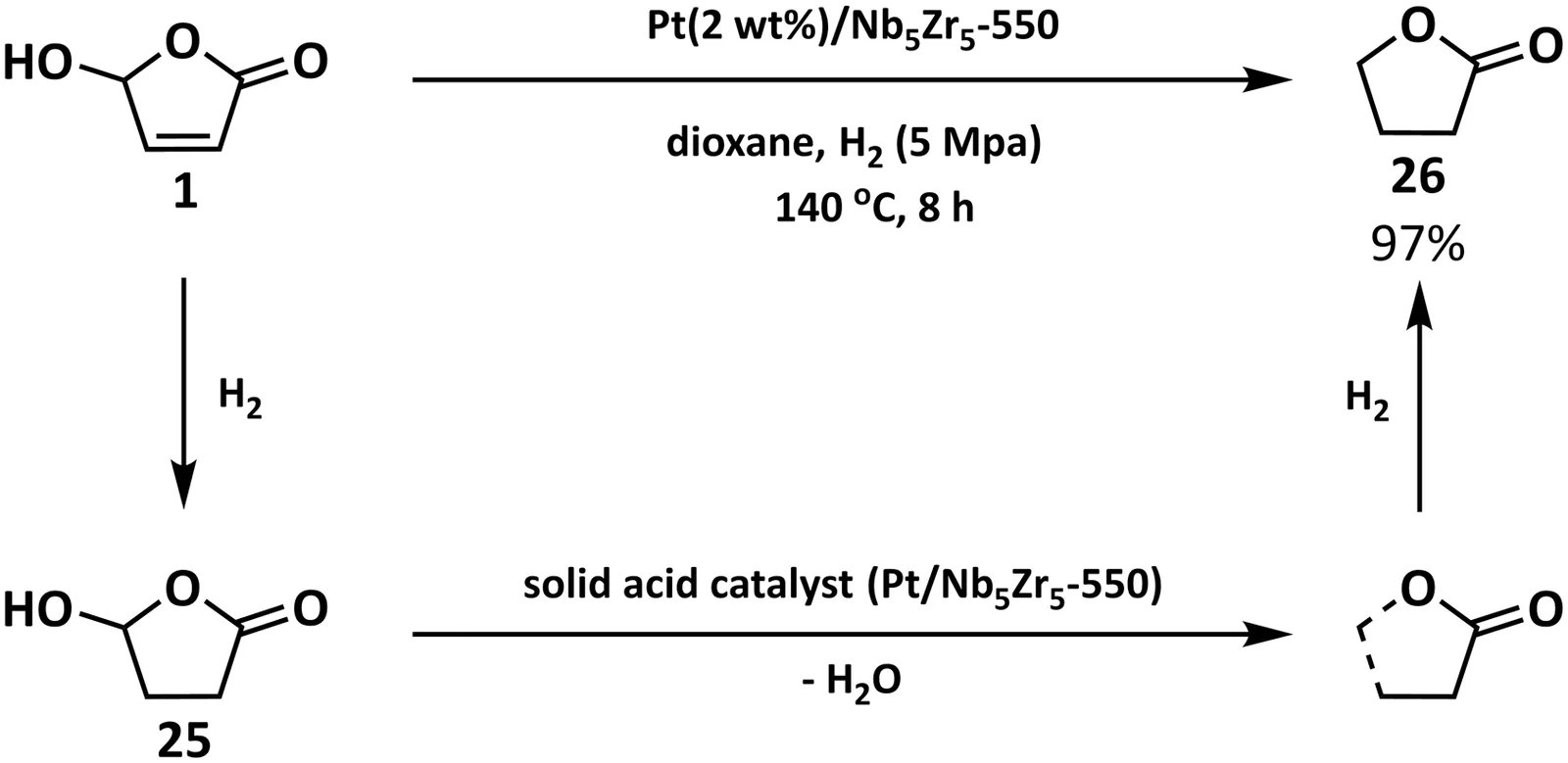
Catalytic conversion of HFO into γ-butyrolactone (GBL).^156^

This transformation proceeds through two key steps: hydrogenation catalysed by metal nanoparticles and dehydration facilitated by mesoporous acid sites. The catalytic performance depends strongly on both the composition of the support and the identity of the metal (M = Pt, Ir, Ru, Rh, Pd). Incorporation of Zr into the Nb-based matrix enhances thermal stability and increases surface acidity, thereby improving selectivity toward GBL.^156^

Among the catalysts evaluated, Pt/Nb_5_Zr_5_-550 exhibited the best performance, achieving complete conversion of HFO with 97% selectivity toward GBL 26. This superior activity is attributed to the synergistic interaction between metal and acid sites. Notably, Pt-based bifunctional catalysts outperformed conventional systems such as Pt/H-ZSM-5, Pt/C, Rh/C, and Pd/C.^156^

### 3.3. Addition to 5-hydroxybutenolide (HFO)

The presence of a conjugated acrylic-like system, combined with a hemiacetal-type hydroxyl functionality, renders HFO a highly reactive crystalline compound. This structural arrangement makes HFO particularly susceptible to nucleophilic attack by a wide range of reagents. In addition, the hydroxyl group exhibits the typical reactivity of (chiral) alcohols and can be readily derivatised to form ethers, esters, carbonates, and carbamates.

Among these transformations, etherification represents one of the most straightforward and efficient strategies for chemical modification. Typically, etherification is carried out in the corresponding alcohol solvent under acidic conditions, employing catalysts such as sulfuric acid or *p*-toluenesulfonic acid (PTSA), often by heating under reflux.^29,52,56^ This transformation improves both the stability and solubility of the resulting derivatives by reducing intermolecular hydrogen bonding and tuning molecular polarity, thereby enhancing compatibility with a broader range of organic solvents. In some cases, the resulting ether derivatives may also serve as functional solvents.

#### 3.3.1. Etherification (alkylation and acrylation)

Etherification of HFO has been widely investigated in our group using various alcohols under acidic conditions.^54,107^ In 2009 and 2013, Hoffmann and coworkers reported the reaction of HFO 1 with fatty alcohols in toluene under azeotropic distillation, affording the corresponding 5-alkoxybutenolides 7a–c in yields up to 83% (Scheme 22A).^157^ The authors observed that increasing the carbon chain length of the alcohol led to decreased yields, likely due to reduced solubility and/or diminished nucleophilicity. However, prolonged heating under these conditions (110 °C, 72 h) resulted in the formation of open-chain byproducts 31a–c bearing α,β-unsaturated aldehyde functionalities (up to 51% yield), highlighting the sensitivity of the system to the reaction conditions (Scheme 22B).^158^

**Scheme 22:**
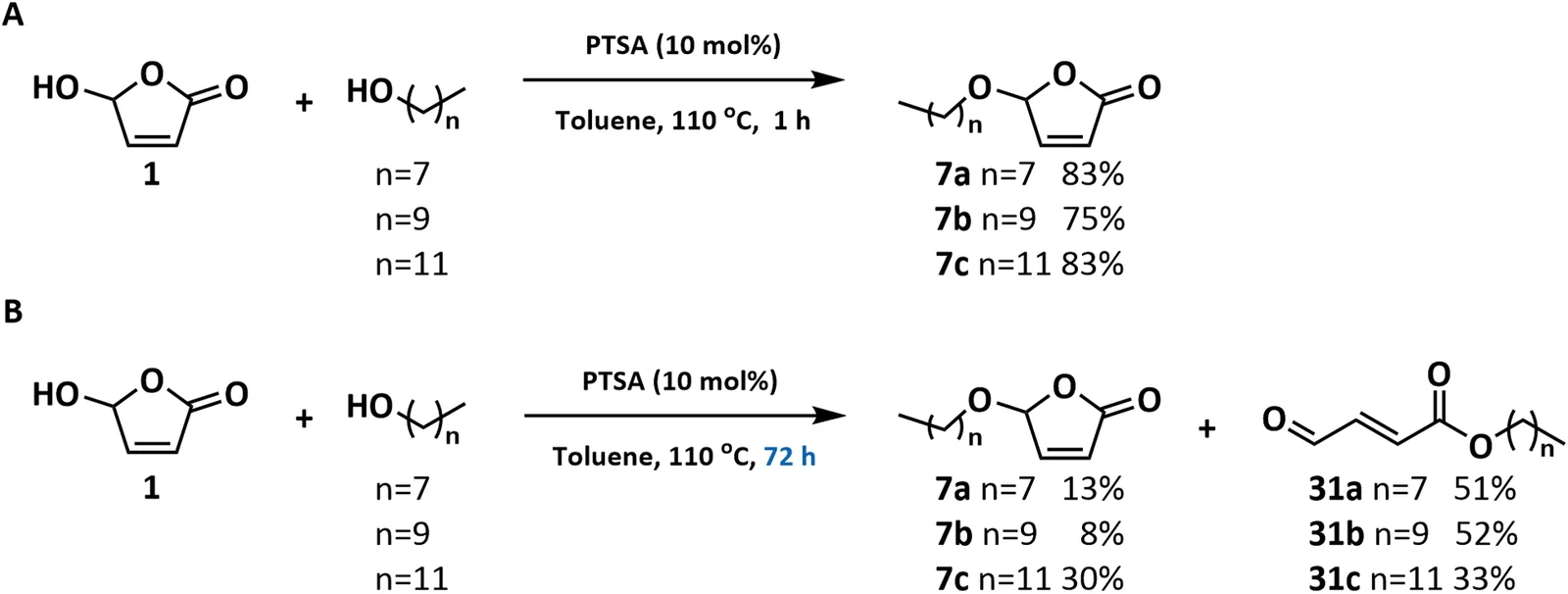
Etherification reactions of HFO with fatty alcohols, (A) for 1 h yielding ring closed butenolide products, (B) prolonged heating results in the formation of ring-opened byproducts.^157,158^

The Feringa group demonstrated that etherification can be efficiently performed on a 100 g scale using linear and branched alcohols, as well as the natural product (−)-menthol, in toluene with PTSA as a catalyst (Scheme 23).^49,54,107^ This work highlights both the scalability and versatility of the transformation, enabling access to a wide range of functionalized 5-alkoxybutenolide derivatives 7. Notably, the (5*R*)-menthyloxybutenolide 7f has emerged as a valuable chiral building block and has been widely employed as a substrate in asymmetric transformations (Section 3.10).^49–56^

**Scheme 23:**
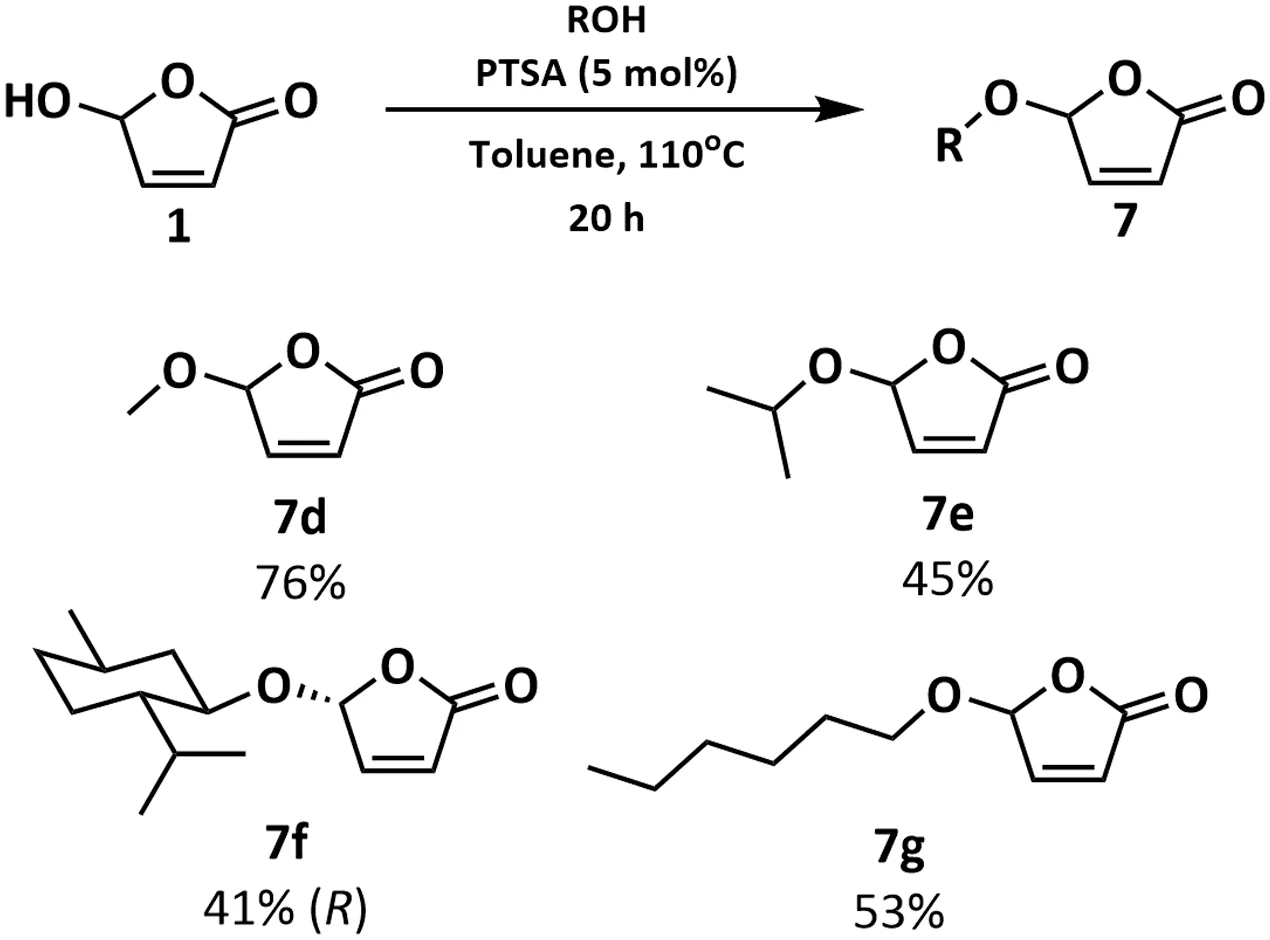
Acid-catalysed etherification of HFO.^49,54,107^

Advances in process intensification have further improved the efficiency of etherification reactions. In 2024, George and coworkers reported a catalyst-free continuous superheated flow process (tubular reactor) for the thermal condensation of unprocessed HFO (directly from photoreactor, containing methylene blue photosensitizer) at 200 °C (Scheme 24). This method enabled the rapid synthesis of 5-methoxybutenolide 7d with residence times of less than 2 min. Using a 3 mL plug-flow reactor, a projected productivity of 732 g day^−1^ was achieved, which could be further increased to 1.1 kg day^−1^ by simple scale-up through increasing the reactor volume.^120^ Notably, this telescoped HFO process required no intermediate purification, underscoring its potential for sustainable large-scale production of bio-based monomers.^120^

**Scheme 24:**
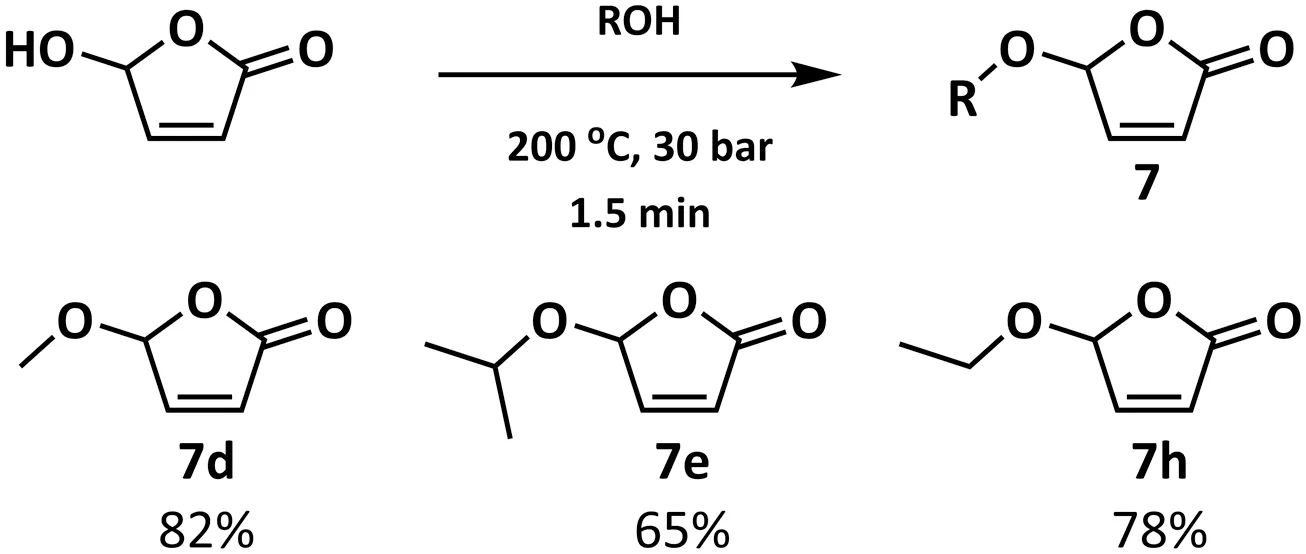
A catalyst-free continuous-flow etherification of HFO under superheated conditions.^120^

In addition to methanol, other alcohols such as isopropanol and ethanol were successfully employed under similar flow conditions (200 °C, 2 mL min^−1^), affording the corresponding 5-alkoxybutenolide derivatives 7e and 7h in moderate to good yields (65% and 78%, respectively).^120^

More recently, Le Dot *et al.* (2024)^159^ expanded the application scope of these 5-alkoxybutenolide derivatives by introducing electron-rich alkene-containing monomers 7i and 7j derived from HFO (Fig. 10). These compounds were shown to undergo homopolymerisation and act as effective comonomers in copolymer synthesis, highlighting their potential in advanced polymer and coating applications (see Section 5).^159^

**Fig. 10:**
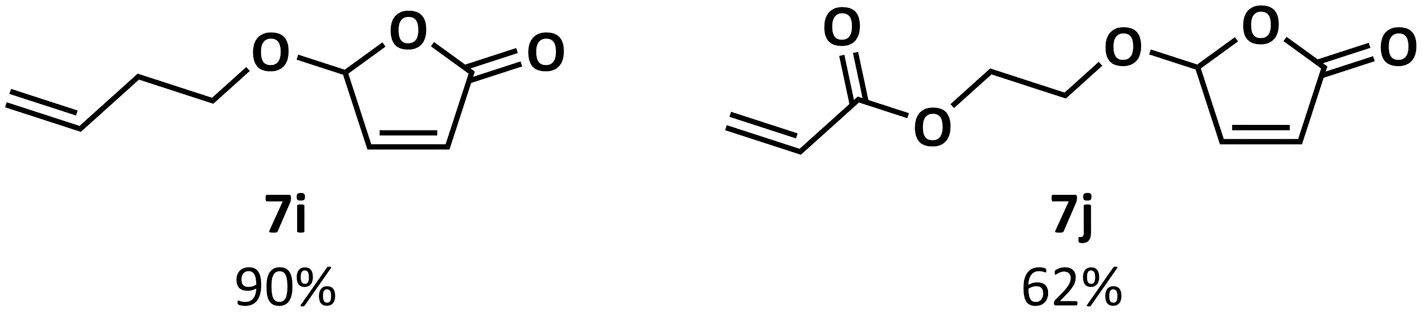
Electron-rich alkene-containing monomers derived from HFO.^159^

#### 3.3.2. Preparation of 5-thio butenolides and corresponding sulphones

The group of Martín reported the synthesis of 5-thiobutenolides from 5-methoxybutenolide (Scheme 25).^160,161^ The corresponding thioethers were prepared by reaction of thiols with 5-methoxybutenolide under reflux in dichloromethane (DCM) in the presence of BF_3_. 5-Ethylthiobutenolide 32 (70%) was obtained and subsequently oxidized to the corresponding sulfone 33 (80%) using 2 eq. of *m*-CPBA.^161^ These sulfone derivatives were further employed as dipolarophiles in 1,3-dipolar cycloadditions reactions, highlighting their utility as versatile synthetic intermediates.^161^

**Scheme 25:**
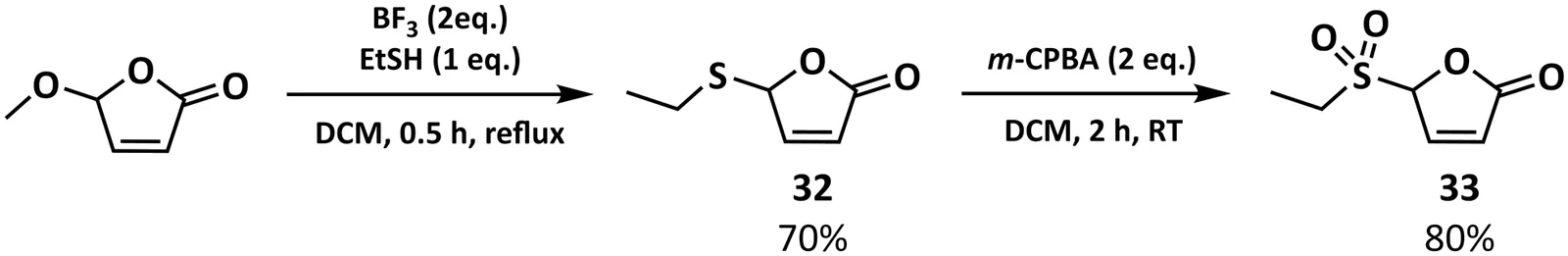
Preparation of 5-thiobutenolides and oxidation to sulphones.^161^

#### 3.3.3. Acylation and esterification

Classical acylation strategies of HFO have traditionally relied on the use of anhydrides, acyl chlorides, and carbodiimide-mediated esterification methods. In 2003, Trost *et al.*^162^ demonstrated that butenolide esters and carbonates can be efficiently synthesized from unpurified HFO 1 (crude product obtained *via* photooxidation of furfural).^162^ In this approach, acyl chlorides were used for esterification to 5-acyloxybutenolide 34a, while di-*tert*-butyl dicarbonate was employed for formation of butenolide carbonate 35a, affording yields of 69% and 61%, respectively (Scheme 26).

**Scheme 26:**
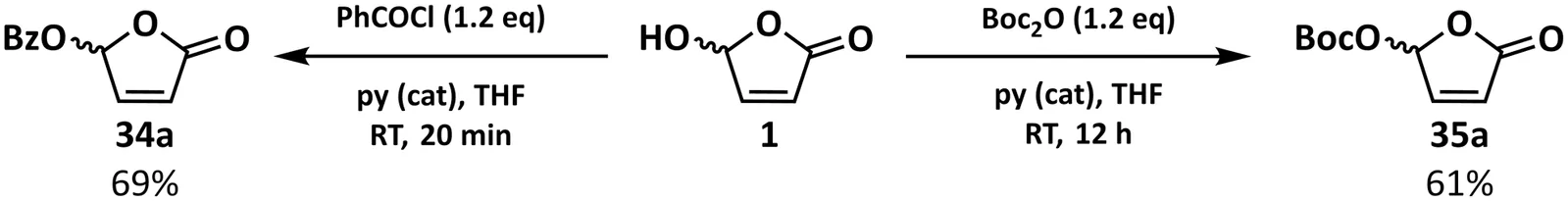
Acylation of the cyclic hemiacetal of HFO with anhydrides and acyl chlorides.^162^

Subsequently, in 2023, Feringa and coworkers expanded the derivatization scope by exploring the reactivity of the cyclic hemiacetal of HFO with anhydrides and carboxylic acids to access a broader range of esters and carbonates.^100^ For example, DMAP-catalyzed acylation (method A, Scheme 27) of HFO 1 with anhydrides afforded 5-acyloxybutenolides 34a–c in good yields (73–76%). In addition, ring-opening reactions with succinic anhydride enabled the synthesis of carboxylic acid-functionalized succinyloxy butenolide 34g in 56% yield. Butenolide 34g could be further converted into methyl ester 34h*via* addition of methanol (78% yield) or difunctional derivative 34i (potential polymer crosslinkers, *vide infra*) *via* subsequent addition of HFO (68%).^100^ Esterification under Steglich conditions (method B, Scheme 27) using bio-based carboxylic acids such as isobutyric, lauric, and oleic acid afforded 5-acyloxybutenolides 34d–f in yields of 71%, 66%, and 26%, respectively, with nearly quantitative bio-based carbon content. Butenolide carbonates 35a and 35b could be accessed through reaction with respectively dimethyl dicarbonate and Boc-anhydride.^100^

**Scheme 27:**
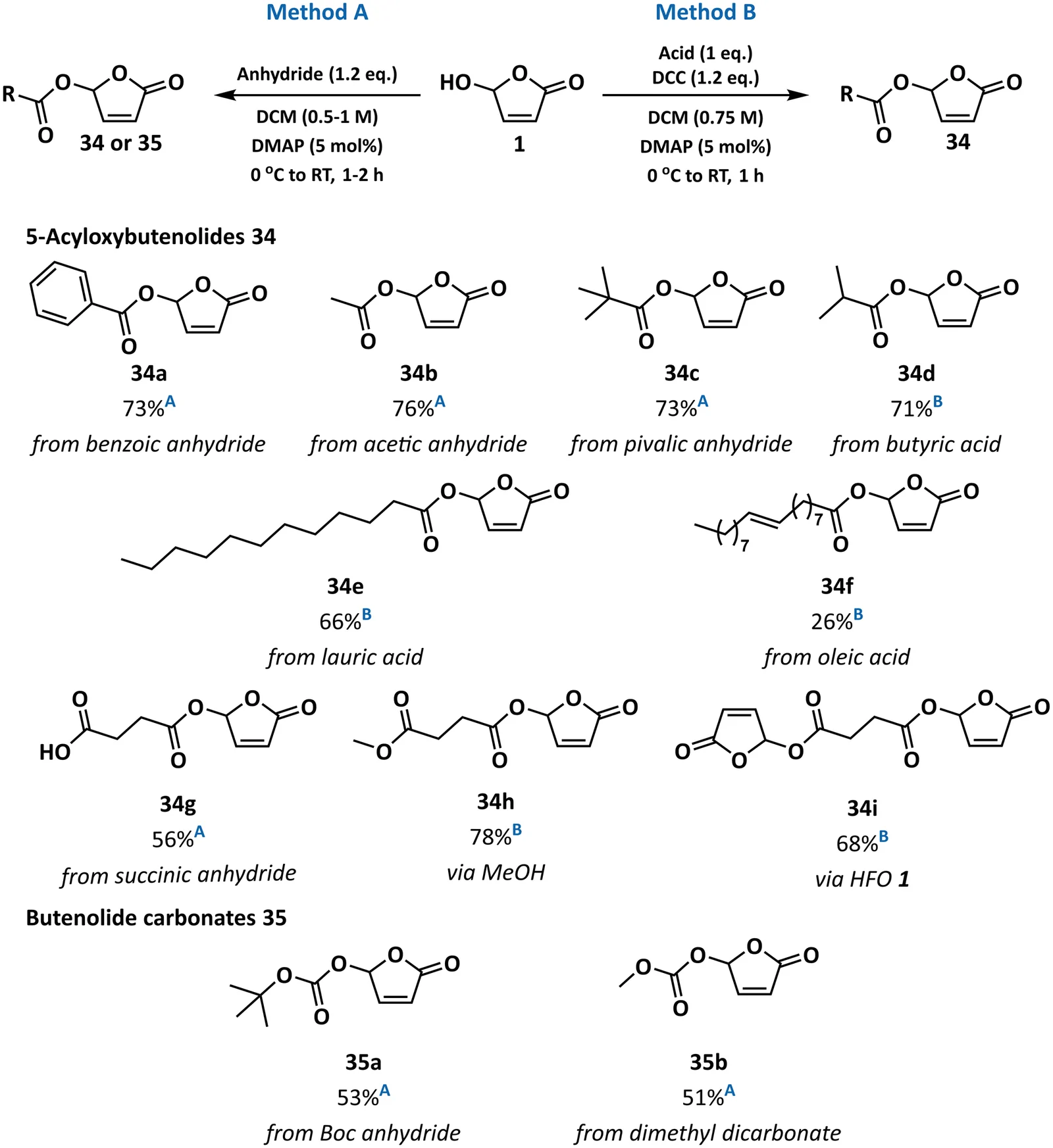
Synthesis of 5-acyloxybutenolides 34 and butenolide carbonates 35*via* DMAP-catalyzed acylation (method A) or Steglich esterification (method B).^100^

Moreover, the Feringa group demonstrated that butenolide carbamates 36 can be synthesized *via* tin-catalysed reactions (1 mol%) of HFO 1 with isocyanates (1.1 eq.), affording products in high yields ranging from 71% to 85% (Scheme 28).^100^

**Scheme 28:**
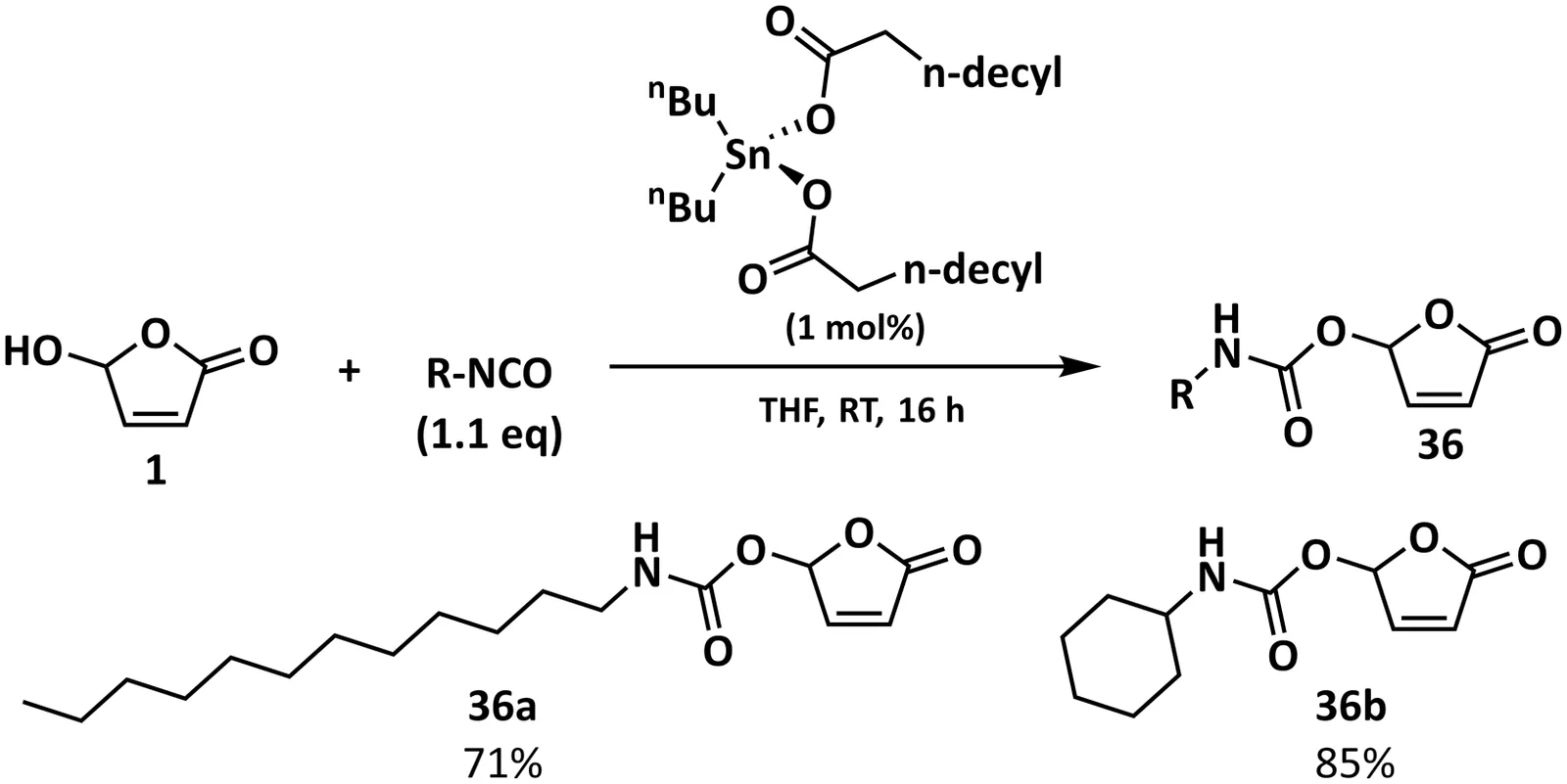
Carbamate derivatives from HFO *via* a tin-catalysed reaction with isocyanates.^100^

#### 3.3.4. Enzymatic acylation and dynamic kinetic resolution of 5-hydroxyfuran-2(5*H*)-one

The enantioselective acylation of HFO 1 has emerged as a key methodology for the preparation of enantiomerically enriched lactones, which serve as valuable intermediates in the synthesis of bioactive molecules. Vinyl acetate is commonly employed as the acyl donor due to its high reactivity under mild enzymatic conditions. The presence of a hemiacetal moiety in HFO enables spontaneous racemization, likely proceeding *via* a transient aldehyde intermediate, thereby allowing complete conversion to enantiomerically pure (*R*)-5-acetoxyfuran-2(5*H*)-one through dynamic kinetic resolution (DKR).

Early studies by Feringa and coworkers (1996–1998) demonstrated that lipase R (Amano, Penicillium roqueforti), immobilized on Hyflo Super Cel, catalyses the acylation of HFO 1 with vinyl acetate in diethyl ether (Et_2_O) at room temperature over 10 d (Scheme 29A), affording 90% conversion to (5*R*)-34b with excellent enantiomeric excess (ee = 99%).^94,97^ This transformation represents a classic example of enzyme-catalysed dynamic kinetic resolution, in which the less reactive enantiomer undergoes continuous racemization, enabling full conversion and high enantiomeric purity. Subsequent work by Feringa and coworkers, using lipase PS (Amano, *Pseudomonas fluorescens*), also immobilized on Hyflo Super Cel, significantly accelerated the reaction (Scheme 29B).^94,97^ Under ambient conditions in a 1 : 1 cyclohexane-butyl acetate mixture, complete conversion to (5*R*)-34b (99%) was achieved within 21 h, albeit with slightly reduced enantioselectivity (ee = 89%). Other acyl donors, such as vinyl propionate, were also successfully employed, providing (5*R*)-34j in high conversion (99%) with moderate enantioselectivity (ee = 83%).

**Scheme 29:**
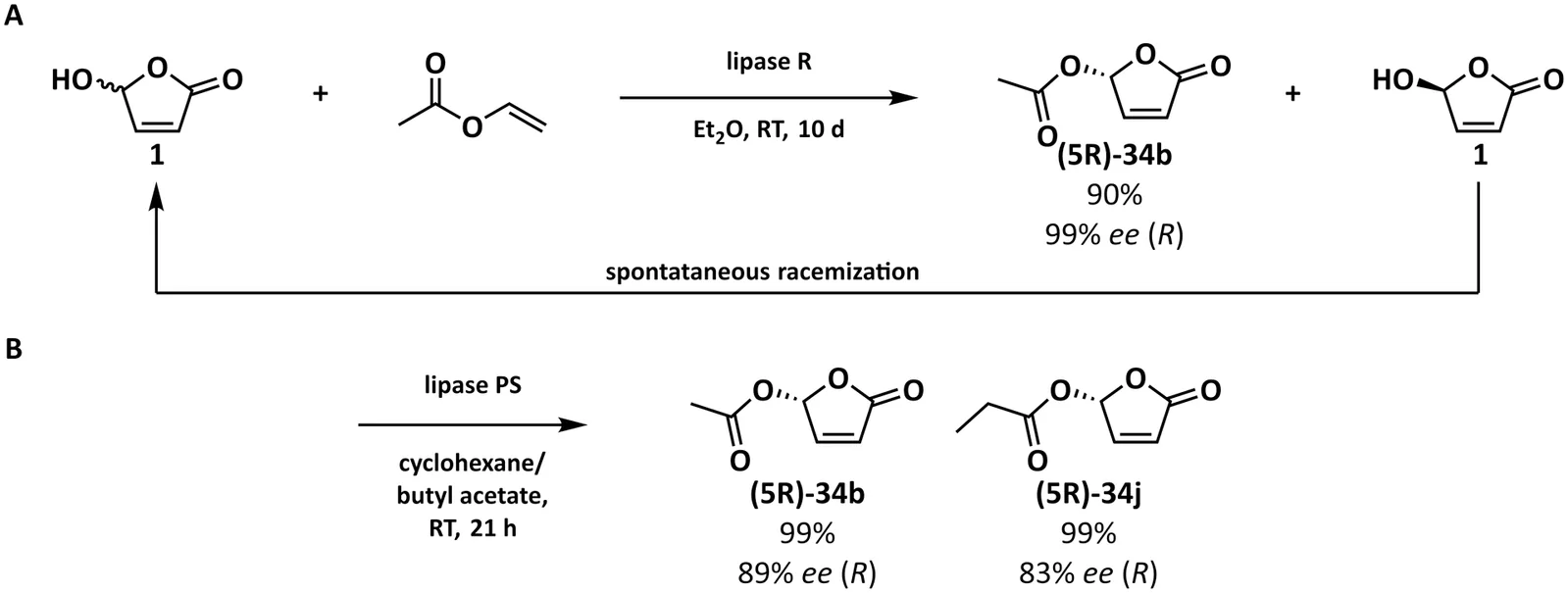
Dynamic kinetic resolution (DKR) of HFO with lipase R and lipase PS.^94,97^

Given the inherent enantioselectivity of enzymes and the suitability of the resulting products for downstream transformations, further optimization was pursued. In 2005, Morita *et al.*^163^ improved this methodology by employing lipase AK in vinyl acetate as both solvent and acyl donor (Scheme 30).^163^ This system was then successfully applied in the synthesis of (−)-kainic acid from HFO 1. At higher substrate loading (12 g) and using lipase AK (30% on Celite, 24 g), the reaction time was reduced to 21 h, affording 5-acetoxybutenolide (5*R*)-34b in quantitative yield with 93% ee.

**Scheme 30:**
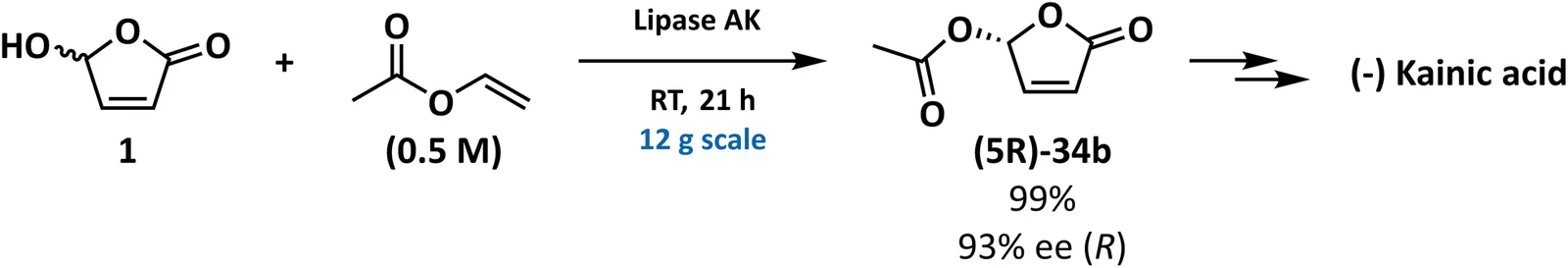
Dynamic kinetic resolution (DKR) of HFO with lipase AK.^163^

More recently, He and Li (2026) systematically investigated the enzymatic acylation of HFO using various vinyl esters as acyl donors.^93^ Six commercially available lipases, Lipozyme TL IM, Novozym 435, Lipozyme RM IM, Lipura Flex, Lipura Select, and lipase AK, were evaluated under identical conditions (50 mM HFO, 50 g L^−1^ enzyme, 2 mL 2-MeTHF containing 10% (v/v) vinyl acetate, 220 rpm, 50 °C, 2 h). Among these, Lipozyme TL IM exhibited the highest catalytic activity, affording the acylated product (5*R*)-34b in 94% yield and moderate ee (73%) within 2 h. In contrast, the other lipases provided yields below 65% under the same conditions, although yields above 90% were achieved upon extending the reaction time; notably, lipase AK afforded (5*R*)-34b in 95% yield and 89% ee after 18 h (Scheme 31).^93^

**Scheme 31:**
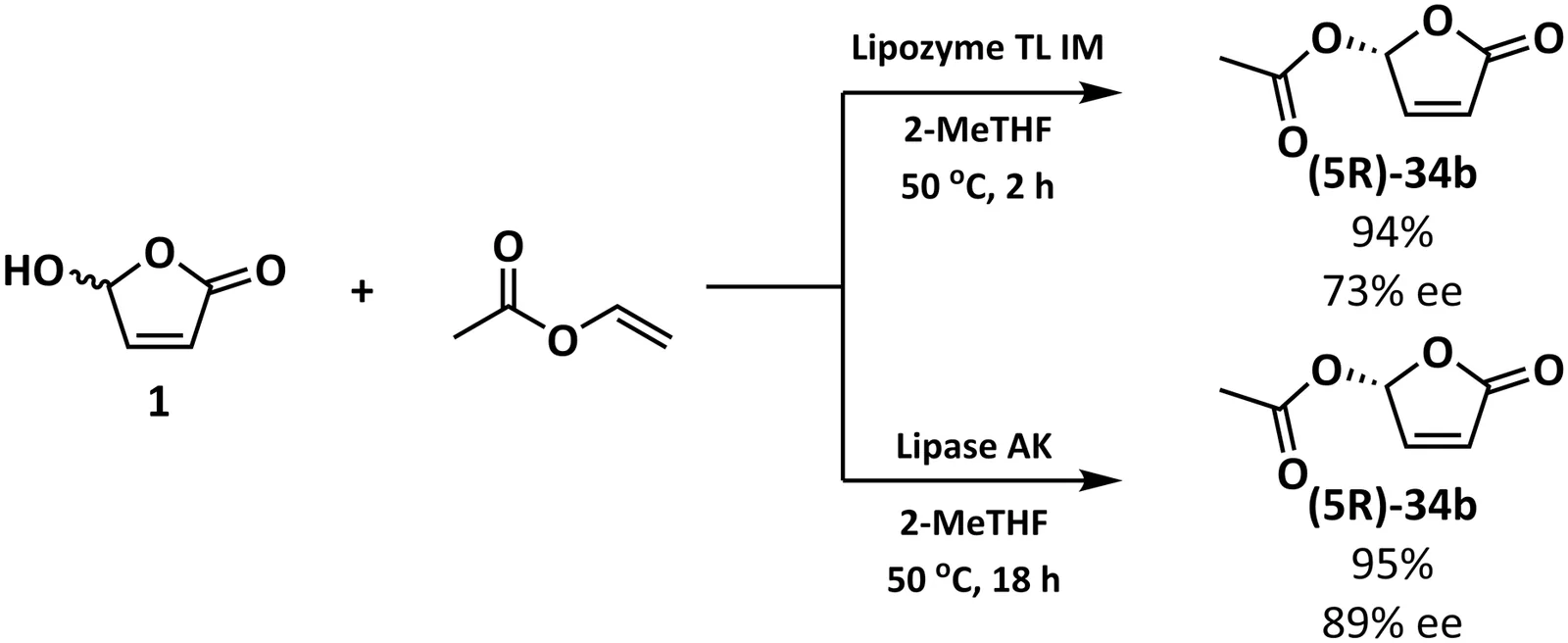
DKR of HFO with lipozyme TL IM and lipase AK.^93^

The authors extended the scope of the acylation by employing a range of linear fatty acid vinyl esters as acyl donors in combination with lipase AK in a continuous-flow system.^93^ Notably, crude HFO 1 was directly utilised after photooxidation in 2-MeTHF as the reaction medium. Under these conditions, moderate to good yields were achieved, ranging from 54% to 96% (Fig. 11). Importantly, the enantiomeric excess (ee) of the products was not affected by increasing the chain length of the acyl donors ((5*R*)-34e, (5*R*)-34k–n), achieving up to 89% ee. However, branched-chain fatty acid acyl donors resulted in (5*S*)-34c and (5*S*)-34o with lower yields (8% and 27% respectively) and slightly lower enantiomeric excess values (90% ee and 51% ee). This behaviour is likely attributed to increased steric hindrance of the acyl donors, which reduces reaction rates and can negatively impact selectivity (Fig. 11).^93^

**Fig. 11:**
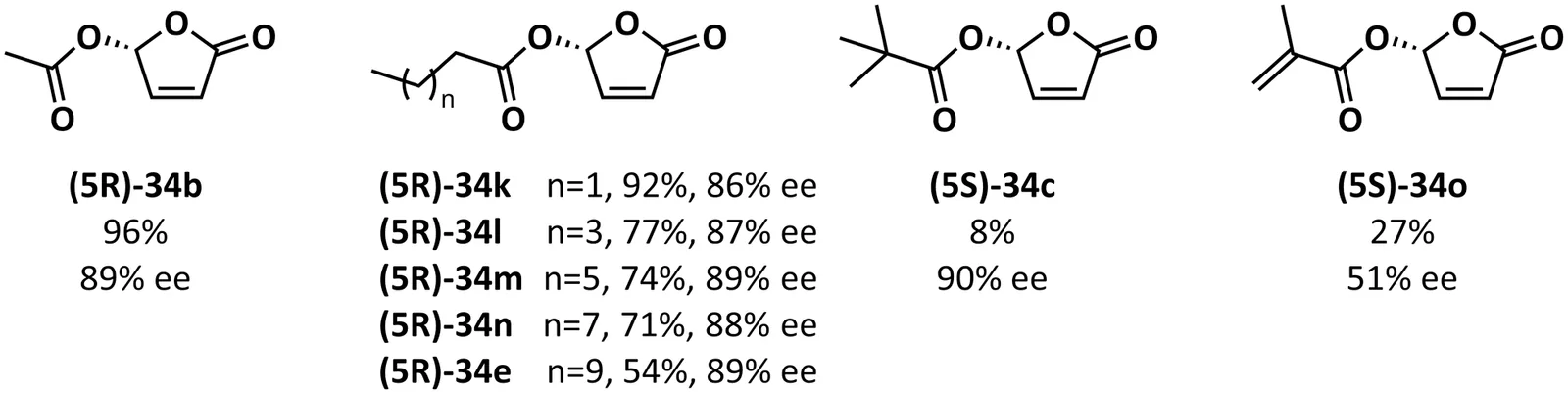
DKR products of HFO with different vinyl esters, identification of (*S*)-configuration of 34c and 34o was based on priority of the acyl group compared to butenolide motif.^93^

Notably, the yield obtained with lipase AK (96%, (5*R*)-34b) significantly exceeds the theoretical maximum of 50% expected for classical kinetic resolution of racemic substrates. This observation strongly supports the earlier discoveries^94,97^ on the occurrence of dynamic kinetic resolution, likely enabled by continuous *in situ* racemization of the chiral substrate under the reaction conditions, allowing conversion of not only (*R*)- but also (*S*)-enantiomers into the desired product.^93^

Collectively, these studies highlight the powerful synergy between enzymatic catalysis and substrate racemization, enabling efficient access to enantiomerically enriched (5*R*)-acyloxybutenolides. The choice of lipase, solvent, and reaction conditions plays a critical role in balancing reaction rate and stereoselectivity, enabling fine-tuning toward high-yield and high-enantioselectivity transformations. These methodologies have facilitated the scalable synthesis of chiral intermediates for natural product synthesis, including (−)-kainic acid, and remain a cornerstone in the biocatalytic functionalisation of HFO 1.

### 3.4. Organocatalytic and multicomponent reactions of 5-hydroxybutenolide (HFO)

The unique combination of a reactive α,β-unsaturated carbonyl system and a hemiacetal hydroxyl group in HFO enables its application in enantioselective organocatalysis and multicomponent reactions. These transformations provide efficient access to chiral lactones and structurally diverse heterocycles.^93^

#### 3.4.1. Organocatalytic enantioselective Friedel–Crafts reaction

In 2011, Riguet demonstrated that HFO can serve as an efficient electrophile in organocatalyzed enantioselective Friedel–Crafts alkylation reactions (Scheme 32).^164^ This methodology provided access to chiral lactones 37a-g in high yields (80–93%) and excellent enantioselectivities (er = 91 : 9 to 96 : 4). Among the catalysts evaluated, diphenylprolinol silyl ethers, C1 outperformed simple diphenylprolinol, delivering improved reaction rates and higher enantiocontrol (up to er = 96 : 4).^164^

**Scheme 32:**
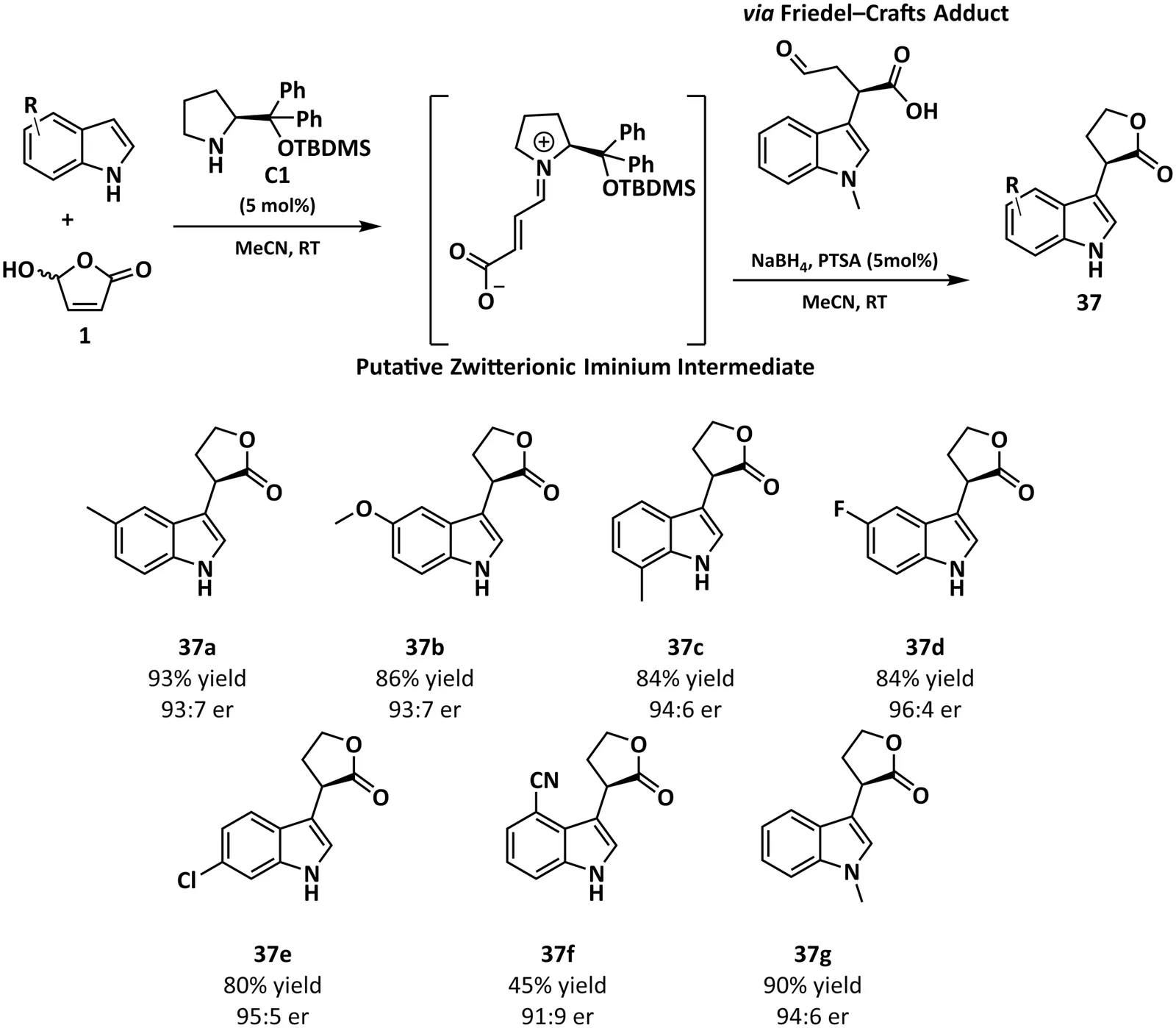
(A) An organocatalyzed enantioselective Friedel–Crafts alkylation reaction with HFO, (B): representative chiral lactone derivatives obtained *via* Friedel–Crafts alkylation reaction with HFO.^164^

The authors reported that solvent effects play a crucial role in both reactivity and selectivity. For instance, in 2-MeTHF, the reaction proceeded more slowly (12 h) but afforded high enantioselectivity (er = 96 : 4). In contrast, polar protic solvents such as ethanol or water (H_2_O) (with a small amount of THF for solubility) significantly accelerated the reaction, reaching full conversion within 2 h while maintaining high enantiomeric ratios (er ≈ 95 : 5). DCM enabled very rapid conversion (1 h), albeit with slightly diminished enantioselectivity (er 89 : 11). A variety of indole derivatives, including *N*-methyl and unsubstituted indoles, were successfully employed, consistently affording high yields and selectivities (Scheme 32).^164^

#### 3.4.2. Tandem Friedel–Crafts–Ugi multicomponent reaction

Building on this reactivity, Riguet developed a tandem sequence combining an enantioselective Friedel–Crafts reaction with a Ugi four-component reaction.^164^ This strategy enables rapid diversification of the indole scaffold. The author developed a one-pot variant of this process using ethanol as solvent which is suitable for both reactions (Scheme 33). While this approach simplified the procedure, it required higher catalyst loadings (10 mol%) and resulted in slightly reduced enantiomeric excess (78–88% ee). The intramolecular Ugi step remained poorly diastereoselective, yielding the two diastereomers in similar amounts. Although the Ugi step exhibited low diastereoselectivity, the resulting diastereomers 38 were readily separated by column chromatography (Scheme 33).^164^ Importantly, the enantiomeric ratio (er ≈ 94 : 6) established during the Friedel–Crafts step was preserved throughout the sequence. The author further demonstrated that this tandem methodology is also applicable to NH-indole derivatives. For example, reactions involving 5-fluoro-1*H*-indole afforded diastereomers 38g and 38h in moderate yields (∼30%) while maintaining good enantioselectivity (er 91 : 9).^164^

**Scheme 33:**
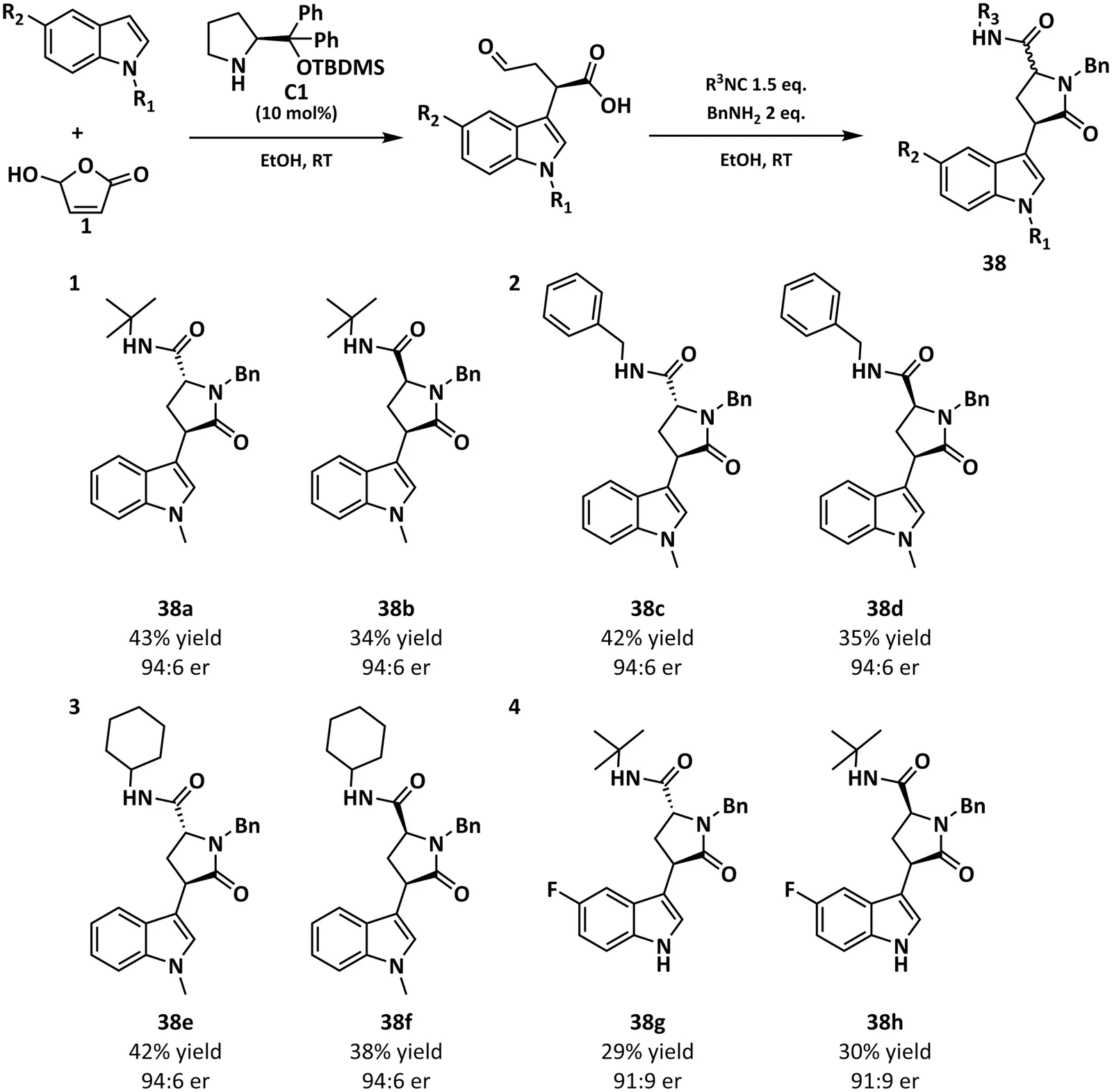
(A): One-pot Friedel–Crafts/Ugi reaction with HFO. (B) Scope of chiral Friedel–Crafts/Ugi products, yields of diastereomers after separation given.^164^

#### 3.4.3. Intramolecular Passerini-type reaction

In 2014, Riguet and coworkers further expanded the synthetic utility of HFO 1 through a one-pot sequence combining enantioselective organocatalytic boronic acid transfer with an intramolecular Passerini reaction.^165^ The study began with an organocatalytic Michael-type addition of boronic acids. The enantioselective transfer of the boronic acid substituent to HFO 1 was evaluated *via* conversion of the addition product to monosubstituted lactone 38. Six catalysts derived from diphenyl prolinol were tested of which the one containing the OTBDMS (C1) emerged as the most promising (Scheme 34 in blue). Investigation revealed that H_2_O content critically influenced the transformation; anhydrous conditions gave low conversion, while excess H_2_O slowed the transfer. Optimal results were achieved with 5 equiv. of H_2_O leading to complete consumption of the substrate in 2 h. Subsequent NaBH_4_ reduction and lactonisation (using PTSA) afforded lactone 38 in 98% yield with an enantiomeric ratio of 92 : 8 (Scheme 34 in blue).^165^

**Scheme 34:**
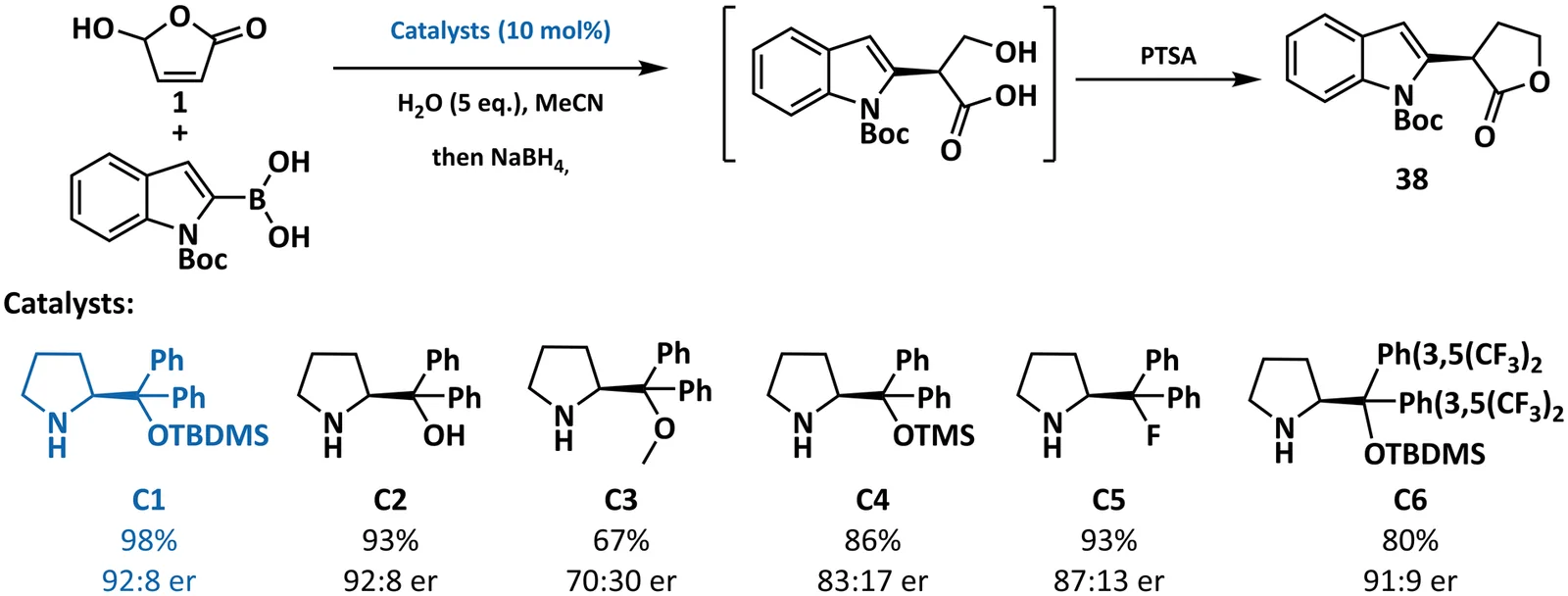
Organocatalytic transfer of boronic acids to 5-hydroxyfuranone-2(5*H*)-one, scope of catalysts tested with optimal catalyst highlighted in blue.^165^

Using *N*-Boc-indole-2-boronic acid, the initial transfer step proceeded with good enantioselectivity. Subsequent addition of an isocyanide triggered an intramolecular Passerini reaction, affording α,γ-substituted chiral γ-lactones 39 in high yields (Scheme 35A).^165^ The products were obtained as separable diastereomeric mixtures (*e.g.*, dr = 72 : 28 for compound 39a), with both isomers exhibiting similar enantiomeric ratios (er ≈ 90 : 10) (Scheme 35B). X-ray crystallographic analysis confirmed the relative stereochemistry of the minor diastereomer.^165^

**Scheme 35:**
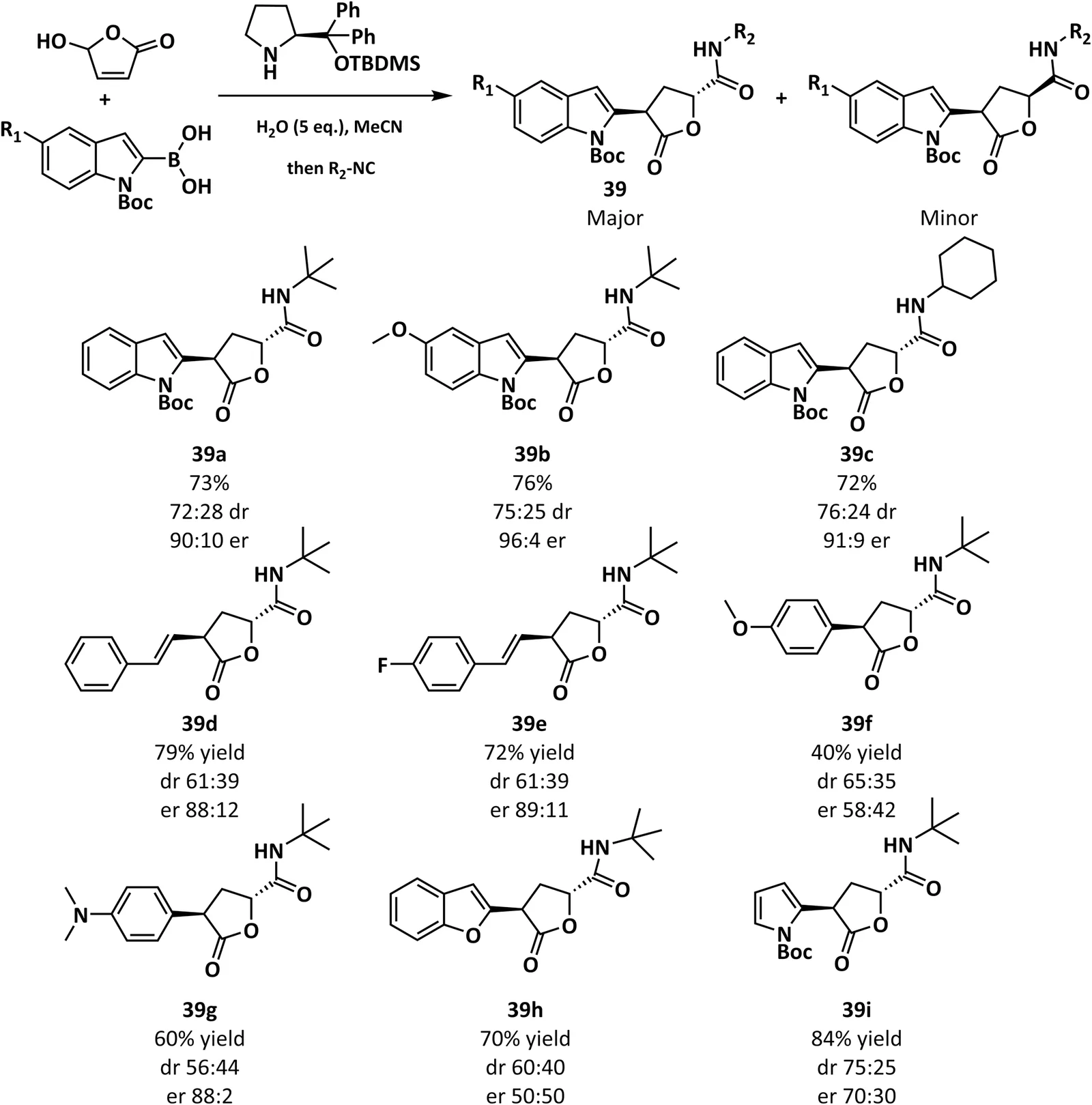
Access to chiral γ-lactones by one pot sequential organocatalytic Michael addition of boronic acid and diastereoselective intramolecular Passerini reaction with HFO.^165^

This methodology was successfully extended to a range of boronic acids, including styrenyl (39d–e), electron-rich aromatic (39f–g), and heteroaromatic derivatives (39h–i). While good yields were generally obtained (63–79%), diastereoselectivity was somewhat reduced for non-indole substrates (dr ≈ 61 : 39). Notably, this work represents one of the first examples enabling direct access to chiral lactones *via* an intramolecular Passerini reaction.^165^

#### 3.4.4. Base-catalysed and condensation reactions

Earlier studies have also demonstrated the versatility of HFO in base-catalysed condensation reactions. In 1982, Yuste *et al.*^166^ reported that HFO 1 reacts with both cyclic and acyclic vinylogous amides, as well as 1,3-dicarbonyl compounds to afford a range of heterocycles, including pyrroles, furans, tetrahydroindoles, and tetrahydrobenzofurans in moderate to good yields (37–83%, Scheme 36).^166^ The base catalysed reaction of enaminone 40 with HFO 1 afforded 41 in 83% yield as an insoluble crystalline solid. Subsequent treatment with base (5% Na_2_CO_3_) resulted in formation of tetrahydroindole 42 (54%). Base-catalyse reaction of vinylogous secondary amide 43 with HFO 1 directly yielded tetrahydroindole 44. Similarly, acyclic vinylogous primary amide 45 with HFO 1 under base catalysed conditions provided pyrrole 46 (60%). Furans 47 (66%) and 48 (77%) are accessible from HFO 1 and acyclic 1,3-dicarbonyl compounds *via* heating at reflux in ethanol in the presence of 2% triethylamine. Finally, tetrahydrobenzofurans 49 (40%) and 50 (37%) were obtained from HFO 1 and cyclic β-diketones by first heating at reflux in the presence of base and subsequent heating with dilute acid.^166^

**Scheme 36:**
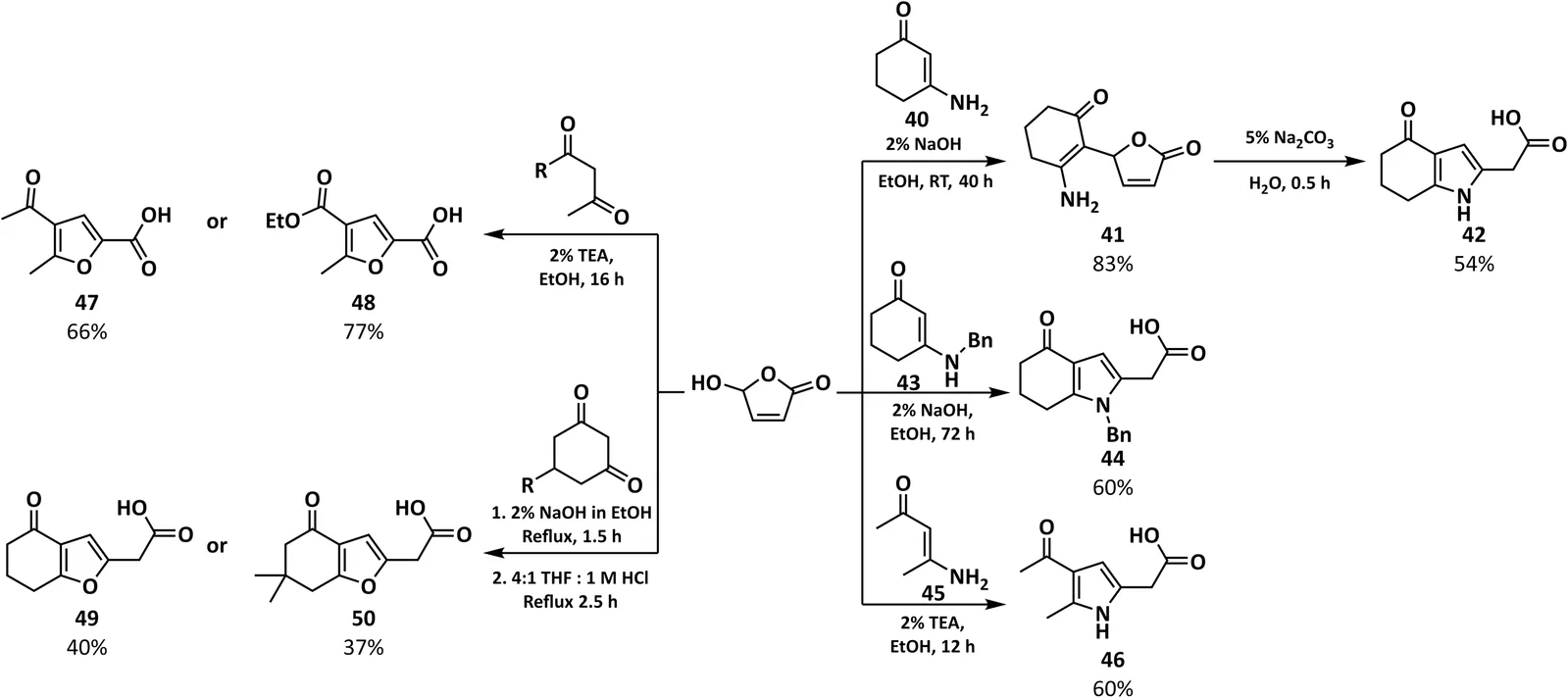
Base-catalyzed reactions of HFO with several cyclic and acyclic 1,3-dicarbonyl compounds and vinylogues amides for the preparation of pyrrole, furan, tetrahydroindole and tetrahydrobenzofuran ring systems.^166^

Similarly, Yuste *et al.*^167^ (1988) described the condensation of HFO 1 with activated methylene compounds such as ethyl acetoacetate and β-keto esters. These reactions proceed *via* nucleophilic attack at the lactone carbon, followed by intramolecular cyclization (Michael-type addition) and aromatization to yield substituted furan derivatives 50a-e in good yields (56–90%, Scheme 37).^167^

**Scheme 37:**
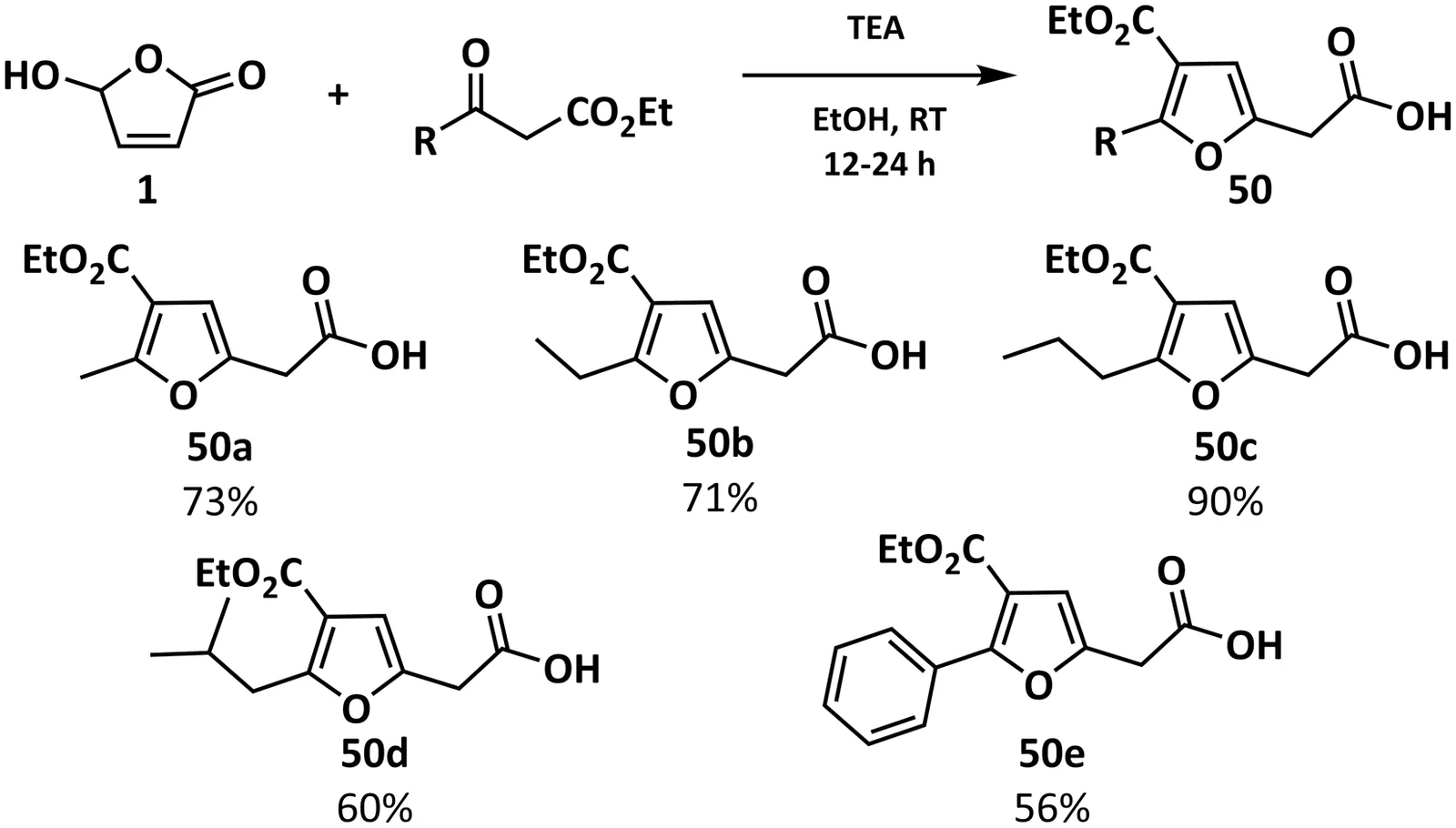
Condensation of HFO with activated methylene groups.^167^

### 3.5. Ring-opening alcoholysis and synthetic applications

Under acidic conditions, HFO 1 readily undergoes ring-opening alcoholysis to generate α,β-unsaturated acetal intermediates. Early work in 1973 by Meyers *et al.*^168^ and later by the group of González demonstrated that treatment with BF_3_·Et_2_O in ethanol affords the ring-opened product, ethyl 4,4-diethoxycrotonate 51 in moderate to excellent yields (up to 96%, Scheme 38).^168,169^ It was important to distill the crude over potassium bisulfate to convert the undesired 52 into the desired alkene 51. The authors further derivatized 51 by hydrolysis to afford aldehyde 53, which upon treatment with methyl magnesium chloride gave hydroxyester 54.^169^

**Scheme 38:**
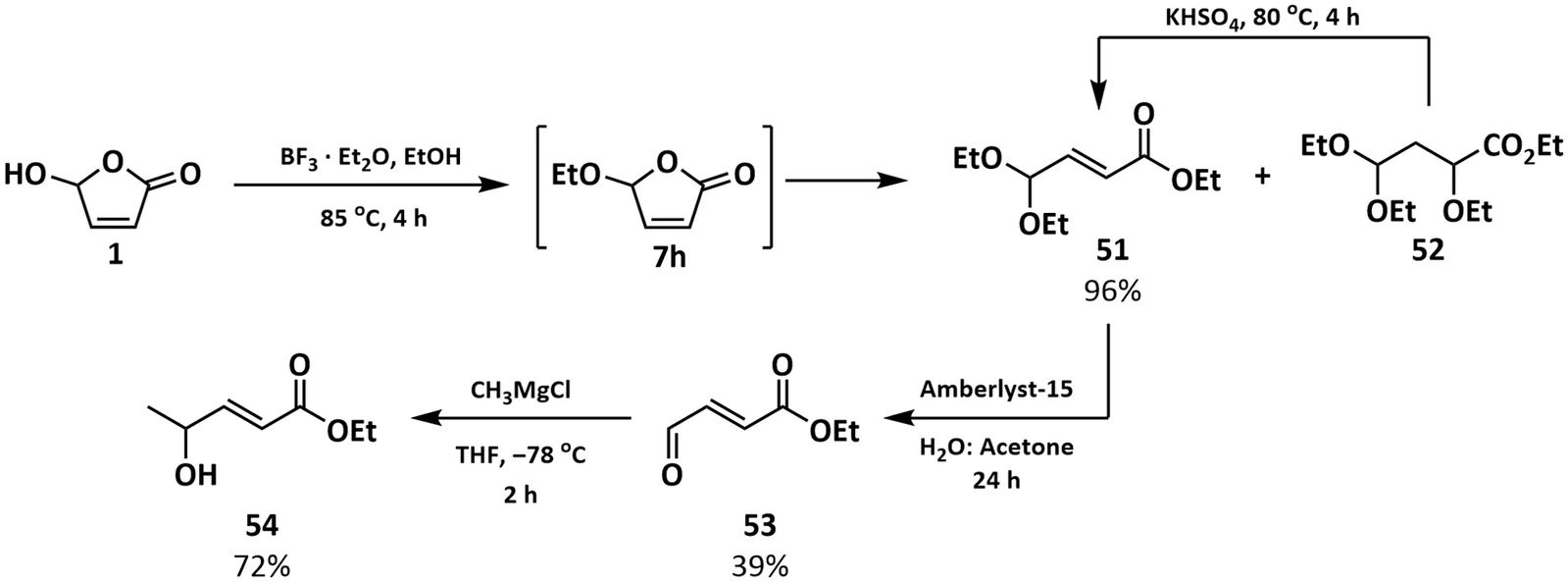
Ring-opening alcoholysis HFO and their subsequent derivatization reactions.^169^

More recently in 2019, Li *et al.*^170^ reported that *p*-toluenesulfonic acid (PTSA)-catalysed alcoholysis of HFO 1 in dry ethanol produces ethyl 4,4-diethoxycrotonate 51 in 72% yield (Scheme 39). The mixture was stirred until full completion, and the product was purified *via* vacuum distillation. This intermediate serves as a versatile building block for heterocycle synthesis. For example, it can undergo a Doebner-Miller reaction with aniline to produce quinoline derivatives in yields up to 80%, further derivatizable towards 2-quinaldic acid 55. This approach avoids the use of toxic reagents such as cyanides and is consistent with the Green Chemistry principles.^2,4,170^

**Scheme 39:**
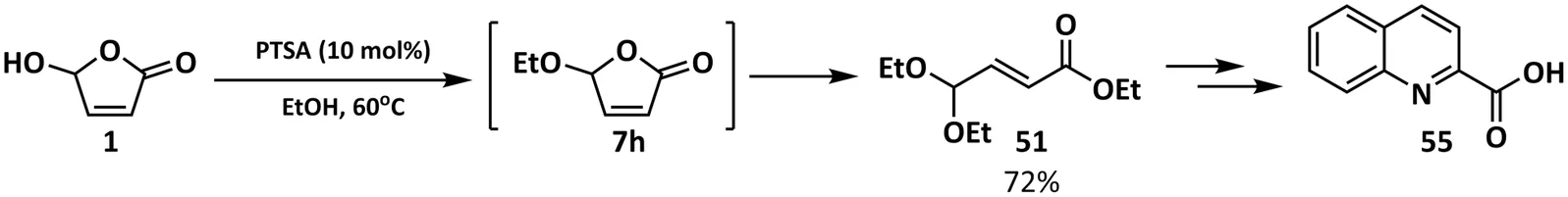
Ring-opening alcoholysis HFO leading to quinoline derivatives.^170^

### 3.6. Tautomerization and aqueous transformations

Hydroxyfuranic compounds, particularly 5-hydroxy-2(5*H*)-furanone (HFO), have emerged as versatile precursors for the sustainable production of succinic acid (SA, 59) and related platform chemicals. Studies by Poskonin *et al.*^96^ demonstrated that slow hydrolysis of HFO 1 in aqueous solution yields SA 59 in modest amounts when heated at 60 °C for 24 h (∼13%, Scheme 40A).^96^ The reaction was accelerated under alkaline conditions (pH 9–10, NaOH), resulting in practically complete conversion in minutes when heated at 70–80 °C (Scheme 40B), highlighting the strong influence of pH on reaction efficiency. The authors propose a mechanism linked to migration of the proton at position 5 of HFO without ring-opening of HFO, where HFO is in equilibrium with unstable tautomeric forms (56 and 57), and hydrolysis of succinic anhydride **58** results in formation of SA **59** (Scheme 40A). This is in contradiction with the mobile equilibrium described between HFO 1 and *cis*-β-formylacrylic acid 2*via* ring-chain tautomerization. These findings underscore the critical role of reaction conditions in governing both yield and selectivity in the conversion of HFO to C4 dicarboxylic acids.^96^

**Scheme 40:**
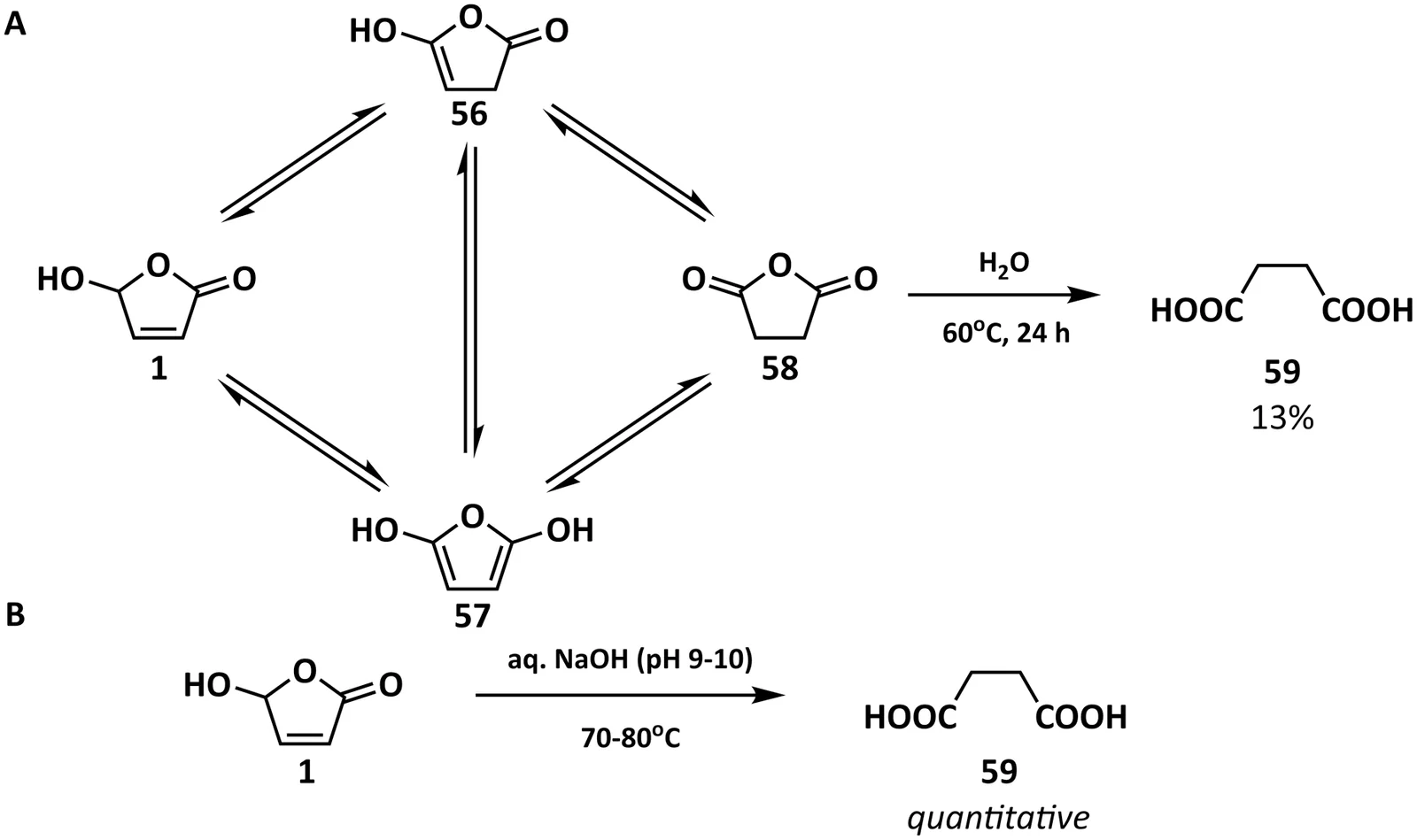
Sustainable production of succinic acid (SA) from HFO, (A) HFO and its equilibrium with tautomeric forms in water, (B) reaction of HFO to SA under alkaline conditions.^96^

More recently, Huang *et al.*^171^ demonstrated that the ring-opening and concurrent isomerization of HFO 1 to the intermediate fumaric semi-aldehyde (FSA, 6) can be achieved under mild aqueous conditions using a weakly basic resin, such as divinylbenzene (DVB)-based resin (Scheme 41).^171^ The resulting FSA 6 can be efficiently oxidized to fumaric acid (FA, 24) using an enzymatic laccase-TEMPO system. FA 24 can subsequently be converted into amino acids, including l-aspartic acid (l-Asp, 61) and β-alanine (β-Ala, 62), while chemical reductions employing NaBH_4_ or NaH_4_/Al/CuCl systems enable access to other functionalized derivatives such as ((2*E*)-4-hydroxy-2-butenoic acid (HBEA, 60) and 4-hydroxybutyric acid (HBA, 63), Scheme 41).^171^

**Scheme 41:**
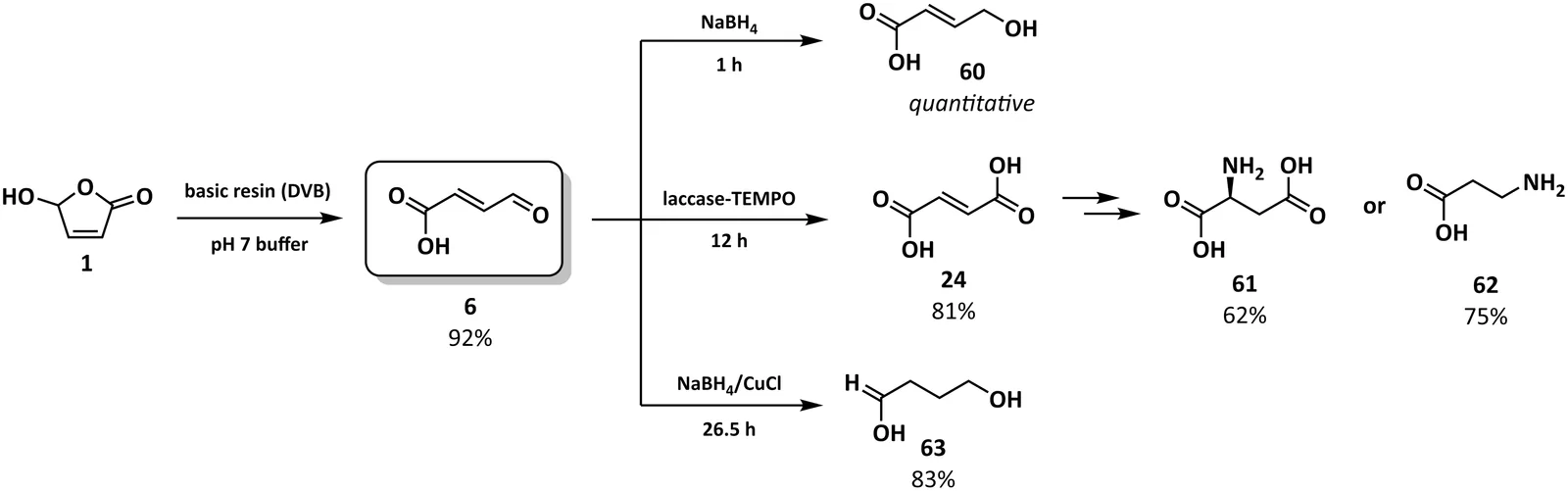
Ring-opening product of HFO.^171^

Compared to traditional thermal or strongly alkaline based methods, these resin- and enzyme-mediated approaches offer improved selectivity and higher yields under mild and environmentally benign conditions, representing a promising route for sustainable production of C4 dicarboxylic acids and their downstream derivatives.

Other pathways have been reported in 2000 by Porco *et al.*,^172^ where (*Z*)-*O*-methyloxime acids were efficiently prepared from HFO 1.^172,173^ Treatment of HFO 1 with aqueous methoxyamine hydrochloride (1.1 equiv.) afforded 4-(methoxyimino)-(2*Z*)-butenoic acid 64 in 92% yield (Scheme 42). The corresponding 4-(methoxyimino)-(2*Z*)-butenamide 65 was subsequently obtained in 88% yield *via* formation of the mixed anhydride of the acid, followed by reaction with aqueous ammonia. Furthermore, (2*Z*)-butenamide 65 could be fully isomerized to the corresponding (2*E*)-butenamide 66 in 81% yield under acidic conditions. These intermediates serve as valuable building blocks for the synthesis of antitumor macrolides, including *Lobatamide C* and related salicylate-based macrolide natural products.^172,173^

**Scheme 42:**
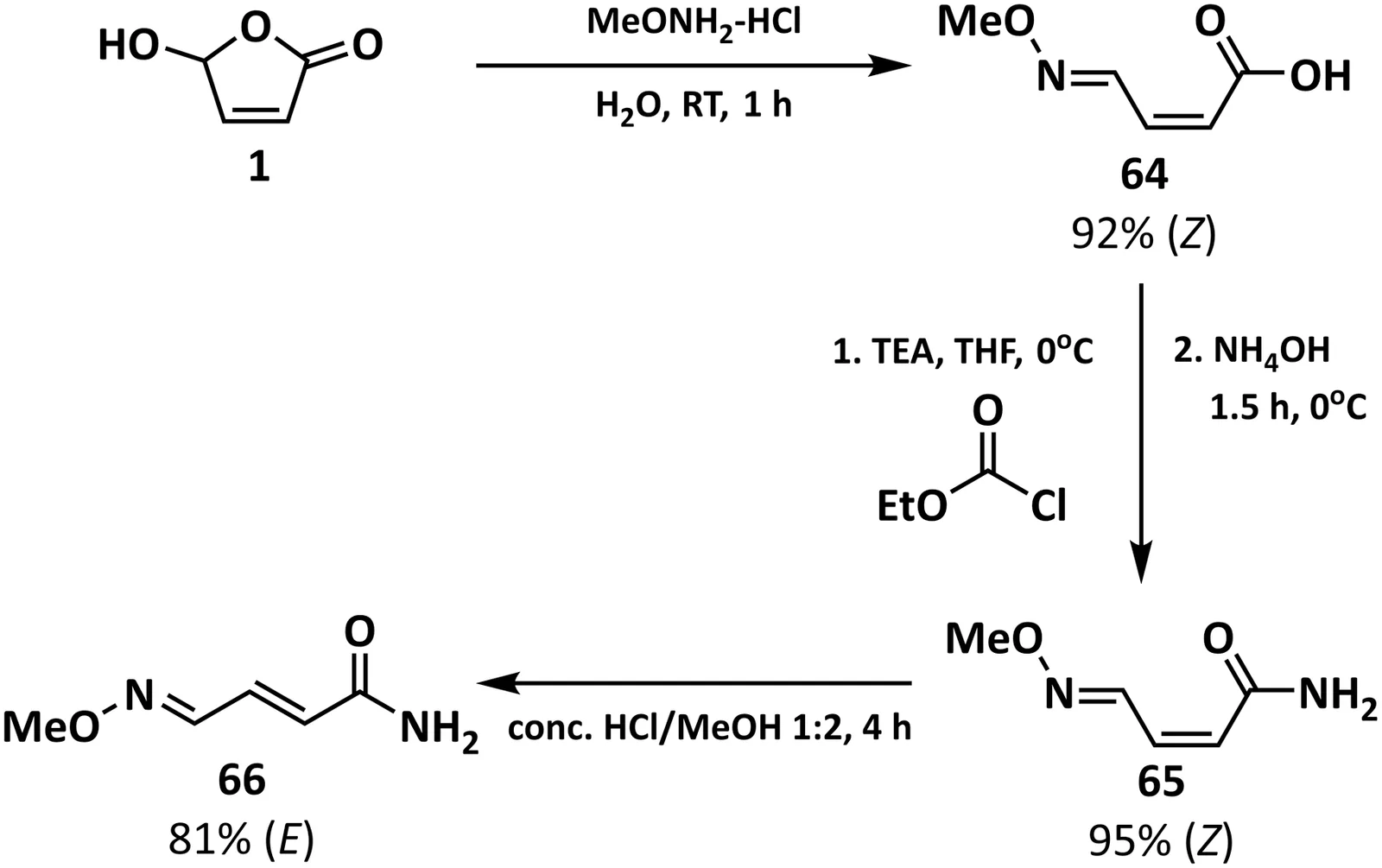
Formation of (*Z*)-*O*-methyloxime acid and (*E*)- and (*Z*)-*O*-methyloxime amides.^172,173^

### 3.7. Halogenation

Halogenation of 2(5*H*)-furanones has been widely explored to access functionalized derivatives useful as synthetic intermediates. In 1966, Schroeter, Appel and Schenck^29^ reported that treatment of HFO 1 with excess thionyl chloride afforded 5-chloro-2(5*H*)-furanone 67 in 45% yield,^29^ while later studies by Poskonin and coworkers (1997) improved this transformation using phosphorus pentachloride in Et_2_O, achieving yields up to 72% (Scheme 43).^79,174^

**Scheme 43:**
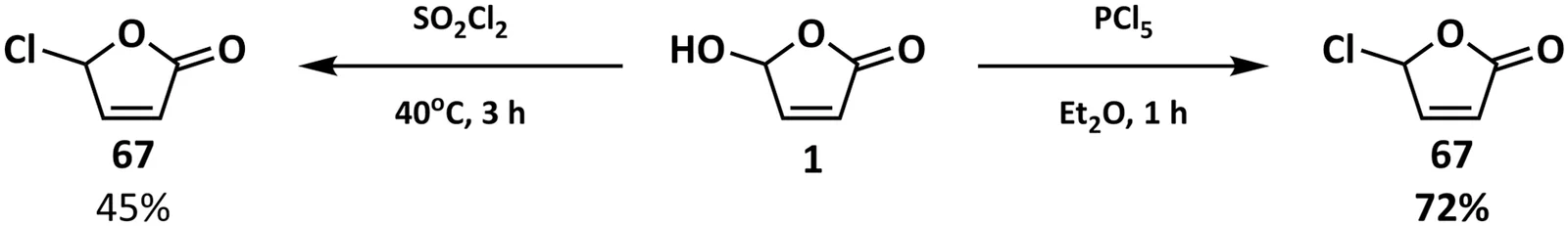
Chlorination of HFO *via* thionyl chloride or phosphorus pentachloride as chlorinating agent.^29,79,174^

Bromination strategies have also evolved over time. In 1996, Lattmann *et al.*^175^ described a dehydrobrominative bromination of 7d catalysed by a small amount of PBr_3_ at low temperature (4 °C).^175^ Stirring overnight, followed by continuous removal of HBr by distillation, affording the brominated product at the 4-position 68 in 95%, which upon recrystallization yielded colourless crystals (66%, Scheme 44). In 2013, Vasamasetty *et al.*^176^ reported that addition of 7d to bromine furnished 69 in 65% yield under controlled conditions at 0 °C to room temperature upon HBr elimination with pyridine (Scheme 44).^176^

**Scheme 44:**
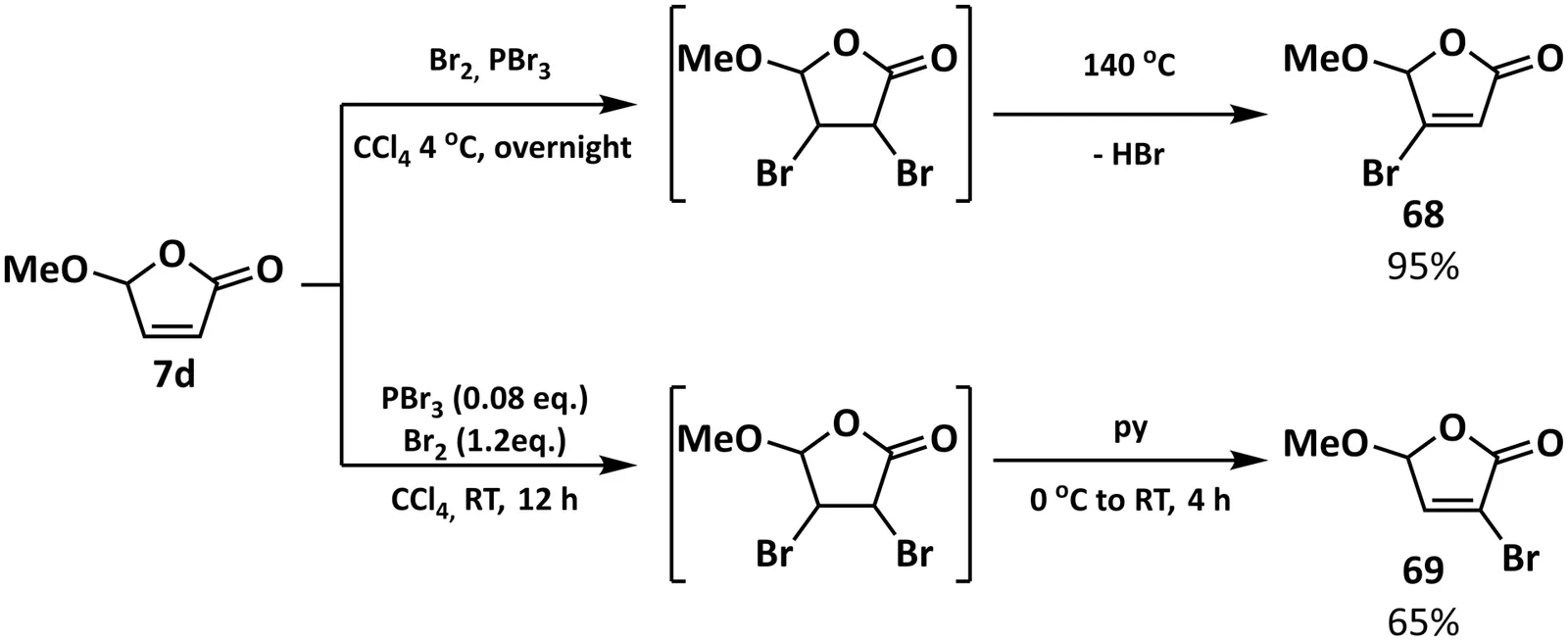
Bromination of 5-methoxy-2(5*H*)-furanone at 3- or 4-position depending on conditions.^175,176^

More recently, the Hoffmann group (2022) optimized this methodology using 5-ethoxy-2(5*H*)-furanone (5-ethoxybutenolide) and triethylamine, achieving brominated product 69 in 66% yield and enabling the synthesis of polymethine dyes with thiobarbituric and carboxylic acid functionalities (Scheme 45).^177^

**Scheme 45:**
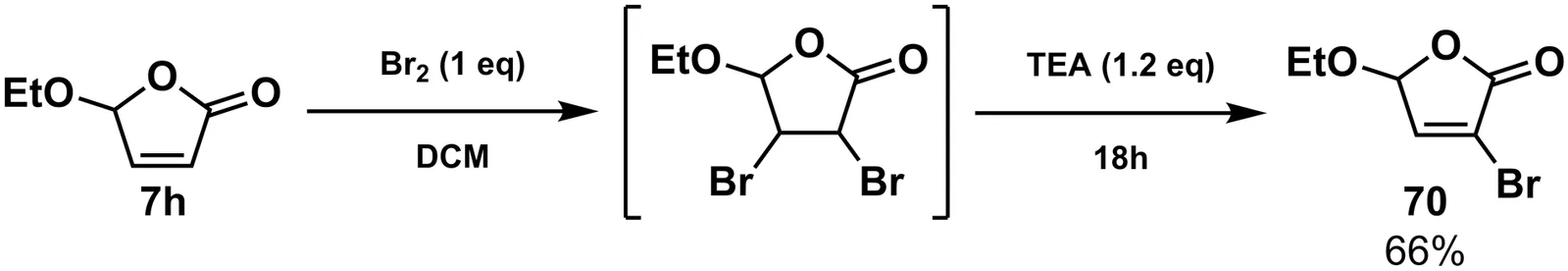
3-Bromination of 5-ethoxy-2(5*H*)-furanone.^177^

### 3.8. Amidation and ammonolysis of HFO

HFO is a versatile intermediate for the synthesis of amidofuranones and hydroxylactam derivatives through amidation and ammonolysis reactions. In 1997, Poskonin and coworkers reported the reaction of HFO 1 with amides such as furan-2-carboxamide or benzamide at 70 °C for 4–7 h to afford amidofuranones 71 and 72 in yields of 48% and 55%, respectively (Scheme 46).^79,174^

**Scheme 46:**
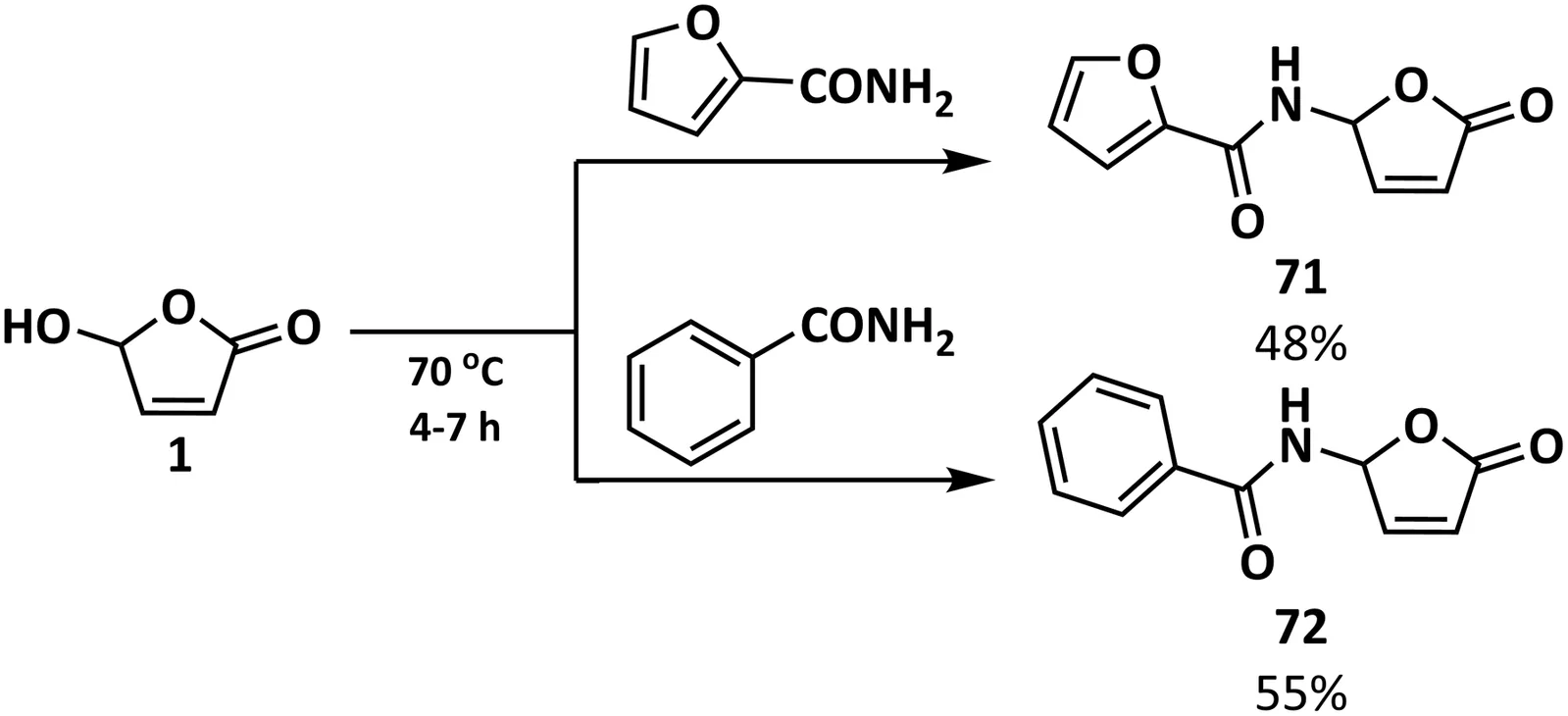
Direct amidation of HFO.^79,174^

Ammonolysis of HFO 1 is less common. In 1973, Farina *et al.*^178^ showed that 5-methoxybutenolide 7d reacts with excess concentrated aqueous ammonia, furnishing 5-hydroxy-3-pyrrolin-2-ones 73 in 65% yield (Scheme 47).^178^ Subsequently, Feringa and coworkers reported quantitative yields for the same reaction.^94,179^ Work-up must be carefully controlled below 25 °C to prevent ring-chain tautomerism between hydroxylactam and open-chain keto-amide forms. Subsequent acid catalysed condensation of methanol resulted in 74 (60%) when methanol was used as solvent.^178^

**Scheme 47:**
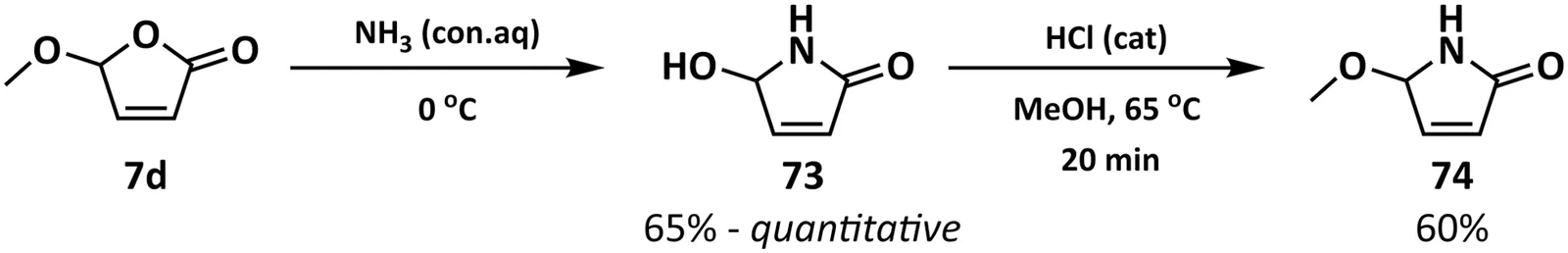
Ammonolysis of methoxy butanolide to 5-hydroxy-3-pyrrolin-2-ones and subsequent condensation of methanol to 5-methoxy-3-pyrrolin-2-ones.^94,178^

### 3.9. Substitution and condensation reactions

HFO and its derivatives also undergo substitution and condensation reactions to form more complex structures. Early studies by Schroeter *et al.*^29^ (1966) showed that treatment of HFO 1 with excess thionyl chloride affords HFO dimer 75 in 30% yield (Scheme 48).^29^ Later, Poskonin and coworkers (1997) demonstrated that self-condensation of HFO can be achieved by heating a chloroform solution in the presence of acid catalysts such as formic acid, MA, or cation-exchange acidic resins while removing H_2_O azeotropically, giving the HFO-dimer 75 in 40% yield.^79,174^

**Scheme 48:**
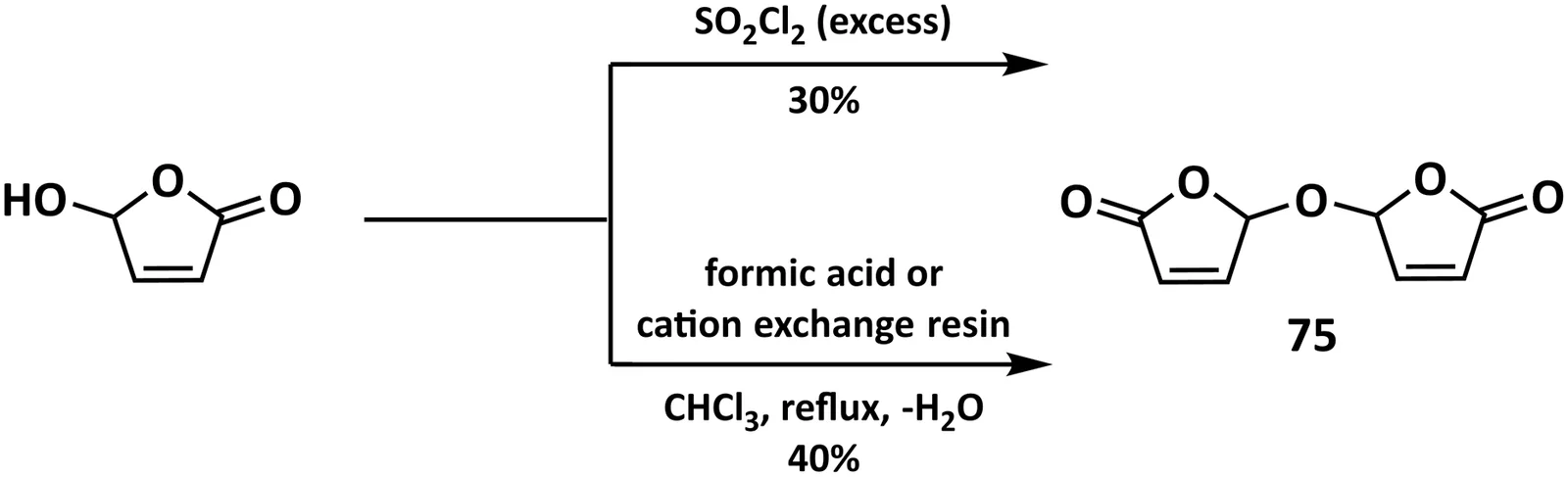
Direct condensation of HFO, forming dimer 5,5′-oxybis(furan-2(5*H*)-one).^29,79,174^

Condensation reactions involving heteroaromatic substrates were also investigated by the Poskonin group in 1997.^79,174^ For example, treating HFO 1 with 2-methylfuran in Et_2_O at room temperature, using a few drops of perchloric acid as a catalyst for 5 h, afforded the *cis*-configured gem-bis(4-(5-methyl-2-furyl)-2-butenoic acid) 76 in 13% yield (Scheme 49).^79,174^

**Scheme 49:**
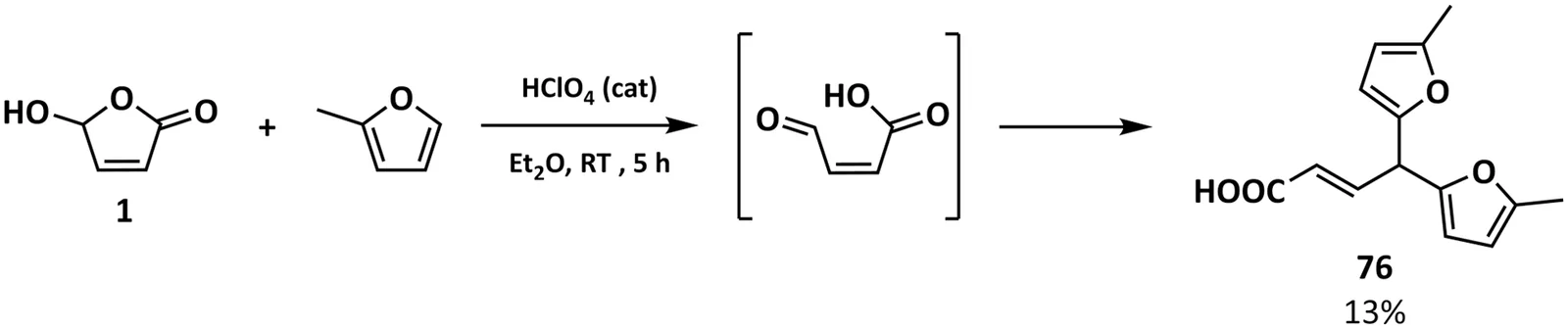
Substitution reaction of HFO with 2-methylfuran using perchloric acid.^79,174^

These findings highlight the versatility of HFO as scaffold for constructing dimeric and substituted derivatives. Such compounds can serve as valuable intermediates for further functionalisation and the synthesis of structurally complex and potentially bioactive molecules.^79,174^

### 3.10. Reactions of 5-menthyloxy-2-[5*H*]-furanone

Although catalytic approaches to asymmetric induction are widely studied, chiral auxiliary based stoichiometric methods remain important due to their high flexibility and selectivity. For this purpose, 5-menthyloxyfuran-2[5*H*]-one (5-menthyloxybutenolide) was pioneered to be a particularly interesting synthon, since both enantiomers are readily available from natural sources.^49–56,85,87^

Enantiomerically pure 5-menthyloxybutenolides are easily accessible through condensation (acetalization) of HFO with the corresponding menthol (Scheme 50A). Condensation with (−)-menthol ((1*R*,2*S*,5*R*)-5-Methyl-2-(propan-2-yl)cyclohexan-1-ol) results in a diastereomeric mixture of (5*R*)-5-menthyloxyfuran-2(5*H*)-one (7f) and (5*S*)-5-menthyloxyfuran-2(5*H*)-one (7k). For clarity, the major isomer 7f is hereafter referred to as (5*R*)-menthyloxybutenolide. This isomer can be selectively crystallized, while the minor isomer 7k readily epimerizes to 7f under acidic conditions.^49–56,87^

**Scheme 50:**
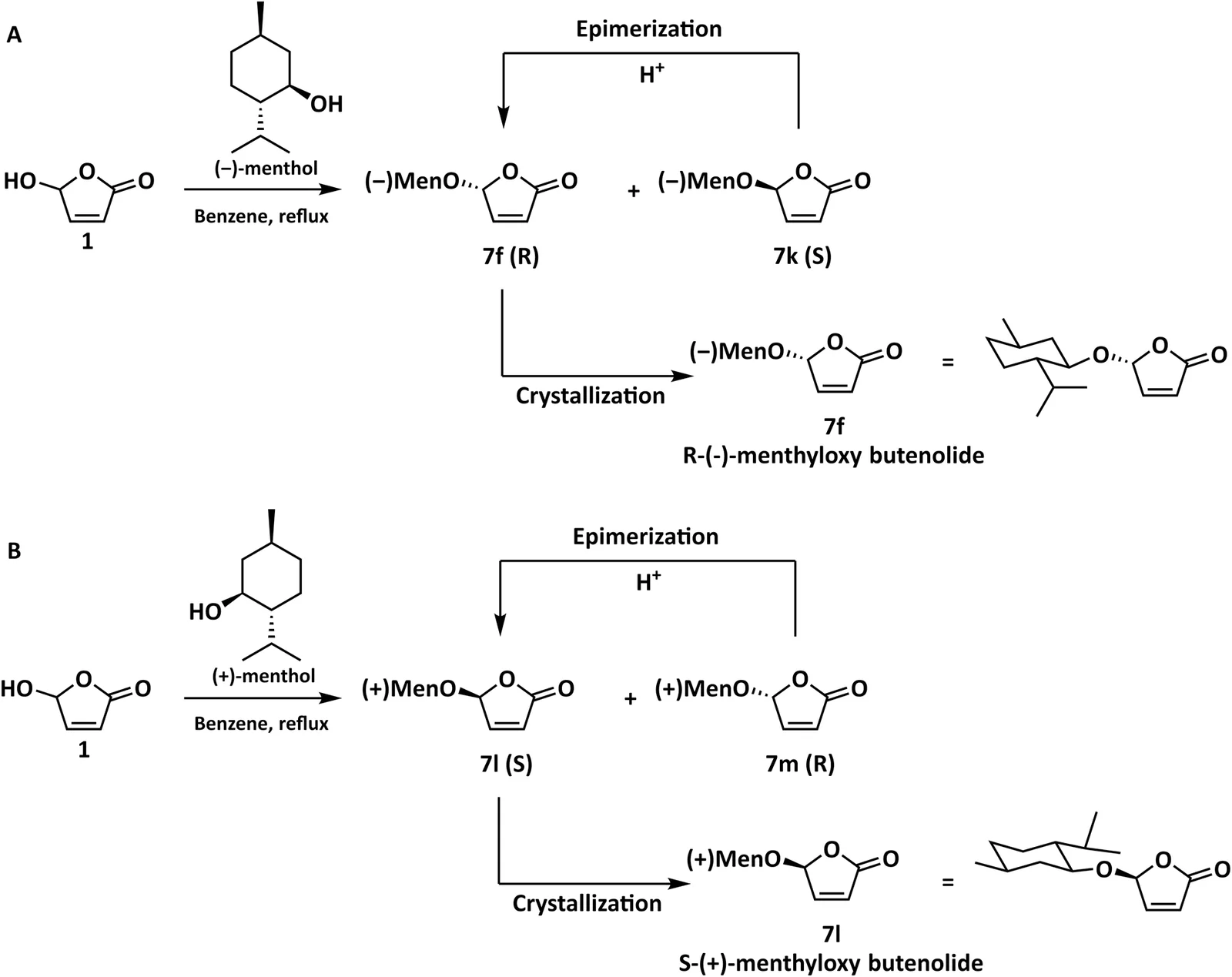
General synthesis of menthyloxybutenolide using (A) (−)-menthol, (B) (+)-menthol.^49–56,85,87^

Condensation with (+)-menthol ((1S,2R,5*S*)-2-isopropyl-5-methylcyclohexan-1-ol) analogously leads to preferential crystallization of the opposite enantiomer, thereby affording predominantly (5*S*)-menthyloxybutenolide (7l) instead of (5*R*)-menthyloxybutenolide (7m) (Scheme 50B).^50,180^ Notably, (−)-menthol is the naturally abundant and more readily available enantiomer, whereas (+)-menthol is less commonly accessible.

Importantly, the stereochemistry of the menthol auxiliary is retained upon attachment to the butenolide. Although the initial condensation can, in principle, generate up to four diastereomers (two for each menthol) due to the combination of the chiral menthol auxiliary and the newly formed stereocentre, crystallization and acid-promoted epimerization selectively funnel the mixture toward a single diastereomer. As (−)- and (+)-menthol are enantiomeric, their use ultimately provides access to a final pair of enantiomeric menthyloxybutenolides, in which all stereocentres, including those of the menthol moiety and the butenolide core, are inverted. Condensation with (−)-menthol affords predominantly the (5*R*)-menthyloxybutenolide (MFO), whereas the use of (+)-menthol provides access to the enantiomeric (5*S*)-menthyloxybutenolide, highlighting the straightforward access and role of the chiral auxiliary in controlling the stereochemical outcome. The incorporation of this bulky menthyloxy group has enabled the use of 5-menthyloxybutenolides as chiral building blocks for the induction of diastereoselectivity in numerous asymmetric transformations, including Michael reactions,^50–52,55,97,181–191^ cyclopropanations,^192^ Diels–Alder reactions,^49,54,193^ dipolar^193–197^ and radical additions,^198–200^ tandem (domino) reactions,^201^ and *cis*-dihydroxylations^202^ (Fig. 12). A review on the use of menthol by Oertling *et al.*^87^ covers many of these transformations; for completeness, representative examples are discussed below.^87^

**Fig. 12:**
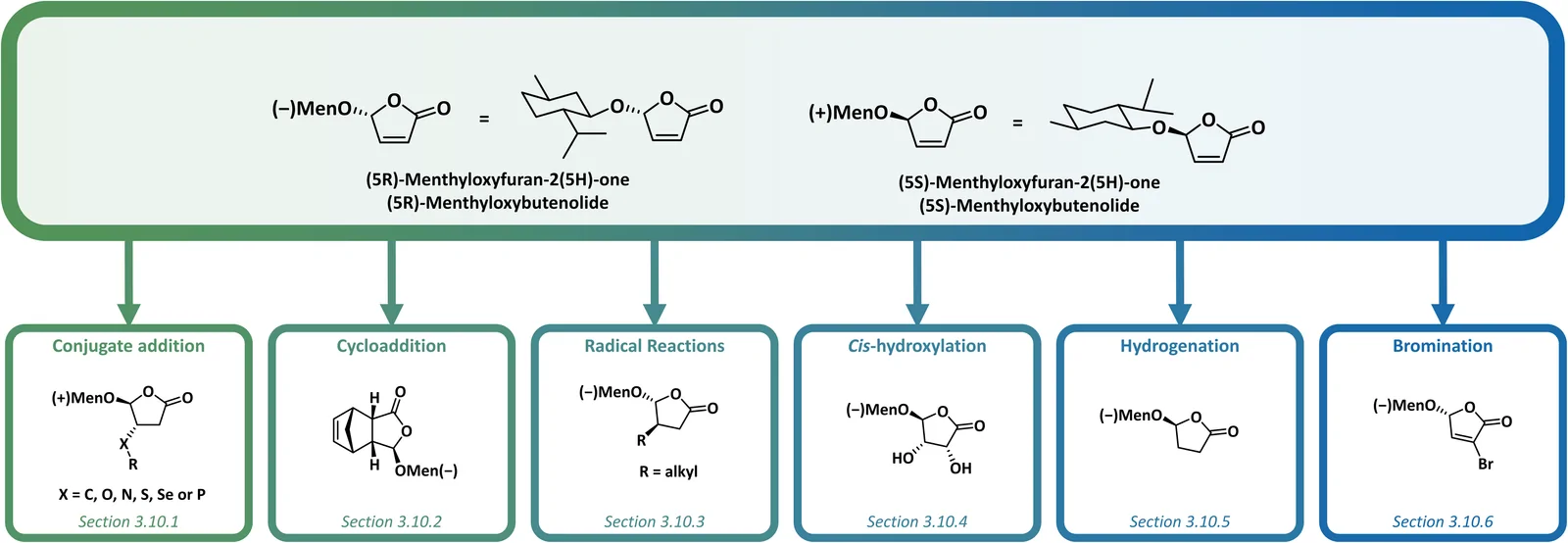
Various transformations on 5-menthyloxybutenolide covered in different sections of this section.

#### 3.10.1. Conjugate additions

##### 3.10.1.1. Conjugate additions of carbon nucleophiles

Mulzer *et al.*^181^ utilised vinyl Grignard reagent in combination with copper iodide to achieve a 1,4-addition with (5*S*)-menthyloxybutenolide 7l, affording the desired lactone 77 (Scheme 51).^181^ The addition proceeded from the less hindered side due to the shielding effect of the menthyloxy substituent. Further reactions on the obtained lactone led to the C-fragment of cobyric acid 78.

**Scheme 51:**
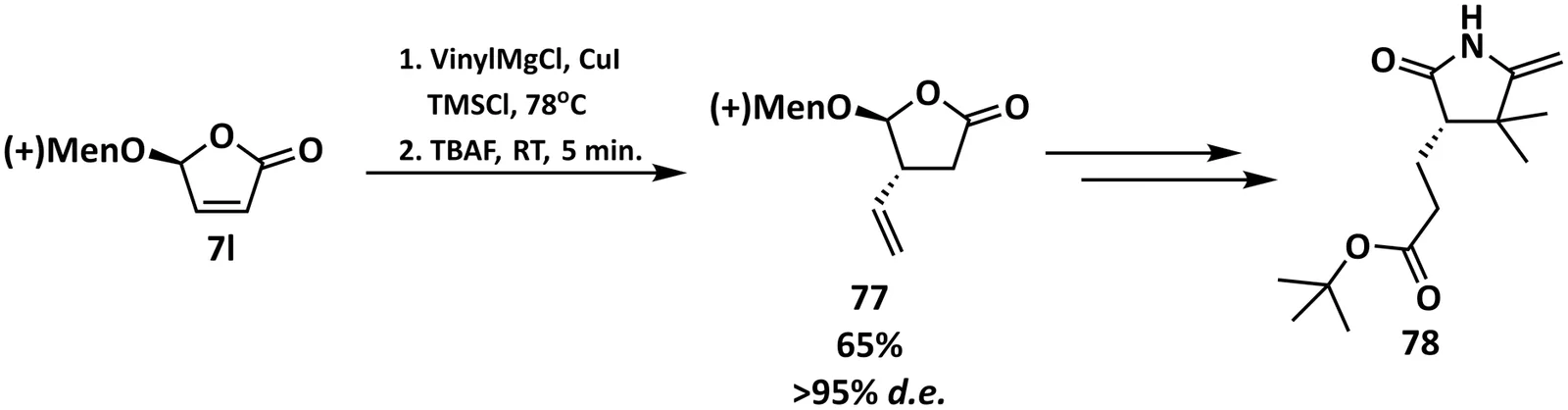
1,4-addition of vinyl cuprate to (5*S*)-menthyloxybutenolide, and derivatization towards the C-fragment of cobyric acid.^181^

Conjugate tandem addition of sulphur-stabilized carbanions to (5*R*)-menthyloxybutenolide 7f has been utilised for the synthesis of a series of lignans (Scheme 52).^53,97,182,183,191,203,204^ The double bond readily participates in 1,4-additions with these nucleophiles. The resulting enolate intermediate is subsequently trapped *in situ* by electrophiles such as aldehydes or halogenating agents. The double bond addition proceeds with complete stereocontrol, affording lactones in which the substituents are introduced in an all-*trans* relationship (Scheme 52A). Pelter *et al.* utilised this strategy for the synthesis of lignan (−)-4-deoxyisopodophyllotoxin 81 in 29% overall yield, where the desulfurization of 80 to 79a was performed using NiCl_2_/NaBH_4_ (Scheme 52B).^182,183,205,206^

**Scheme 52:**
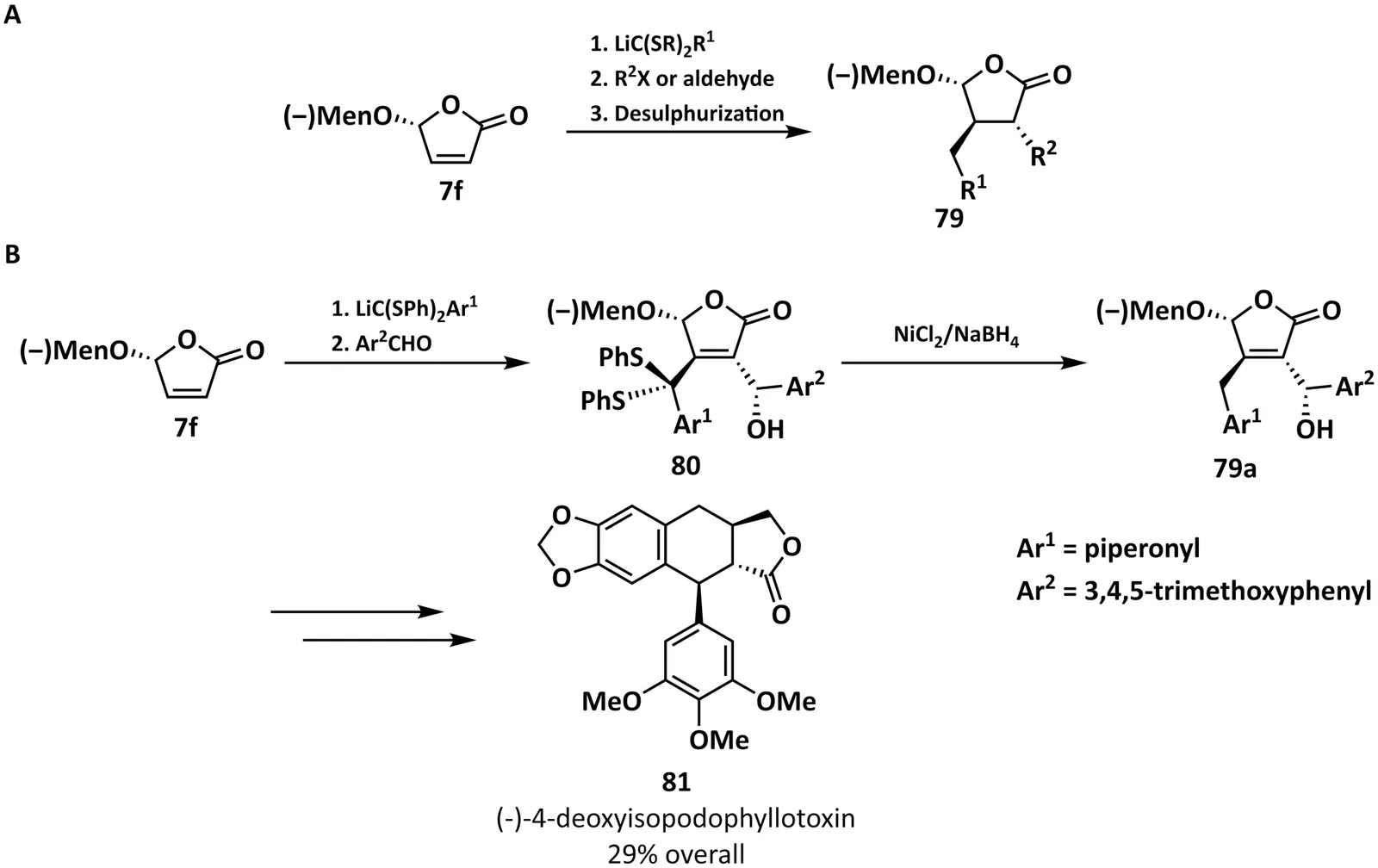
(A) Synthesis of all-*trans* stereo-triad *via* tandem addition, (B) enantioselective synthesis of (−)-4-deoxyisopodophyllotoxin from (5*R*)-menthyloxybutenolide *via* tandem addition.^182,183^

Kang *et al.*^185^ investigated the 1,4-addition of activated methylene compounds to (5*R*)-menthyloxybutenolide 7f.^185^ High conversions and diastereomerically pure products 82a-d were obtained under mild conditions (room temperature, DMF, catalytic NaOEt, (Scheme 53A). As observed previously, nucleophilic addition occurs from the face opposite to the menthyloxy substituent. The resulting nitro derivative 82a was further transformed into enantiomerically pure nitronic esters 84*via O*-alkylation with alkyl halides in the presence of K_2_CO_3_ and DMF (Scheme 53B).^184^

**Scheme 53:**
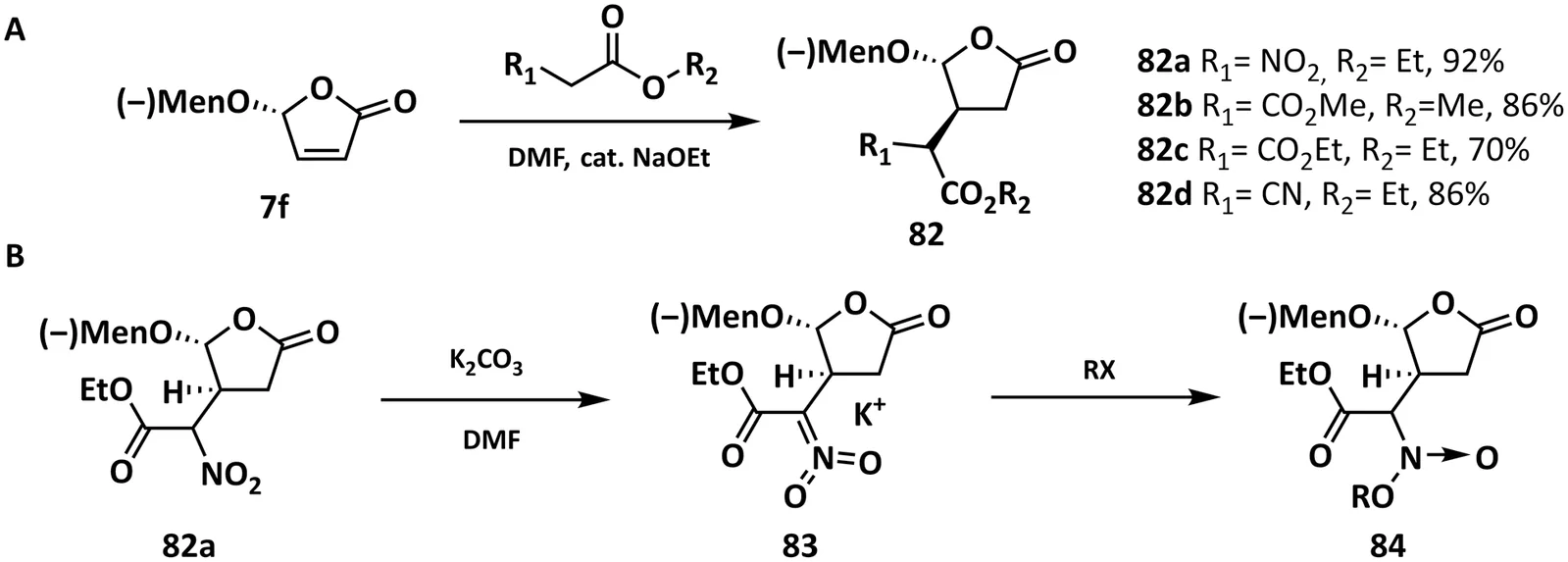
(A) 1,4-addition of activated methylene compounds to (5*R*)-menthyloxy butenolide, (B) subsequent transformation into chiral nitronic esters *via O*-alkylation.^184,185^

##### 3.10.1.2. Conjugate additions of O-nucleophiles

Kang *et al.*^185^ also reported asymmetric conjugate additions of oxygen-containing nucleophiles to (5*R*)-menthyloxybutenolide 7f, affording products 85a–e (Scheme 54).^185^ Alcohols do not readily undergo conjugate addition to butenolides; however, upon addition of catalytic amounts of metallic sodium to an excess of primary alcohol, sodium alkoxides are formed, which readily undergo asymmetric 1,4-addition to the butenolide upon stirring.^185^

**Scheme 54:**
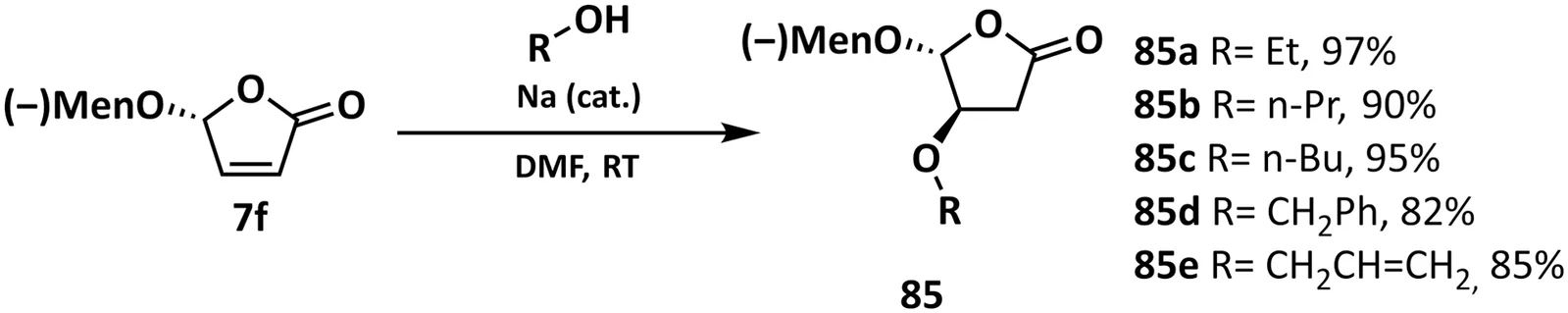
1,4-addition of primary alcohols to (5*R*)-menthyloxy butenolide.^42^

##### 3.10.1.3. Conjugate additions of amines

Amines add readily to the double bond of (5*R*)-menthyloxybutenolide 7f at the β-position without opening of the lactone, as reported by Feringa and coworkers in several studies (1988–2002).^52,55,186^ Simple stirring at room temperature in DCM or DMF affords 86a-c in excellent yield (>95%) and moderate to excellent isolated yields within short reaction times (0.5–8 h, Scheme 55A, B). The addition of the amine proceeds in a diastereoselective manner, producing enantiomerically pure products, where the amine adds to the ring anti to the menthyloxy substituent.

**Scheme 55:**
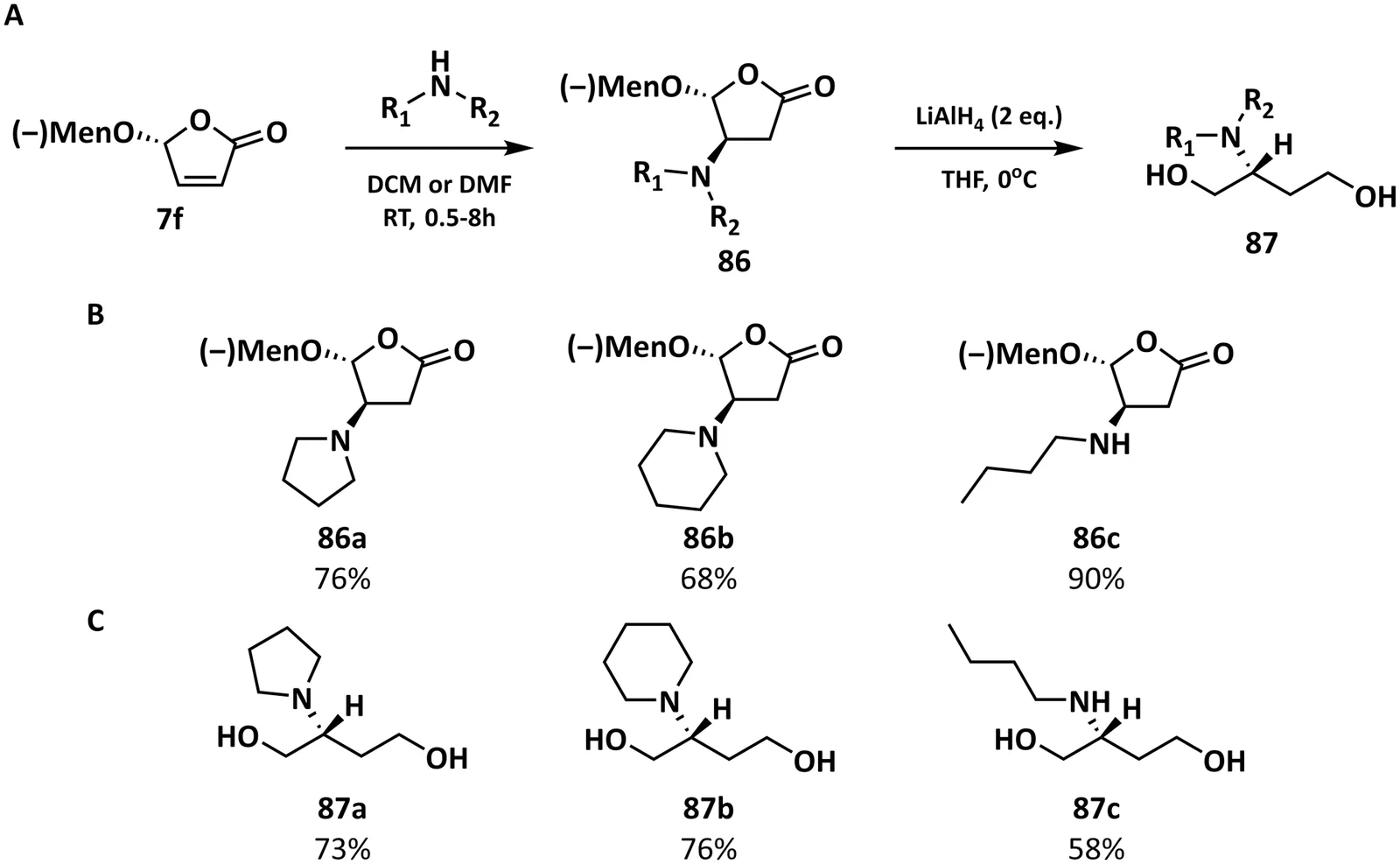
(A) Conjugate addition of amines to menthyloxybutenolide and subsequent reduction towards chiral aminodiols, selected examples (isolated yields given) for (B) diastereselective amine addition, (C) reductions towards chiral aminodiols.^52^

Since the 1,4-addition of amines is a reversible process, purification *via* distillation leads to losses in yield; therefore, the products are typically isolated by crystallization.^52^ Further reduction using LiAlH_4_ provided enantiomerically pure aminodiols 87a–c (Scheme 55A and C).^52^

By employing a 2 : 1 ratio of (5*R*)-menthyloxybutenolide 7f to piperazine, double addition reactions can be achieved under similar conditions as described above (stirring for 1 h in DMF at room temperature, Scheme 56).^186^ It was found that unlike for monoamine additions, this diamine addition was not completely stereoselective, however after recrystallization the enantiomerically pure adduct 88 was still obtained in 83% yield. Subsequent oxidation using pyridinium dichromate in acetone for 24 h afforded the tetraacid product 89 in 21% isolated yield. This methodology allows for structural flexibility of the diamines and affords multidentate chelating ligands capable of complexing *e.g.* europium ions in aqueous solution and are therefore highly suitable as water-soluble chiral shift reagents for the determination of enantiomeric excess of unprotected amino acids.^186^

**Scheme 56:**
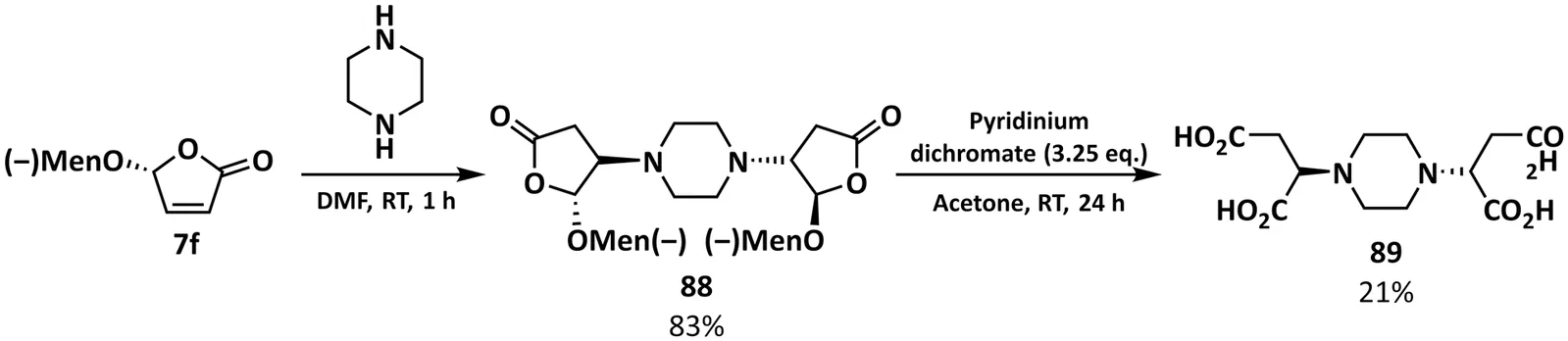
Synthesis of chiral ligand for Europium shift reagents by 1,4-addition of (5*R*)-menthyloxybutenolide with piperazine, and subsequent oxidation using pyridinium dichromate (isolated yields given).^186^

Similarly, they showed that conjugate additions of amines to racemic 5-methoxybutenolide 7d afforded the corresponding racemic aminolactones (Scheme 57).^55,187^ Here, again the amine adds to the ring anti to the methoxy substituent. Addition of ethanolamine to racemic 5-methoxybutenolide 7d afforded the corresponding racemic amino lactone 90 in 70% isolated yield.^55^ Similarly, piperazine reacted with two equivalents of 5-methoxybutenolide 7d to provide the diadduct 91 in 95% isolated yield.^55^ Remarkably, this transformation led to the formation of a single diastereomer despite the use of a racemic starting material. The 1,4-addition of tryptamine to alkoxybutenolides provided attractive precursors for various indole alkaloids.^55^ A facile, stereoselective, and nearly quantitative addition of tryptamine to 7d was observed, affording the *trans* adducts 92 in 97% isolated yield within only 1 h.^55^

**Scheme 57:**
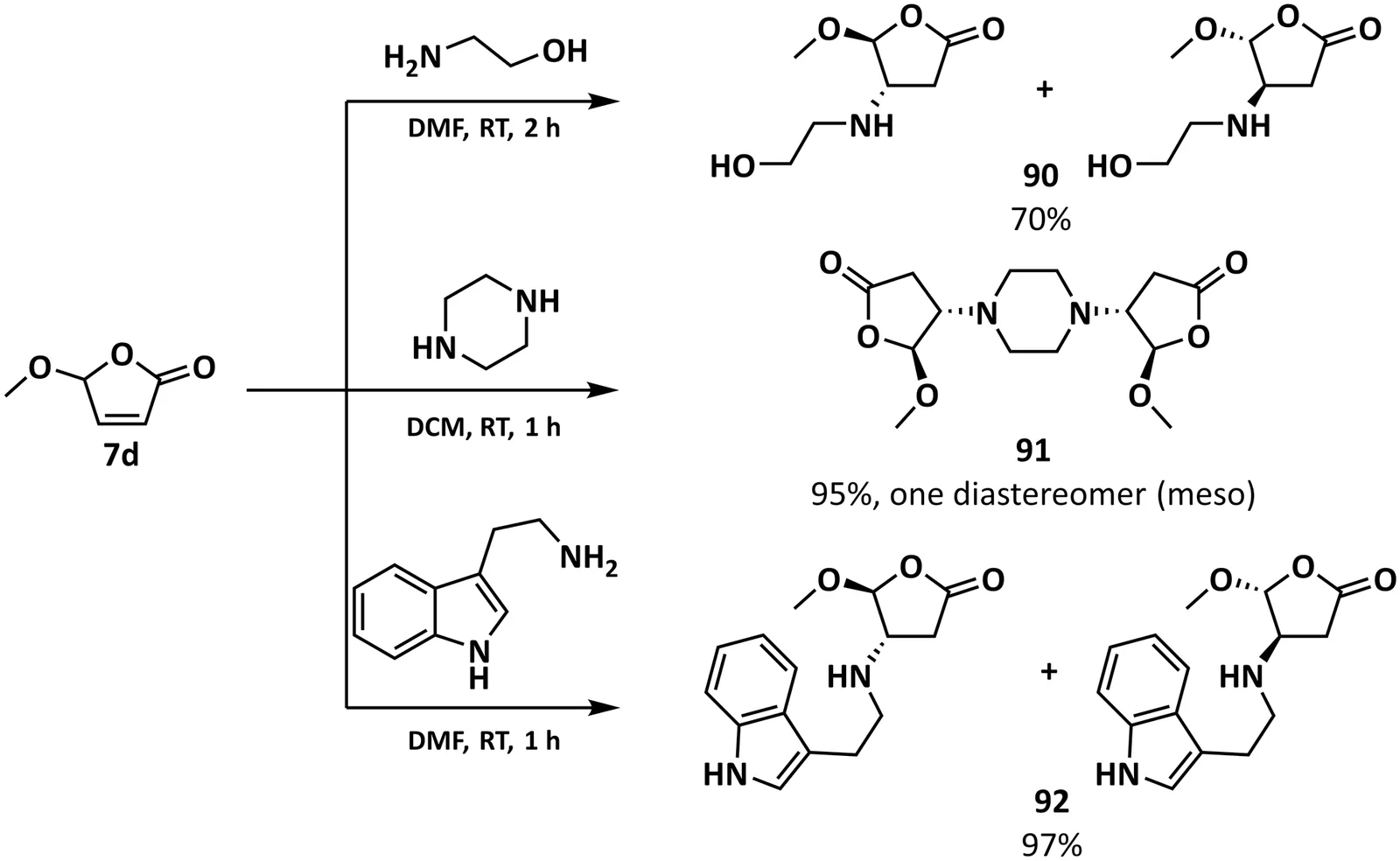
1,4-additions of amines to racemic 5-methoxybutenolide 7d.^55^

##### 3.10.1.4. Conjugate addition of thiols and selenides

Addition of thiols to butenolide double bonds proceeds with quantitative conversions at room temperature in the presence of triethylamine.^51,207^ Under these conditions, complete diastereoselective Michael-type addition of thiophenols to (5*R*)-menthyloxybutenolide 7f was observed, affording 93 (Scheme 58). These adducts were utilised to synthesize enantiomerically pure (*R*)-3,4-epoxy-1-butanol. Reduction with LiAlH_4_ resulted in the (*R*)-diol 94 in 81% yield, where the menthol auxiliary could be recovered by distillation in 80–90%. Subsequent alkylation using trimethyloxonium tetrafluoroborate generated the sulphonium salt intermediate 95, followed by base treatment provided the enantiomerically pure (*R*)-3,4-epoxy-1-butanol 96 in 71% (Scheme 58).^51^ Starting from (5*S*)-menthyloxybutenolide 7l would afford (*S*)-3,4-epoxy-1-butanol.

**Scheme 58:**
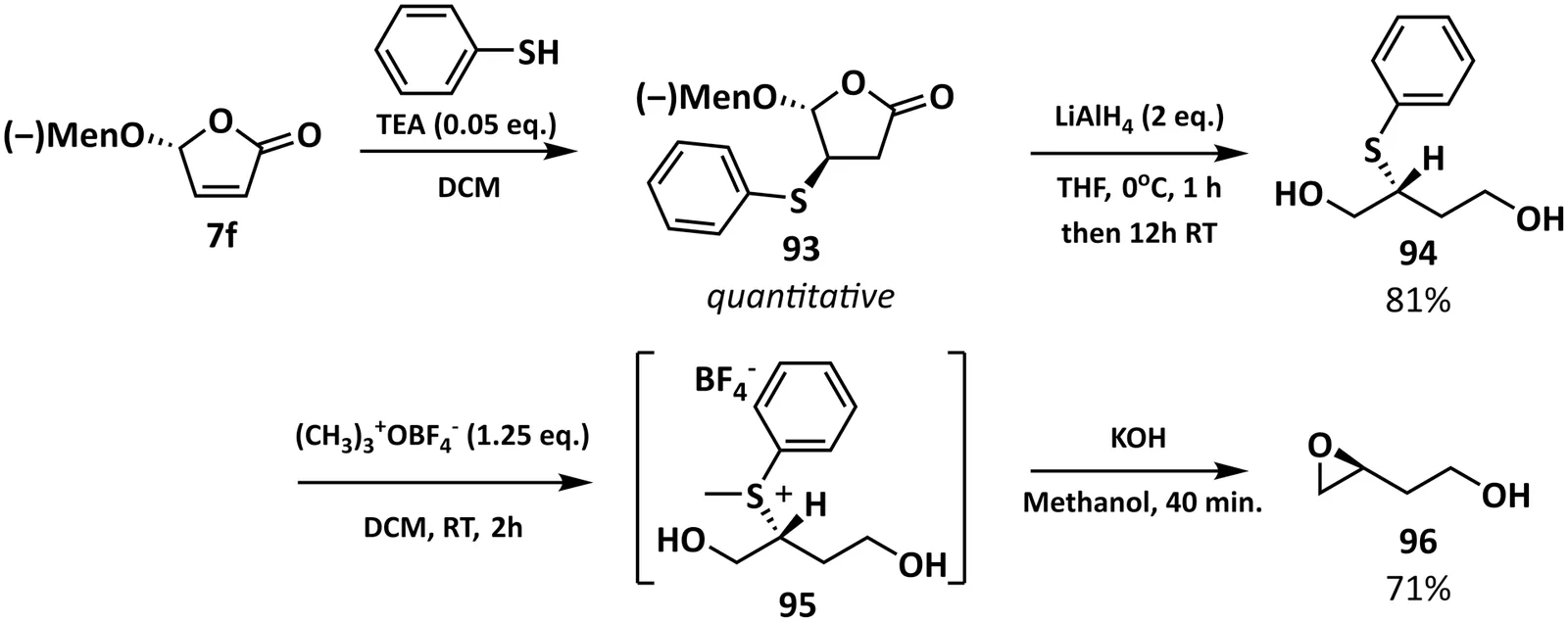
Conjugate thiol addition to (5*R*)-menthyloxybutenolide and subsequent derivatization to enantiomerically pure 3,4-epoxy-1-butanol *via* reduction with LiAlH_4_ and alkylation using trimethyloxonium tetrafluoroborate.^51^

For the synthesis of chiral 1,4-butanols Feringa and coworkers (1992) utilised lithiated benzyl mercaptan as nucleophile and methyl iodide as quenching agent, affording the desired product 97 in quantitative yield as a single stereoisomer (Scheme 59).^50^ However, this method could not be generalized to other mercaptans or alkyl halides.

**Scheme 59:**
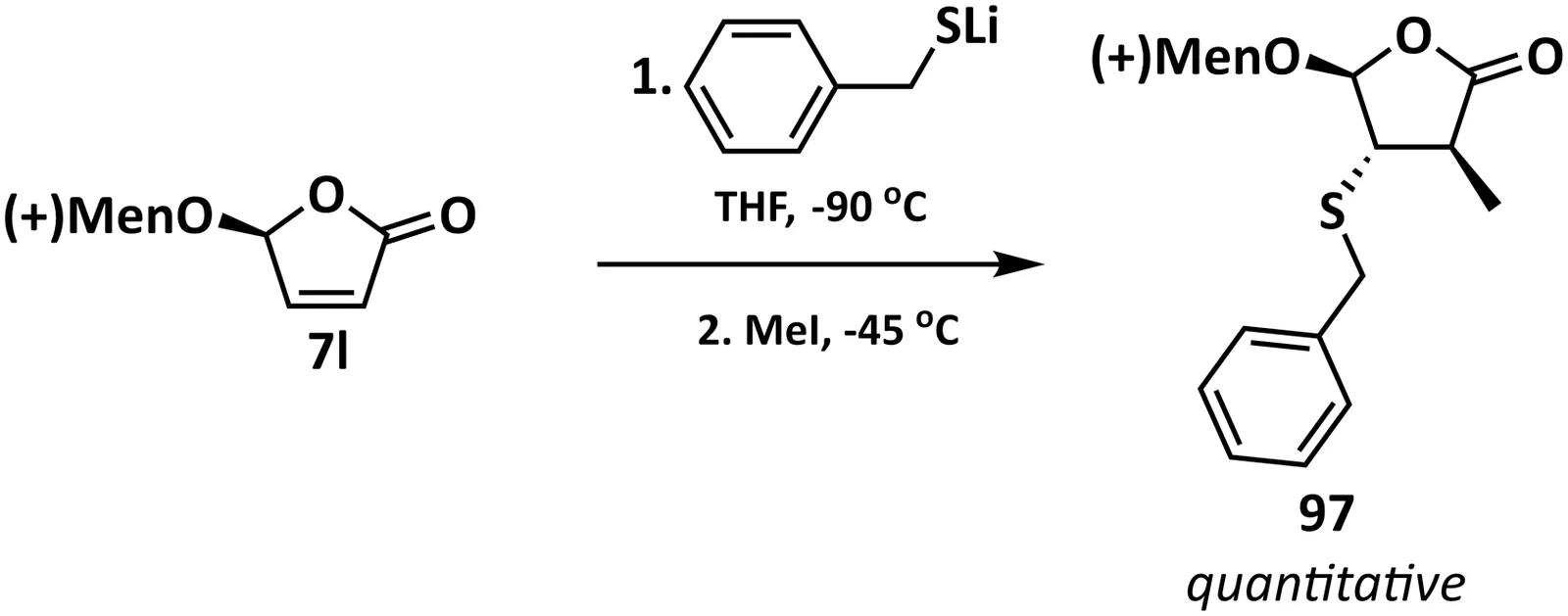
Tandem 1,4-addition of lithiated benzylmercaptan followed lactone-enolate methylation.^50^

When a solution of lithium phenylselenide was treated with (5*R*)-menthyloxybutenolide and cinnamaldehyde, the Michael-aldol product 98 was obtained in high yield (80%) and excellent diasteroselectivity (>99% de) after quenching with saturated aq. NH_4_Cl solution, forming three new stereocentres in a one pot procedure (Scheme 60).^188^

**Scheme 60:**
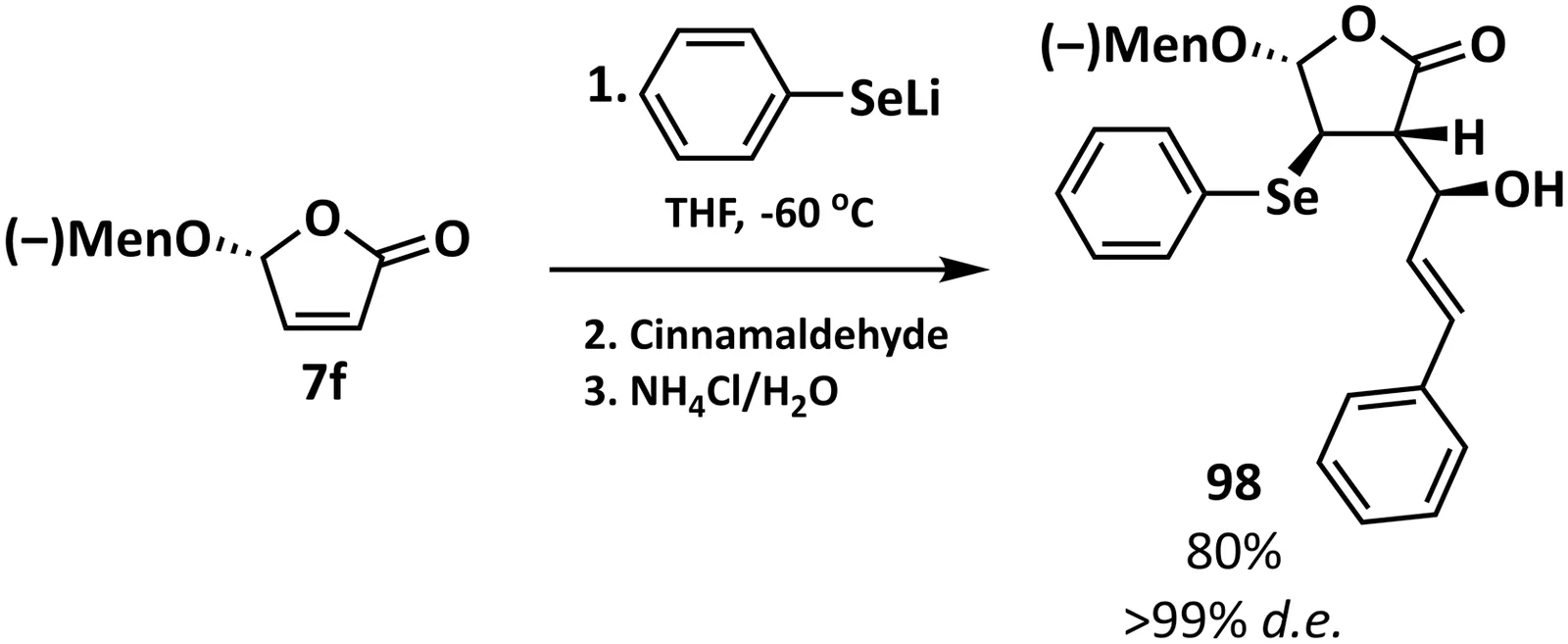
Tandem Michael-Aldol reaction between lithium phenylselenide, cinnamaldehyde and (5*R*)-menthyloxybutenolide.^188^

##### 3.10.1.5. Conjugate addition of phosphorus nucleophiles

Jansen and Feringa (1990) reported the synthesis of enantiopure (S,S)-Chiraphos 100 utilising (5*R*)-menthyloxybutenolide 7f as the starting material (Scheme 61).^189^ The one-pot sequential 1,4-addition and quenching reaction with lithio diphenylphosphide and diphenylphosphine chloride affords the all-*trans* lactone 99 as a single diastereomer in 92% yield. Subsequent reduction, tosylation, and further reduction afford (S,S)-Chiraphos in 35% overall yield.^189^

**Scheme 61:**
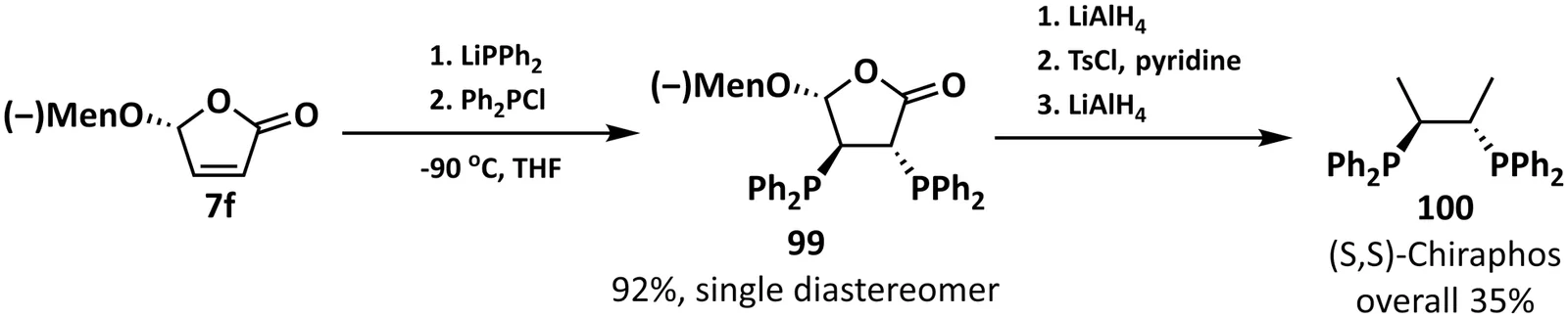
Synthesis of enantiopure (*S*,*S*)-chiraphos by Michael addition of lithio diphenylphosphide to (5*R*)-menthyloxybutenolide, followed by reduction, tosylation, and further reduction.^189^

#### 3.10.2. Cycloadditions

##### 3.10.2.1. Diels–Alder cycloaddition

5-Menthyloxybutenolide readily participates in Diels–Alder cycloadditions as dienophile with a variety of cyclic and acyclic dienes, acting as a chiral analogue of MA.^49^ These reactions take place with strict π-face selectivity as shown schematically for cyclopentadiene in Fig. 13. Feringa and coworkers reported that addition of cyclopentadiene showed highly stereoselective addition at the less hindered face of (5*R*)-menthyloxybutenolide 7f, yielding the endo product 101 (Scheme 62A). Subsequent hydrolysis or methanolysis affords enantiomerically pure hydroxy- or methoxy-substituted lactones 102.^49,54^

**Fig. 13:**
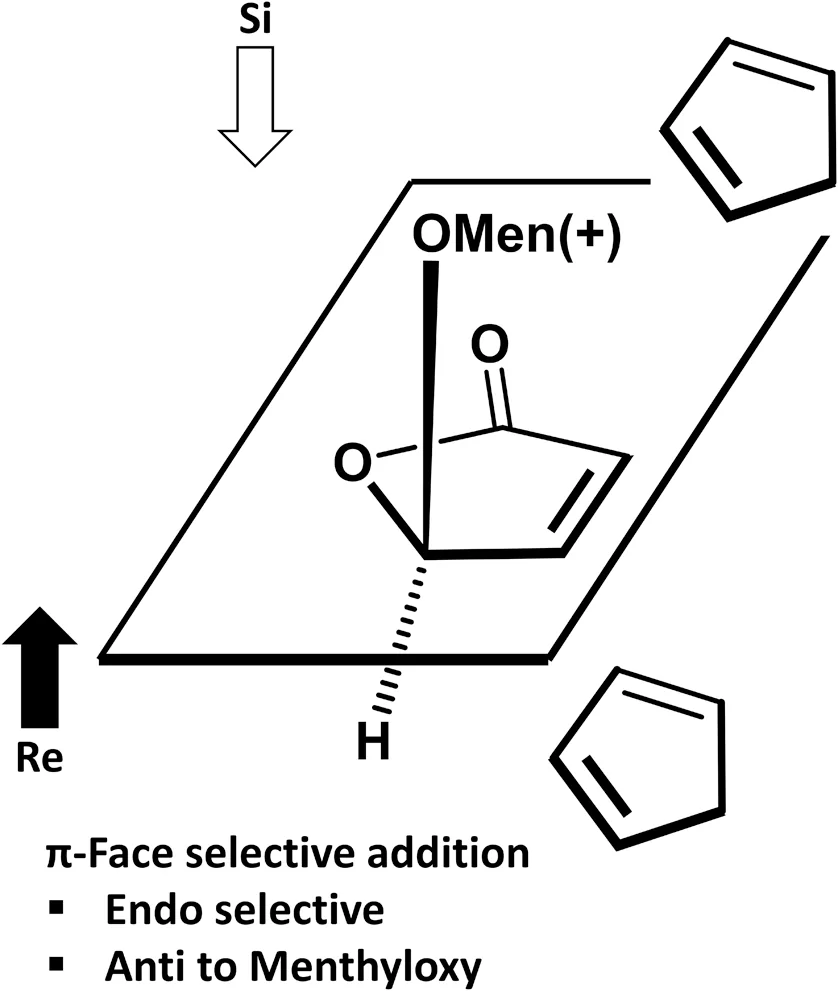
π-Face selective Diels–Alder addition of cyclopentadiene to (5*S*)-menthyloxybutenolide.^49^ Adapted from ref. 49 with permission from the American Chemical Society (ACS), © 2002.

**Scheme 62:**
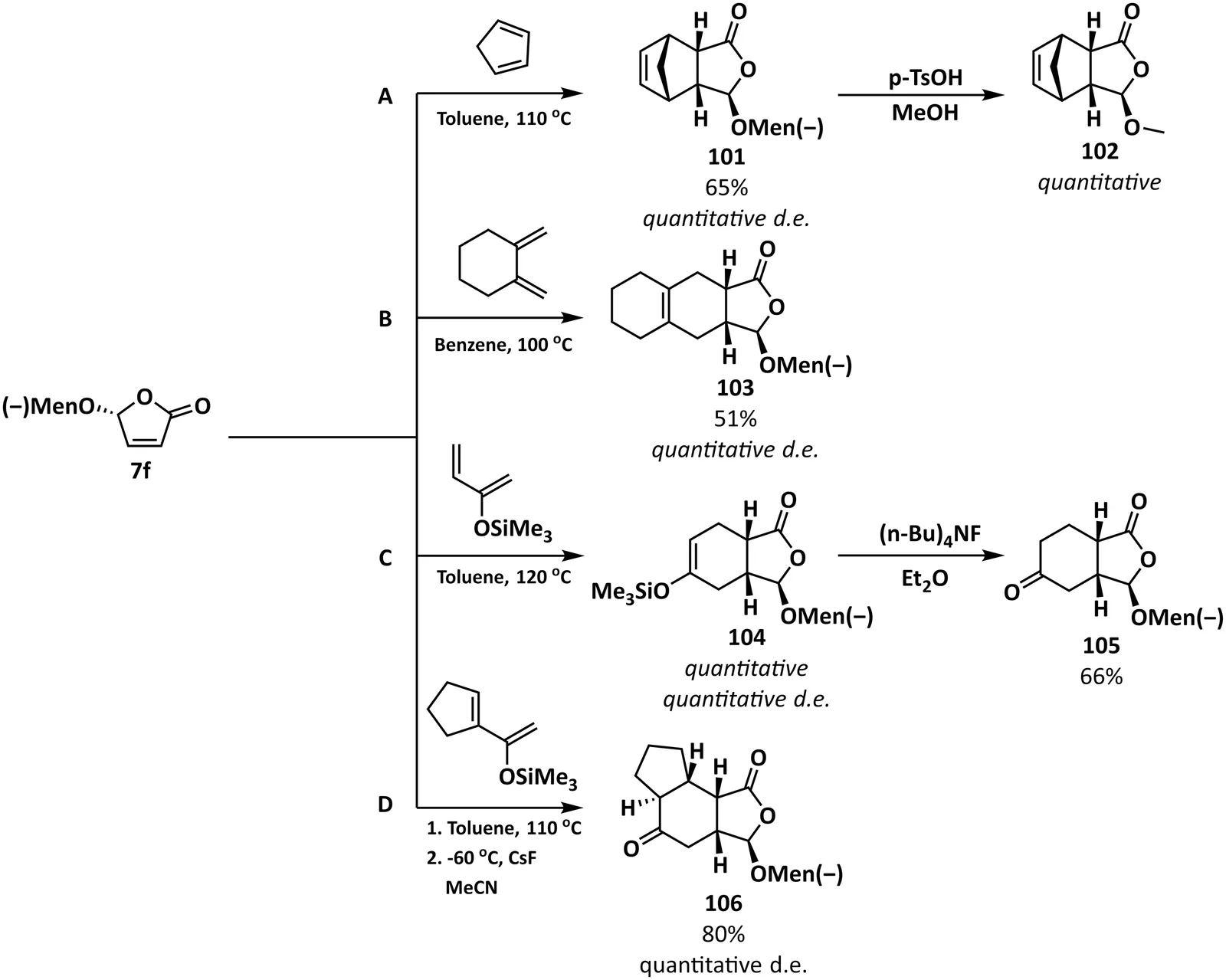
Diels-Alder reactions on (5*R*)-menthyloxybutenolide.^49,54,193^

In an effort to prepare optically active decalines, asymmetric Diels–Alder reactions with the exocyclic diene 1,2-bis(methylene)cyclohexane were investigated. This reaction, performed in benzene in a sealed tube at 100 °C for 42 h, afforded a single enantiomer of 103 in 51% yield (Scheme 62B).^54^

The Diels–Alder reactions were significantly accelerated when more activated dienes, such as silyl enol ethers, were employed. For example, addition of 2-trimethylsilyloxy-1,3-butadiene to 7f afforded 104 in quantitative conversion and complete stereoselectivity (Scheme 62C). Subsequent treatment with tetrabutyl ammonium fluoride in Et_2_O yielded the 3,4-disubstituted cyclohexanone 105 as a single isomer in 66% yield.^54^ In a similar fashion, four new stereogenic centres were introduced in a one-pot sequence *via* cycloaddition and desilylation of 7f with CsF in wet MeCN, resulting in diastereomerically pure 106 (Scheme 62D).^193^

##### 3.10.2.2. 1,3-dipolar cycloadditions

1,3-Dipolar cycloadditions are valuable transformations in organic synthesis for the simultaneous introduction of multiple stereocentres with high selectivity (Scheme 57). Feringa and coworkers (1988–1994) demonstrated the addition of various dipoles, including diazo compounds, nitrile oxides, nitrones and azomethine ylides, to (5*R*)-menthyloxybutenolide 7d.^193–195,197^

Addition of ethyl diazoacetate proceeded with complete diastereofacial- and regio-selectivity, affording enantiomerically pure 107 (67%, Scheme 63A).^195^ Nitrile oxide additions occurred with complete diastereoselectivity from the π-face opposite to the menthyloxy substituent, although two regioisomers 108 and 109 were formed in a 92 : 8 ratio (Scheme 63B).^194^

**Scheme 63:**
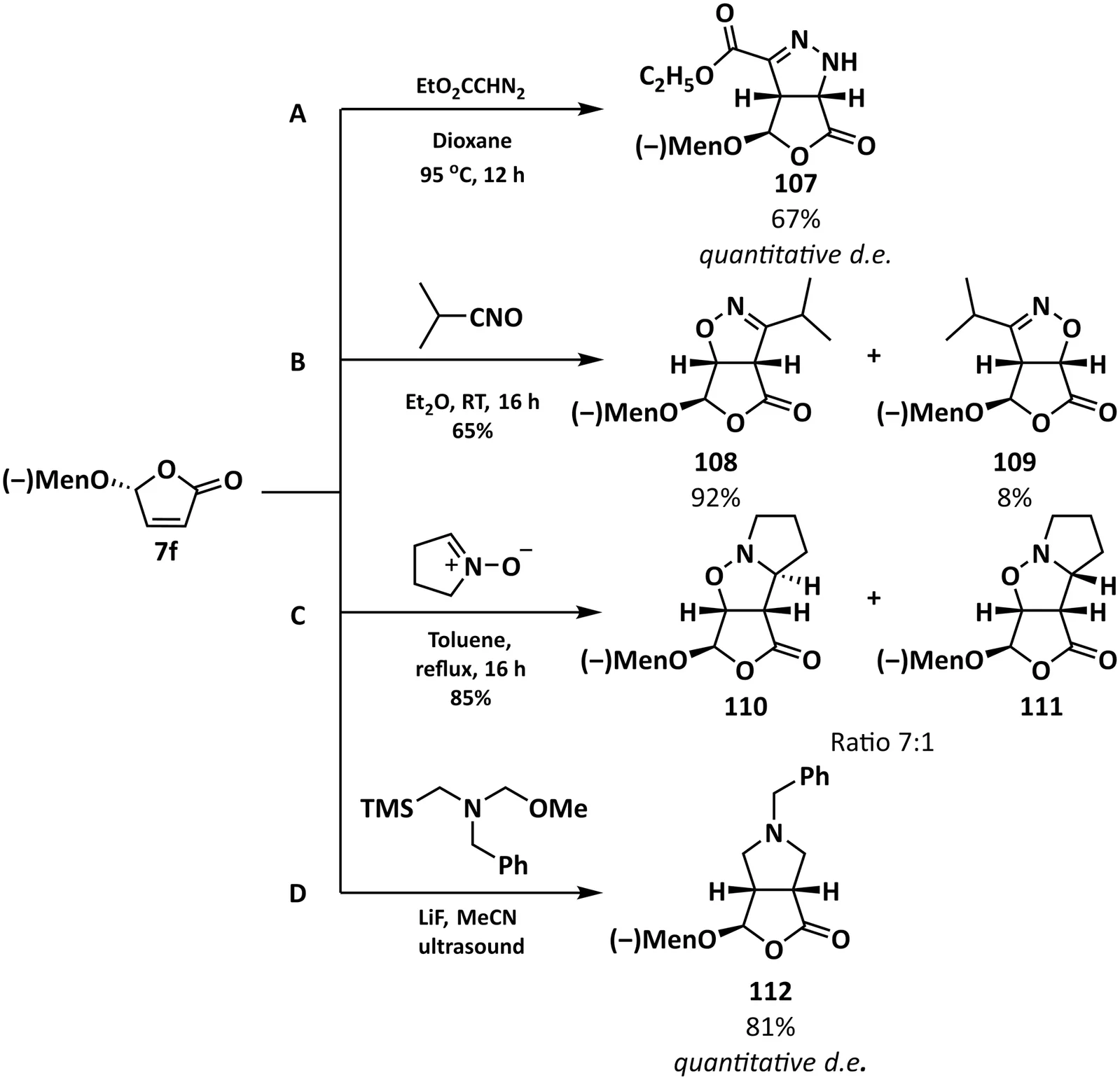
1,3-Dipolar cycloadditions on (5*R*)-menthyloxy butenolide.^194,195^

The dipolar addition of dihydropyrrole oxide afforded cycloadducts 110 and 111 in 85% yield with a 7 : 1 diasteromeric ratio. In this case, the major product resulted from an exo approach, while the minor product arose from an endo approach (Scheme 63C).^194^

Azomethane ylide addition was performed under heterogeneous conditions using sonication, as lithium fluoride is poorly soluble in MeCN. Within 30 min, diastereomerically pure product 112 was obtained in 81% yield (Scheme 63D).^194^

Treating (5*R*)-menthyloxybutenolide 7f with an excess of diazomethane afforded the cycloadduct 113 as a mixture of two diastereomers in a 60 : 40 ratio (Scheme 64).^196^ Subsequent thermolysis expelled nitrogen as byproduct, affording enantiomerically pure 4-methyl-substituted 114.

**Scheme 64:**
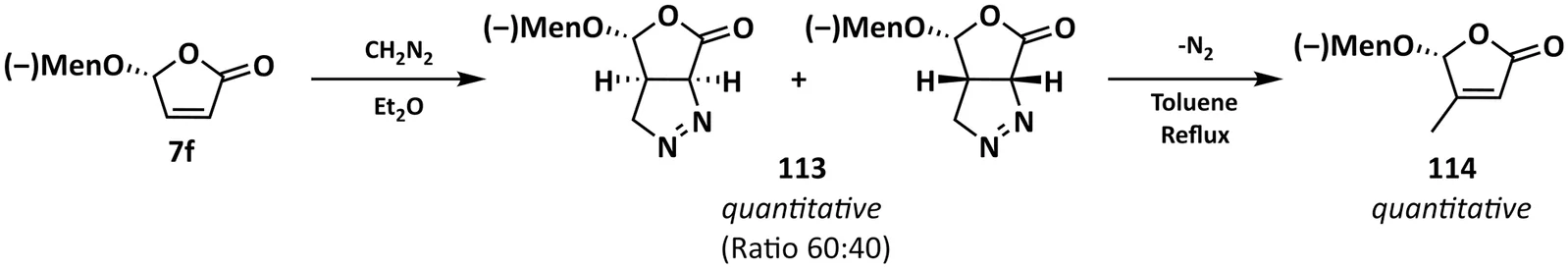
1,3-Dipolar cycloaddition of diazomethane and thermolysis to methyl substituted (5*R*)-menthyloxybutenolide.^196^

##### 3.10.2.3. [2+2]-cycloadditions

Hoffmann *et al.*^208^ (1989) performed acetone-sensitised photochemical [2+2]-cycloadditions on (5*S*)-menthyloxybutenolide 7l with ethylene (Scheme 65).^208^ A diastereomeric mixture of cyclobutane-product 115 was obtained in high chemical yields. Lower temperatures led to improved diastereoselectivity, albeit at the expense of yield. Although the selectivity was moderate, diasteromerically pure cyclobutanes 115 could be obtained *via* simple recrystallization due to the difference in physical properties.^208^ Hoffmann also studied photochemical [2+2]-cycloadditions on (5*R*)-menthyloxybutenolide 7f with cyclic enones.^209^ The diastereoselectivity observed in these reactions indicates an energy transfer from triplet excited cyclopentenone to (5*R*)-menthyloxybutenolide 7f. Triplet excited (5*R*)-menthyloxybutenolide 7f reacts with ground state cyclopentenones yielding cyclobutanes without facial diastereoselectivity. They were able to optimize facial selectivity up to a 9 : 1 ratio by irradiating (5*R*)-menthyloxybutenolide 7f with isophorone, for which energy transfer between triplet excited cyclic enone to (5*R*)-menthyloxybutenolide 7f becomes inefficient. Nonetheless, all isomers could be separated by chromatography.^209^

**Scheme 65:**
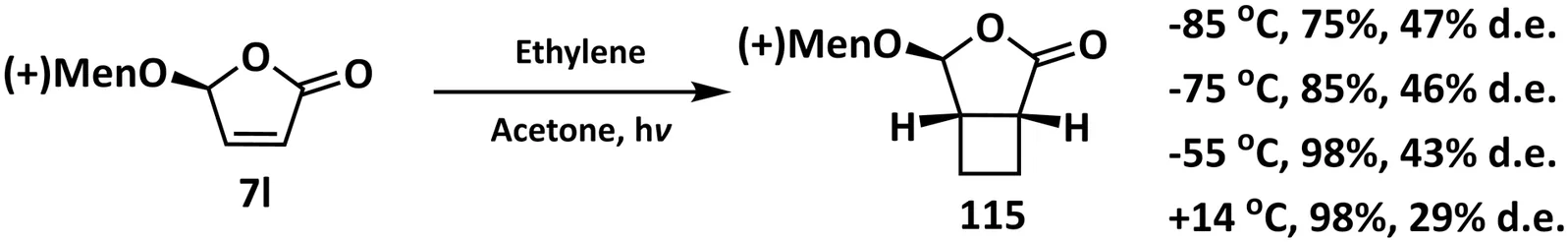
[2+2]-cycloadditions of (5*S*)-menthyloxybutenolide with ethylene.^208^

##### 3.10.2.4. Cyclopropanation

Cyclopropanation of (5*R*)-menthyloxybutenolide 7f was achieved enantioselectivity using sulfur ylides (Scheme 66).^192^ Isopropylidene diphenylsulfurane adds selectively to the less hindered face of (5*R*)-menthyloxybutenolide 7f with excellent diastereoselectivity, affording 116. Subsequent treatment with aqueous perchloric acid resulted in ring-opening to afford the corresponding aldehyde 117, which was used as a precursor in the synthesis of the insecticide deltamethrin 118.^192^

**Scheme 66:**
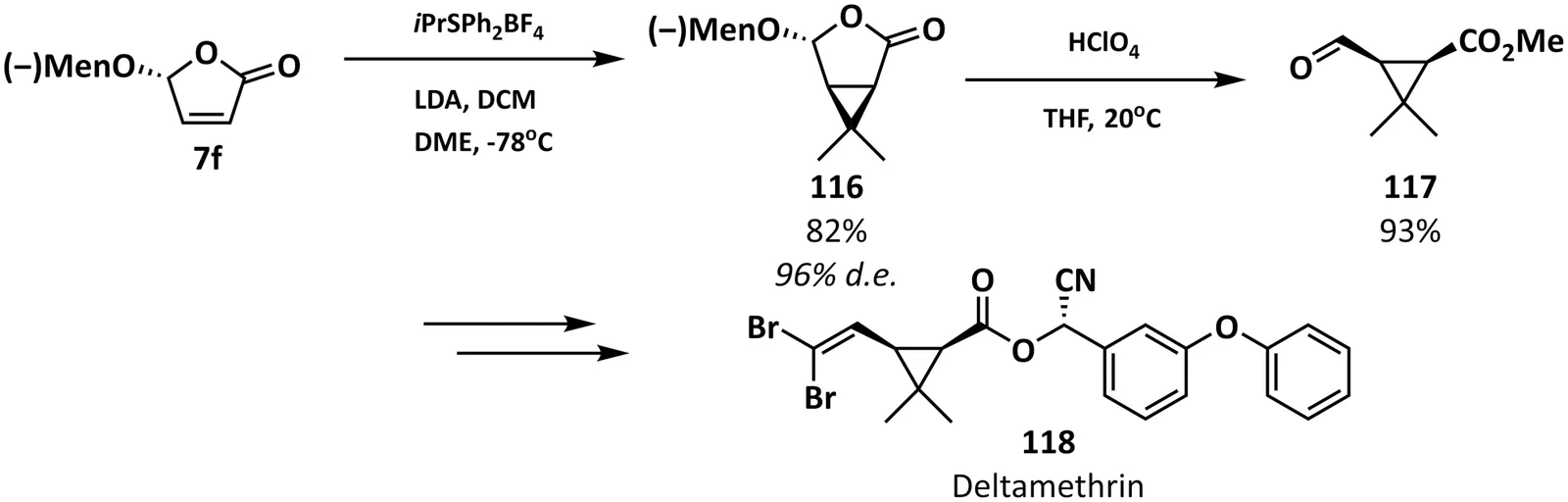
Cyclopropanation of (5*R*)-menthyloxybutenolide and ring-opening of the lactone towards an aldehyde as starting material for insecticide deltamethrin.^192^

#### 3.10.3. Radical reactions

Photochemically generated ketyl and 2-dioxolanyl radicals can also be added to (5*R*)-menthyloxy butenolide 7f with high diastereoselectivity, affording 119 and 120 (Scheme 67).^198^ The radicals add from the face opposite to the menthyloxy substituent, consistent with other 1,4-additions. Irradiations were carried out under nitrogen with a high-pressure mercury lamp fitted with a pyrex immersion well. Acetone was used as sensitizer, and the secondary alcohol served both as reagent and as solvent. Similarly, dioxolanyl radicals could be generated and added when 1,3-dioxolane was used as solvent.^198^

**Scheme 67:**
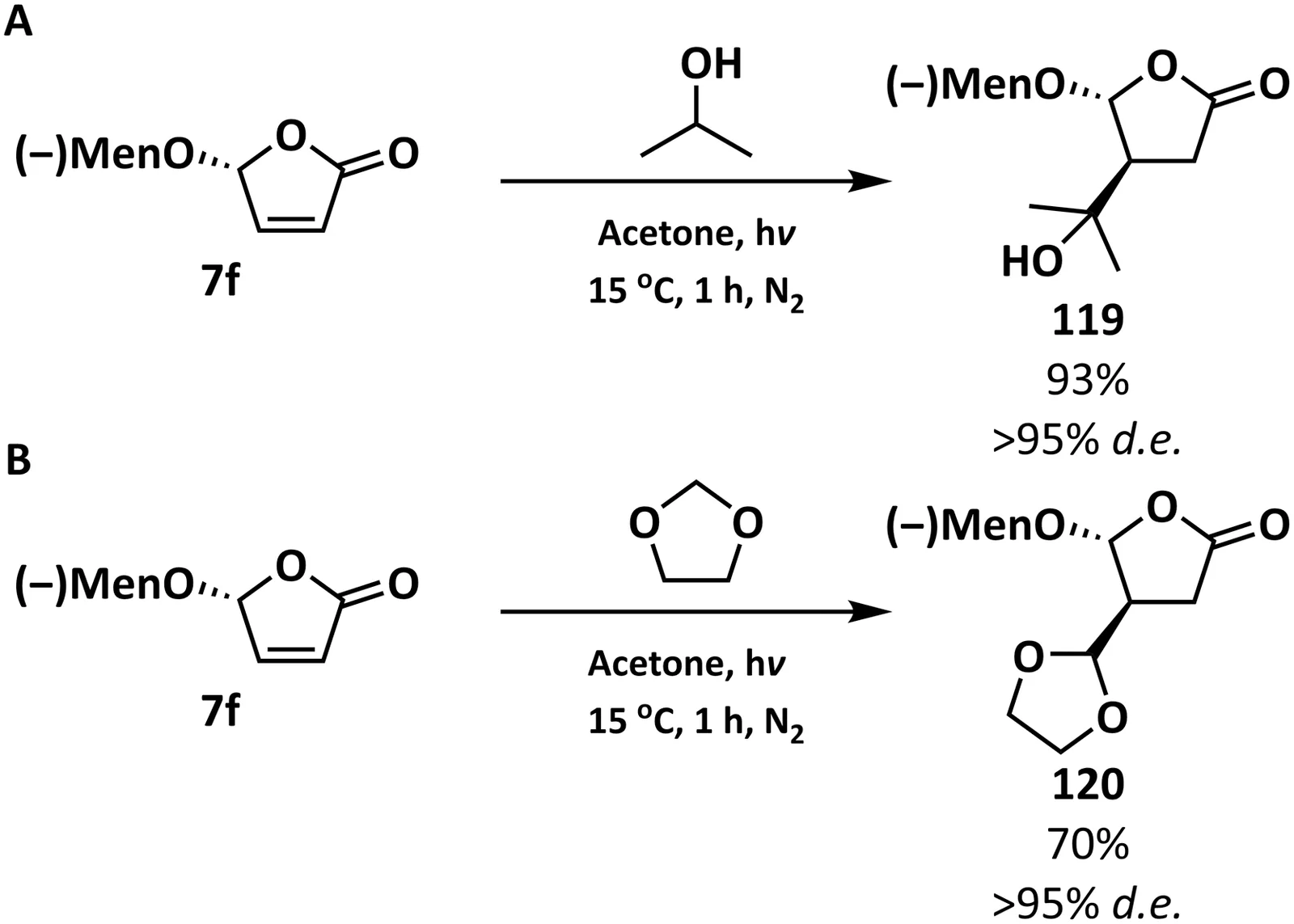
1,4-Addition of photochemically generated radicals, (A) addition of isopropanol, (B) addition of 1,3-dioxolane.^198^

Subsequently, Hoffmann *et al.*^199^ (1999) showed that photochemical addition of tertiary amines derived from pyrrolidine to (5*R*)-menthyloxybutenolide 7f proceeds with complete facial selectivity (Scheme 68). The reaction towards 121 occurs within minutes in MeCN in the presence of 4,4′-dimethoxybenzophenone as photosensitizer.^199^ This concept was extended to tandem radical addition–cyclization reactions of unsaturated tertiary amines (Scheme 69).^201^ In this case, α-aminoalkyl radicals were generated photochemically using Michler's ketone (4,4′-bis-*N*,*N*-dimethyl-aminobenzophenone) as sensitizer, which add diastereoselectively to (5*R*)-menthyloxybutenolide 7f, followed by intramolecular cyclization to 122.^201^

**Scheme 68:**
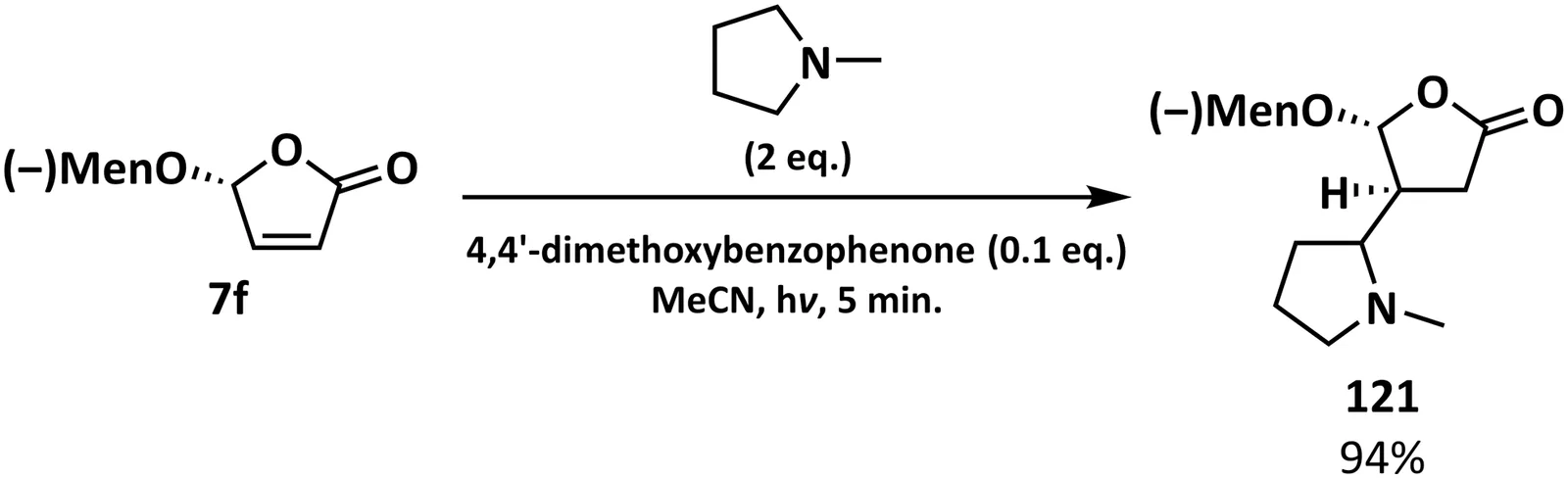
Photochemical addition of tertiary amines from pyrrolidine to (5*R*)-menthyloxybutenolide.^199^

**Scheme 69:**
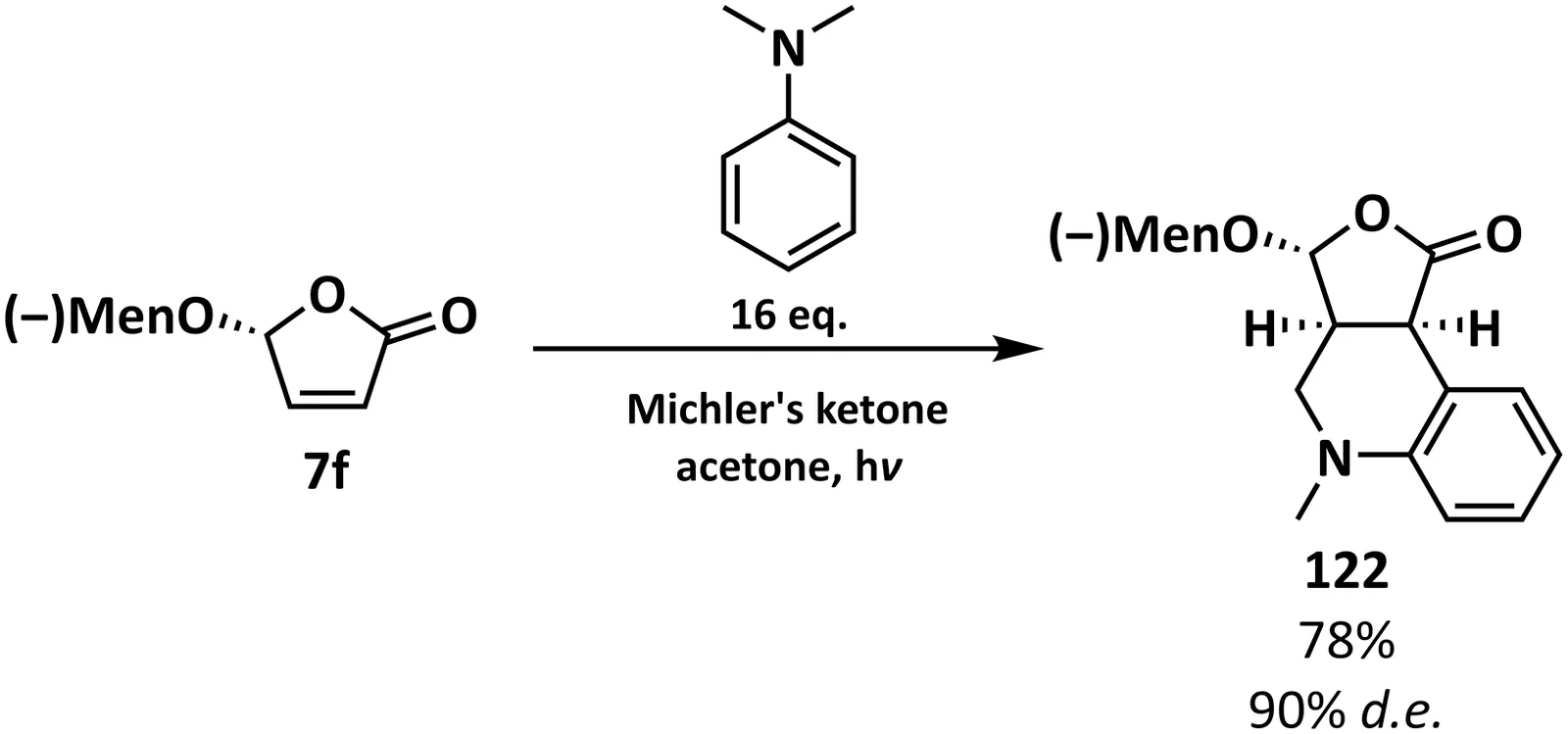
Radical tandem addition–cyclization of tertiary amines.^201^

Interestingly, performing a similar reaction with naphthylamine under these conditions with Michler's ketone resulted in no conversion. However, under heterogeneous conditions using TiO_2_, naphthylamine was added at the 4-position to the α-position of the double bond (Scheme 70). The two diastereomeric adducts 123 and 124 were obtained in a ratio of 1 : 3.3 (53% de).^200^

**Scheme 70:**
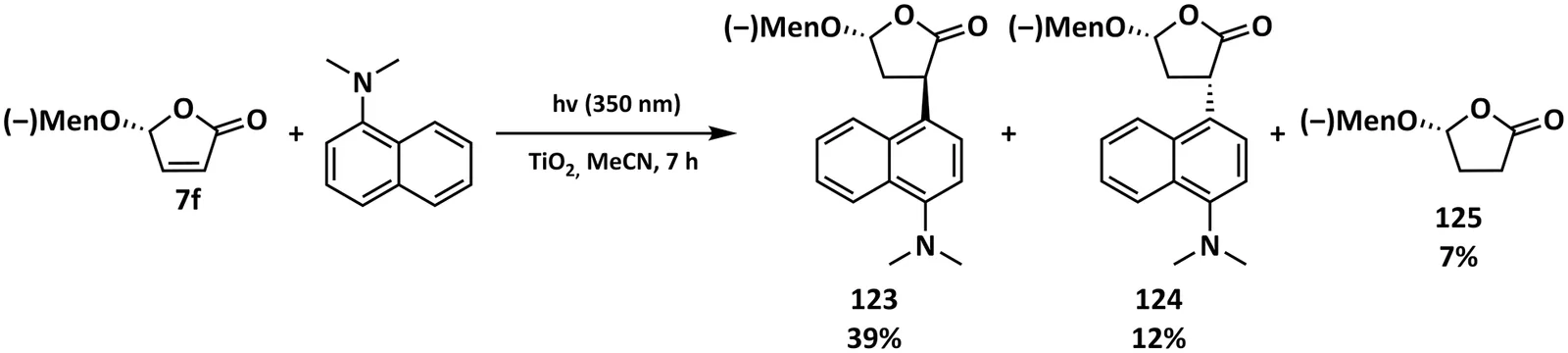
Heterogeneous photochemical addition of naphtylamine to (5*R*)-menthyloxy butenolide.^200^

#### 3.10.4. *cis*-Hydroxylation

Sundermann *et al.*^202^ presented a short and efficient synthesis of chiral cyclopentones from (5*S*)-menthyloxybutenolide 7l (Scheme 71).^202^ The key step in this process was the diastereoselective dihydroxylation of (5*S*)-menthyloxybutenolide 7l to diol 126 using a slight excess of KMnO_4_ (62% yield). Subsequent boron trifluoride-mediated acetalization with cyclohexanone (127, 84% yield), followed by reaction with lithiated dimethyl methylphosphonate, resulted in the formation of the chiral cyclopentone 128*via* sequential ring-opening, loss of menthol, and ring-closure (87% yield).^202^

**Scheme 71:**
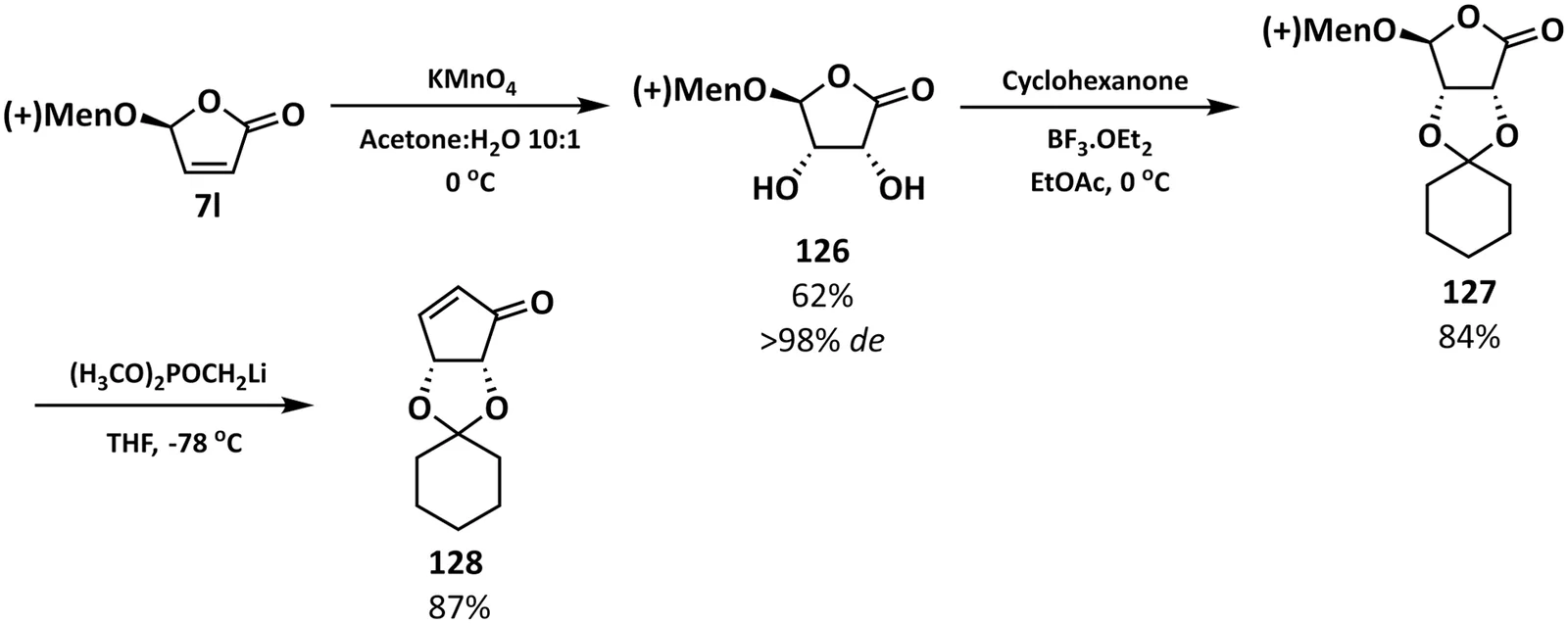
Chiral cyclopentenones from (5*S*)-menthyloxy butenolide *via* diastereoselective dihydroxylation.^202^

#### 3.10.5. Hydrogenation

Hydrogenation of (5*R*)-menthyloxybutenolide 7f proceeds in near-quantitative yield under pressurized conditions in ethyl acetate/triethylamine, affording the corresponding butyrolactone derivative (Scheme 72).^196,210^

**Scheme 72:**
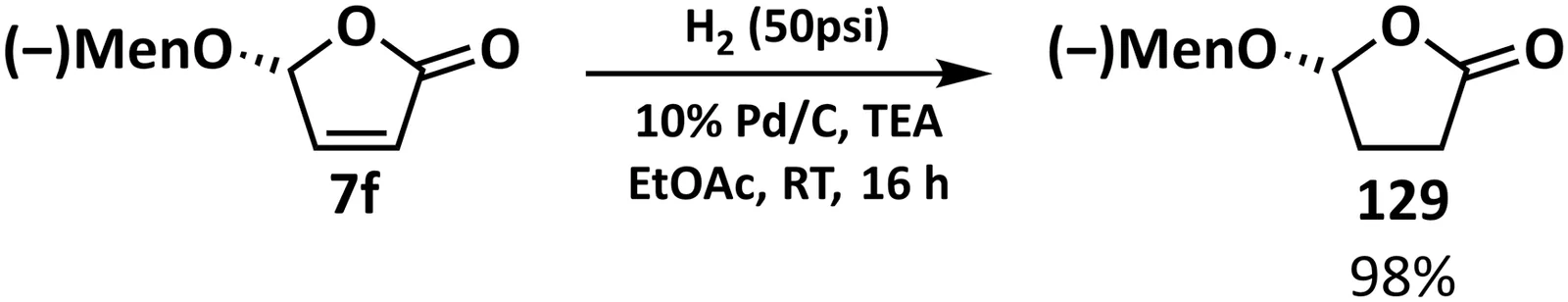
Hydrogenation of (5*R*)-menthyloxybutenolide.^210^

#### 3.10.6. Bromination

As shown by Chavan *et al.*,^146^ bromination of a mixture of 5-menthyloxybutenolides 7f and 7k in benzene at 0 °C, followed by treatment with pyridine, efficiently generates diastereomeric 3-bromo-5-menthyloxybutenolides in 85% yield with a 2 : 3 diastereomeric ratio (Scheme 73).^146^ Pure diastereomer 130 can be isolated in up to 55% by repetitions of low-temperature crystallizations (−18 °C, from *n*-hexane) and epimerization with PTSA.

**Scheme 73:**
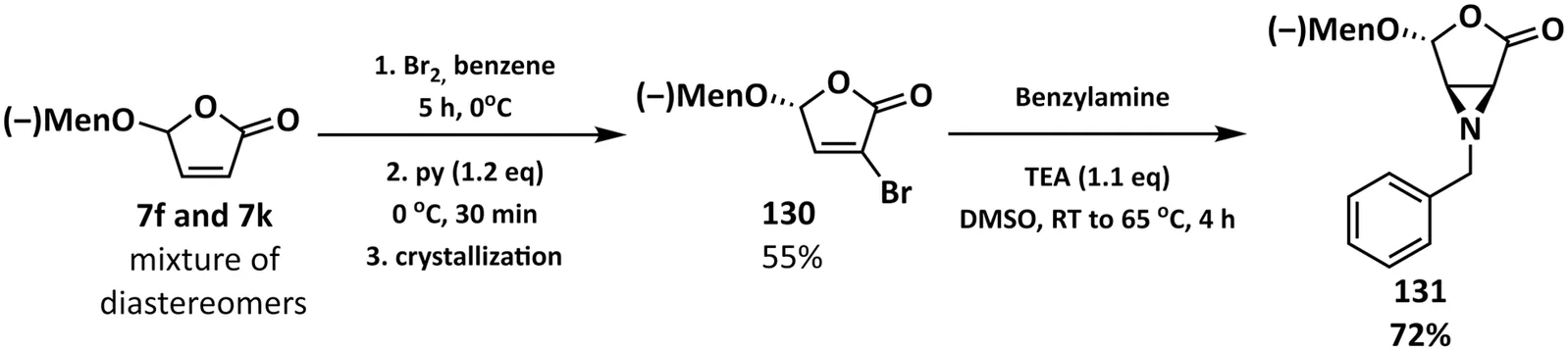
Synthesis of chiral bicycloaziridino lactone from 5-menthyloxybutenolide.^146^

These 3-bromobutenolides serve as versatile intermediates; for instance, their reaction with benzylamine and triethylamine in DMSO affords *cis*-bicycloaziridino lactones 131 (72%), which are chiral, highly stable crystalline synthons with well-defined absolute stereochemistry (Scheme 73).^146^ Such intermediates are particularly valuable for the synthesis of complex natural products.

### 3.11. Dynamic kinetic asymmetric transformations of γ-acyloxybutenolides

Dynamic kinetic asymmetric transformation (DYKAT) is an efficient method for asymmetric synthesis, as up to 100% of a racemic starting material can be converted into a single enantiomeric product.^211^ The key difference between kinetic asymmetric transformation (KAT) and DYKAT comes down to whether the substrate can interconvert between stereoisomers during the reaction. This results in a maximum theoretical yield of 50% for KAT because the chiral catalyst reacts with one enantiomer while the other remains unreacted. With DYKAT quantitative yields of a single enantiomeric product can be achieved through reaction of the chiral catalyst with one enantiomer, while the other enantiomer continuously racemizes. In this context Trost *et al.*^98^ (1999) investigated asymmetric allylic substitution reactions on racemic γ-acyloxybutenolides (5-acyloxybutenolides) bearing good leaving groups in the γ-position.^98^

In their work, they utilised chiral Pd(0) complexes that coordinate to γ-acyloxybutenolide to form two different η^2^-olefin complexes (Scheme 74). Ionization of the γ-acyloxybutenolides generates two η^3^-allyl palladium complexes, which can interconvert through the intermediacy of a palladium furanoate species. If this interconversion is faster than the nucleophilic attack, and if one of the diastereomeric η^3^-complexes reacts faster than the other, an effective palladium-catalysed DYKAT is achieved.^73,98^

**Scheme 74:**
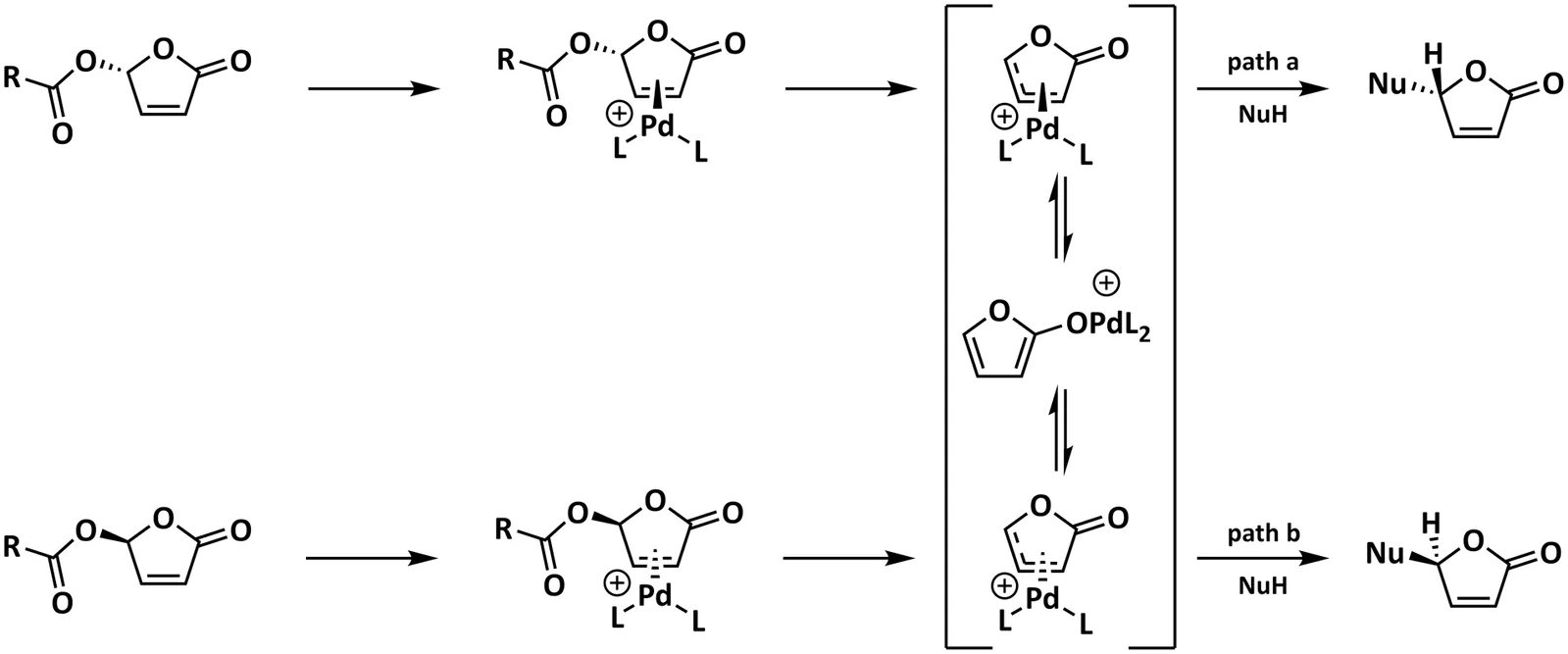
Concept for dynamic kinetic asymmetric transformation (DYKAT) of γ-acyloxybutenolides; the enantiomer produced from this reaction depends on the chirality of catalyst/ligand.^73,98^

The reaction of racemic Boc-protected butenolide 35a with phenolic nucleophiles in the presence of Pd(0) complexes and chiral ligands can be performed under conditions that favour either a KAT or DYKAT. Performing the reaction at high concentrations (0.5 M) in the presence of carbonate base favours the KAT pathway, whereas conducting the reaction at 0.1 M in the presence of tetra-*n*-butylammonium chloride promotes the DYKAT process. Using conditions favouring the DYKAT process, Trost *et al.*^162^ showed enantioselective allylic substitution of 2-naphthol onto Boc-butenolide 35a, affording the S-enantiomeric product 132 in 87% and 97% ee (Scheme 75).^162^ This approach provides access to either enantiomer in high enantiomeric excess simply by switching the ligand chirality, and yields a derivative suitable for subsequent C–C bond forming events like cycloaddition and Michael reactions. These transformations exhibited stereoselective addition from the π-face opposite to the phenolic substituent, analogous to reactions on 5-menthyloxybutenolides. Using these intermediates, biologically active targets like (−)-aflatoxin, BAY 36–7620, and (+)-Brefeldin A were synthesized (Scheme 75).^162,212,213^

**Scheme 75:**
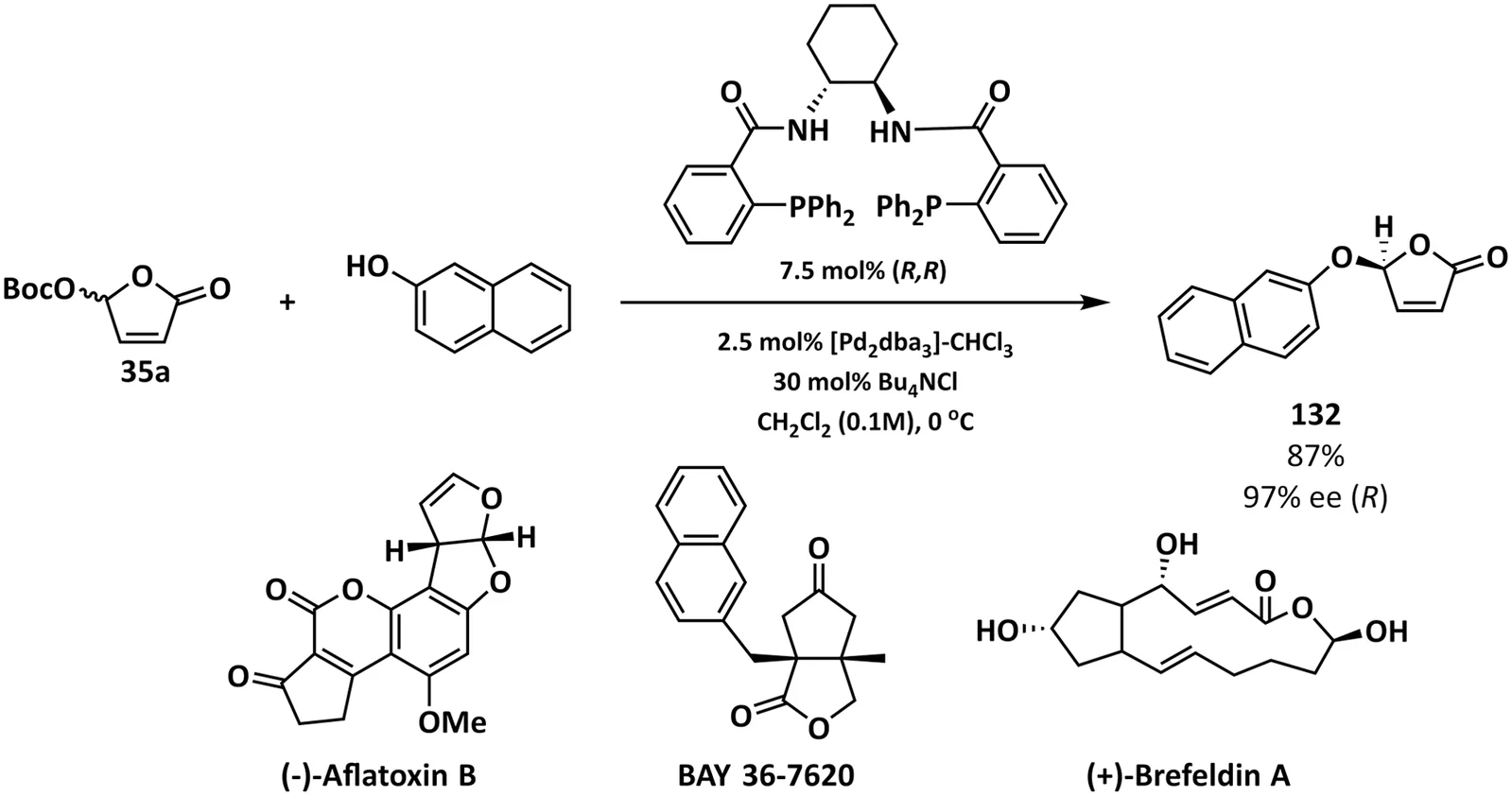
Enantioselective allylic substitution of Boc-butenolide with 2-naphthol nucleophile *via* DYKAT.^162^

Ma *et al.*^214^ utilised this palladium-catalysed strategy for asymmetric allylic alkylation of carboxylic acids with racemic Boc-protected butenolide 35a (Scheme 76A). They demonstrated a broad substrate scope and excellent stereocontrol using Trost's chiral diphosphines, achieving up to 97% ee and >20 : 1 dr, while tolerating diverse carboxylic acids, including natural products, pharmaceuticals and peptides (Scheme 76B).^214^ They further reported Michael addition of cysteine-containing peptides to chiral butenolide esters in aqueous buffer at room temperature (Scheme 76C). These chiral butenolide esters functioned as prodrug scaffolds and peptide-drug conjugation units, providing a new strategy for the construction of stereodefined bioconjugates and chiral prodrugs.^214^

**Scheme 76:**
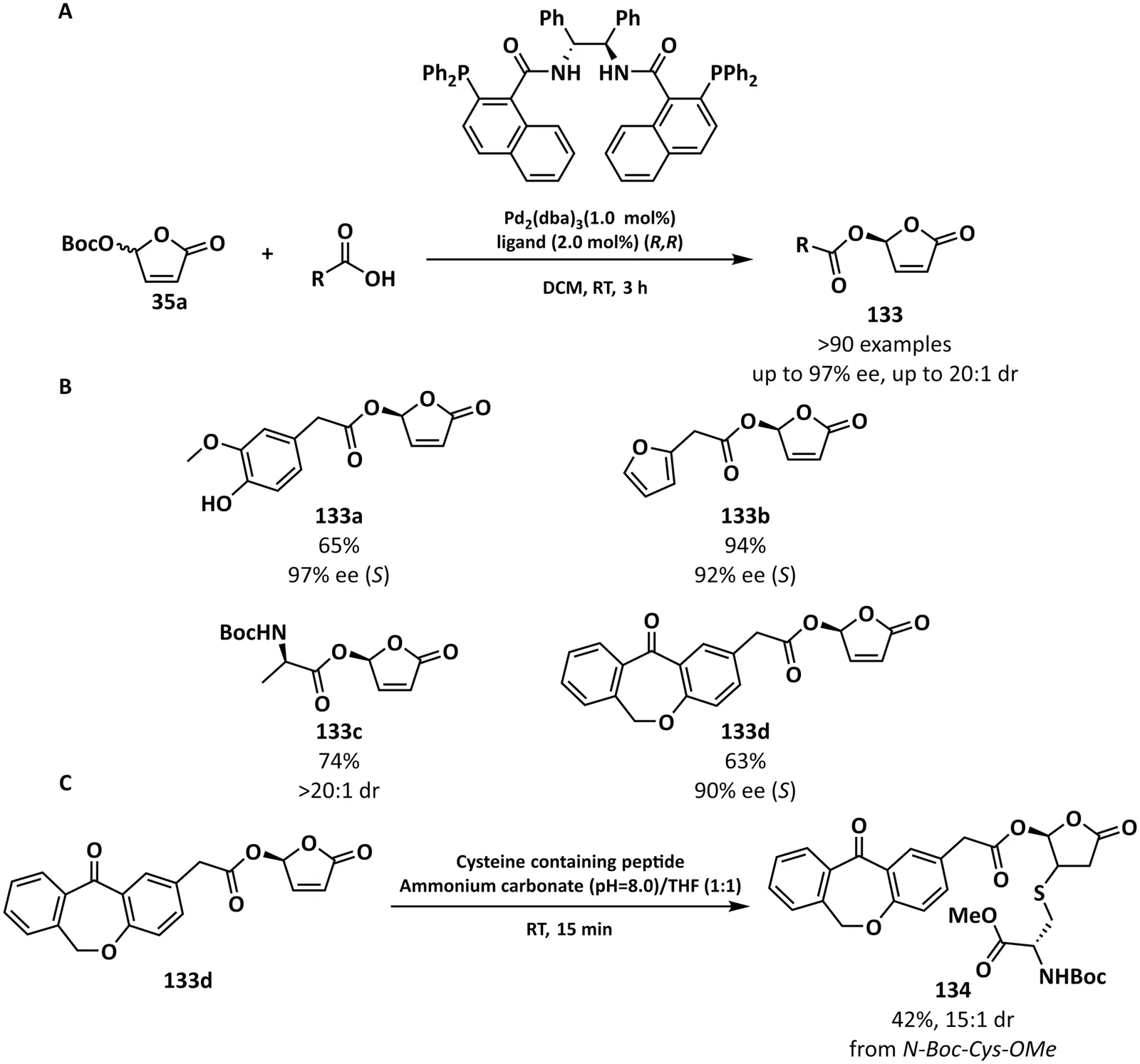
Palladium-catalyzed asymmetric allylic alkylation of carboxylic acids to Boc-butenolide, (A) general scheme, (B) selected examples of products, (C) selected example for coupling cysteine containing peptides to butenolides *via* Michael addition.^214^

### 3.12. Organocatalyzed [3+2] asymmetric cycloaddition for kinetic resolution

Valle-Amores *et al.*^215^ presented an enantioselective organocatalysed [3+2] cycloaddition of racemic butenolide derivatives with azomethine ylides (Scheme 77).^215^ This transformation enables the formation of bicyclic adducts bearing up to five contiguous stereogenic centres, proceeding *via* a stereocontrolled endo approach with high enantioselectivity. In addition, the reaction is accompanied by a highly efficient kinetic resolution, since the use of Takemoto's catalyst only promotes the [3+2] cycloaddition with the S-enantiomer of 5-methoxybutenolide 7d to products 135. In this system, hydrogen bonding between the catalyst donor units and both reactants play a crucial role, promoting facial discrimination and thereby enabling highly selective kinetic resolution.^215^

**Scheme 77:**
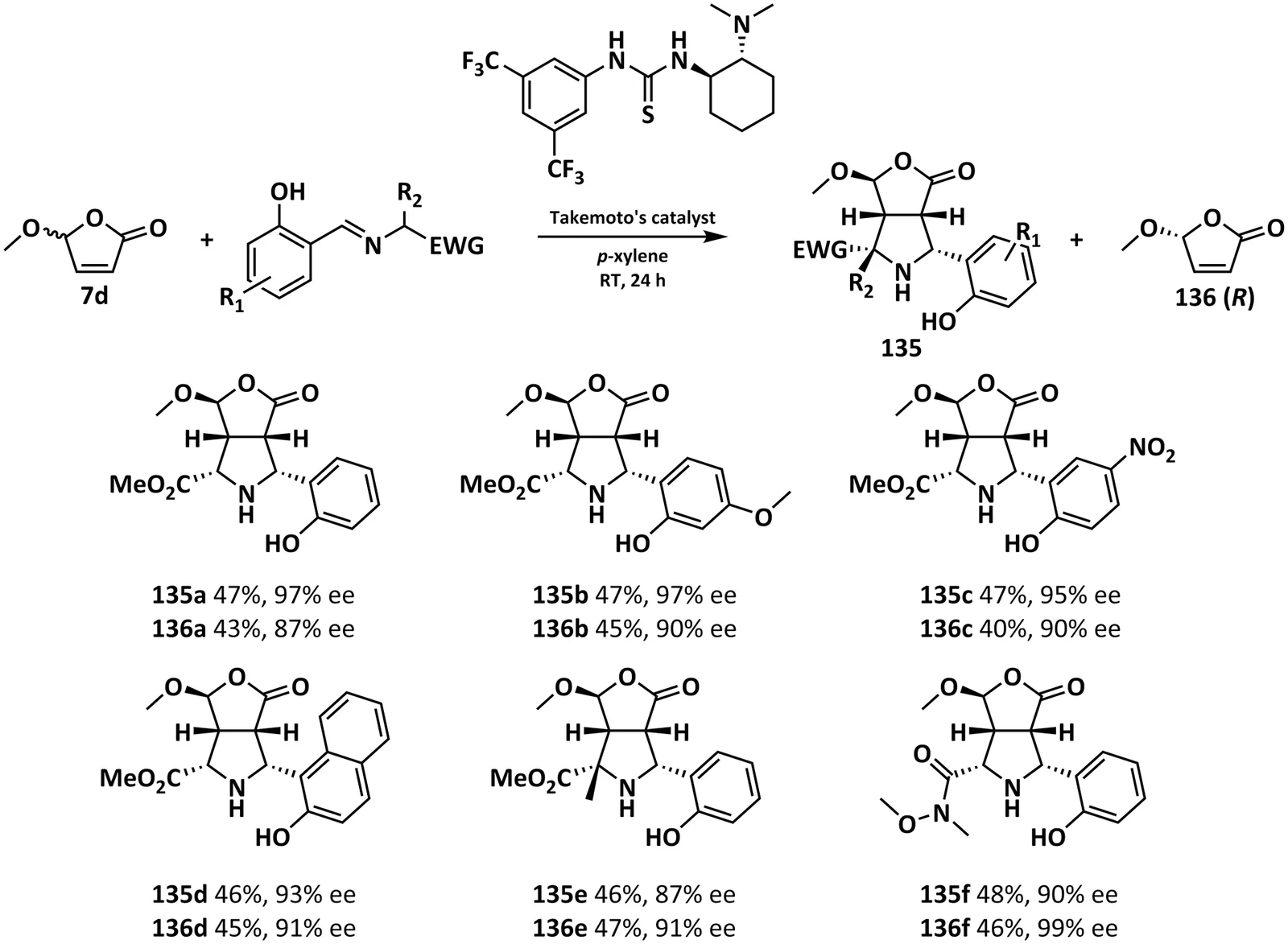
Organocatalyzed [3+2] cycloaddition between racemic methoxy butenolide and azomethine ylide.^215^

## 4. Application as bio-based building block

As discussed in the previous sections, 5-hydroxy-2(5*H*)-furanone (HFO, 5-hydroxybutenolide) demonstrates exceptional reactivity, primarily attributed to its unique structural composition. The molecule features the presence of a lactone ring, a hydroxyl group, a chiral center, and an α,β-unsaturated carbonyl moiety, all of which synergistically contribute to its diverse chemical behaviour. These functionalities not only enable a broad range of chemical transformations but also enhance its value as a key intermediate in synthetic chemistry. Building on these characteristics, this section highlights the pivotal role of HFO as a bio-based, sustainable platform chemical. Its applicability spans multiple fields, where it serves as a fundamental building block for the synthesis of solvents, fine chemicals, and pharmaceuticals, underscoring its significance in both academic research and industrial applications.

### 4.1. As a platform towards C4 building blocks

HFO serves as a valuable precursor in the synthesis of diverse C4 chemical building blocks (Fig. 14). Through oxidation, it can be converted into maleic anhydride (MAnh) and its corresponding acid, which find extensive utility as raw materials in polymer applications, agricultural chemicals, pharmaceuticals, and other sectors.^1,2^ Conventionally, MAnh and maleic acid (MA) are produced by vapor-phase oxidation of petroleum-derived *n*-butane at elevated temperatures.^216,217^ As previously shown (Section 3.1), the oxidation of HFO into MAnh and MA can be performed using milder conditions (*i.e.*, lower temperatures and molecular oxygen as the oxidant).^151,152^ Hydrogenation of HFO leads to the formation of key C4 chemicals such as 1,4-butanediol (BDO), tetrahydrofuran (THF), and γ-butyrolactone (GBL), which are widely used as raw materials for polymer production, solvents, pharmaceuticals, cleaning agents, and related applications.^218,219^ These compounds are typically produced from acetylene and formaldehyde following the Reppe process, or from MAnh (derived from petrochemical *n*-butane) using the Davy process.^219–222^ In contrast, catalytic hydrogenation of HFO enables the production of BDO, THF, and GBL under comparatively mild conditions, thereby demonstrating its effectiveness as a sustainable platform chemical.^99^ Additionally, isomerization of HFO leads to the production of succinic anhydride, which is readily hydrolysed to succinic acid.^223,224^ Both compounds have emerged as key bio-based building blocks,^225,226^ due to their versatility and are widely utilised in the food and polymer industries.^224^ Conventionally, succinic anhydride is produced from MAnh (derived from *n*-butane) *via* hydrogenation.^227^

**Fig. 14:**
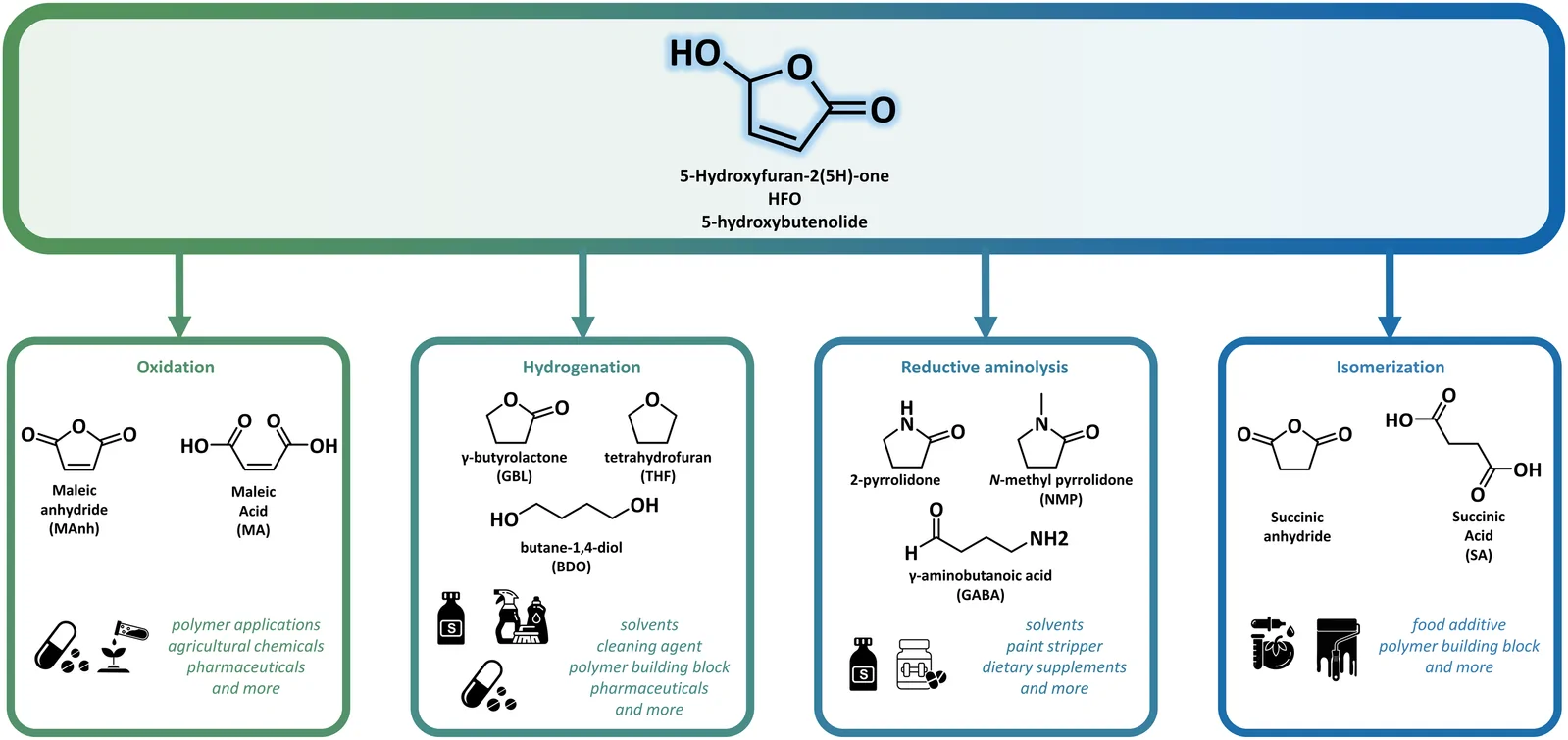
Overview of possible routes toward other C4 chemical building blocks *via* isomerization,^96,228^ oxidation,^151,152^ hydrogenation^99^ and reductive aminolysis.^99^

In contrast, isomerization of HFO can be achieved under mild conditions, yielding succinic anhydride^228^ (*via* photochemical pathways) and succinic acid^96^ (in basic media). Reductive aminolysis of HFO also enables the synthesis of nitrogen-containing C4 compounds such as 2-pyrrolidone, *N*-methyl pyrrolidone (NMP) and γ-aminobutanoic acid (GABA).^99^ These compounds are widely used as solvents, paint strippers, dietary supplements, and in other applications.^229,230^ They are conventionally produced from GBL at high temperatures and pressures over solid magnesium silicate catalysts.^231^ In contrast, HFO can be converted into these nitrogen-containing compounds *via* hydrogenation followed by amination under comparatively milder conditions. This further underscores HFO as a sustainable scaffold for the production of value-added C4 chemicals in line with the 12 Principles of Green Chemistry.

### 4.2. Fine chemicals and pharmaceuticals

While HFO is a key platform chemical to small C4 chemical building blocks, it can also be used as a platform toward a wide range of natural products as the butenolide moiety represents a structural motif present in a wide range of biologically active molecules. This motif has been identified in numerous plant species and marine organisms.^232–234^ Consequently, significant synthetic effort has been devoted, and continues to be directed, toward the total and partial synthesis of butenolide-containing natural products.^235–241^ Their diverse biological activities include, but are not limited to, bactericidal, fungicidal, antibiotic, and anticancer properties.^61^ For a comprehensive overview of butenolide-containing natural products, the reader is referred to the relevant review articles cited in the following.^59,61,73,87,88,242^ In the next sections, we aim to highlight the use of HFO as a versatile synthetic scaffold for the preparation of selected natural products.

#### 4.2.1. HFO in fine chemicals and pharmaceuticals

One of the earliest reported butenolide-based pharmaceuticals was in 1971 by DeGraw, starting from 5-ethoxy-2(5*H*)-furanone toward pilocarpine (Scheme 78).^243^*Michael* addition of diethyl ethylmalone, followed by treatment with hot hydrogen bromide-acetic acid afforded 2-ethyl-3-carboxymethylbutenolide, a key intermediate in the synthesis of pilocarpine. Typically, this lactone alkaloid is extracted from plants of the *Pilocarpus genus*,^244^ and is used to treat angle closure glaucoma.^245^ In 2021, Schmidt *et al.*^246^ also reported the concise synthesis of both enantiomers of pilocarpine utilising HFO as platform chemical. Esterification of HFO, followed by hydrogenation, enzymatic hydrolysis, reduction of *Weinreb amides* and the single-step attachment of 1-methyl-imidazole allows the synthesis of both enantiomers.^246^

**Scheme 78:**
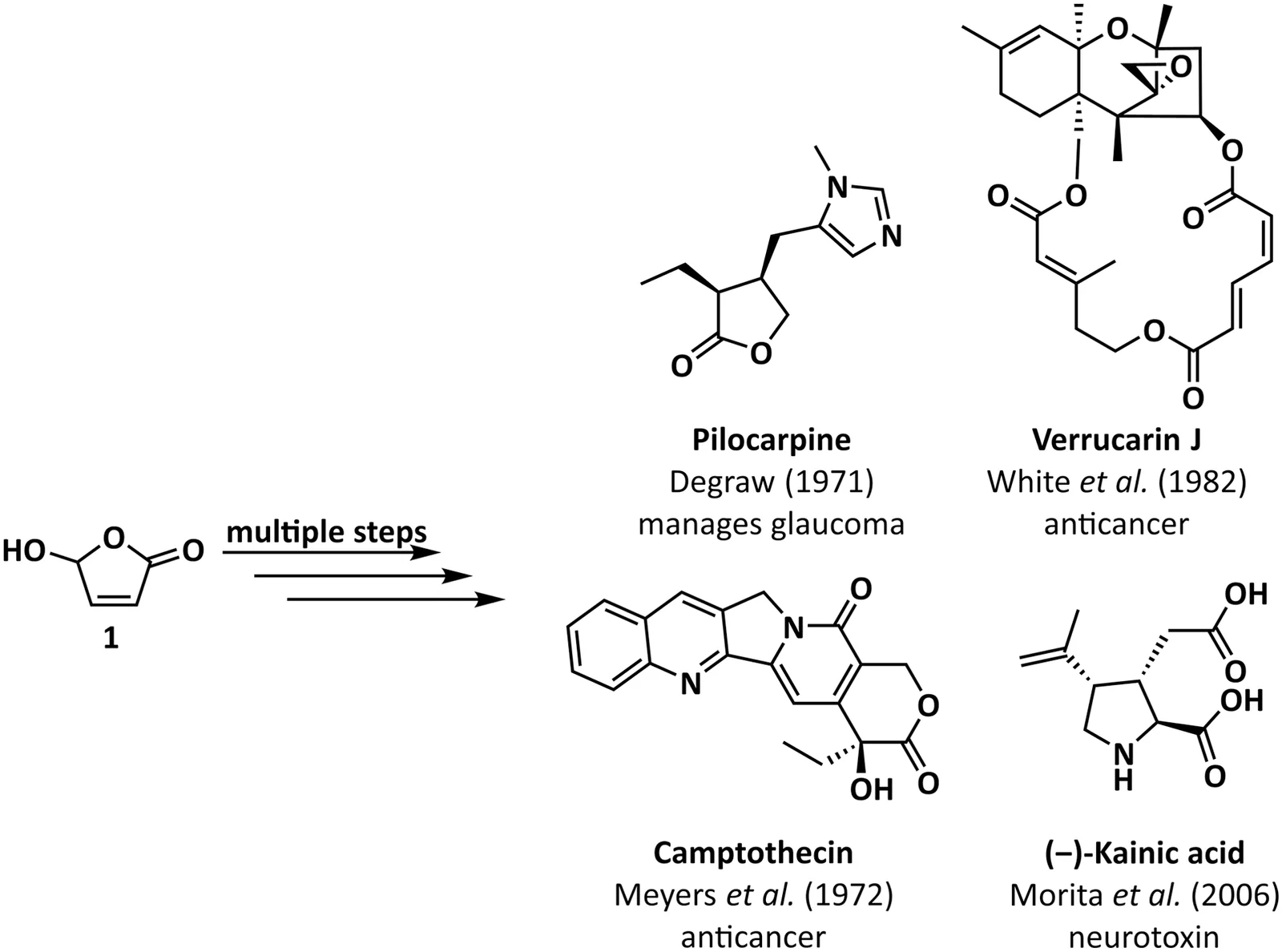
Overview of a selection of natural products synthesized using HFO as a platform chemical.^111,163,243,246,247^

Another example is the total synthesis of the plant antitumour agent C*amptothecin* utilising HFO (Scheme 78) reported Meyers *et al.*^168^ in 1973.^168^ After acetalization into 5-ethoxybutenolide, hydrolysis led to a glyoxal diethyl acetal. This compound then underwent a Wittig coupling reaction to form the key intermediate unsaturated ester containing a potential or masked aldehyde functionality.^168^*Camptothecin*, a pentacyclic alkaloid, was first isolated from stem wood of *Camptotheca acuminata* and has been explored in preliminary trials for anticancer treatment.^248^

Later, in 1982, White *et al.*^111^ utilised HFO as a substrate for a Horner–Wadsworth–Emmons reaction with a phosphonate to afford the macrocycle segment of *Verrucarin J* (Scheme 78).^111^ This step was performed with good yields of 80–84%. The natural product *Verrucarin J* is known to possess antibiotic, antifungal and antileukemic activity.^249^ Unfortunately, they have proven to be too toxic for chemotherapy.^250^

In 2005, Morita *et al.*^163^ reported the stereocontrolled total synthesis of (−)-kainic acid utilising HFO (Scheme 78).^163^ Here, lipase AK was used in combination with vinyl acetate to introduce a chiral acetoxybutenolide followed by an 1,3-dipolar cycloaddition to form a key intermediate for the synthesis of kainic acid. This compound is used for studying neurotoxicity and neurodegenerative pathways.^251^

#### 4.2.2. Menthyloxy-2(5*H*)-furanone in fine chemicals and pharmaceuticals

Menthyloxy-2(5*H*)-furanone, *i.e.* 5-menthyloxybutenolide, is a key chiral building block due to its versatile reactivity, high diastereoselectivity, crystallinity and synthetic scalability.^49–56,87^ Acetalization of HFO with (−)- or (+)-menthol produces a diastereomeric mixture, and epimerization under acidic conditions allows conversion to the desired diastereomer. Since our first synthesis in 1988 5-menthyloxybutenolide has been a platform toward many complex natural products.^49^ Here, we highlight a few of these synthetic procedures utilising this chiral auxiliary (Scheme 79).

**Scheme 79:**
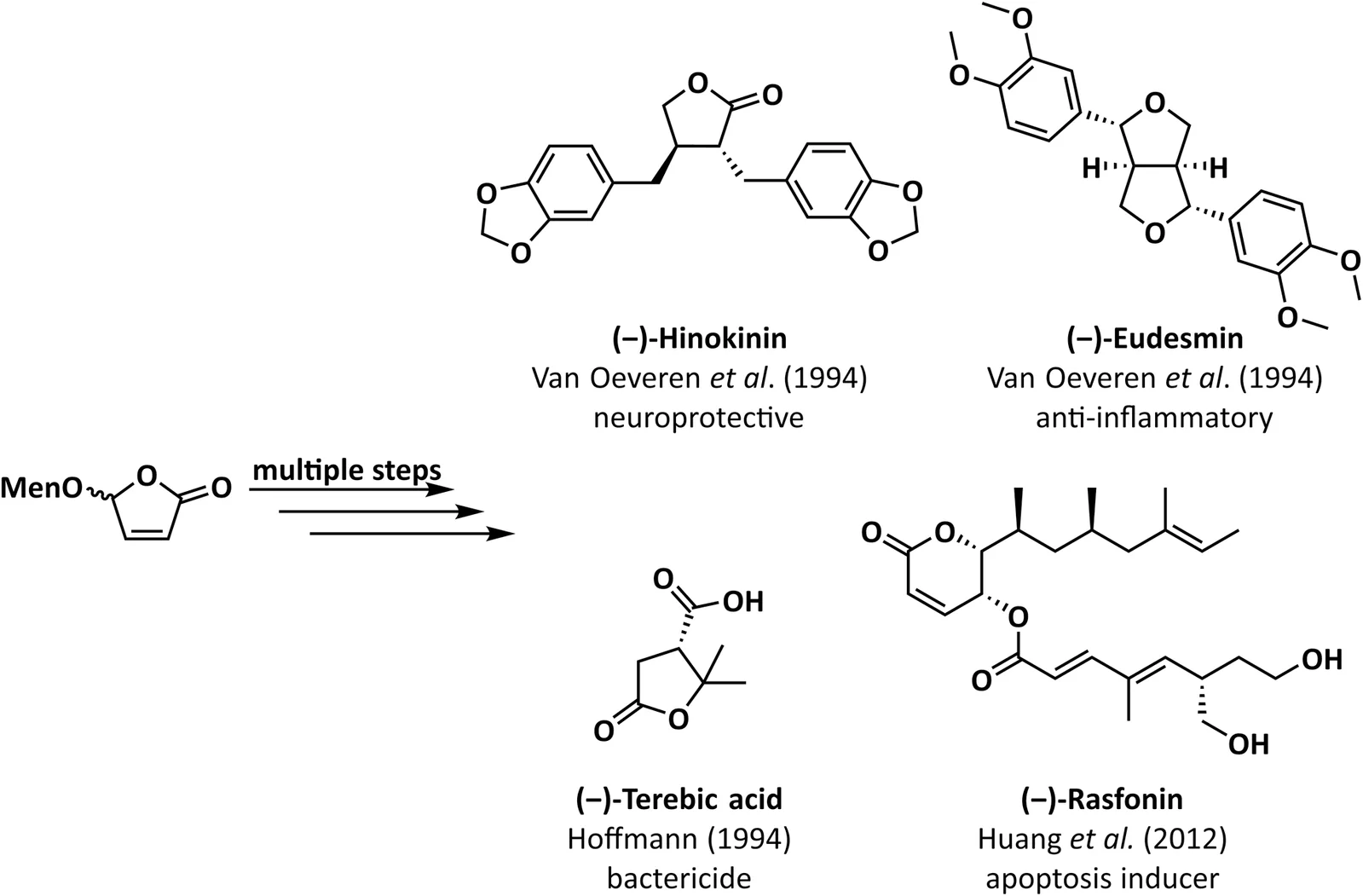
Overview of a selection of bioactive products synthesized from 5-menthyloxy-2(5*H*)-furanone (5-menthyloxybutenolide).^53,191,203,204,252,253^

In the 1990s, Feringa and coworkers used 5-menthyloxy-2(5*H*)-furanones as chiral synthons to synthesise novel lignans.^53,191,203,204^ The basic lignan carbon framework, *i.e.*, the dibenzylbutyrolactone structure, was synthesised *via* a tandem conjugate addition-alkylation of enantiomerically pure menthyloxybutenolide (yields typically 50–70%). This methodology was used for a series of natural products, including (–)-hinokinin (total yield 37%) and (–)-eudesmin (total yield 16%). While both compounds show anti-inflammatory and anti-tumour properties, they also possess neuroprotective,^254,255^ and antimicrobial effects, respectively.^256^

In 1994, using (5*R*)-menthyloxy-2(5*H*)-furanone as key synthon, Hoffmann reported the synthesis of racemic and enantiomerically enriched derivatives of (−)-terebic acid (Scheme 79).^252^ This compound and its derivatives belong to the γ-lactone family and have been shown to have, among others, antibiotic and antitumour properties.^257^

5-Menthyloxybutenolide was also reported as a key substrate for the synthesis of (−)-rasfonin as reported by Huang *et al.*^253^ in 2012. Here, they utilize the butenolide to synthesize the lower half of the natural product. The stereogenic centre in the lower half of the molecule was introduced by Michael addition of lithium bis(phenylthiol)methane to 5-menthyloxybutenolide and provides the *trans* product as the single diastereoisomer in 86% yield. This specific reaction acts as a key step for the synthesis of the final natural product (−)-rasfonin. (−)-Rasfonin, which can be isolated from fungi or fermented mycelium, has been reported as an active apoptosis inducer in *ras*-dependent cells.^258^

#### 4.2.3. 5-Boc-protected butenolides in fine chemicals and pharmaceuticals

*tert*-Butyl carbonoxybutenolide (or 5-Boc-protected butenolide) offers a racemic starting material for asymmetric allylic alkylations with phenol nucleophiles. This process, utilising a palladium catalyst alongside the chiral Trost ligand, can be performed under conditions that favour a kinetic resolution or a kinetic asymmetric transformation (KAT), or dynamic asymmetric transformation (DYKAT).^259^

Trost and Toste explored the use of palladium-catalysed asymmetric allylic alkylation to DYKAT of racemic 5-Boc-protected butenolide for the synthesis of Aflatoxin B_1_ (1999 and 2003) (Scheme 80).^98,162^ Aflatoxins are among the most potent naturally occurring carcinogens known, and are produced from fungi that contaminate staple crops like corn, rice and wheat.^260,261^ Synthesizing these compounds provides essential analytical standards for food safety testing to support toxicology and carcinogenicity mechanistic studies. Here, 5-Boc-protected butenolides enable efficient and stereoselective synthesis of these aflatoxins. In subsequent work by Trost and Crawley, (+)-*brefeldin* A was synthesized using DYKAT with 5-Boc-protected butenolide in 2002 (Scheme 80).^212,213^ Using a palladium catalyst with *Trost ligands*, they synthesized 5-naphthoxybutenolide *via* 5-Boc-protected butenolide. Then, the butenolide cyclic moiety underwent enantioselective cycloaddition utilising a trimethylenemethane-palladium complex to yield a key precursor for the synthesis of (+)-brefeldin A. This compound was initially tested for its antiviral properties but is now primarily utilised in research to study protein transport.^262^

**Scheme 80:**
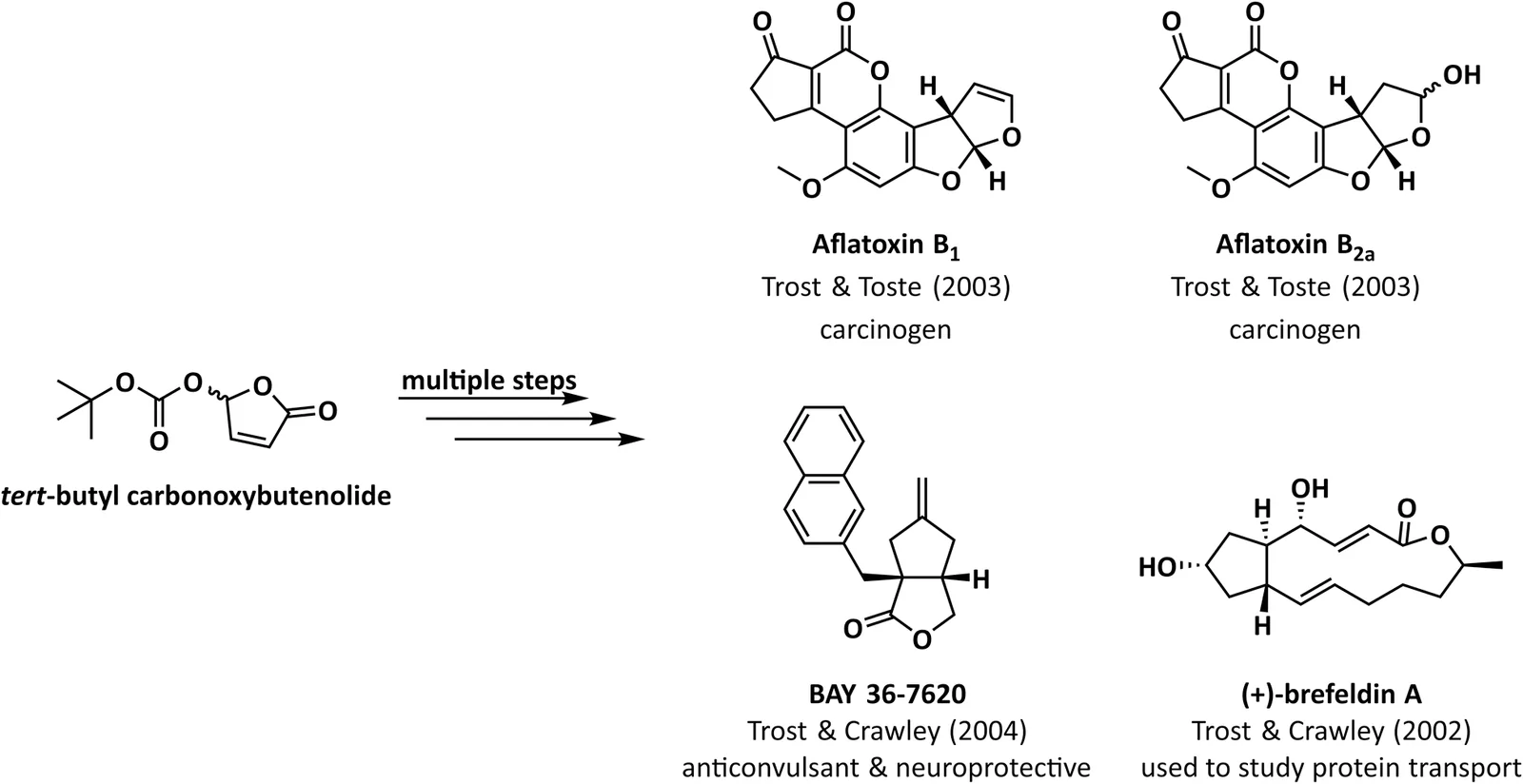
Overview of a selection of bioactive products synthesized from *tert*-butyl carbonoxybutenolide.^98,162,212,213^

In addition, BAY 36-7620 was synthesized using similar methodology in 2004 (Scheme 80).^213^ Here, 5-naphthoxybutenolide (originating from 5-Boc-protected butenolide) underwent an enantioselective cycloaddition with trimethylenemethane to form a key intermediate for the synthesis of the *Bayer* chemical, BAY 36-7620. This compound is known for its anticonvulsant and neuroprotective properties.^263^

In this section, we have highlighted a selection of synthetic pathways to bioactive products utilising HFO as key platform chemical. HFO offers versatility in natural product synthesis due to its unique structure and chirality, with multiple functionalities. Furthermore, 5-menthyloxybutenolide and 5-Boc-protected butenolide offer direct and efficient stereoselective synthesis of key natural products.

### 4.3. Surfactants

Surfactants play a crucial role in the global chemical industry due to their unique characteristics, which enable them to reduce surface tension and stabilize emulsions.^264^ These amphiphilic compounds consist of both hydrophobic and hydrophilic components, enabling them to effectively modify surface and interfacial properties.^265^ Synthetic surfactants, predominantly derived from petrochemical feedstocks, are widely used in detergents,^266^ cosmetics,^267^ agrochemicals,^268,269^ pharmaceuticals,^270^ and coatings.^271,272^ Despite their widespread utility, they pose several risks to both human health and the environment.^273^ Many conventional surfactants exhibit low biodegradability, leading to their accumulation in aquatic environments and subsequent harm to aquatic life. In addition, the reliance on finite fossil resources for their production further underscores the need for more sustainable alternatives. Consequently, there is growing interest in the development of biodegradable surfactants from renewable sources.

In this context, bio-based surfactants,^274,275^ including those derived from furfural,^157,276^ are particularly promising as they often demonstrate improved biodegradability compared to their conventional counterparts.

In a study by Gassama *et al.*,^158^ a novel synthetic route toward bio-based anionic surfactants was developed, employing furfural as the starting material and HFO as a key intermediate (Scheme 81).^158^ The synthetic pathway involves the photo-oxidation of furfural to yield HFO, which is subsequently subjected to acetalization with fatty alcohols. This transformation introduces hydrophobic alkyl chains, a structural feature essential for surfactant properties. The resulting intermediates are then further functionalized *via* the addition of sodium bisulfite (NaHSO_3_) to the α,β-unsaturated carbonyl moiety of HFO, affording two distinct sulfonated surfactant structures: one arising from sulfonate addition across the C=C double bond (45–65% yield over two steps), and the other from sulfonate addition at the aldehyde functionality of the open-chain form (31–37% yield over two steps).

**Scheme 81:**
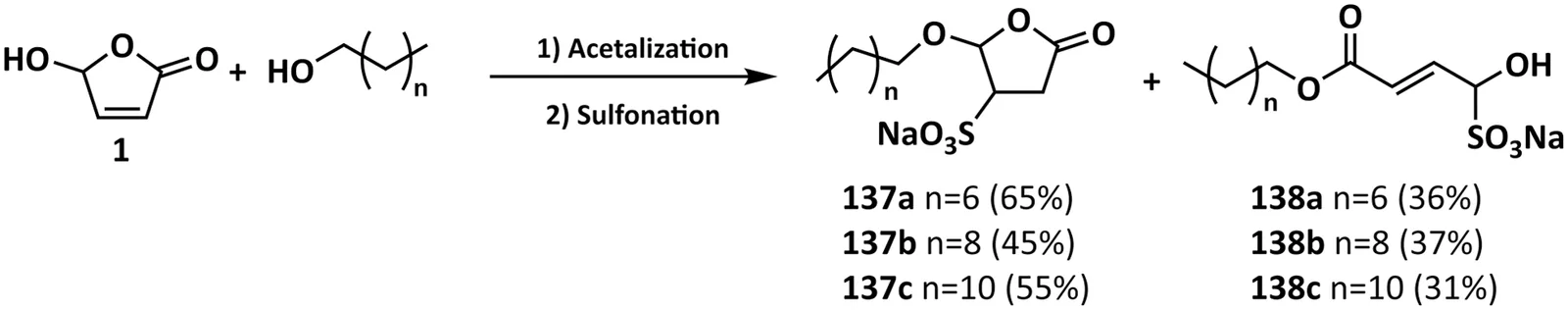
Synthesis of bio-based surfactants from HFO, using acetalization and sulfonation, as reported by Gassama *et al.* in 2013.^158^

The bio-based surfactants obtained through this approach (137a–c) exhibit detergency performance comparable to that of conventional petroleum-derived analogues, while the linear sulfonated species (138a–c) exhibit inferior surfactant properties (Table 2). By altering the hydrophobic chain length, key physiochemical properties such as the critical micelle concentration (CMC) and Krafft point can be tuned. Finally, biodegradability studies revealed significant degradation for compounds 137a–c (67–78% after 28 d), while the linear sulfonated species (138a–c) showed poorer biodegradability (26–50% after 28 d). These findings highlight the potential of HFO as a versatile platform for the development of sustainable, biomass-derived surfactants with improved biodegradability.

**Table 2:** Surfactant properties of the synthesized butenolide surfactants. CMC: critical micelle concentration

Surfactant	CMC [mM]	Krafft point [°C]	Biodegradability after 28 d^a^ [%]
137a	28.72	n.d.	74
137b	5.81	31	78
137c	0.81	43–45	67
138a	n.d.	n.d.	37.5
138b	5.76	n.d.	50
138c	n.d.	n.d.	26

a Biodegradation according to the OECD Test guideline 301 F.^277^

### 4.4. Synthetic dyes

The global dye and pigment industry relies heavily on petrochemical feedstocks, contributing significantly to environmental pollution and resource depletion.^278,279^ In contrast, plant-based dyes often suffer from limited performance and high production costs.^280^ An ideal compromise lies in the development of synthetic dyes derived from biomass intermediates. For example, the work by Saska *et al.*^281^ highlights the sustainable production of dyes using butenolide derivatives obtained from bio-based furan platforms.^281^ Similarly, HFO can serve as a valuable platform chemical for the synthesis of functional dyes.

Desvals *et al.*^177^ demonstrated that HFO can be utilised as a building block for the scalable synthesis of polymethine dyes 139 (Scheme 82).^177^ Their synthetic route starts with the acetalization of HFO 1 to form 5-ethoxy-2(5*H*)-furanone (5-ethoxybutenolide, 7h), followed by α-bromination and subsequent hydrolysis to generate formylpyruvate, a key intermediate. Condensation of formylpyruvate with thiobarbituric acid derivatives under acidic conditions yields the target polymethine dyes 139 with overall yields of 56–62%.

**Scheme 82:**
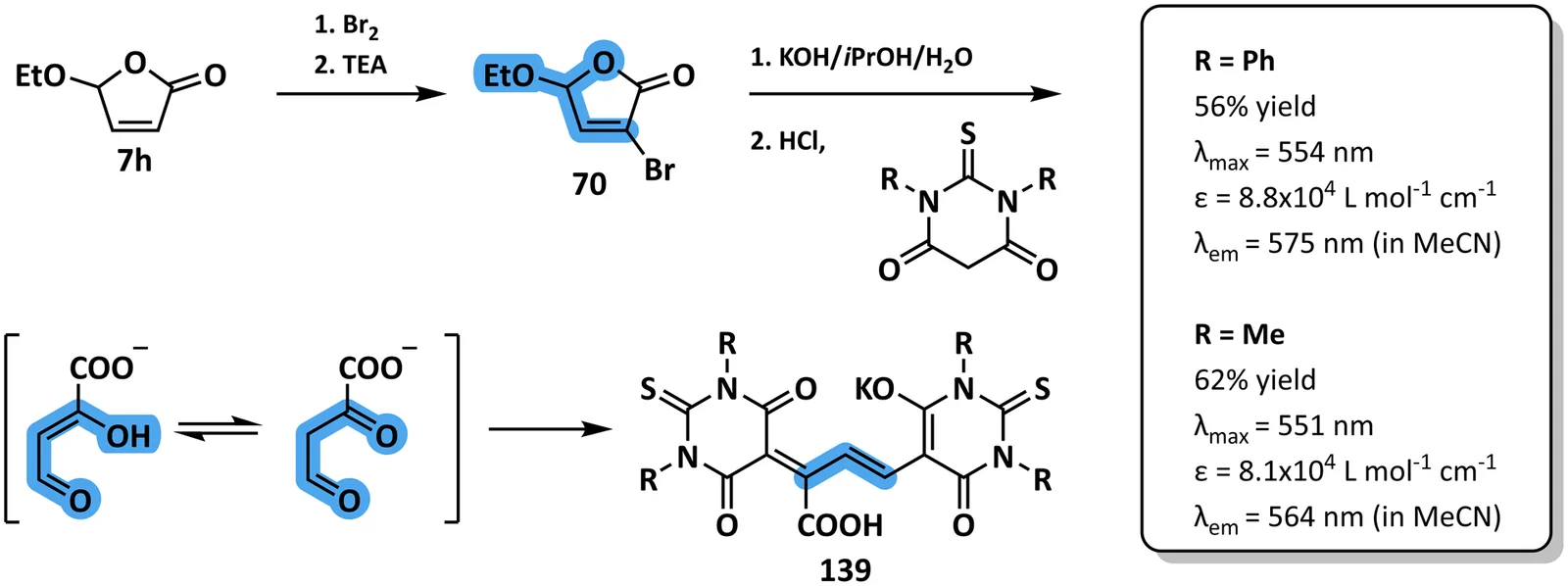
Synthetic route toward polymethine dye, utilising 5-ethoxy butenolide as starting material, as reported by Desvals *et al.* in 2022.^177^

The photophysical properties of the synthesized dyes were investigated using a combination of experimental and theoretical approaches. Absorption and emission spectroscopy revealed a low solvatochromic effect, while luminescence quenching by molecular oxygen indicated partial triplet multiplicity character of the lowest excited state. Density functional theory (DFT) calculations were employed to determine the preferred conformations and optical properties of these compounds. Furthermore, simulations of the vibrationally resolved electronic absorption spectra, based on the Franck–Condon principle, revealed a dominant absorption band accompanied by a vibronic sideband, characteristic of organic chromophores in solution.^282,283^ Given their established applications in photometric detection of enzyme activities, these results further demonstrate the versatility of HFO as a sustainable building platform for the development of functional dye systems.

## 5. HFO-based polymers and their application

Conventional, petroleum-derived polymers are globally used across a broad range of applications, including packaging,^284^ coatings,^285,286^ lubricants,^287^ and healthcare products.^288,289^ However, as global awareness of the climate crisis, resource depletion, and plastic pollution continues to grow, the transition toward materials derived from renewable resources has evolved from a niche scientific interest into a mainstream industrial and political priority.^4,290^

### 5.1. Radical polymerisation

Within the broader landscape of bio-based polymers, the development of bio-based monomers suitable for radical polymerisation represents a key area of research.^291–293^ Conventional radical polymerisation, alongside controlled techniques such as atom transfer radical polymerisation (ATRP),^294–296^ reversible addition–fragmentation chain transfer (RAFT),^297,298^ and nitroxide-mediated polymerisation,^299,300^ remains one of the most widely employed and industrially scalable techniques for producing synthetic polymers. These processes typically require monomers bearing reactive double bonds that readily participate in radical reactions, such as acrylates and styrene. Although the polymer industry continues to rely heavily on such fossil-derived monomers, a significant shift toward bio-based alternatives is underway.^293^ Prominent examples of renewable monomers are α-methylene-γ-butyrolactones,^301–304^ itaconic acid derivatives^305^ and muconates.^306^

In this context, 5-substituted butenolides have emerged as promising candidates for radical polymerisation. Early investigations date back to the 1960s, when Schenck and colleagues reported a homopolymerisation of 5-ethoxybutenolide 7h, by using 0.1 eq. BPO as a radical initiator, they successfully obtained the first butenolide-based homopolymer (Scheme 83). However, only limited characterisation was reported. The primary and only polymer property described was the molecular weight of *ca.* 3 kg mol^−1^, while other key parameters such as thermal and mechanical properties remained unexplored.^29^

**Scheme 83:**
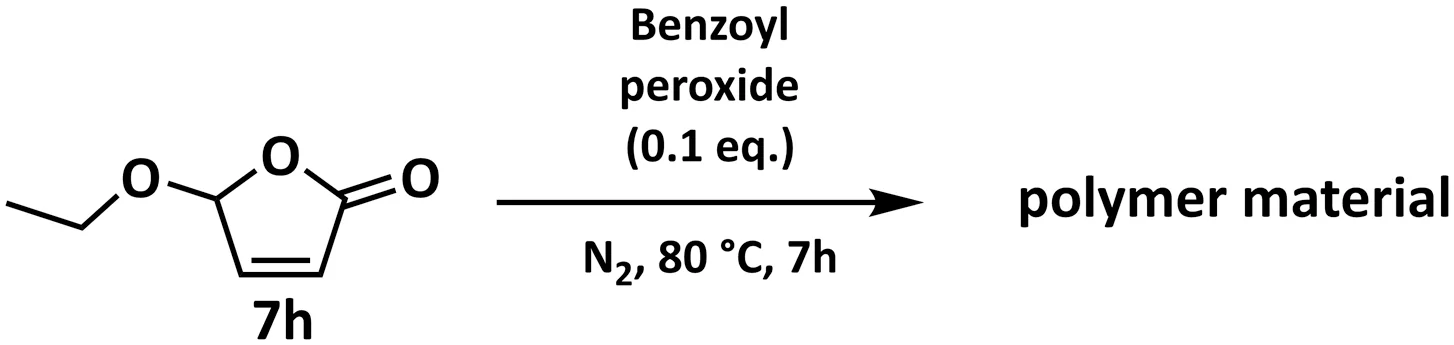
First homopolymerisation of 5-ethoxybutenolide by Schenck and colleagues in 1966.^29^

Poskonin *et al.*^307^ (1997) further investigated the homo- and copolymerisation of alkoxy- and acyloxy-substituted butenolides (Scheme 84).^307,308^ In bulk at 85 °C, using BPO (0.03 eq.) as radical initiator, homopolymerisation resulted in relatively low conversions of approximately 35%.^307^ Copolymerisation with common monomers such as methyl methacrylate (MMA) and styrene led to improved overall yields, however the incorporation of the butenolide into the resulting copolymers remained limited. For example, the copolymerisation of 5-ethoxybutenolide with MMA (Table 3, entry 5) yielded only ∼5% incorporation of the butenolide. Similar trends were found for styrene and for 5-butoxybutenolide.

**Scheme 84:**
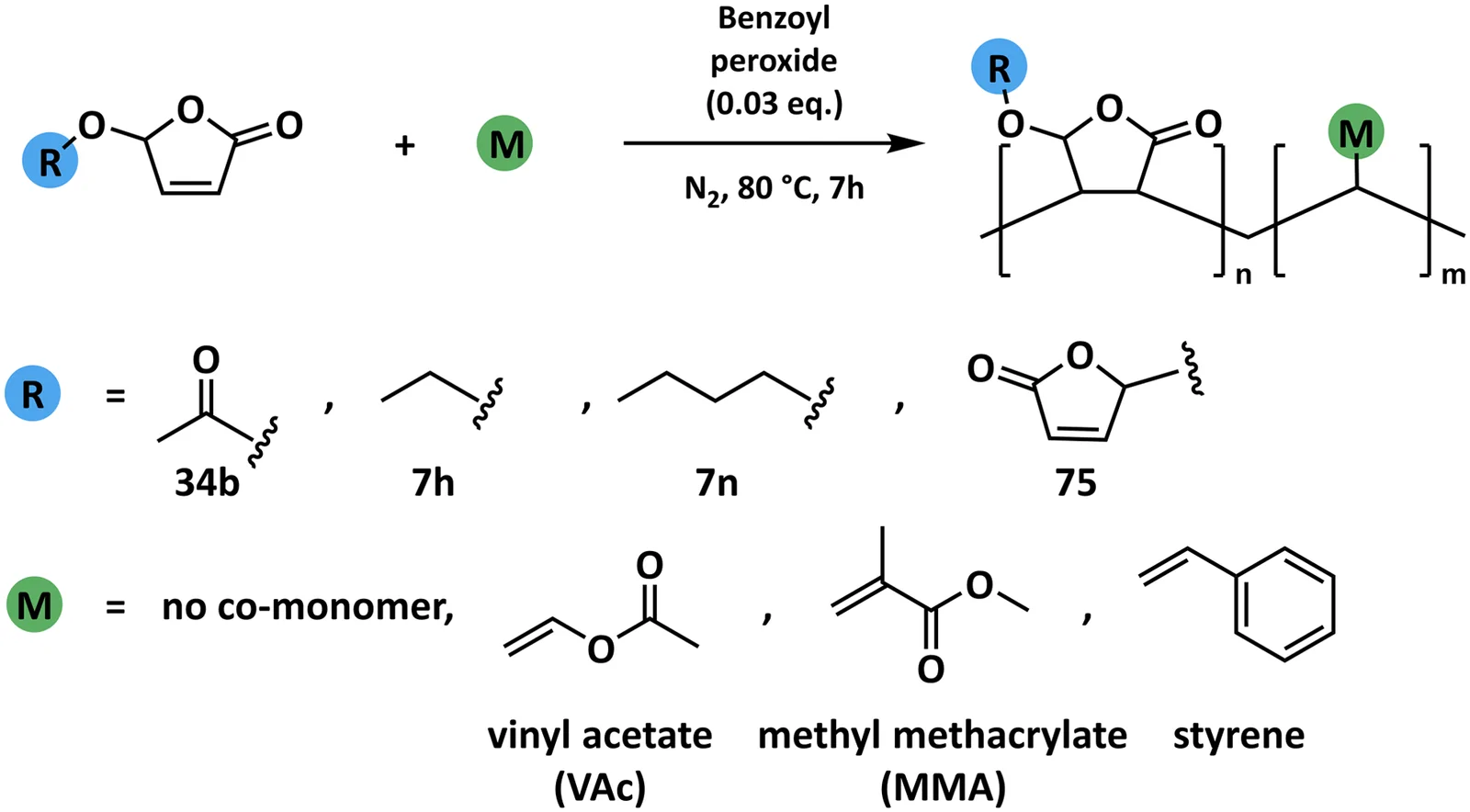
Homo- and copolymerisations of 5-acetoxy-, 5-ethoxy-, 5-butoxybutenolide and 5,5′-oxybis(furan-2(5*H*)-one) with vinyl acetate, methyl methacrylate and styrene by Poskonin *et al.* in 1997 and 1999.^307,308^

**Table 3:** Homo- and copolymerisation of 5-acetoxy-, 5-ethoxy-, 5-butoxybutenolide and 5,5′-oxybis(furan-2(5*H*)-one) (B) with vinyl acetate (VAc), methyl methacrylate (MMA) and styrene by Poskonin *et al.* in 1997 and 1999.^307,308^ B : M2 : BPO 1 : 1 : 0.03 at 85 °C (entries 1–4) or 80 °C (entries 5–13), solventless, atmosphere not reported

Entry	B	M2	B : M2 [%]	*t* [h]	*Y* [%]	*M*_w_ [kg mol^−1^]^a^	*T*_m_ [°C]
1	34b	—	n.d.	7	35	0.6	182–190
2	34b	MMA	n.d.	7	67	3.5	140–155
3	34b	Styrene	n.d.	7	68	3.9	172–180
4	34b	VAc	n.d.	2	75	3.4	153–158
5	7h	MMA	5 : 95	4.5	68	4.0	174–187
6	7h	Styrene	4 : 96	9	64	4.0	130–143
7	7h	VAc	60 : 40	4.5	61	4.6	115–125
8	7n	MMA	5 : 95	4.5	62	4.0	195–200
9	7n	Styrene	4 : 96	12	28	4.0	157–172
10	7n	VAc	33 : 67	18	57	4.2	137–150
11	75	MMA	5 : 95	6	63	4.0	178–190
12	75	Styrene	4 : 96	10	70	3.5	123–129
13	75	VAc	60 : 40	4.5	55	4.7	124–131

a By cryoscopy in 1,4-dioxane.

In contrast, the electron-rich vinyl acetate (Table 3, entries 4, 7 and 10) achieved significantly higher incorporations of the butenolide-based monomers. When the authors further investigated 5,5′-oxybis(furan-2(5*H*)-one), which contains two reactive C=C bonds (Table 3, entries 11–13), they observed similar trends. This compound was anticipated to produce branched polymers, and indeed swelling was observed upon dissolution of the final polymers.^307,308^

By using cryoscopy in 1,4-dioxane the molecular weights were determined (Table 3). While the chain lengths of the copolymers were in a similar range from 3.3–4.6 kg mol^−1^, the homopolymerisation achieved only 0.6 kg mol^−1^, likely due to the low conversion (entry 1). The melting temperatures (*T*_m_) ranged from 115 °C to 200 °C, indicating that the polymer properties can be tuned through variation of substituents. The authors investigated different ratios between monomer and initiator and observed the for chain-growth polymerisation typical increase in molecular weights when lowering the initiator concentration.^307,308^ Finally, the authors compared copolymerisations using MAnh instead of 5-substituted butenolides and observed a significant increase in molecular weight (13.8–15.2 kg mol^−1^). They attributed this to the ability of the 5-substituted butenolide to undergo chain transfer to monomer *via* hydrogen abstraction at the 5-position. This phenomenon cannot occur with MAnh, resulting in higher molecular weights.

Driven by the goal of developing bio-based alternatives to petroleum-derived acrylates, the Feringa group studied the polymerisation of 5-alkoxybutenolides in depth (Scheme 85).^101,107^ Hermens *et al.*^107^ examined the copolymerisation reactions between four different 5-alkoxybutenolides with the comonomers vinyl neodecanoate (VeoVa-10) and dodecyl vinyl ether (DVE), using Trigonox 42S (*t*_1/2_: 1 h at 114 °C) as initiator in 1-methoxy-2-propanol (PGME). Polymerisation kinetics were monitored by ^1^H-NMR spectroscopy using an internal standard in a closed vial. These studies confirmed low conversion in homopolymerisation; however, copolymerisation with electron-rich comonomers (VeoVa 10 or DVE) resulted in significantly higher conversions.^107^

**Scheme 85:**
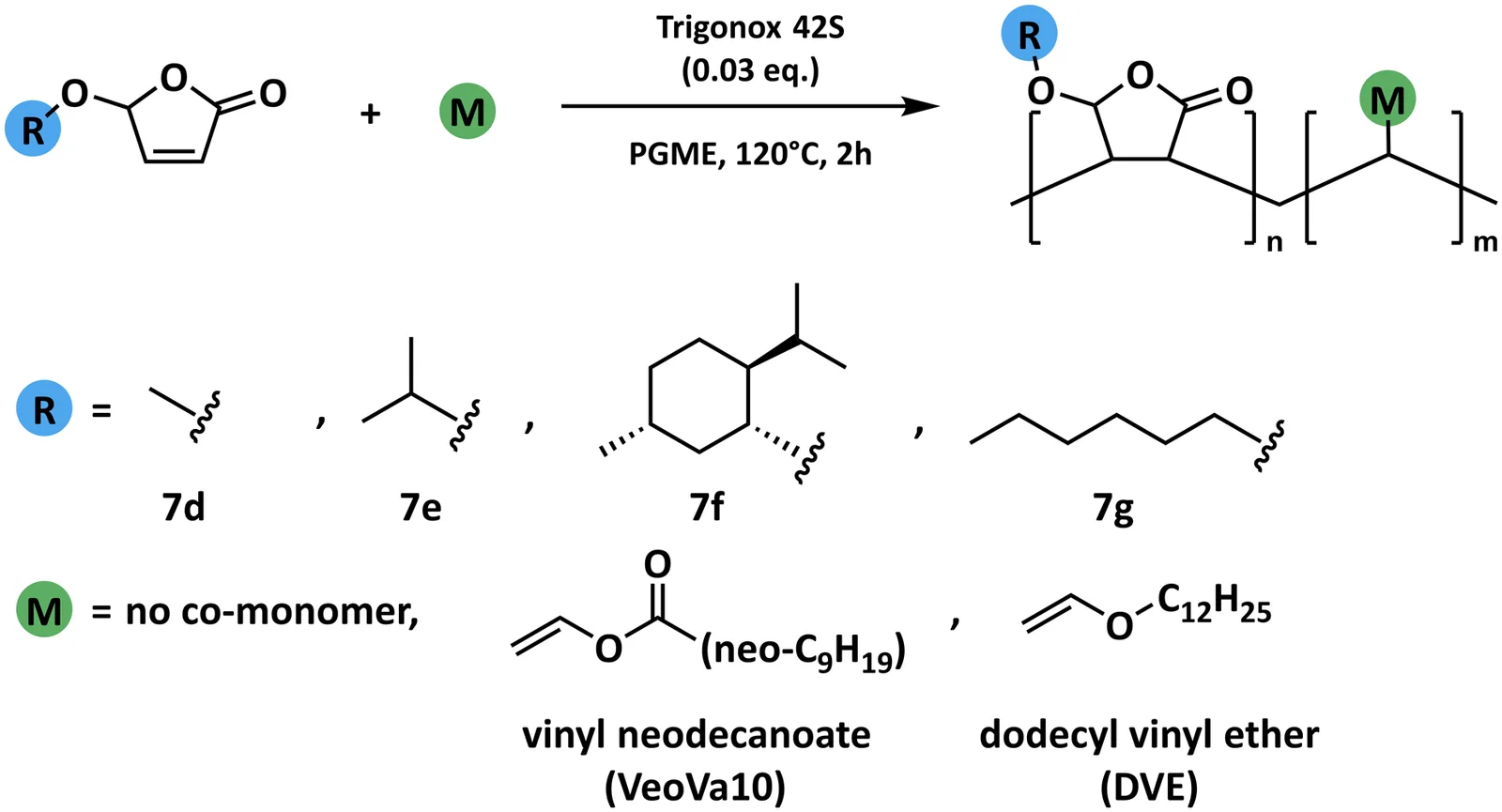
Homo- and copolymerisations in organic solvent of 5-alkoxybutenolides with vinyl monomers by Hermens *et al.* (2020).^107^

Properties of the copolymers were investigated using gel permeation chromatography (GPC) and differential scanning calorimetry (DSC) (Table 4). Molecular weights ranged from 2.8 to 4.9 kg mol^−1^, corresponding to oligomeric chains of 7 to 15 repetition units. Here, the authors observed a difference in *M*_w_ when using VeoVa-10 (*M*_w_ around 3 kg mol^−1^) and DVE (*M*_w_ around 4.5 kg mol^−1^). Investigation of the *T*_g_s of the copolymers indicated a difference in *T*_g_ when using DVE as comonomer (*T*_g_ ranging from −62 to −72 °C) and for VeoVa-10 (*T*_g_ ranging from −27 to 12 °C). The *T*_g_ could be slightly altered by changing the alkoxy chain length and rigidity of the alcohol at the 5-alkoxybutenolide. It should be noted however that the molecular weights and *T*_g_s were measured without thorough purification of the polymer. Therefore, residual monomers in the system could influence the final polymer properties.

**Table 4:** Comparison of initial copolymerisation rates, molecular weight distribution and glass transition temperatures *T*_g_ for equimolar mixtures of butenolide and comonomer in 1-methoxy-2-propanol at 120 °C in a closed vial (air), initiated by the addition of 3 mol% Trigonox 42S^107^

Entry	B	M2	*C*_comb_ [%]	*K*_ini_ [10^−4^ s^−1^]	*M*_w_ [kg mol^−1^]	*Đ*	*T*_g_ [°C]
1	7d	—	53	1.4	n.d.	n.d.	n.d.
2	7d	VeoVa10	99	5.5	2.8	2.4	12
3	7d	DVE	95	14	4.9	3.0	−62
4	7e	—	37	1.1	n.d.	n.d.	n.d.
5	7e	VeoVa10	96	3.7	3.2	2.0	12
6	7e	DVE	>99	20	4.3	2.4	−67
7	7f	—	30	0.92	n.d.	n.d.	n.d.
8	7f	VeoVa10	92	2.9	3.1	2.7	24
9	7f	DVE	>99	11	4.7	3.0	16
10	7g	—	51	0.92	n.d.	n.d.	n.d.
11	7g	VeoVa10	92	3.6	3.3	2.6	−27
12	7g	DVE	>99	21	4.6	3.0	−72

Building on these initial findings, Jensma *et al.*^309^ from the same group further explored the functional reactivity of the resulting butenolide copolymers (Scheme 86).^309^ Taking advantage of the acetal functionality present in the butenolide moiety, they investigated the acid-catalysed hydrolysis of bioactive alcohol in aqueous media. Hydrolysis rates were found to depend on three key parameters: (i) pH: lower pH accelerates hydrolysis; (ii) polymer polarity: increased hydrophilicity enhances water accessibility; (iii) the nature of the released alcohol: bulkier or more hydrophobic groups slow down the release. This tunability could enable applications in fragrance delivery (controlled release of volatile alcohols for long-lasting scents), agriculture (controlled pesticide release), and biomedical fields (drug delivery systems).^309^

**Scheme 86:**
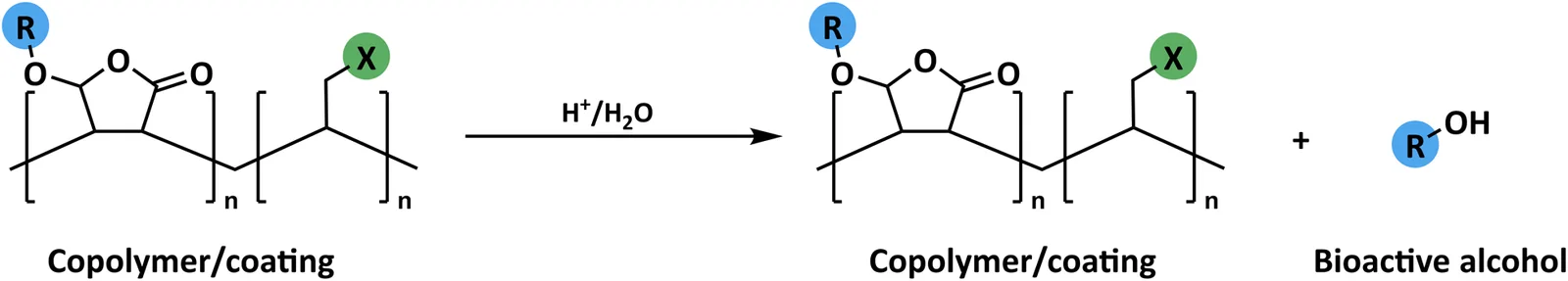
Controlled release of bioactive alcohols from 5-alkoxybutenolide copolymers with ethylene glycol vinyl ether (EGVE) and dodecyl vinyl ether (DVE) as reported by the Feringa group in 2026.^309^

The initial studies on 5-alkoxybutenolides revealed minimal differences in reactivity across varying alkyl substituents. In contrast, extension to butenolide esters, carbonates, and carbamates resulted in a significant increase in copolymerisation reactivity (*K*_ini_) (Scheme 87 and Table 5) as reported by Feringa and coworkers in 2023.^100^ This enhancement is attributed to the electron-withdrawing nature of these substituents, which activates the double bond toward radical copolymerisation. When using an even more electron-withdrawing substituent, 5-benzoyloxybutenolide (2b) (Table 5, entry 2) the authors observed an enhanced reactivity compared to the other 5-acyloxybutenolides, further supporting their hypothesis.

**Scheme 87:**
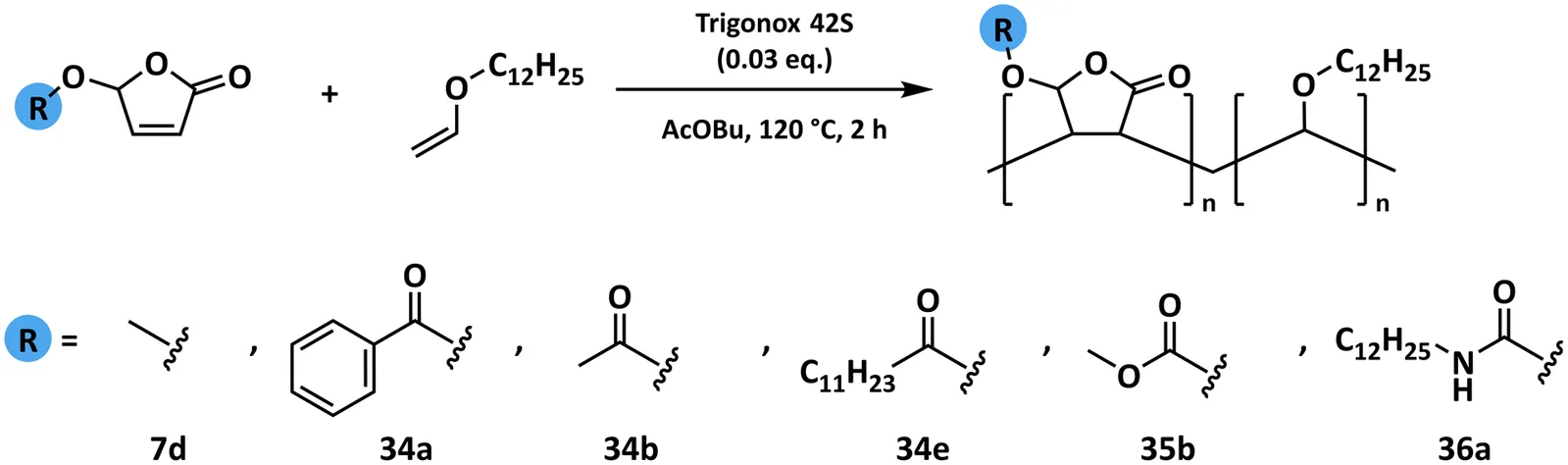
Copolymerisation of 5-substituted butenolides with dodecyl vinyl ether (DVE) using Trigonox 42S as initiator at 120 °C in a closed vial (air) in *n*-butyl acetate (AcOBu) as reported by the Feringa group in 2023.^100^

**Table 5:** Comparison of initial copolymerisation rates, molecular weight distribution (*M*_w_), degree of polymerisation (DP) and glass transition temperatures (*T*_g_) and combined conversion (*C*_comb_), for equimolar mixtures of butenolide (*B*) and dodecyl vinyl ether (DVE) in *n*-butyl acetate at 120 °C in a closed vial (air), initiated by the addition of Trigonox 42S (3 mol% *vs.* total monomer content), as reported by the Feringa group in 2023^100^

Entry	B	*C*_comb_ [%]	*K*_ini_ [10−^4^ s^−1^]	*M*_w_ [kg mol^−1^]	*Đ*	DP	*T*_g_ [°C]
1	7d	91	7.9	4.1	1.8	14	−4
2	34a	96	36.8	12.4	2.9	21	34
3	34b	95	21.3	9.8	2.5	22	30
4	34e	98	26.6	13.9	3.1	18	10
5	35b	91	23.5	9.7	2.4	21	41
6	36a	87	14.9	10.8	2.8	14	58

Analysis of the resulting copolymers (GPC and DSC) revealed increased molecular weights for 5-acyloxybutenolides, butenolide carbonates and carbamates (9.7–13.9 kg mol^−1^, Table 5, entries 2–6) compared to those of 5-methoxybutenolide (4.1 kg mol^−1^, Table 5, entry 1), along with higher degrees of polymerisation (≈ 20 *vs*. 14 repetition units). Additionally, the incorporation of acyloxy, carbonate and carbamate substituents led to increased and tuneable *T*_g_s, likely due to enhanced intermolecular interactions (*e.g.*, hydrogen bonding) and higher molecular weights. Comparing entries 3 and 4, the authors observed a typical change in *T*_g_ with the flexible alkyl chain of butenolide 34e lowering this characteristic thermal property.

Furthermore, the copolymerisation of 5-acetoxybutenolide (monomer 34b) with a broader range of comonomers such as (meth)acrylates and styrene was investigated (Table 6). Electron-rich vinyl monomers such as vinyl ethers (entries 1 and 2), vinyl esters (entry 3), and vinyl lactams (entries 4 and 5) exhibited favourable reactivity, consistent with the electron-deficient nature of the 5-acetoxybutenolide double bond. In contrast, highly reactive monomers such as butyl acrylate (entry 6), butyl methacrylate (entry 7) and styrene (entry 8) showed poor copolymerisation behaviour, with high conversion of the comonomer but limited conversion of the butenolide (16–30%).^100^

**Table 6:** Comparison of initial (co)polymerisation rates for equimolar mixtures of 5-acetoxybutenolide B-34b and comonomer (M2) in *n*-butyl acetate at 120 °C in a closed vial (air), initiated by the addition of Trigonox 42S (3 mol% *vs*. total monomer content), as reported by the Feringa group in 2023. Comonomers used are dodecyl vinyl ether (DVE), ethylene glycol vinyl ether (EGVE), vinyl neodecanoate (VeoVa-10), *N*-vinyl pyrrolidone (NVP), *N*-vinyl caprolactam (NVC), butyl acrylate, butyl methacrylate and styrene^100^

Entry	M2	*C*_comb_ [%]	*C* (B-34b) [%]	*C* (M2) [%]	*K*_ini_ [10^−4^ s^−1^]
1	DVE	95	100	91	21.3
2	EGVE^a^	92	99	86	111
3	VeoVa10	94	90	97	7.3
4	NVP^a^	99	99	98	167
5	NVC^a^	95	97	93	48.5
6	Butyl acrylate	65	30	100	11.4
7	Butyl methacrylate	57	16	97	3.9
8	Styrene	59	21	97	3.4
9	—	—	30	—	0.22

a *N*-Methyl pyrrolidone was used as solvent due to monomer solubility issues with *n*-butyl acetate.

Combining kinetic studies with density functional theory (DFT) calculations, Feringa and coworkers demonstrated that 5-alkoxy- and 5-acyloxy- substituted butenolides occupy an intermediate position between acrylates and MAnh in terms of radical polymerisation reactivity (Fig. 15).^100^ Substituent effects at the 5-position play a decisive role in reactivity and governing both homo- and copolymerisation behaviour: unsubstituted (RO-5C-H, *i.e.* 2-furanone) or alkyl-substituted butenolides (RO-5C-C, *i.e.* 5-alkyl furanone) exhibit generally lower reactivity, which is attributed to hyperconjugative donation from σ_C-H_ or σ_C-C_ bonds into the π-system of the α,β-unsaturated carbonyl. Thus, the introduction of electron-withdrawing substituents, such as 5-alkoxy or 5-acyloxy groups, reduces this electron donation and increases the reactivity of the α,β-unsaturated olefinic bond toward radical polymerisation.

**Fig. 15:**
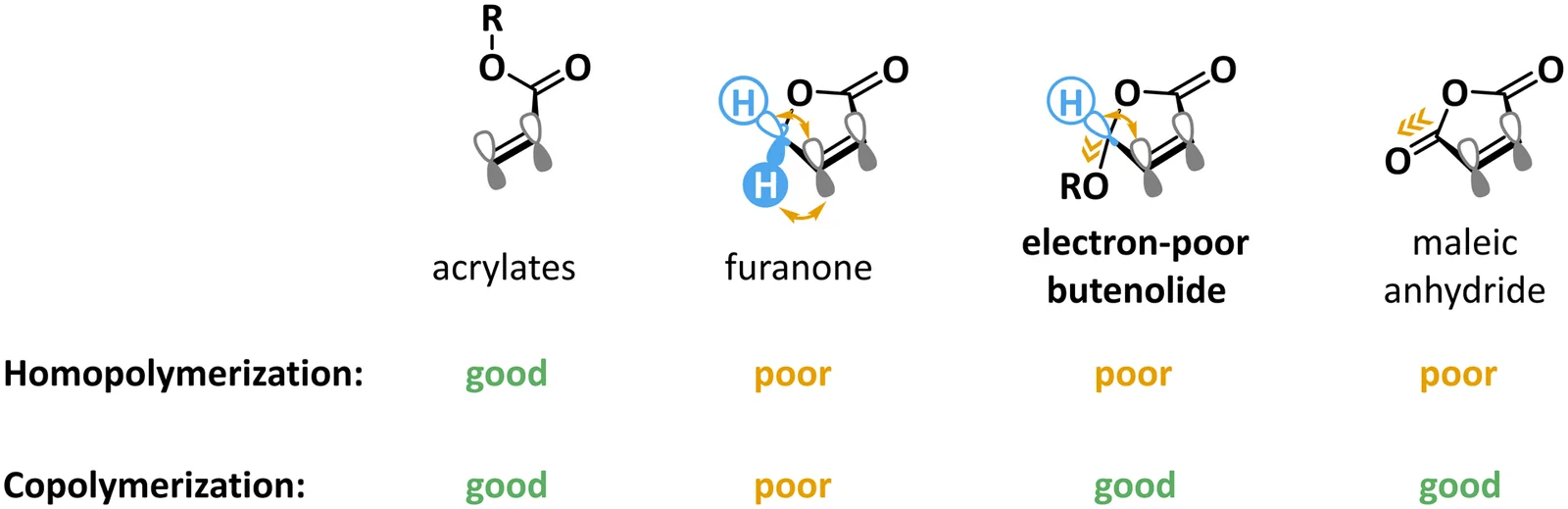
Reactivity of 5-substituted butenolides for radical polymerisation compared with acrylates and maleic anhydride (MAnh) as reported by the Feringa group in 2023.^100^ Adapted from ref. 100 under the licence CC BY 4.0 (ACS, © 2023).

The early work of Schenck and Poskonin established the foundation for subsequent studies by Feringa and colleagues, in which a wide range of bulk and solventborne radical polymerisations were systematically investigated. Homopolymerisation of 5-alkoxy- and 5-acetoxybutenolides was consistently found to be inefficient. In this context, electron-poor butenolides exhibit behaviour analogous to MAnh as a monomer in radical polymerisations, where homopolymerisation is unfavourable, while copolymerisation proceeds efficiently. Suitable comonomers for 5-substituted butenolides include vinyl ethers, vinyl esters, and vinyl lactams, which possess electron-rich double bonds due to resonance donation from heteroatoms (oxygen or nitrogen). In contrast, highly reactive comonomers, such as (meth)acrylates and styrene result in poor conversion of the butenolide units. However, copolymerisation with electron-rich monomers such as DVE affords materials with molecular weights and thermal properties comparable to conventional acrylic polymers, while additionally introducing an acetal functionality that is absent in acrylate-based systems, thereby providing a versatile handle for post-polymerisation modification or controlled release applications. Finally, the availability of bio-based electron-rich co-monomers offers a viable pathway toward fully bio-based copolymer system.^101^

### 5.2. Cationic ring-opening polymerisation

An alternative to radical polymerisation of butenolides is cationic ring-opening polymerisation (CROP).^310–313^ This technique allows for precise control over polymer architecture and molecular weight distribution. It is used to synthesize a variety of polymers, particularly those derived from cyclic ethers, lactones, lactams and other heterocyclic monomers. Unlike radical polymerisation, CROP proceeds *via* a cationic mechanism, where the monomer ring is opened and incorporated into the polymer chain. This process is initiated by strong electrophiles, such as Brønsted or Lewis acids.

Recently, in 2025, the cationic ring-opening copolymerisation of 5-alkoxybutenolides was investigated by Higashibata *et al.*^104^ (Scheme 88).^104^ They started their work synthesizing three different 5-alkoxybutenolides (R = Me, iPr and *t*Bu) and investigated them for cationic ring-opening homopolymerisation. Utilising a Lewis acid catalyst, B(C_6_F_5_)_3_ and dichloromethane (DCM) as solvent at −78 °C, the authors did not observe any conversion at all. The inertness of the 5-alkoxybutenolide is likely derived from low ring-strain and unfavourable propagation kinetics. They then switched to cationic ring-opening copolymerisation and found vinylcyclohexene oxide (VCHO) as a suitable comonomer (Scheme 88). The authors observed 82% conversion of the oxirane and 23% conversion of 5-methoxybutenolide 7d (Table 7, entry 1) after 3 min, using the previously mentioned conditions. Switching to 5-isopropoxybutenolide 7e (Table 7, entry 2) and 5-tertbutoxybutenolide 7o (Table 7, entry 3) yielded similar conversions.^104^

**Scheme 88:**
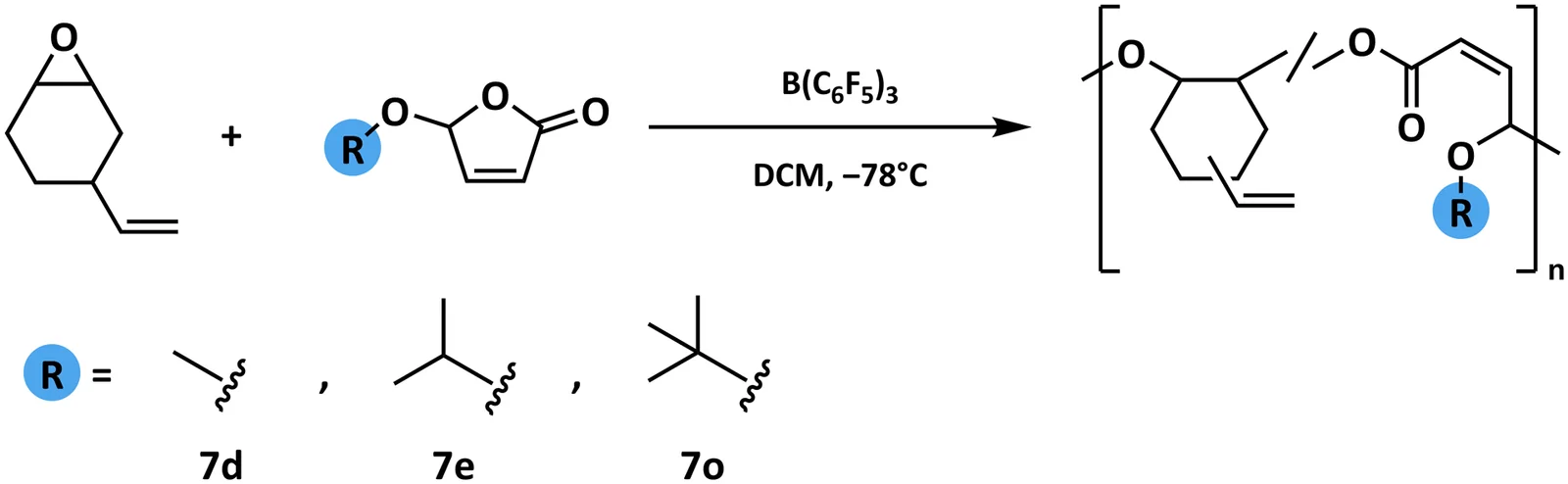
Cationic copolymerisation of 5-alkoxy-2(5*H*)-furanones with vinylcyclohexene oxide as reported by Higashibata *et al.* (2025).^104^

**Table 7:** Cationic copolymerisation of 5-alkoxy-2(5*H*)-furanones (B, 5-alkoxybutenolides) with VCHO using a Lewis acid catalyst B(C_6_F_5_)_3_, as reported by Higashibata *et al.* (2025).^104^ [VCHO]_0_ = 0.75 M, [B]_0_ = 0.75 M, [B(C_6_F_5_)_3_]_0_ = 1.0 (entry 1) or 3.0 (entries 2 and 3) mM, in DCM at −78 °C, N_2_. Acid hydrolysis of the polymers was conducted with 0.5 M HCl (aq.) in 1,2-dimethoxyethane at room temperature for 1 h (polymer: approximately 1 wt%)

Entry	B	Time (min)	*C* (VCHO) (%)	*C* (B) (%)	*M*_n_ (kg mol^−1^)	*M*_n_ (kg mol^−1^) after degradation	*T*_g_ (°C)
1	7d	3	82	23	17.8	1.2	60
2	7e	1	80	26	21.2	0.4	45
3	7o	7.5	74	25	13.8	0.4	66

The number-average molecular weights (*M*_n_) were determined by GPC and found to be in the range of 13.8–21.2 kg mol^−1^ (Table 7). The resulting copolymers featured ester and acetal linkages in the polymer backbone, which enabled acid-triggered degradability. Upon acid-mediated hydrolysis with HCl, the *M*_n_ values decreased significantly to 0.4–1.2 kg mol^−1^, demonstrating efficient degradation. The thermal properties of the polymer were determined *via* DSC and the authors observed *T*_g_ in the range of 45 to 66 °C. The difference of the alkoxy substituent affected the thermal properties, as the side chains add flexibility and bulkiness. The order of *T*_g_s (substituents: *t*Bu > Me > iPr) is similar to poly(alkyl acrylates) and poly(alkyl methacrylates).^314^

The team also demonstrated cationic terpolymerisation (Scheme 89) of 5-alkoxybutenolides with oxiranes and vinyl ethers (VE), yielding sequence-controlled polymers. Here, even lower conversions were observed for the 5-alkoxybutenolide monomers (7–11%). Depending on the substituent of the 5-alkoxybutenolide and the vinyl ether, different molecular weights between 5.4–28.1 kg mol^−1^ were found.

**Scheme 89:**
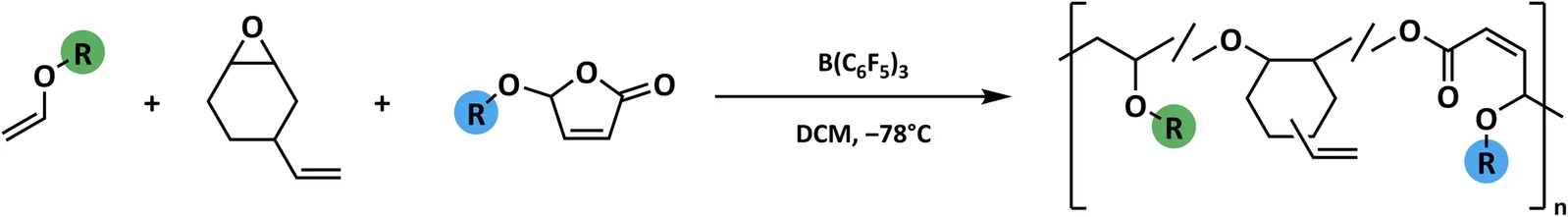
Cationic terpolymerisation of 5-alkoxy-2(5*H*)-furanones with vinylcyclohexene oxide and vinyl ethers, as reported by Higashibata *et al.* (2025).^104^ [VE]_0_ = 0.80 M, [VCHO]_0_ = 0.20 M, [ROFO]_0_ = 0.80 M, [B(C_6_F_5_)_3_]_0_ = 1.0 mM, in dichloromethane at −78 °C.

Finally, the resulting copolymers or terpolymers underwent post-polymerisation functionalisation *via* thiol–ene chemistry (Scheme 90), expanding their potential applications. The authors used 1-dodecanethiol as model thiol, in combination with 2,2′-azobis(isobutyronitrile) (AIBN) as a radical generator. By following the reaction with ^1^H-NMR they successfully showed incorporation of the thiol in the copolymer.

**Scheme 90:**
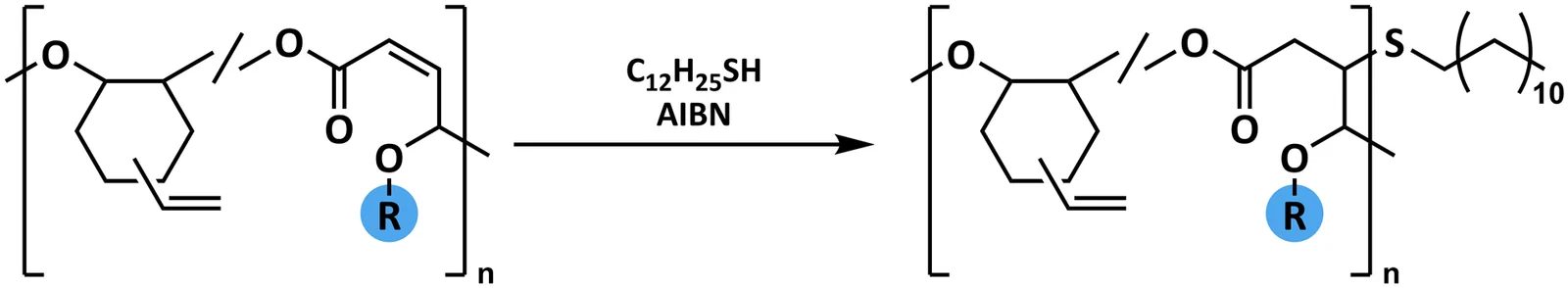
Post-polymerisation modification *via* thiol–ene chemistry with 1-dodecanethiol and AIBN as radical initiator, as reported by Higashibata *et al.* (2025). ([double bonds in copolymer]_0_ = 0.062 M, [C_12_H_25_SH]_0_ = 0.31 M, [AIBN]_0_ = 0.031 M, in toluene at 80 °C, 24 h), incorporated ratio = 40%.

The work of Higashibata *et al.*^104^ shows a promising methodology for cationic ring-opening copolymerisation of 5-alkoxybutenolides. It opens the path toward degradable polymers utilising the ring-opened form of the butenolide, alongside more control over polymer architecture and molecular weights compared to radical polymerisation.^104^

### 5.3. Resin technology

Synthetic resins are versatile polymers engineered to replace natural resins, offering tailored properties such as durability, adhesion, and chemical resistance. They can be formulated as thermoplastics^315^ (meltable and reusable) or thermosets^316^ (permanently hardened after curing). Widely used in adhesives, coatings, plastics, and composites, they are indispensable for modern polymer industries. However, the petrochemical origin of conventional synthetic resins poses significant sustainability concerns. Accordingly, the development of bio-based resins, derived from renewable feedstocks, such as 5-substituted butenolides has emerged as an important research direction.

Feringa and colleagues (2021) explored the application of 5-substituted butenolides in resin technologies for the paint and coatings industry. Initial studies focused on solventborne systems, in which the monomers were polymerized in organic solvents such as 1-methoxy-2-propanol using thermal initiators.^103^ Following casting and film formation of the polymer mixture on a substrate (Fig. 16), the resulting coatings exhibited tuneable properties, which could be modulated by varying the substituent at the 5-position and the identity of the comonomer. Utilising this method for applications in coatings does, however, lead to the emission of volatile organic compounds (VOC).

**Fig. 16:**
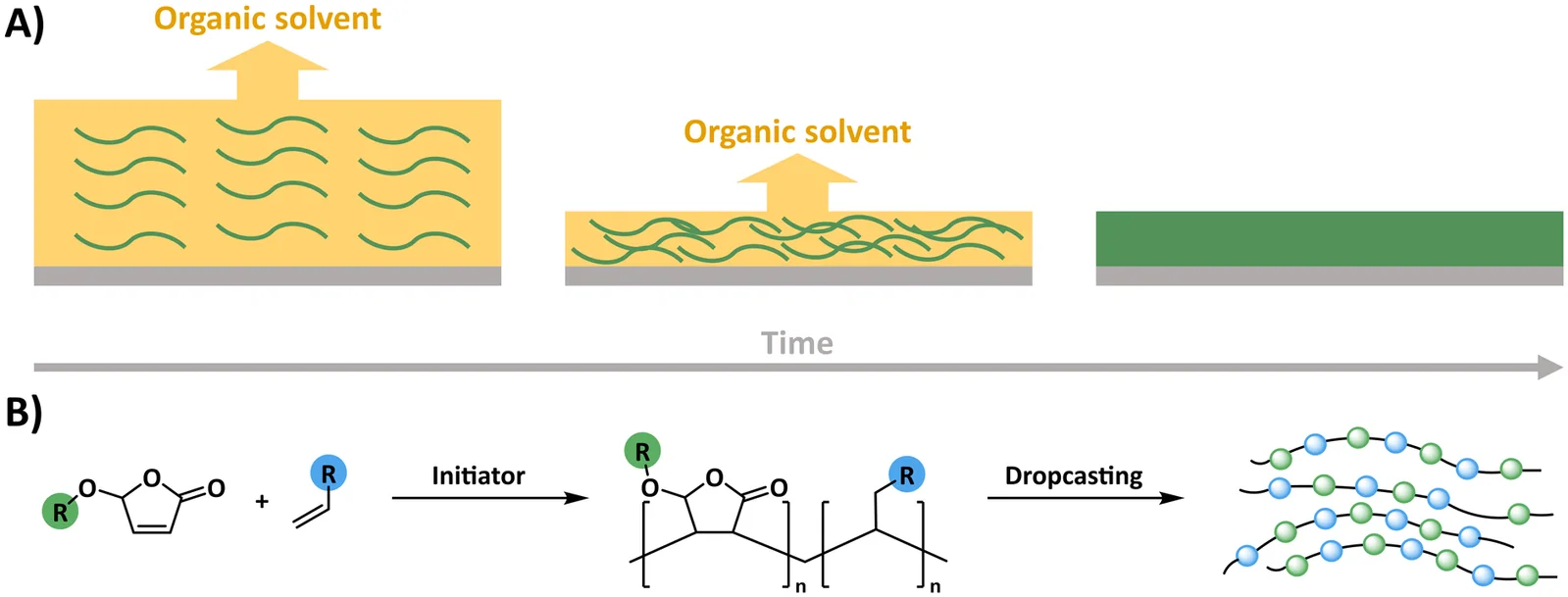
Preparation of solventborne butenolide based resins and coatings. (A) Preparation of coatings from solventborne resins. After evaporation of organic solvent polymer chains entangle and form a small film. (B) Copolymerisation of 5-substituted butenolides with a vinyl comonomer to form a copolymer for film formation.^103^

In subsequent work by Hoevelmann *et al.* (2025), the resin properties (such as solvent resistance and coating hardness) could be improved by utilising a polyamine hardener (Scheme 91).^317^ Upon thermal curing, the polyamine reacts (*i.e.* crosslinking) with the polybutenolide to form crosslinked networks, affording coatings and adhesives with excellent properties.

**Scheme 91:**
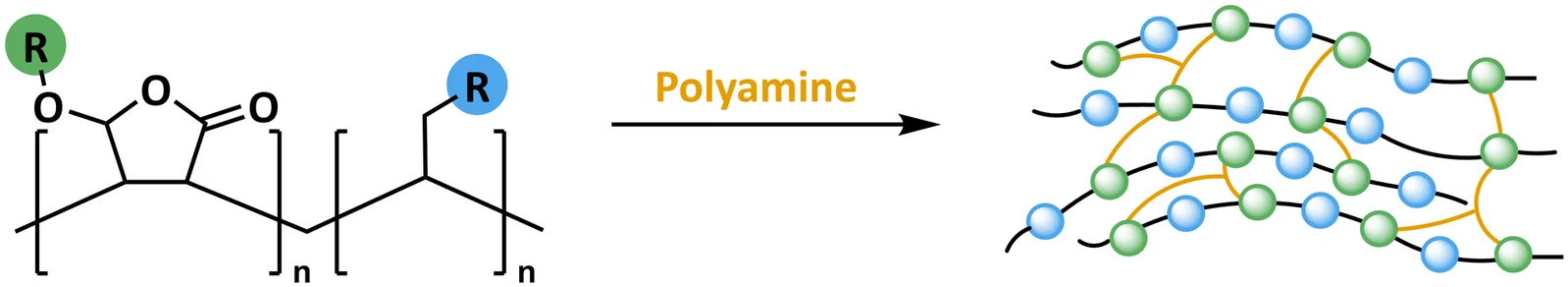
Preparation of solventborne butenolide based coatings and adhesives, crosslinked by polyamines as reported by Hoevelmann *et al.* (2025).^317^

Multiple polyamines for crosslinking were compared; two types of polyamines were selected as example. They first tested 2-methyl-1,5-diaminopentane as crosslinker and observed good solvent resistance (MEK double rubs) after 20 min curing at 100 °C (Table 8, entry 1). Through increasing the curing temperature to 130 °C (entry 2), they observed an increase in MEK double rubs to 200, indicating increased solvent resistance. The authors also examined the gloss and Buchholz hardness but did not observe any differences. Switching to triethylenetetramine they observed an increased solvent resistance already at a curing temperature of 80 °C (entry 3). By increasing the temperature, a decrease in gloss and Buchholz hardness was observed (entries 4 and 5).

**Table 8:** Coating performances of 5-methoxybutenolide 7a with isobutyl vinyl ether (1 : 1 copolymer) after curing for 20 min with a polyamine (calculated molar ratio to 7a 1 : 1), as described by Hoevelmann *et al.* in 2025^317^

Entry	Polyamine	*T*_curing_ [°C]	MEK double rubs	Gloss (GU)	Buchholz hardness (mm)
1	2-Methyl-1,5-diaminopentane	100	135	82.7	1.0
2	2-Methyl-1,5-diaminopentane	130	200	81.4	1.1
3	Triethylenetetramine	80	200	72.2	1.4
4	Triethylenetetramine	100	200	57.4	1.0
5	Triethylenetetramine	130	200	57.1	0.8

Despite these promising results, the exact crosslinking mechanism remains unclear. Proposed pathways include nucleophilic addition to, or incorporation of nitrogen into, the butenolide ring (*e.g.* imine formation) or ring-opening processes leading to amide-like structures. The study further demonstrated that curing temperature and polyamine functionality (di- *vs*. tetrafunctional) (Table 8) strongly influence coating performance, with elevated temperatures required to achieve optimal hardness and solvent resistance.

Another approach to butenolide-based resins involves radiation curing, as shown by Feringa and coworkers (Fig. 17).^101,107,318–320^ In this strategy, a formulation containing butenolide monomer, comonomer, and photoinitiator is applied to a substrate and subsequently polymerized upon UV-vis irradiation.^321^ This approach enables the formation of coatings from solvent-free (100% solids) formulations, thereby minimizing or eliminating VOC emissions. As in solventborne systems,^103^ coating properties can be tuned through variation of the comonomer, crosslinker and butenolide substituent. The incorporation of difunctional monomers enables crosslinking, leading to coatings with enhanced solvent and H_2_O resistance, as well as improved mechanical properties.

**Fig. 17:**
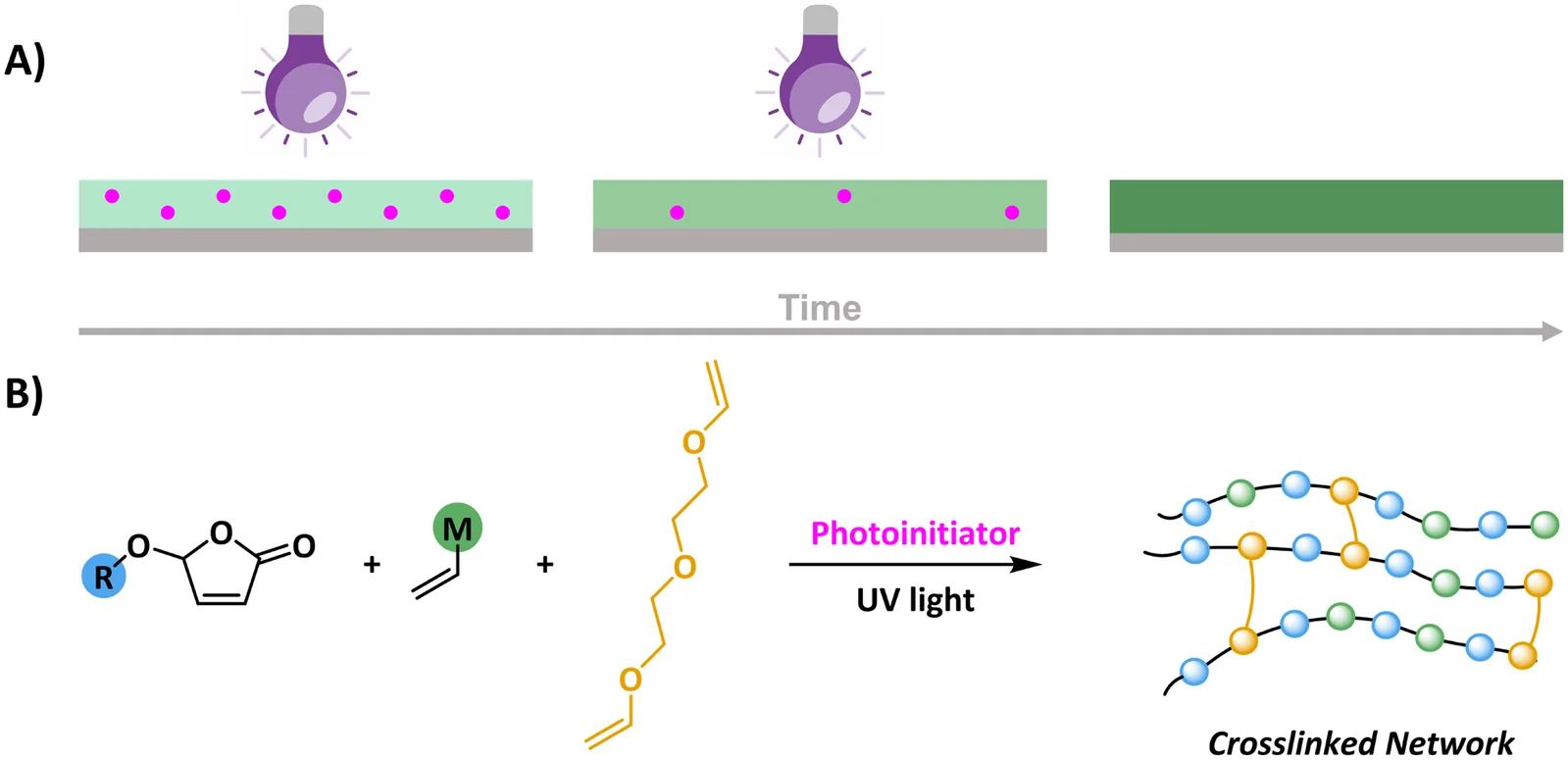
Preparation of butenolide based coatings using radiation cured coating formation. (A) Coating formation from monomer mixture (light green) and photoinitiator (pink) using UV light. (B) Radiation cured coatings using butenolide monomer, vinyl comonomer and crosslinker.^101,107,318–321^

Feringa and coworkers examined multiple formulations for UV-curable coatings with 5-substituted butenolides, changing butenolide monomers, comonomers, crosslinkers and monomer concentrations, of which key examples are depicted in Table 9. Using 5-methoxybutenolide (0.5 eq.), ethylene glycol vinyl ether (EGVE, 0.4 eq.) and diethylene glycol divinyl ether (DEGDVE, 0.05 eq.) poor solvent resistance (60 MEK double rubs) was observed (entry 1). Switching to 5-acetoxybutenolide (entry 2), an increase in solvent resistance was found which could be attributed to a higher degree of polymerisation (*vide supra*, Table 5). When decreasing the amount of crosslinker (DEGDVE) (entries 3 and 4), the authors did not observe a significant change. Ultimately, by utilising different coating formulations with different substituents on the butenolide monomer, the coating properties can be tuned for specific requirements.

**Table 9:** Coating performances including Knoop Hardness (HK) and solvent resistance (MEK double rubs) of UV-cured coatings of different butenolide-based monomers, EGVE as comonomer and DEGDVE as difunctional monomer (crosslinker). The coatings were prepared with 0.1 eq. photoinitiator phenyl bis(2,4,6-trimethylbenzoyl)phosphine oxide (BAPO)^101,107,318–320^

Entry	B	*n* (EGVE) [eq.]	*n* (DEGDVE) [eq.]	MEK double rubs	HK [kg mm^−2^]	*T*_g_ [°C]
1	7d	0.4	0.05	60	9.0	120
2	34b	0.4	0.05	>200	16.1	132
3	34b	0.45	0.025	>200	17.2	n.d.
4	34b	0.475	0.0125	>200	17.1	130

Le Dot *et al.* (2024)^159^ investigated the photopolymerisation of 5-alkoxybutenolides, including their copolymerisation with Elium® thermoplastic resins (Scheme 92).^159^ Elium® is a low-viscosity, MMA- resin developed for applications ranging from consumer products to high-performance composites, notable for its recyclability (up to 90% MMA monomer recovery).^322^ While simple 5-alkoxybutenolides (*i.e.* 5-ethoxybutenolide, 5-isopropoxybutenolide) exhibited poor homo- and copolymerisation behaviour with MMA, consistent with earlier studies by Poskonin^307,308^ and Feringa,^100^ functionalized monomers 7i and 7j showed improved performance.

**Scheme 92:**
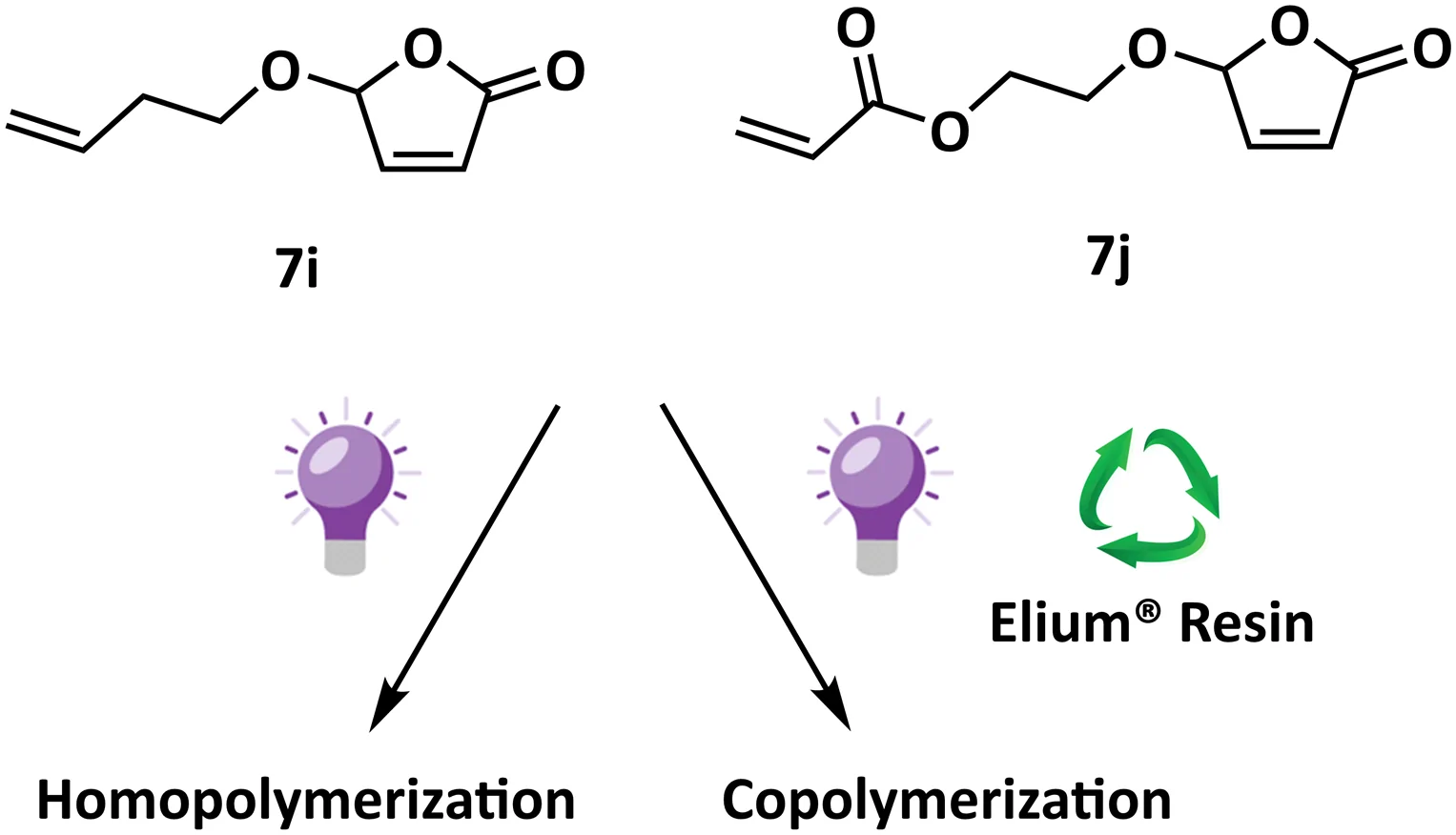
Homopolymerisation and copolymerisation with Elium® resin of 5-alkoxybutenolide derivatives using photopolymerisation as reported by Le Dot *et al.* in 2024.^159^ Adapted from ref. 159 under the licence CC BY 4.0 (Elsevier Ltd, © 2023).

To increase the bio-based content of the Elium® resin (E), various formulations incorporating butenolide monomers were evaluated (Table 10). In the case of butenolide monomer 7i, low conversions of both vinyl functionalities were observed in mixtures with the Elium® resin (entries 2–4), which was attributed to inhibition effects. In these systems, butenolide monomer 7i primarily acts as a plasticizer, leading to a pronounced decrease in *T*_g_ with increasing monomer 7i loading. Finally, the homopolymerisation of 7i was performed with a conversion of 56% and a relatively high *T*_g_ (142 °C).^159^

**Table 10:** Conversions obtained from the photopolymerisation of butenolides (B) (7i and 7j) and Elium® resin (E), and *T*_g_ obtained from the polymer pellets, obtained based on the corresponding formulations, as reported by Le Dot *et al.*^159^ in 2024. Phenyl bis(2,4,6-trimethylbenzoyl)phosphine oxide (BAPO) was used as photoinitiator (3 wt% of total mass). Polymerization experiments were conducted solventless, under air, at room temperature, and without any purification operation of the resin^159^

Entry	B	B : E [%]	*C*_B_ [%]	*C*_E_ [%]	*T*_g_ [°C]
1	7i	0 : 100	—	100	89
2	7i	25 : 75	28	100	31
3	7i	50 : 50	6	100	n.d.
4	7i	75 : 25	1	100	n.d.
5	7i	100 : 0	56	—	142
6	7j	25 : 75	100^a^	100	69
7	7j	50 : 50	100^a^	100	52
8	7j	75 : 25	100^a^	100	41
9	7j	100 : 0	100^a^	—	29

a The conversion was determined by the acrylate C=C bond and not *via* the butenolide C=C bond.

In contrast, switching to the acrylate/butenolide hybrid monomer 7j, full conversion was observed in all homo- and copolymerisations (entries 6–9). Notably, only the acrylate functionality participated in the photopolymerisation conditions, consistent with previous reports by Poskonin^307,308^ and Feringa.^323^ As a result, hybrid resins are formed through copolymerisation of MMA from *E* with the acrylate moiety of 7j, while the butenolide moiety remains pendant and acts as a flexible side chain, lowering the *T*_g_ with increasing monomer 7j content (entries 6–9). Although complete conversion is achieved with monomer 7j, the accompanying reduction in *T*_g_ limits its applicability in high-performance applications requiring thermal robustness. Despite this challenge, the study demonstrates 5-alkoxybutenolides can serve as viable bio-based modifiers for MMA-based resins, offering a promising pathway for integrating renewable content into advanced resin technologies.

Another method to prepare butenolide resins with low or negligible VOC emission involves polymerisation under waterborne conditions. This can be performed *via* conventional emulsion polymerisation with surfactants^103,324^ or *via* hybrid systems incorporating hydrophilic stabilizing polymer segments. In 2024, the Feringa group reported polyurethane (PU)-based hybrid dispersions for preparation of high-performance coatings containing with polybutenolides.^102^ For the preparation of the hybrid system, the process starts with the synthesis of a low-molecular weight PU from dimethylolpropionic acid (DMPA), poly(tetramethylene ether) glycol (*M*_n_ 1.0 kg mol^−1^) (PTMEG) and isophorone diisocyanate (IPDI) (Fig. 18). This low molecular weight PU is blended with the vinyl monomers (butenolide and comonomer) and emulsified by addition of a basic amine (triethylamine, TEA). Then, the PU chain is extended by a chain extender (ethylenediamine) to get high molecular weight PU. Finally, a radical initiator is added to the emulsion to polymerize the 5-substituted butenolide and comonomer inside the particles.^325^

**Fig. 18:**
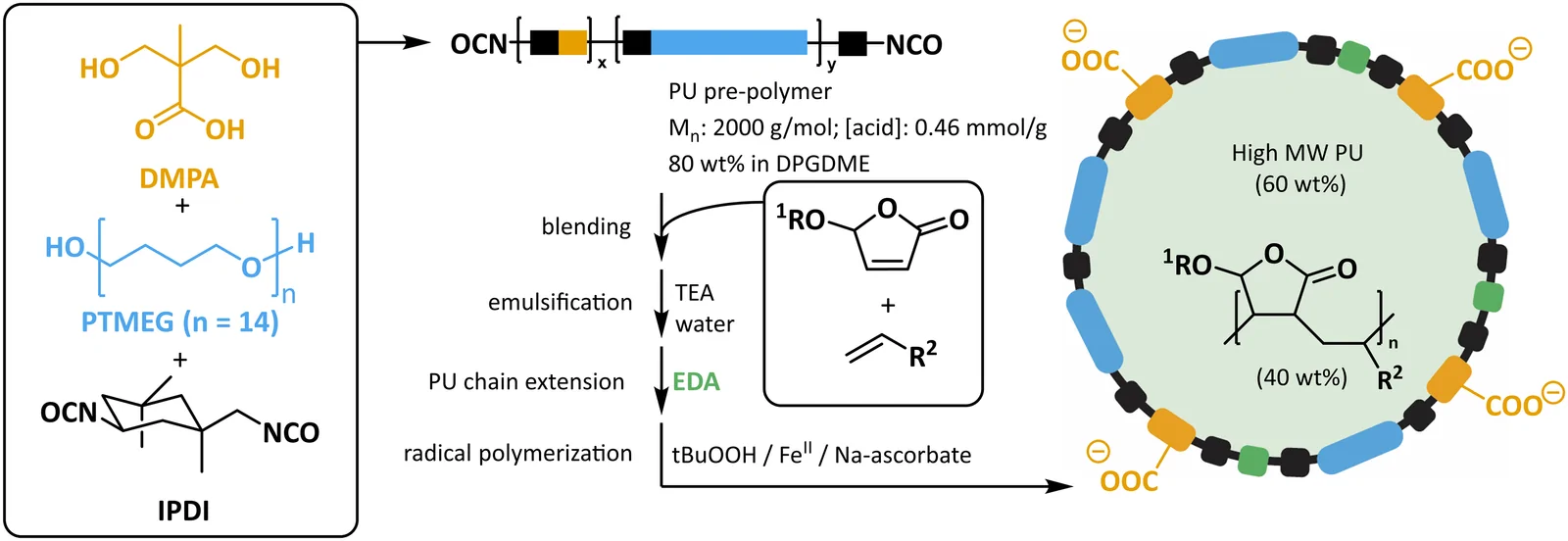
Methodology for producing aqueous polyurethane/polybutenolide hybrid dispersion. (1) Preparation of low-*M*_w_ polyurethane. (2) Blending with vinyl monomers. (3) Emulsification with triethylamine (TEA). 4) PU chain extension to get high *M*_w_ polyurethane. (5) Radical polymerisation using *t*-butyl hydroperoxide and redox couple (Fe^2+^ and sodium ascorbate) to form the final hybrid dispersions.^102,325^ Adapted from ref. 102 under the licence CC BY-NC 3.0 (RSC, © 2024).

A key requirement for obtaining stable dispersions was sufficient hydrophobicity of the butenolide monomer. Hydrophilic monomers, such as 5-methoxy- and 5-acetoxybutenolide, led to unstable dispersions, likely due to hydrolysis of the 5-substituted butenolide moiety. Similar results were obtained in the post-polymerisation release studies, where hydrophilic substituents were found to hydrolyse more readily than their hydrophobic counterparts.^309^

Coatings were prepared by casting the emulsion on a glass plate (Fig. 19). After drying, transparent, mechanically robust and hard coatings were obtained.^102^ By comparing the properties of these materials (Table 11) to a conventional poly-iBMA (isobutyl methacrylate) reference (entry 1) and a control lacking reactive monomer (entry 2), the effect of the poly(butenolide-*co*-butyl vinyl ether) phase could be evaluated. Here it was observed that the solvent resistance (MEK double-rub (dr) resistance) is slightly worse compared to the poly-iBMA reference for 5-alkoxybutenolide based systems (entries 1, 3 and 4). Switching to the 5-acyloxybutenolide (entry 5,5-hexanoyloxybutenolide) the MEK dr resistance was significantly improved. The Knoop hardnesses were also investigated and were generally similar to the iBMA reference, except for 5-hexyloxybutenolide (entry 4). In this case, the long alkyl chain imparts increasing flexibility, resulting in reduced hardness. Finally, two *T*_g_s in all systems, except for the PU only dispersion (entry 2), were measured, due to the fact of two distinct polymers being present in the hybrid system. The first *T*_g_ at room temperature is attributed to PU, while the second *T*_g_ corresponds to the polyacrylate or polybutenolide. Our group also investigated the H_2_O resistance of the coating, and it was evident that the polyacrylate or polybutenolide component is essential for good H_2_O resistance, as the pure PU coating from Entry 2 dissolved in H_2_O.

**Fig. 19:**
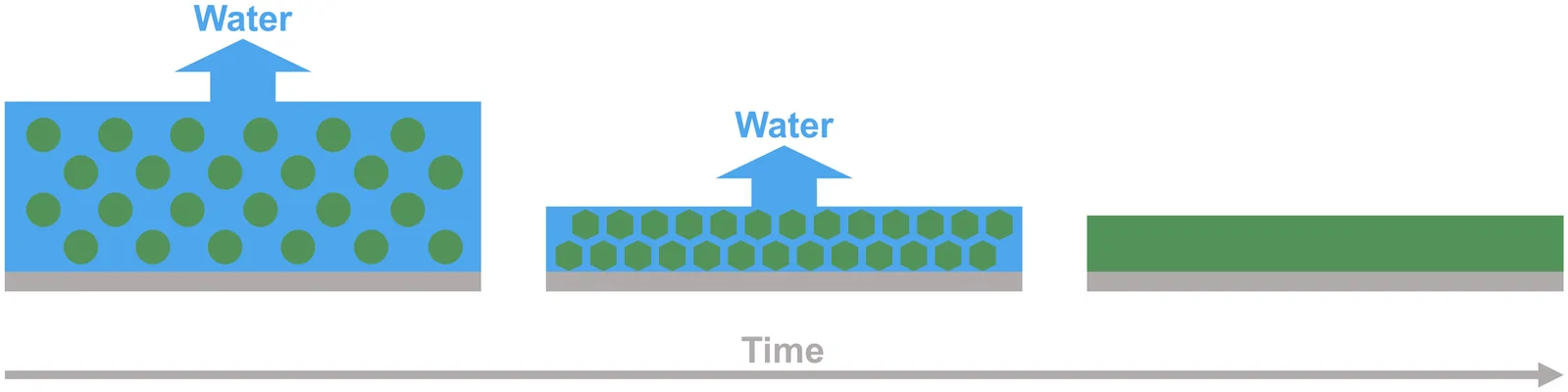
Preparation of waterborne coatings. Upon water evaporation, polymer particles deform and form a honey grate like structure in the final coating.^102^

**Table 11:** Coating properties, including Knoop Hardness (HK) and glass transition temperature (*T*_g_) of the aqueous polyurethane/polybutenolide hybrid dispersions as reported by Jensma *et al.* in 2024^102^

Entry	B	M2	MEK double rubs	HK [kg mm^−2^]	*T*_g_ [°C]	
					1st	2nd
1	iBMA	—	50	0.47	17	66
2	—	—	30	0.54	17	—
3	Butoxy-	BVE	40	0.38	22	47
4	Hexyloxy-	BVE	30	0.09	19	—
5	Hexanoyloxy-	BVE	110	0.78	20	51

In the past years, extensive research has been performed on utilising 5-alkoxy-2(5*H*)furanones and 5-acyloxy-2(5*H*)furanones in advanced resin technologies. Applying these monomers enables increased bio-based content while, in certain cases, maintaining or even improving material properties and featuring structural flexibility to allow tuning of properties. The resin technology has been examined in solventborne and waterborne systems, as well as 100% solid photo-curable systems. Direct applications of these resins have been found in the coatings industry, where significant progress has been achieved.

Overall, 5-alkoxy-2(5*H*)furanones and 5-acyloxy-2(5*H*)furanones (*i.e.* 5-substituted butenolides) emerge as versatile platform chemicals for the development of sustainable polymers, resins, and coatings, although further optimization is required to balance reactivity, stability, and thermomechanical performance.

## 6. Sustainability, reactivity, and toxicological profile of HFO and its derivatives

As outlined in the preceding sections, 5-Hydroxy-2(5*H*)-furanone (HFO, 5-hydroxybutenolide) is of particular interest among emerging bio-based molecular platforms. It can be produced from bio-based furanics, derivatised through various methodologies, and directed toward a wide range of applications.^102,107,308^ Having discussed the synthesis, downstream transformations, and emerging applications of HFO and its derivatives, it is now necessary to critically evaluate whether this molecule genuinely fulfils the criteria of a sustainable and “green” platform chemical, as we introduced in Section 1. Based on Sections 1 and 3, its renewable origin and synthetic versatility are well supported, but these properties alone are insufficient to constitute true sustainability. Therefore, the following section will evaluate HFO in the context of the 12 Principles of Green Chemistry.^2,4^ This does not by itself establish overall sustainability, which must ultimately be assessed through comprehensive life cycle considerations, but it provides a useful framework for evaluating the strengths and limitations of the HFO platform. The overarching objective is to assess the extent to which HFO can genuinely be regarded as a promising next-generation sustainable platform chemical.

### 6.1. Structural features, reactivity and synthetic versatility

At a broader conceptual level, HFO is best regarded as a multifunctional, bio-based, bottom-up platform chemical. As outlined in Section 1, the search for future sustainable feedstocks is no longer driven solely by the question of renewable carbon origin, but increasingly by the need for molecular platforms whose structures and reactivity patterns are not easily reproduced from conventional petrochemical building blocks.^43,99,326–330^ HFO fits this description exceptionally well. Its appeal lies not merely in the fact that it can be prepared from furfural and related biomass-derived furanics, but in the fact that it provides a rare example of a small, oxygen-rich, highly transformable scaffold. It combines an α,β-unsaturated lactone, a derivatizable hydroxy functionality at the C5 position, and a stereogenic centre (see Scheme 1). Additionally, facile tautomerization to its ring-opened form (*cis*-β-formylacrylic acid) further opens possibilities for further derivatization. In this sense, HFO is best understood not as an endpoint but as a hub molecule. Once available, it can be directed into multiple downstream value chains. This high degree of synthetic flexibility, constitutes one of its strongest arguments as a bio-based platform chemical.

This multidirectionality is also reflected in the breadth of transformations discussed in Sections 3 and 4. Hydrogenation and reductive aminolysis enable conversion to 5-hydroxy-γ-butyrolactone, γ-butyrolactone (GBL),^156^ 1,4-butanediol, tetrahydrofuran, γ-aminobutyric acid, 2-pyrrolidone, and N-methylpyrrolidone, often under milder or more selective conditions than conventional petrochemical routes.^99^ HFO therefore serves as a branching point toward several industrially relevant C4 value chain chemicals, including maleic anhydride (MAnh)/maleic acid (MA),^99,151,152^ succinic anhydride/succinic acid,^96^ BDO/THF/GBL,^99,156^ and pyrrolidone/GABA-type products.^99^

Especially the oxidation chemistry discussed in Section 3.1 illustrates the platform character of HFO and compatibility with contemporary approaches to greener synthesis particularly well. HFO can be transformed into MAnh and MA under relatively mild conditions using for example homogeneous aerobic catalysis,^151,152^ heterogeneous peroxide-based catalysis,^99^ enzymatic^153^ and electrocatalytic oxidations.^79,153,154^ Fe/TEMPO aerobic oxidation,^331^ batch-to-flow intensification studies,^119,120,332^ laccase–TEMPO systems, and ACT-mediated electrocatalytic protocols collectively demonstrate compatibility with several Principles of Green Chemistry, including the use of molecular oxygen, aqueous or otherwise mild conditions, catalysis by earth-abundant metals, biocatalysis, and flow processing. Although these studies do not establish overall sustainability, they demonstrate that HFO chemistry is well aligned with current strategies for process intensification and greener synthetic methodology.

The same structural features that make HFO attractive as a platform molecule for C4 building blocks, also explain its long-standing importance in asymmetric synthesis (Section 3).^190,195,197,207,333^ Chiral 5-substituted butenolides particularly 5-menthyloxybutenolides, were established as highly effective electrophilic building block in stereoselective synthesis.^49,53,54,189,194,334^ These compounds combine the reactivity of an electron-poor α,β-unsaturated lactone with conformational rigidity and, when appropriately substituted, effective stereochemical control. In this way, HFO-derived butenolides provide a chiral, functionalized analogue of the reactivity associated with MAnh, while granting access to stereochemically defined products. Pioneering studies by Feringa and coworkers demonstrated their broad utility in Diels–Alder reactions,^49,54^ 1,4-conjugate additions,^50,53,189,191,203,334^ and 1,3-dipolar cycloadditions,^194,195,197^ enabling efficient access to structurally complex and enantioenriched compounds. More recent developments have shifted away from stoichiometric chiral auxiliaries towards catalytic asymmetric methods for the direct construction of butenolide and butyrolactone frameworks, improving atom economy and synthetic efficiency.^98,162,212–214^

This synthetic utility also contributes to the broader sustainability argument surrounding HFO. As noted in Section 4, the butenolide motif is found in a wide range of biologically active natural products, and HFO-derived intermediates such as 5-menthyloxybutenolide and Boc-protected butenolides have enabled efficient and stereoselective routes to structurally complex targets.^53,98,162,191,203,204,212,213,252,253^ HFO is therefore attractive not only because it can be produced from renewable, biomass-derived furfural, but because it already contains oxygenation patterns, stereochemical information, and latent functional group relationships that are often difficult to install efficiently on simple petrochemical scaffolds. Molecules that intrinsically encode such synthetic and structural versatility may ultimately outperform simpler feedstocks, even if their selective preparation initially appears more challenging.

### 6.2. Radical polymerisation behaviour of HFO-derived monomers

The previously discussed tuneable reactivity is particularly evident in their conjugate addition chemistry and thus radical polymerisation behaviour. Acrylates remain the benchmark for highly activated Michael acceptors and reactive radical monomers, often requiring stabilizers to suppress uncontrolled polymerisation during storage and handling.^335^ Butenolides are likewise α,β-unsaturated carbonyl compounds and therefore retain electrophilic character, but their reactivity is qualitatively distinct and strongly substitution-dependent. In general, alkoxy substituents at the 5-position are weakly electron-donating and tend to reduce electrophilicity, whereas acyloxy groups are less donating, or more weakly withdrawing, and therefore increase activation relative to alkoxy analogues.^100^ This trend is consistent with observations in radical copolymerisation studies, where 5-acyloxybutenolides were introduced to enhance reactivity relative to less activated derivatives.^100^ It is therefore reasonable to expect 5-acyloxybutenolides to be more reactive in conjugate addition and radical copolymerisation contexts than their 5-alkoxy counterparts.^100^ Although direct quantitative comparison with acrylates remains context-dependent, these substituent effects highlight an important feature of the HFO platform: electrophilicity and polymerisation behaviour are tuneable rather than fixed.

Although butenolides are often compared with acrylates because of their structural similarity as α,β-unsaturated carbonyl compounds, their reactivity ratios and polymerisation kinetics differ substantially from those of classical acrylates. Importantly, these studies are not establishing butenolides as direct drop-in replacements for acrylates but instead defining them as a distinct reactivity class suitable for radical polymerisation chemistry.^307,308^ Homopolymerisation of butenolides is generally modest, while copolymerisation behaviour depends strongly on the electronic nature of the comonomer. Copolymerisation with common monomers such as methyl methacrylate (electron poor) and styrene (electron rich) improved overall conversion but often resulted in relatively low 5-alkoxybutenolide incorporation, whereas significantly higher incorporation was observed with more electron-rich comonomers such as vinyl acetate.^307,308^ It is important to clarify here that these observations with standard radical partners should not be interpreted as butenolides being inferior to conventional acrylates, but rather that their radical reactivity is more selective and more strongly partner-dependent.

Feringa and coworkers recently showed that copolymerisation with electron-rich comonomers such as VeoVa-10 and dodecyl vinyl ether gave substantially higher conversions than homopolymerisation.^100,107^ The thermal properties of the material could be tuned through both the identity of the comonomer and the nature of the substituent at the 5-position.^101,107^ Consequently, the value of butenolides in polymer chemistry lies not in reproducing acrylate behaviour as closely as possible, but rather in occupying an intermediate reactivity space between highly activated acrylates and more electron-deficient systems such as MAnh.^49^ In that sense, they are viable alternatives which are opening access to new monomer/comonomer design spaces rather than serving as one-to-one replacements for established acrylate monomers.

### 6.3. Sustainability assessment in the context of the 12 Principles of Green Chemistry

Despite this considerable synthetic versatility, multifunctionality alone is insufficient to establish HFO as a sustainable platform chemical. In this context, the 12 Principles of Green Chemistry provide an important framework for assessing the sustainability of HFO and its derivatives, including aspects such as atom economy, catalytic efficiency, energy demand, solvent selection, renewability of feedstocks, and the minimization of waste and hazardous substances.^1–4^ It is important to note however that these principles should not be interpreted as rigid criteria that a molecule either fulfils or fails. Rather, they provide a multidimensional framework through which the strengths, limitations, and development priorities of a chemical platform can be assessed. The following sections discuss the 12 Principles of Green Chemistry in the context of HFO and evaluate both the opportunities and current limitations of this platform molecule.

#### 6.3.1. Bio-based origin

Among the 12 Principles of Green Chemistry, Principle 7, the use of renewable feedstocks, stands out as particularly fundamental. In many respects, this principle provides the foundation upon which many of the other green chemistry principles build, as the sustainability of a chemical process is inherently influenced by the origin of its molecular framework. If the core structure is already bio-based and rooted in naturally occurring carbon cycles, the basis for more sustainable chemical design is established from the outset. In this regard, HFO chemistry aligns strongly with the central goals of sustainable chemistry, as HFOs are produced *via* oxidation of furfural, a biobased compound directly derived from lignocellulosic biomass through the dehydration of carbohydrate-based feedstocks (not interfering with the food chain). By replacing fossil-derived carbon with renewable biomass-derived carbon, HFO chemistry contributes to reduced dependence on petrochemical resources while simultaneously improving carbon circularity. Moreover, the oxygen-rich molecular architecture inherent to HFOs provides access to functionalities that are hard-to-synthesize from conventional hydrocarbon feedstocks, enabling novel sustainable materials and transformations. Thus, HFOs exemplify how renewable platform molecules can serve not merely as substitutes for fossil resources, but as enabling structures for the future development of sustainable chemical manufacturing.

#### 6.3.2. Prevention of waste, atom economy, catalysis, and energy efficiency

While the origin of a molecule is often regarded as a fundamental aspect of sustainability, the prevention of waste (Principle 1) remains one of the central Principles of Green Chemistry. This reflects the fact that avoiding waste formation is inherently more sustainable than subsequent treatment or remediation. In this context, HFO exhibits several favourable characteristics. Owing to its high functional density, HFO provides access to a broad range of downstream product classes without requiring extensive protecting-group strategies, repeated redox adjustments, or unnecessary functional-group interconversions. The molecule already contains multiple synthetically useful functionalities within a compact framework, including an α,β-unsaturated lactone and a derivatisable hydroxy group. As a result, fewer synthetic steps are required to access structurally complex targets. Such inherent structural organization can contribute to shorter synthetic sequences, lower reagent consumption, and reduced waste generation compared with routes starting from simpler petrochemical feedstocks.

These features are also closely connected to other green chemistry principles, including atom economy (Principle 2), design for energy efficiency (Principle 6), reduction of unnecessary derivatization (Principle 8), and catalysis (Principle 9). Many transformations to or from HFO retain a substantial part of the molecular framework, particularly in hydrogenation, oxidation, aminolysis, and polymerisation reactions. This limits unnecessary carbon loss and improves overall material efficiency. In addition, several reported synthetic approaches toward HFO and its derivatives employ catalytic rather than stoichiometric methodologies, including photocatalytic oxidation,^40,107^ aerobic oxidation,^117,151,152^ electrocatalytic oxidation^125,128^ and enzymatic catalysis.^93,94^ This not only ensures a high atom economy but also reduces the amount of potential hazardous reactants and byproducts. Additionally, flow-systems enable comparatively mild conditions, improving energy efficiency through enhanced heat and mass transfer as well as reduced reaction times.^107,118–120^

However, the sustainability of HFO-based methodologies should not be evaluated solely in terms of waste reduction and atom economy. Equal attention must be given to Principle 11: real-time analysis for pollution prevention. To fully realize the industrial potential of HFO chemistry, analytical methodologies capable of real-time, in-process monitoring and control must be further developed to prevent the formation of hazardous substances before they occur.^120^ The incorporation of spectroscopic process analysis, automated feedback control, and continuous monitoring systems offers significant opportunities to minimize waste formation, improve energy efficiency, and enhance operational safety. Although this area remains underdeveloped despite ongoing efforts, the compatibility of HFO chemistry with modern photochemical, electrochemical, and flow technologies provides a compelling foundation for future advances in sustainable process control, greener manufacturing, and pollution prevention.^120^

#### 6.3.3. Toxicology and safety

The principles of less hazardous chemical synthesis (Principle 3), designing safer chemicals (Principle 4), safer solvents and auxiliaries (Principle 5), and inherently safer chemistry for accident prevention (Principle 12) collectively emphasize that sustainable chemistry must extend beyond renewable feedstocks and synthetic efficiency alone. A genuinely sustainable platform chemical should ideally minimise hazardous reagents, reduce toxicological and environmental risks, operate under safer process conditions, and enable the deliberate design of products with controlled and application-specific reactivity profiles.

A significant proportion of reported HFO transformations already align these objectives. In particular, catalytic methodologies and continuous-flow processing offer clear advantages by avoiding the use of stoichiometric hazardous oxidants, thereby reducing hazardous reagent inventories while improving process efficiency and controllability.^93,118–120,122,152^ Flow chemistry further enhances operational safety through improved heat and mass transfer, reduced reactor volumes, and more precise process control. However, much of the current synthetic and polymer literature still relies on conventional organic solvents and chromatographic purification procedures typical of fine chemical synthesis,^100,107^ indicating that further optimisation towards greener solvent systems and intensified industrial processing is necessary.^101^ Nevertheless, initial steps in this direction are emerging, for example through the development of waterborne polymer systems, which reduce reliance on organic solvents and contribute to safer and more sustainable processing.^101,102^

The principle of designing safer chemicals is particularly applicable to butenolide systems, given that their reactivity can be systematically tuned through structural modification. In particular, substitution at the 5-position has a pronounced influence on reactivity. This tunability is highly relevant in applications such as polymer and surfactant chemistry, where molecular structure directly influences material properties and functional behaviour.

At the same time, the toxicological profile of butenolides remains incompletely characterized and must be interpreted with caution. Extensive data exist for halogenated furanones, especially 3-chloro-4-(dichloromethyl)-5-hydroxy-2(5*H*)-furanone (MX) and related chlorinated hydroxyfuranones,^81,336–394^ which have been shown to be strongly mutagenic in bacterial systems, genotoxic in mammalian cells, and carcinogenic in rodent models.^391^ These effects are generally attributed to the high electrophilicity of the halogenated systems, which enables direct covalent interaction with DNA, including the formation of nucleoside adducts, and is further associated with oxidative stress processes.^382,385,390,393,394^

However, the translation of this intrinsic reactivity into *in vivo* toxicity is not straightforward. Toxicokinetic factors, including metabolism and excretion, appear to limit systemic genotoxic effects,^390,391^ and available evidence suggests that responses may be tissue-specific and more pronounced in directly exposed organs such as the gastrointestinal tract.^393^

In contrast, no comparable toxicological dataset has been identified for non-halogenated HFO or its 5-alkoxy and 5-acyloxy derivatives, which are of primary relevance for asymmetric synthesis, polymer chemistry, and coating applications. Structurally, these non-halogenated butenolides retain an α,β-unsaturated lactone motif and are therefore capable of acting as Michael acceptors, suggesting the potential for covalent interaction with biological nucleophiles and possible irritation or sensitization effects. However, they lack the strongly activating halogen substituents that are characteristic of MX-type compounds, and it is therefore not appropriate to infer similar toxicological behaviour from halogenated analogues.

Accordingly, the toxicology of non-halogenated butenolides represents a genuine data gap. This interpretation is supported by inconsistencies in supplier safety data sheets, which frequently rely on precautionary classification methods and may assign different hazard categories to the same compound. Such discrepancies suggest that current classifications reflect structural alerts and conservative assumptions rather than comprehensive experimental datasets. Consequently, while safe handling practices remain essential, the toxicological behaviour of non-halogenated butenolides must be regarded as insufficiently characterized and in need of systematic study.

#### 6.3.4. Degradation and pollution prevention

Although Principle 1 and 11 emphasise that “it is better to prevent waste than to treat or clean up waste after it has been formed”^2^ sustainability considerations must also extend beyond process efficiency to include the long-term environmental persistence of chemical products. Principle 10 therefore stresses that chemical products should at the end of their function, degrade into harmless substances.^4^

In this context, the oxygen-rich structure of HFOs offers considerable promise. The presence of multiple oxygen-containing functionalities, including the lactone moiety, may increase susceptibility towards hydrolytic and biological degradation compared with highly persistent hydrocarbon-based materials.

A clear example is provided by the surfactant chemistry discussed in Section 4.^158^ HFO can be converted into bio-based anionic surfactants through acetalization with fatty alcohols followed by sulfonation.^158^ The resulting compounds were reported to exhibit detergency performances comparable to those of petroleum-derived analogues. More importantly, biodegradation data obtained according to OECD 301F showed that one family of HFO-derived surfactants reached 67–78% degradation after 28 days, compared with 60% for a conventional linear alkylbenzene sulfonate benchmark, whereas related linear sulfonated analogues performed significantly worse.^158^ This result demonstrates that high performance and enhanced environmental compatibility, including biodegradability, can coexist within HFO-based platforms. At the same time, such outcomes are strongly dependent on molecular architecture and cannot be guaranteed by renewable origin alone.

Despite these promising features, the available evidence remains limited. Most current studies focus on relatively low-molecular-weight surfactant systems, whereas comprehensive investigations into the degradation pathways, environmental persistence, and ecotoxicological profiles of HFO-derived polymers and advanced materials are still largely lacking.

### 6.4. Overall assessment of sustainability and life cycle

Taken together, the evaluation of HFO chemistry in the context of the 12 Principles of Green Chemistry indicates that HFO and its derivatives indeed represent highly promising sustainable platform chemicals. Their bio-based and renewable origin, high functional density, broad derivatization potential, and compatibility with (bio)catalytic, photo- and electrochemical, and flow-based methodologies align closely with the central goals of sustainable chemical manufacturing.^93,94,120,152,171^ At the same time, their multifunctional nature enables access to diverse application areas, including fine chemicals, surfactants, coatings, and polymer systems, thereby reflecting the emerging bottom-up platform concept discussed in Section 1. This combination distinguishes HFO from simpler petrochemical analogues and aligns well with the emerging paradigm of bottom-up sustainable chemical design.

The exceptional versatility of HFO renders the platform particularly attractive but also renders it difficult to predict a single dominant development trajectory. HFO-based systems are already expanding into a wide range of application fields, underscoring a key strength of the platform: its ability to connect renewable biomass-derived feedstocks with diverse, potentially high-value products.

At the same time, the future development of HFO chemistry will depend on a deeper understanding of its overall sustainability. Life cycle assessments (LCAs) provide a crucial framework for evaluating whether emerging bio-based platform chemicals can deliver genuine environmental benefits across their entire life cycle. Rather than focusing solely on renewable feedstock origin, LCAs assess impacts associated with raw material sourcing, chemical production, processing efficiency, product performance, and end-of-life behaviour.

In this context, a comprehensive, peer-reviewed LCA for HFO and its derivatives are notably lacking, and current sustainability claims therefore remain largely prospective, based primarily on feedstock origin and process-level considerations rather than fully quantified cradle-to-grave analyses. A rigorous assessment would need to account for biomass processing, oxidation to HFO, derivatisation steps, polymerisation and curing, and end-of-life scenarios. In particular, oxidation efficiency, purification requirements, solvent and catalyst use, and allocation across multiple downstream product pathways are likely to represent important environmental hotspots.^395–403^

From a platform-chemical perspective, the breadth of HFO is highly significant. A molecule that can feed multiple downstream product networks is intrinsically more attractive for sustainable development than one that serves only a single niche application, because the environmental and economic burdens of upstream production may be distributed across a broader portfolio of products.

At the same time, this very flexibility that makes HFO attractive as a platform chemical thus also complicates its sustainability and life cycle assessment, because system boundaries and allocation choices may differ substantially depending on whether the downstream target is a monomer, a surfactant, a fine chemical intermediate, or a solvent.

## 7. Scalability and industrial relevance

The scalability of HFO production is increasingly promising, particularly because several routes use furfural as the starting material. Furfural is an established biomass-derived platform chemical with a global market price of US$1200–2000 per t.^404^ The technical challenge for HFO production is achieving high selectivity while suppressing overoxidation to maleic acid and other degradation products. Therefore, the process economics will mostly depend on the catalyst selectivity and lifetime, substrate concentration, oxidant equivalents, reaction conversion, and product recovery rather than on the cost of the furfural feedstock.

### 7.1. Production pathways and scalability

The principal production routes are compared in Table 12 in terms of yield, reaction conditions, material requirements, isolation, demonstrated scale, and E-factor. This comparison highlights that the major distinction between the current approaches is not only reaction yield, but also their level of process development and scalability.

**Table 12:** Comparison of HFO production routes from furfural

	Carbon yield^a^ [%]	Oxidant equivalent	Solvent	Substrate Concentration [M]	Catalyst loading	Reaction temperature [°C]	Energy/light requirements	Isolation procedure	Demonstrated scale	E-Factor
Heterogeneous catalytic oxidation^99^	73.6	H_2_O_2_ 16 (3.1)^b^	H_2_O	0.2	104 wt%	Room temperature	Stirring	Extraction	100 mg (batch)	3.4
Electrochemical oxidation^128^	61.4	H_2_O solvent	H_2_O	0.07	20 wt%	Room temperature	N.A.^c^	Not isolated	50 mg (batch)	5.2
Photochemical oxidation^120^	68	O_2_ 1.2	Methanol	1	0.2 mol%	20	72 kWh day^−1^ LED power required	Crystallization	1.3 kg day^−1^ (continuous)	0.2

a Theoretical maximum of 80% as HFO is C4, while furfural is C5.

b Number in parentheses shows the amount of H_2_O_2_ used during the reaction.

c Current density at 1.9 V *vs*. Ag/Ag^+^ and actual power consumption not reported.

#### 7.1.1. Heterogeneous catalysis

Industrially, the heterogeneous catalytic oxidation of furfural could be the most attractive route for direct implementation using current technologies and infrastructure. Reaction yields of 92% have been reported using titanium silicate (TS-1) as a catalyst and H_2_O_2_ in water.^99^ Product isolation was achieved *via* extraction using an organic solvent (ethyl acetate). Technically, this process design is potentially compatible with conventional batch or continuous-flow reactor technologies, with the catalyst placed in a fixed-bed reactor, recovered, and recycled, while HFO could be isolated *via* liquid–liquid extraction. A by-product of this process is formic acid, which is itself a value-added chemical with a price of US$600–1000 per t for 95% grade material.^405^ After extraction of HFO into the organic phase, the formic acid remaining in the aqueous phase could be concentrated *via* distillation/dehydration to the desired purity. This additional separation and concentration step would, however, need to be taken into account in the overall process design and economics. For industrial implementation, however, the reported laboratory performance (1 mmol scale, 0.2 M) would need to be improved to achieve significantly higher space-time yields and substrate concentrations, while minimizing the oxidant requirement (16 equivalents of H_2_O_2_).

#### 7.1.2. Electrochemical oxidation

Electrochemical oxidation of furfural represents another promising option, especially with access to inexpensive renewable electricity. This route is particularly interesting since it allows for water to serve as a potential oxygen source. Recent studies have demonstrated high HFO selectivities and faradaic efficiencies using heterogeneous and metal-free electrodes. Zhu *et al.* reported faradaic efficiencies of 92.6% with an HFO selectivity of 96.4% and a furfural conversion of 79.6% over a mesoporous polydopamine polymer (*meso*-PDA).^128^ However, it should be noted that these values were obtained in laboratory settings without confirmation at a sufficiently large scale for industrial application. The reported electrochemical oxidations of furfural to HFO have been performed at small scales (7 mL electrolyte solution) and low furfural concentrations (0.07–0.1 M) over non-commercial/self-synthesized electrodes (1 cm × 1 cm) at 1.9 V *vs.* Ag/Ag^+^. While these initial results are promising, for electrochemical oxidation of furfural to HFO to become industrially feasible, the process needs to be demonstrated at a larger scale and at higher substrate concentrations. The catalyst stability and performance should be measured over longer periods of time than currently reported, preferably in continuous flow, while the isolation of HFO from the electrolyte solution also remains to be demonstrated. The biggest challenge is the overoxidation of HFO to MA at higher potentials, which limits the process to a narrow reaction window.

#### 7.1.3. Photochemical oxidation

Photochemical oxidation of furfural has so far been the only method reported to produce HFO in continuous-flow reactors with demonstrated kilogram scale-processing.^120^ Here, furfural reacts with singlet oxygen, generated by light irradiation in methanol/ethanol with methylene blue as an organic photosensitizer. In batch reactions, these transformations have reported quantitative yields of HFO. Using a large Taylor vortex flow reactor (185 mL irradiated volume), Edwards *et al.* reported an 85% yield of HFO while producing 1.3 kg day^−1^ using 15 × 200 W LEDs at 660 nm (*i.e.* 3 kW in total).^120^ Their furfural solution had a concentration of 1 M, with 2 mM methylene blue. Overoxidation of HFO to MA does not occur in the photochemical oxidation of furfural; however, condensation with the alcoholic solvents can yield the corresponding alkoxybutenolide as a by-product. Additionally, methyl formate is formed as a byproduct during HFO production, which is itself a value-added product with a price of US$1600 per t.^406^ Due to its low boiling point (32 °C), it would be possible to separate it by distillation, although this has not yet been demonstrated in the reported literature. Regarding scale and space-time yields, the photochemical oxidation of furfural to HFO is at a considerably more advanced stage than heterogeneous catalytic oxidation and electrochemical oxidation. This process has been demonstrated in continuous flow reactors, giving satisfactory yields at scales of kilograms per day. With the availability of renewable electricity, the increasing efficiency of LEDs, and the development of continuous photo-flow reactors, this production route has the potential to produce bulk quantities of HFO. Considering HFO as an intermediate, alkoxybutenolides can be obtained without the necessity of purifying the crude product from the photochemical reaction. Edwards *et al.* demonstrated subsequent alcohol oxidation of the HFO crude in a pressurized thermal flow reactor with a reactor volume of 3 mL.^120^ The reaction in superheated methanol at 200 °C afforded methoxybutenolide 7 d with an overall two-step yield of 67% and a projected productivity of 1.1 kg day^−1^. He *et al.* demonstrated that acyloxybutenolides can also be accessed continuously without the need for HFO isolation.^93^ Furoic acid was first converted to HFO by photooxidation over a self-synthesized heterogeneous MOF photocatalyst in a packed bed reactor in 2-MeTHF (0.5 M).^93^ The HFO solution was then passed over an immobilized lipase packed-bed reactor for subsequent enzymatic acylation, affording an overall yield of 92% for acetoxybutenolide 34b. Converting HFO to alkoxy- and acyloxybutenolides without intermediate purification presents an opportunity to improve industrial feasibility. While HFO can be isolated through crystallization as a white powder in high purity, most alkoxy- and acyloxybutenolides are liquids and can therefore be isolated *via* distillation.^100,107^

Taken together, the comparison in Table 12 shows that each production route presents a different techno-economic trade-off. Of the three pathways, photochemical oxidation of furfural has progressed beyond purely academic demonstrations, with Circolide Technologies B.V. producing butenolides from secondary biomass waste using light-driven processes.^407^ Supplier aggregators list dozens of other suppliers of HFO, predominantly in China, plus suppliers in the US, UK and Switzerland. Capot Chemical, JIAXING REGEN CHEMICAL CO. LTD and Zhengzhou Alfa Chemical Co. Ltd offer packages up to 100 kg, with prices available upon request.^408^ The cheapest prices for HFO we could identify were offered by Fluorochem (£401.00 for 5 g),^409^ Ambeed (US$480.00 for 5 g),^410^ Enamine (US$731 for 10 g),^411^ and BLD Pharmatech (€512.00 for 5 g).^412^

### 7.2. Techno-economic considerations and downstream markets

From a techno-economic perspective, the commercial value of HFO arises from its position as a versatile chemical intermediate between biomass-derived feedstocks and higher-value chemical products. Its distinct reactive functionalities allow it to serve as a platform for industrial C4 products *via* downstream oxidation, reduction, substitution, and amination reactions, providing producers with access to several product markets from a single intermediate. Table 13 summarizes the C4 chemicals obtainable *via* HFO and the current routes by which they are produced. The comparison illustrates the breadth of the HFO platform, spanning oxidation, hydrogenation, hydrolysis, and reductive amination products that are currently accessed through several distinct industrial feedstocks and processes. While several of these pathways lead to favourable E-factors, such transformations have only been demonstrated on small scales. With the current price of HFO and the limited scale at which HFO is available, competing with industrially established routes to C4 chemicals will take more time. However, as the companies mentioned earlier increase their production capacities and thereby reduce the price of HFO and other intermediates, this situation may change in the near future.

**Table 13:** C4 chemicals obtainable *via* HFO and their current chemical production routes

Product	Currently produced *via*	Production *via* HFO	*Y* [%]	*E*-factor
MA	Hydrolysis of MAnh, produced by oxidation of benzene/butane	Oxidation of HFO with H_2_O_2_	93^99^	5.9
MAnh	Oxidation of benzene/butane	Oxidation with O_2_ over catalytic TEMPO and iron catalyst	97^152^	0.1
GBL	Hydrogenation of MA or dehydrogenation of BDO	Hydrogenation over Ru/C	93^99^	0.1
THF	Dehydration of BDO	Hydrogenation over Ru–Re/C	64^99^	0.8
BDO	Reppe process or MAnh hydrogenation	Hydrogenation over Ru–Re/C	60^99^	0.7
GABA	Fermentation and enzymatic biotransformation	Reductive aminolysis over Ru/C	71^99^	4.7
2-pyrrolidone	Treating GABA with NH_3_	Reductive aminolysis over Ru/C	67^99^	6.1
SA	Hydrogenation of MA or oxidation of BDO	Hydrolysis in NaOH	Quantitative^96^	0

A particularly interesting opportunity arises in polymer, materials, and coating applications, with the coating market size valued at 82.8 billion in 2025, and projected to grow at a CAGR of 6.1% from 2026 to 2033.^413^ The coating industry is undergoing a gradual transition towards more sustainable raw materials, driven by a combination of climate targets, regulations, reduced reliance on fossil feedstocks, and customer/brand pressure. Since conventional coatings rely heavily on petrochemical-derived carbon, the coating industry is in need of bio-based building blocks that deliver comparable performance with a lower carbon footprint. For commercial adoption, bio-based building blocks must meet performance requirements comparable to those of conventional petrochemical alternatives. Bio-based coatings should retain adequate durability, adhesion, mechanical strength, chemical/UV resistance, suitable curing kinetics, drying behaviour, processability, and formulation compatibility. These requirements are particularly important since a reduction in coating lifetime could diminish the environmental benefit of using renewable raw materials.^414,415^ Alkoxy- and acyloxybutenolides have been demonstrated in academic studies to provide excellent and tuneable coating properties in UV-curable, solvent-borne, and water-borne formulations,^100–102,107,119,309^ and several related technologies have been patented by AkzoNobel.^103,318–320,324^ Their applicability across different coating technologies, combined with the possibility of tuning their chemical functionality, makes butenolides particularly interesting as bio-based building blocks for higher-value material applications. This also provides an opportunity for butenolides to compete not solely on renewable carbon content, but on the functional performance they can provide in coating formulations. While these markets are currently dominated by inexpensive acrylic binders and epoxy/polyurethane systems, access to bulk quantities of butenolides could make them competitive if the economics allow it.

HFO is an attractive building block for pharmaceuticals and fine chemicals with several reactive functionalities embedded within a compact scaffold, enabling diverse downstream transformations and access to structurally complex bioactive molecules. Menthyloxybutenolide has been extensively employed as a chiral synthon for the preparation of biologically active compounds. Its stereochemically defined menthyloxy substituent enables highly diastereoselective transformations, providing access to enantiomerically enriched building blocks for natural products and medicinal chemistry. Beyond their role as synthetic intermediates, functionalized butenolides have recently been demonstrated for direct pharmaceutical applications. Ma *et al.* reported the Pd-catalysed asymmetric allylic alkylation of Boc-protected butenolide with carboxylic acids of pharmaceutical, natural product, and peptide origin.^214^ The resulting chiral butenolidyl esters were demonstrated to function as chiral prodrug scaffolds and peptide-drug conjugation units. Together, these examples illustrate a potential value cascade from a renewable platform molecule (HFO) to complex, high-value products. From a techno-economic perspective, this product flexibility could be advantageous, since the same butenolide platform can serve multiple markets, while the higher value of chiral pharmaceutical intermediates and prodrug architectures could help offset the additional costs associated with catalyst use, stereoselective synthesis, and purification. This makes pharmaceutical and fine-chemical applications particularly relevant as potential early markets for HFO-derived butenolides, where product value may be more important than achieving the lowest possible feedstock cost.

Taken together, these downstream applications represent different commercialization opportunities for HFO, ranging from relatively high-volume C4 platform chemicals to higher-value materials and pharmaceutical applications. Table 14 summarizes their current development status and principal commercial rationale. The most appropriate market for HFO is therefore likely to depend strongly on production scale: higher-value pharmaceutical and specialty-material applications may tolerate higher HFO costs at smaller production volumes, whereas penetration into bulk C4 chemical or coating markets will require substantially lower production costs and larger-scale availability.

**Table 14:** Commercial status and relevance of downstream markets for HFO

Application	Commercial status/relevance	Rationale
Bio-based polymer/coating monomers	Emerging/commercially promising	Access to functional alkoxy- and acyloxybutenolides with demonstrated coating applications and patented technologies.
C4 platform chemicals	Laboratory-stage/emerging	Provides access to several established C4 chemicals through diverse downstream transformations, offering a potential bio-based alternative to conventional production routes.
Pharmaceuticals/fine-chemicals	Established synthetic intermediate/specialty scale	Versatile chiral building block for the synthesis of natural products, bioactive compounds, and other high-value pharmaceutical intermediates.

### 7.3. Positioning of HFO among bio-based platform chemicals

HFO is best viewed not as a direct competitor to established bio-based platform chemicals such as furfural, HMF, levulinic acid and succinic acid, but rather as a highly functionalized intermediate occupying a distinct region of the bio-based chemical landscape. As summarized in Table 15, these established platform chemicals generally combine mature production routes with broad downstream chemistry and recognized industrial applications. HFO, in contrast, derives much of its potential from the fact that conversion of the C5 platform chemical furfural generates a structurally distinct and highly functionalized C4 framework with different reactivity and downstream opportunities. It combines an α,β-unsaturated lactone, derivatizable hydroxy functionality and a stereocenter within a small molecule, providing multiple handles for selective functionalization and access to structurally diverse products.

**Table 15:** Benchmarking HFO against other bio-based platform chemicals

Bio-based platform chemical	Feedstock basis	Commercial maturity	Structural functionality	Downstream diversity	Stability/handling	Available sustainability evidence
Furfural	Lignocellulosic biomass	Large scale commercial production (300 kt per year)^416^	Furan aldehyde	Broad established C5 chemistry, furan derivatives, solvents, resins, fuels	Volatile liquid, can undergo oxidation/polymerization upon storage	LCA and TEA available
HMF	Lignocellulosic biomass, fructose, glucose	Commercial scale interest and production (300 t per year)^417^	Furan aldehyde + alcohol	Furan derivatives, FDCA/PEF precursor, polymers, specialty chemicals	Solid, thermally/chemically unstable	LCA and TEA available
Succinic acid	Lignocellulosic biomass, glucose, sucrose, glycerol	Established commercial market (74 kt per year)^418^	Difunctional carboxylic acid	Polyesters, solvents, specialty chemicals	Stable solid, easy to handle	LCA and TEA available
Levulinic acid	Lignocellulosic biomass, glucose	Commercial-scale interest and production (17.5 kt per year)^419^	Ketone + carboxylic acid	Solvents, resins, fuel additives, fine chemicals	Liquid, easy to handle, corrosive	LCA and TEA available
HFO	Furfural	Industrially emerging; limited commercial availability	α,β-unsaturated lactone, + hydroxy functionality + stereocenter	C4 platform chemicals, polymers/coatings, pharmaceuticals, surfactants	Solid, easy to handle	Bio-based feedstock; dedicated LCA and TEA lacking

This functionality has already enabled applications across several areas, including chiral building blocks for complex bioactive molecules, pharmaceutical and prodrug chemistry, C4 platform chemicals, polymerizable monomers, surfactants, and functional coatings. HFO may therefore be particularly attractive in applications where molecular complexity and selective derivatization provide greater value than production volume alone. Its position is consequently somewhat different from simpler and more established platform molecules, for which large-scale availability and mature downstream markets are major strengths.

Commercial maturity, however, extends well beyond the ability to produce a compound at tonne scale. Established platforms have accumulated well-developed feedstock and production routes, process and separation procedures, suppliers and infrastructure, standardized specifications, extensive downstream chemistry, demonstrated markets, TEA and LCA data, and customers familiar with their use. HFO can therefore be regarded as a chemically versatile but industrially emerging platform intermediate. Its principal challenges remain production scale, process economics, the lack of dedicated TEA and LCA studies, and the establishment of sufficiently large downstream markets.

Overall, the comparison in Table 15 suggests that the main opportunity for HFO lies not in replacing established platform chemicals, but in complementing them through its higher degree of structural complexity and functionalization. Whether this chemical versatility translates into a broader platform role will depend on the development of scalable production routes and applications in which these structural features provide a clear synthetic or performance advantage.

## 8. Conclusion

Overall, HFO and its 5-alkoxy- and 5-acyloxy derivatives are attractive not merely because they are derived from biomass, but because they exemplify what a next-generation bottom-up platform chemical can be. This combination distinguishes them from simpler petrochemical analogues and aligns well with the emerging paradigm of bottom-up sustainable chemical design.

Their branching into C4 chemicals, fine chemicals, surfactants, dyes, and polymers (*e.g.* coatings) provides economic and technological breadth. Additionally, derivatization chemistry enables deliberate modulation of solubility, stability, stereochemical value, polymerisation behaviour and material properties. At the same time, precisely because HFO and its derivatives are not simple drop-in analogues of established petrochemicals, they must be evaluated on their own terms. Their sustainable production, distinct reactivity, substitution-dependent behaviour, and multifunctional character are major strengths, but also complicate direct comparison.

From an industrial perspective, HFO should currently be regarded as a chemically versatile but still emerging platform chemical, for which scalable production, process economics, and market development remain key challenges. Higher-value pharmaceutical, fine-chemical, and specialty-material applications may provide attractive early opportunities, whereas penetration into bulk C4 chemical and coating markets will require substantially lower production costs and greater large-scale availability.

Alongside advances in production scale and process economics, a more complete assessment of the sustainability of HFO and its derivatives is still required. Future work should therefore focus on comprehensive life cycle assessment, systematic toxicological profiling, and evaluation of environmental fate. If these gaps are addressed, HFO has great potential as one of the leading bio-based platform chemicals. Its molecular complexity can be translated into both synthetic versatility and sustainable materials innovation, setting the stage for the chemistry of the future.

## Author contributions

T. F. and B. L. F. conceptualized the project and coordinated it. J. T. M., M. M. K., A. J., J. P. K., and T. F. conducted the literature review research and its validation. J. T. M. prepared the manuscript with input from M. M. K., A. J., J. P. K., and T. F. All authors reviewed the manuscript.

## Conflicts of interest

The authors declare no conflict of interest.

## Supplementary Material

CS-055-D6CS00842A-s001

## Data Availability

The data that support the findings of this study are available through the reference list and *via* the corresponding author upon reasonable request. The supplementary information provides equations, assumptions, and detailed calculations for *E*-factors and carbon yields associated with HFO production routes and its conversion into selected C4 platform chemicals. See DOI: https://doi.org/10.1039/d6cs00842a.

## References

[cit1] Anastas P. T. and Charles Warner J., Green Chemistry: Theory and Practice: Paperback: Paul Anastas, Oxford University Press, 1998

[cit2] Jessop P. G. , Trakhtenberg S. and Warner J., ACS Symposium Series, American Chemical Society, 2008, vol. 1000, pp. 401–436

[cit3] Warner J. C., Cannon A. S., Dye K. M. (2004). Environ. Impact Assess. Rev..

[cit4] Freese T. , Vidal J. L., George M. W., Warner J. C., Poliakoff M. and Feringa B. L., Green and Sustainable Chemistry for a Circular Economy, in The Sustainability of Science: How Science Impacts the Environment, and What Can Be Done, ed. M. Farley, Royal Society of Chemistry, 2026, pp. 75–148 10.1039/9781837672202-00075

[cit5] Freese T. , Farley M., Anastas P. T., Feringa B. L. and Lerch M. M., Chemrxiv, preprint, 2026 10.26434/chemrxiv.15000541/v1

[cit6] European Parliament, Greenhouse gas emissions by country and sector (infographic)|News|European Parliament, 2024

[cit7] Georges A. and Johansson S., European Environmental Bureau

[cit8] European Environmental Bureau, The problem of pharmaceutical pollution, https://eeb.org/en/the-problem-of-pharmaceutical-pollution/, (accessed 6 March 2026)

[cit9] European Commision and Directorate-General for Environment, Microplastics - Environment - European Commission, https://environment.ec.europa.eu/topics/plastics/microplastics_en, (accessed 6 March 2026)

[cit10] Directorate-General for Research and Innovation, Chemicals and advanced materials, https://research-and-innovation.ec.europa.eu/research-area/industrial-research-and-innovation/chemicals-and-advanced-materials_en, (accessed 22 April 2026)

[cit11] Takkellapati S., Li T., Gonzalez M. A. (2018). Clean Technol. Environ. Policy.

[cit12] Qiang G., Ansari M. F., Sun Z., Elangovan S. (2024). Adv. Synth. Catal..

[cit13] Burelo M., Hernández-Varela J. D., Medina D. I., Treviño-Quintanilla C. D. (2023). Heliyon.

[cit14] Lange J. P. (2021). Energy Environ. Sci..

[cit15] Plastics Europe AISBL, Plastics the Fast Facts, 2025, https://plasticseurope.org/knowledge-hub/plastics-the-fast-facts-2025/, (accessed 30 March 2026)

[cit16] Zhang Y., Xing E., Han W., Yang P., Zhang S., Liu S., Cao D., Li M. (2024). Engineering.

[cit17] Siracusa V., Blanco I. (2020). Polymer.

[cit18] Pothu R., Gundeboyina R., Boddula R., Perugopu V., Ma J. (2022). New J. Chem..

[cit19] Nabera A., Istrate I. R., Martín A. J., Pérez-Ramírez J., Guillén-Gosálbez G. (2023). Green Chem..

[cit20] Kumar J. A., Sathish S., Prabu D., Renita A. A., Saravanan A., Deivayanai V. C., Anish M., Jayaprabakar J., Baigenzhenov O., Hosseini-Bandegharaei A. (2023). Chemosphere.

[cit21] Muscat A., de Olde E. M., de Boer I. J. M., Ripoll-Bosch R. (2020). Glob. Food Sec..

[cit22] Deng W., Feng Y., Fu J., Guo H., Guo Y., Han B., Jiang Z., Kong L., Li C., Liu H., Nguyen P. T. T., Ren P., Wang F., Wang S., Wang Y., Wang Y., Wong S. S., Yan K., Yan N., Yang X., Zhang Y., Zhang Z., Zeng X., Zhou H. (2023). Green Energy Environ..

[cit23] Queneau Y., Han B. (2022). Innovation.

[cit24] Alam M. I., Ali M. A., Gupta S., Ali Haider M. (2017). Microb. Appl..

[cit25] Bao Y., Du Z., Liu X., Liu H., Tang J., Qin C., Liang C., Huang C., Yao S. (2024). Green Chem..

[cit26] Madhavan Nampoothiri K., Nair N. R., John R. P. (2010). Bioresour. Technol..

[cit27] Kumar V., Kumar P., Maity S. K., Agrawal D., Narisetty V., Jacob S., Kumar G., Bhatia S. K., Kumar D., Vivekanand V. (2024). Biotechnol. Biofuels Bioprod..

[cit28] Dutta S. (2023). Energy Fuels.

[cit29] Schroeter S. H., Appel R., Brammer R., Schenck G. O. (1966). Justus Liebigs Ann. Chem..

[cit30] Raquez J. M., Habibi Y., Murariu M., Dubois P. (2013). Prog. Polym. Sci..

[cit31] He W., Ye L., Coates P., Caton-Rose F., Zhao X. (2023). Int. J. Biol. Macromol..

[cit32] Bhagwat S. S., Li Y., Cortés-Peña Y. R., Brace E. C., Martin T. A., Zhao H., Guest J. S. (2021). ACS Sustainable Chem. Eng..

[cit33] Jiang X., Meng X., Xian M. (2009). Appl. Microbiol. Biotechnol..

[cit34] Bozell J. J., Moens L., Elliott D. C., Wang Y., Neuenscwander G. G., Fitzpatrick S. W., Bilski R. J., Jarnefeld J. L. (2000). Resour., Conserv. Recycl..

[cit35] Hayes G. C., Becer C. R. (2020). Polym. Chem..

[cit36] Ramchuran S. O., O’Brien F., Dube N., Ramdas V. (2023). Curr. Opin. Green Sustainable Chem..

[cit37] Mosquera M. E. G., Jiménez G., Tabernero V., Vinueza-Vaca J., García-Estrada C., Kosalková K., Sola-Landa A., Monje B., Acosta C., Alonso R., Valera M. Á. (2021). Sustainable Chem..

[cit38] Dunlop A. (1948). Ind. Eng. Chem..

[cit39] Hoydonckx H. E. , Van Rhijn W. M., Van Rhijn W., De Vos D. E. and Jacobs P. A., Ullmann’s Encyclopedia of Industrial Chemistry, 2007 10.1002/14356007.a12_119.pub2

[cit40] Feringa B. L. (1987). Recl. Trav. Chim. Pays-Bas.

[cit41] Feringa B. L., Butselaar R. J. (1981). Tetrahedron Lett..

[cit42] Feringa B. L., Butselaar R. J. (1982). Tetrahedron Lett..

[cit43] Jaswal A., Singh P. P., Mondal T. (2022). Green Chem..

[cit44] Umehara M., Cao M., Akiyama K., Akatsu T., Seto Y., Hanada A., Li W., Takeda-Kamiya N., Morimoto Y., Yamaguchi S. (2015). Plant Cell Physiol..

[cit45] Pepperman A. B. and Cutler H. G., Synthesis and Chemistry of Agrochemicals II, American Chemical Society, 1991, vol. 443, pp. 278–287

[cit46] Trost B. M., Burns A. C., Bartlett M. J., Tautz T., Weiss A. H. (2012). J. Am. Chem. Soc..

[cit47] Kitani S., Miyamoto K. T., Takamatsu S., Herawati E., Iguchi H., Nishitomi K., Uchida M., Nagamitsu T., Omura S., Ikeda H., Nihira T. (2011). Proc. Natl. Acad. Sci. U. S. A..

[cit48] Mares D. (1987). Mycopathologia.

[cit49] Feringa B. L., De Jong J. C. (1988). J. Org. Chem..

[cit50] Jansen J. F. G. A., Feringa B. L. (1992). Synth. Commun..

[cit51] Feringa B. L., De Lange B. (1988). Tetrahedron.

[cit52] Feringa B. L., de Lange B. (1988). Tetrahedron Lett..

[cit53] Jansen J. F. G. A., Jansen C., Feringa B. L. (1991). Tetrahedron: Asymmetry.

[cit54] De Jong J. C., van Bolhuis F., Feringa B. L. (1991). Tetrahedron: Asymmetry.

[cit55] de Lange B., van Bolhuis F., Feringa B. L. (1989). Tetrahedron.

[cit56] Feringa B. L., De Jong J. C. (1992). Bull. SOC. Chim. Belg.

[cit57] Hashem A., Kleinpeter E. (2001). Adv. Heterocycl. Chem..

[cit58] Avetisyan A. A., Dangyan M. T. (1977). Russ. Chem. Rev..

[cit59] Knight D. W. (1994). Contemp. Org. Synth..

[cit60] Javahershenas R., Nikzat S. (2023). RSC Adv..

[cit61] Tadiparthi K., Venkatesh S. (2022). J. Heterocycl. Chem..

[cit62] Mandal S., Thirupathi B. (2020). Org. Biomol. Chem..

[cit63] Princiotto S., Jayasinghe L., Dallavalle S. (2022). Bioorg. Chem..

[cit64] Ray Choudhury A., Mukherjee S. (2020). Chem. Soc. Rev..

[cit65] Flourat A. L., Haudrechy A., Allais F., Renault J. H. (2019). Org. Process Res. Dev..

[cit66] Renzetti A., Fukumoto K. (2019). Molecules.

[cit67] Rossi R., Lessi M., Manzini C., Marianetti G., Bellina F. (2017). Curr. Org. Chem..

[cit68] Jusseau X., Chabaud L., Guillou C. (2014). Tetrahedron.

[cit69] Zhang J., Das Sarma K., Curran T. T. (2013). Synlett.

[cit70] Gil S., Parra M., Rodriguez P., Segura J. (2009). Mini. Rev. Org. Chem..

[cit71] Yan L., Wu X., Liu H., Xie L., Jiang Z. (2013). Mini-Rev. Med. Chem..

[cit72] Badovskaya L. A., Povarova L. V. (2009). Chem. Heterocycl. Compd..

[cit73] Mao B., Fañanás-Mastral M., Feringa B. L. (2017). Chem. Rev..

[cit74] Seitz M., Reiser O. (2005). Curr. Opin. Chem. Biol..

[cit75] Romeo G., Iannazzo D., Piperno A., Romeo R., Corsaro A., Rescifina A., Chiacchio U. (2005). Mini. Rev. Org. Chem..

[cit76] Bellina F., Rossi R. (2007). Synthesis.

[cit77] De Souza M. V. (2005). Mini. Rev. Org. Chem..

[cit78] Jiang J., Tariq M. N. H., Popowycz F., Gu Y., Queneau Y. (2025). Beilstein J. Org. Chem..

[cit79] Badovskaya L. A., Poskonin V. V., Tyukhteneva Z. I., Kozhina N. D. (2021). Russ. J. Gen. Chem..

[cit80] Fedoseev S. V., Belikov M. Y. (2018). Chem. Heterocycl. Compd..

[cit81] Żurawska K., Byczek-Wyrostek A., Kasprzycka A., Walczak K. (2024). Molecules.

[cit82] Tokmajyan G. G., Karapetyan L. V. (2025). Chem. Heterocycl. Compd..

[cit83] Tokmajyan G. G., Karapetyan L. V. (2025). Chem. Heterocycl. Compd..

[cit84] Moutayakine A., Meninno S., Lattanzi A. (2025). ChemCatChem.

[cit85] Esser P., Pohlmann B., Scharf H.-D. (1994). Angew. Chem., Int. Ed. Engl..

[cit86] Ayoub N., Hamie Z., Toufaily J., Guénin E., Enderlin G. (2024). ACS Sustainable Chem. Eng..

[cit87] Oertling H., Reckziegel A., Surburg H., Bertram H. J. (2007). Chem. Rev..

[cit88] Rao Y. S. (1976). Chem. Rev..

[cit89] Rao Y. S. (1964). Chem. Rev..

[cit90] Garst J. E., Schmir G. L. (1974). J. Org. Chem..

[cit91] Yu X., Liu H., Wang Q., Jia W., Wang H., Li W., Zheng J., Sun Y., Tang X., Zeng X., Xu F., Lin L. (2021). ACS Sustainable Chem. Eng..

[cit92] Ciamician G., Silber P. (1913). Ber. Dtsch. Chem. Ges..

[cit93] He Y., Li N. (2026). Green Chem..

[cit94] Van Der Deen H., Cuiper A. D., Hof R. P., Van Oeveren A., Feringa B. L., Kellogg R. M. (1996). J. Am. Chem. Soc..

[cit95] Strizhov N. K., Poskonin V. V., Badovskaya L. A., Kupina E. P. (2002). Russ. J. Org. Chem..

[cit96] Poskonin V. V., Badovskaya L. A. (2003). Chem. Heterocycl. Compd..

[cit97] Brinksma J., Van Der Deen H., Van Oeveren A., Feringa B. L. (1998). J. Chem. Soc., Perkin Trans. 1.

[cit98] Trost B. M., Toste F. D. (1999). J. Am. Chem. Soc..

[cit99] Palai Y. N., Fukuoka A., Shrotri A. (2024). ACS Catal..

[cit100] Lepage M. L., Alachouzos G., Hermens J. G. H., Elders N., van den Berg K. J., Feringa B. L. (2023). J. Am. Chem. Soc..

[cit101] Hermens J. G. H., Freese T., Alachouzos G., Lepage M. L., van den Berg K. J., Elders N., Feringa B. L. (2022). Green Chem..

[cit102] Jensma A., Elders N., van den Berg K. J., Feringa B. L. (2024). Green Chemistry.

[cit103] Feringa B. L. , Hermens J. G. H., Van den Berg K. J. and Van Gemert R., WO 2021/084066 A1, 2021

[cit104] Higashibata N., Aoshima S., Kanazawa A. (2025). Polym. Chem..

[cit105] Doussot A., Bakaï M. F., Fouquet E., Hermange P. (2023). Org. Lett..

[cit106] Schenck G. O. (1953). Justus Liebigs Ann. Chem..

[cit107] Hermens J. G. H., Freese T., Van den Berg K. J., Van Gemert R., Feringa B. L. (2020). Sci. Adv..

[cit108] Salles A. G., Zarra S., Turner R. M., Nitschke J. R. (2013). J. Am. Chem. Soc..

[cit109] Koomen G.-J., Van rhijn J. B. M., Carels R., Pandit U. P. (1984). Heterocycles.

[cit110] Urakami H., Zhang K., Vilela F. (2013). Chem. Commun..

[cit111] White J. D., Carter J. P., Kezar H. S. I. I. I. (1982). J. Org. Chem..

[cit112] Astarita A., Cermola F., DellaGreca M., Iesce M. R., Previtera L., Rubino M. (2009). Green Chem..

[cit113] Chauhan D. K., Battula V. R., Giri A., Patra A., Kailasam K. (2022). Catal. Sci. Technol..

[cit114] Hosseini-Sarvari M., Rahimi M., Saki S. (2025). New J. Chem..

[cit115] Seo J. Y., Kim J. E., Je Kwon Y., Kim S. H., Cho S., Choi D. H., Cho K. Y., Baek K. Y. (2022). Mater. Lett..

[cit116] Fabregat V., Pagán J. M. (2025). Sustainability.

[cit117] Saki S., Hosseini-Sarvari M., Gu Y., Zhang T. (2024). Ind. Eng. Chem. Res..

[cit118] Sambiagio C., Noël T. (2020). Trends Chem..

[cit119] Hermens J. G. H., Lepage M. L., Kloekhorst A., Keller E., Bloem R., Meijer M., Feringa B. L. (2022). React. Chem. Eng.

[cit120] Edwards M. D., Pratley M. T., Gordon C. M., Teixeira R. I., Ali H., Mahmood I., Lester R., Love A., Hermens J. G. H., Freese T., Feringa B. L., Poliakoff M., George M. W. (2024). Org. Process Res. Dev..

[cit121] Clark C. A., Lee D. S., Pickering S. J., Poliakoff M., George M. W. (2016). Org. Process Res. Dev..

[cit122] Valverde D., Porcar R., Izquierdo D., Burguete M. I., Garcia-Verdugo E., Luis S. V. (2019). ChemSusChem.

[cit123] Lee E., Kim J. H., Choi J., Hong Y., Shin D., Yun H., Kim J., Bak G., Hong S., Hwang Y. J. (2023). J. Mater. Chem. A.

[cit124] Yuan X., Huang X., Hu M., Liu J., Guo W., Hao Y., Li Z., Zhang K., Han B. (2024). Green Chem..

[cit125] Wu H., Song J., Liu H., Xie Z., Xie C., Hu Y., Huang X., Hua M., Han B. (2019). Chem. Sci..

[cit126] Román A. M., Hasse J. C., Medlin J. W., Holewinski A. (2019). ACS Catal..

[cit127] Otero K., Armstrong C., Moreno-Piza O. J., Hollis E., Scalia J. C., De Jesús Báez L. R., Holewinski A., Hernández-Pagán E. A. (2025). Inorg. Chem..

[cit128] Zhu W., Yang R., Wang X., Dai K., Su C., Duan J., Chang C., Wu H. (2025). Chem. Commun..

[cit129] Badovskaya L. A., Poskonin V. V., Povarova L. V. (2017). Russ. Chem. Bull..

[cit130] Kumar P., Pandey R. K. (2000). Green Chem..

[cit131] Zhan J., Wu X., Qiu X., Li Z. (2025). ACS Appl. Mater. Interfaces.

[cit132] Chavan S. P., Kadam A. L. (2021). Results Chem..

[cit133] Ayoub N., Toufaily J., Guénin E., Enderlin G. (2022). Green Chem..

[cit134] Badovskaya L. A., Latashko V. M., Poskonin V. V., Grunskaya E. P., Tyukhteneva Z. I., Rudakova S. G., Pestunova S. A., Sarkisyan A. V. (2002). Chem. Heterocycl. Compd..

[cit135] Poskonin V. V. (2010). Chem. Heterocycl. Compd..

[cit136] Strizhov N. K., Poskonin V. V., Oganova O. A., Badovskaya L. A. (1999). Russ. J. Org. Chem..

[cit137] Poskonin V. V., Badovskaya L. A. (1991). Khim. Geterotsikl. Soedin..

[cit138] Grunskaya E. P., Badovskaya L. A., Poskonin V. V., Yakuba Yu. F. (1998). Chem. Heterocycl. Compd..

[cit139] Poskonin V. V., Badovskaya L. A. (1998). Chem. Heterocycl. Compd..

[cit140] Poskonin V. V. (2008). Chem. Heterocycl. Compd..

[cit141] Poskonin V. V., Badovskaya L. A., Povarova L. V. (2025). Russ. Chem. Bull..

[cit142] Poskonin V. V., Badovskaya L. A., Povarova L. V. (1998). Chem. Heterocycl. Compd..

[cit143] Poskonin V. V., Badovskaya L. A., Povarova L. V. (1999). Chem. Heterocycl. Compd..

[cit144] Badovskaya L. A., Poskonin V. V. (2015). Kinet. Catal..

[cit145] Wahlen J., Moens B., De Vos D. E., Alsters P. L., Jacobs P. A. (2004). Adv. Synth. Catal..

[cit146] Chavan S. P., Kadam A. L., Shinde S. S., Gonnade R. G. (2020). Chem. Asian J..

[cit147] Alonso-Fagúndez N., Agirrezabal-Telleria I., Arias P. L., Fierro J. L. G., Mariscal R., Granados M. L. (2014). RSC Adv..

[cit148] Diaz D., Arias D., de León J. N. D., Dongil A. B., Martínez-Quintana L., Escalona N., Pecchi G., Herrera C., Sepulveda C. (2026). Reactions.

[cit149] Boukouvalas J., Lachance N. (1997). Synlett.

[cit150] Wojcieszak R., Santarelli F., Paul S., Dumeignil F., Cavani F., Gonçalves R. V. (2015). Sustainable Chem. Processes.

[cit151] Hermens J. G. H., Jensma A., Feringa B. L. (2022). Angew. Chem., Int. Ed..

[cit152] Mortier J., Stevens C. V., Heugebaert T. S. A. (2025). Green Chem..

[cit153] Thiyagarajan S., Franciolus D., Bisselink R. J. M., Ewing T. A., Boeriu C. G., Van Haveren J. (2020). ACS Sustainable Chem. Eng..

[cit154] Lu G. H., Zong M. H., Li N. (2023). ACS Catal..

[cit155] Warthegau S. S., Salameh R. S., Meier S. (2026). ACS Sustainable Chem. Eng..

[cit156] Chen B., Li F., Yuan G. (2017). RSC Adv..

[cit157] Gassama A., Ernenwein C., Hoffmann N. (2009). ChemSusChem.

[cit158] Gassama A., Ernenwein C., Youssef A., Agach M., Riguet E., Marinković S., Estrine B., Hoffmann N. (2013). Green Chem..

[cit159] Le Dot M., Andrés Gomez Fernandez M., Langovist A., Charrière B., Gérard P., Dumur F., Hoffmann N., Lalevée J. (2023). Eur. Polym. J..

[cit160] Alguacil R., Fariña F., Martín M. V. (1996). Tetrahedron.

[cit161] Farina F., Martin M. V., Martin-Aranda R. M., De Guerenu A. M. (1993). Synth. Commun..

[cit162] Trost B. M., Toste F. D. (2003). J. Am. Chem. Soc..

[cit163] Morita Y., Tokuyama H., Fukuyama T. (2005). Org. Lett..

[cit164] Riguet E. (2011). J. Org. Chem..

[cit165] Bos M., Riguet E. (2014). J. Org. Chem..

[cit166] Yuste F. and Sánchez-Obregón R., 4-Hydroxy-2-butenolide. A Versatile Reagent for the Synthesis of Heterocyclic Compounds, 1982, vol. 47

[cit167] Yuste F., Vergel H., Barrios H., Ortíz B., Sánchez-Obregón R. (1988). Org. Prep. Proced. Int..

[cit168] Meyers A. I., Nolen R. L., Collington E. W., Narwid T. A., Strickland R. C. (1973). J. Org. Chem..

[cit169] Rodríguez S., Vidal A., Monroig J. J., González F. V. (2004). Tetrahedron Lett..

[cit170] Li M., Dong X., Zhang N., Jérôme F., Gu Y. (2019). Green Chemistry.

[cit171] Huang Y. M., Lu G. H., Zong M. H., Cui W. J., Li N. (2021). Green Chem..

[cit172] Shen R., Porco J. A. (2000). Org. Lett..

[cit173] Shen R., Lin C. T., Bowman E. J., Bowman B. J., Porco J. A. (2003). J. Am. Chem. Soc..

[cit174] Poskonin V. V., Badovskaya L. A., Butin A. V. (1997). Russ. J. Org. Chem..

[cit175] Lattmann E., Hoffmann H. M. R. (1996). ChemInform.

[cit176] Vasamsetty L., Khan F. A., Mehta G. (2013). Tetrahedron Lett..

[cit177] Desvals A., Fortino M., Lefebvre C., Rogier J., Michelin C., Alioui S., Rousset E., Pedone A., Lemercier G., Hoffmann N. (2022). New J. Chem..

[cit178] Farina F., Martin M. V., Paredes M. C. (1973). Synthesis.

[cit179] Cuiper A. D., Brzostowska M., Gawronski J. K., Smeets W. J. J., Spek A. L., Hiemstra H., Kellogg R. M., Feringa B. L. (1999). J. Org. Chem..

[cit180] Marinkovič S., Brulé C., Hoffmann N., Prost E., Nuzillard J. M., Bulach V. (2004). J. Org. Chem..

[cit181] Mulzer J., Riether D. (1999). Tetrahedron Lett..

[cit182] Pelter A., Ward R. S., Jones D. M., Maddocks P. (1992). Tetrahedron: Asymmetry.

[cit183] Pelter A., Ward R. S., Jones D. M., Maddocks P. (1990). Tetrahedron: Asymmetry.

[cit184] Kang F. A., Yin C. L., She S. W. (1996). J. Org. Chem..

[cit185] Kang F. A., Yu Z. Q., Yin H. Y., Yin C. L. (1997). Tetrahedron: Asymmetry.

[cit186] Hulst R., de Vries K. N., Feringa B. L. (2002). J. Org. Chem..

[cit187] Feringa B. L., de Lange B. (1988). Heterocycles.

[cit188] Jauch J. (2000). J. Org. Chem..

[cit189] Jansen J. F. G. A., Feringa B. L. (1990). Tetrahedron: Asymmetry.

[cit190] Lubben M., Feringa B. L. (1991). Tetrahedron: Asymmetry.

[cit191] Van Oeveren A., Jansen J. F. G. A., Feringa B. L. (1994). J. Org. Chem..

[cit192] Krief A., Lecomte P., Demoute J. P., Dumont W. (1990). Synthesis.

[cit193] Feringa B. L., de Lange B., Jansen J. F. G. A., de Jong J. C., Lubben M., Faber W., Schudde E. P. (1992). Pure Appl. Chem..

[cit194] Rispens M. T., Keller E., de Lange B., Zijlstra R. W. J., Feringa B. L. (1994). Tetrahedron: Asymmetry.

[cit195] de Lange B., Feringa B. L. (1988). Tetrahedron Lett..

[cit196] Feringa B. L., De Lange B., De Jong J. C. (2002). J. Org. Chem..

[cit197] Keller E., de Lange B., Rispens M. T., Feringa B. L. (1993). Tetrahedron.

[cit198] Hoffmann N. (1994). Tetrahedron: Asymmetry.

[cit199] Bertrand S., Glapski C., Hoffmann N., Pete J. P. (1999). Tetrahedron Lett..

[cit200] Jahjah R., Gassama A., Dumur F., Marinković S., Richert S., Landgraf S., Lebrun A., Cadiou C., Sellès P., Hoffmann N. (2011). J. Org. Chem..

[cit201] Bertrand S., Hoffmann N., Humbel S., Pete J. P. (2000). J. Org. Chem..

[cit202] Sundermann B., Scharf H. D. (1996). Tetrahedron: Asymmetry.

[cit203] Jansen J. F. G. A., Feringa B. L. (1991). Tetrahedron Lett..

[cit204] Middel O., Woerdenbag H. J., van Uden W., van Oeveren A., Jansen J. F. G. A., Feringa B. L., Konings A. W. T., Pras N., Kellogg R. M. (1995). J. Med. Chem..

[cit205] Pelter A., Ward R. S., Jones D. M., Maddocks P. (1993). J. Chem. Soc. Perkin 1.

[cit206] Pelter A., Ward R. S., Jones D. M., Maddocks P. (1993). J. Chem. Soc. Perkin 1.

[cit207] de Jong J. C., van den Berg K. J., van Leusen A. M., Feringa B. L. (1991). Tetrahedron Lett..

[cit208] Hoffmann N., Scharf H. D., Runsink J. (1989). Tetrahedron Lett..

[cit209] Bertrand S., Hoffmann N., Pete J. P. (1998). Tetrahedron.

[cit210] Lee N., Kim Y. W., Chang K., Kim K. H., Jew S. S., Kim D. K. (1996). Tetrahedron Lett..

[cit211] Ward R. S. (1995). Tetrahedron: Asymmetry.

[cit212] Trost B. M., Crawley M. L. (2002). J. Am. Chem. Soc..

[cit213] Trost B. M., Crawley M. L. (2004). Chem. – Eur. J..

[cit214] Ma Y., Ren W., Song C., Xu G., Kong D. (2025). Org. Lett..

[cit215] Valle-Amores M. A., Feberero C., Martin-Somer A., Díaz-Tendero S., Smith A. D., Fraile A., Alemán J. (2024). Organic Chemistry Frontiers.

[cit216] Hood D. K. , Kirk-Othmer Encyclopedia of Chemical Technology, 2024, pp. 1–51

[cit217] Lohbeck K. , Haferkorn H., Fuhrmann W. and Fedtke N., Ullmann's Encyclopedia of Industrial Chemistry, Wiley, 2000

[cit218] Zhu Y., Yang J., Mei F., Li X., Zhao C. (2022). Green Chem..

[cit219] Kumar P., Park H. A., Yuk Y., Kim H., Jang J., Pagolu R., Park S. A., Yeo C., Choi K. Y. (2024). Crit. Rev. Biotechnol..

[cit220] Heilmann M. , Rodewald D. and Weiguny J., Ullmann's Encyclopedia of Industrial Chemistry, Wiley-VCH Verlag GmbH & Co. KGaA, 2023, pp. 1–10

[cit221] Kanetaka J., Asano T., Masamune S. (1970). Ind. Eng. Chem..

[cit222] Karas L. and Piel W. J., Kirk-Othmer Encyclopedia of Chemical Technology, 2004

[cit223] Cornils B. and Lappe P., Ullmann's Encyclopedia of Industrial Chemistry, Wiley-VCH Verlag GmbH & Co. KGaA, 2014, pp. 1–18

[cit224] Worberg A. , in Kirk-Othmer Encyclopedia of Chemical Technology, John Wiley & Sons, Inc., 2025, pp. 1–20

[cit225] Bozell J. J., Petersen G. R. (2010). Green Chemistry.

[cit226] Werpy T. and Petersen G., Top Value Added Chemicals from Biomass Volume I-Results of Screening for Potential Candidates from Sugars and Synthesis Gas Produced by the Staff at Pacific Northwest National Laboratory (PNNL) National Renewable Energy Laboratory (NREL) Office of Biomass Program (EERE) For the Office of the Energy Efficiency and Renewable Energy

[cit227] Cornils B. and Lappe P., Ullmann's Encyclopedia of Industrial Chemistry, Wiley-VCH Verlag GmbH & Co. KGaA, 2014, pp. 1–18

[cit228] Gao X., Tong X., Zhang Y., Xue S. (2023). iScience.

[cit229] Harreus A. L. , Backes R., Eichler J.-O., Feuerhake R., Jäkel C., Mahn U., Pinkos R. and Vogelsang R., Ullmann's Encyclopedia of Industrial Chemistry, Wiley-VCH Verlag GmbH & Co. KGaA., 2011

[cit230] Heli Z., Hongyu C., Dapeng B., Yee Shin T., Yejun Z., Xi Z., Yingying W. (2022). Front. Nutr..

[cit231] Harreus A. L. , Backes R., Eichler J.-O., Feuerhake R., Jäkel C., Mahn U., Pinkos R. and Vogelsang R., Ullmann's Encyclopedia of Industrial Chemistry, Wiley, 2011

[cit232] Zeng J. C., Zhang X. P., Gao L., Yin Q. Q., Wang W. G. (2026). Nat. Prod. Bioprospect..

[cit233] Zhao H., Zhou Z., Feng F., Yuan W., Chen L., Naman C. B., Ju Z., Zhou Z., Lin W., He S., Ding L. (2025). Phytochemistry.

[cit234] Hirata A., Sumiyoshi M., Fujita H., Akimoto M., Padayao M. H. R. A., Eguchi Y., Matsuura M., Otsuka M., Inada K., Teshima A., Arakawa K. (2025). J. Antibiot. (Tokyo)..

[cit235] Aquino M., Bruno I., Riccio R., Gomez-Paloma L. (2006). Org. Lett..

[cit236] Ferreira L. C., de Souza Galaverna R., McBride T., Costa e Silva R., Browne D. L., Pastre J. C. (2025). React. Chem. Eng..

[cit237] Resende G. C. , Alvarenga E. S., Galindo J. C. G. and Macias F. A., Synthesis and Phytotoxicity of 4,5 Functionalized Tetrahydrofuran-2-ones, 2012, vol. 23

[cit238] Shen C., Guo R. T., Chen L., Li Y. Q., Chen J., Tang M. C. (2025). Org. Lett..

[cit239] Katta M., Li S. H., Huang D., Cheng Y. X. (2025). Bioorg. Med. Chem..

[cit240] Ghosh S., Chatterjee S., Nanda S. (2024). Org. Lett..

[cit241] Tang S. Y., Wang Y. P., Qin D. M., Zhang X. M., Liu X. J., Zi X. Q., Zhu S. Y., Pu H., Zhao Q. (2026). Bioorg. Med. Chem. Lett..

[cit242] Carter N. B., Nadany A. E., Sweeney J. B. (2002). ChemInform.

[cit243] DeGraw J. I. (1972). Tetrahedron.

[cit244] Vardanyan R. S. and Hruby V. J., Synthesis of Essential Drugs, Elsevier, Amsterdam, 2006, pp. 179–193

[cit245] Lusthaus J., Goldberg I. (2019). Med. J. Aust..

[cit246] Schmidt T., Heise N., Merzweiler K., Deigner H.-P., Al-Harrasi A., Csuk R. (2021). Molecules.

[cit247] Meyers A. I., Nolen R. L., Collington E. W., Narwid T. A., Strickland R. C. (1973). J. Org. Chem..

[cit248] Li F., Jiang T., Li Q., Ling X. (2017). Am. J. Cancer Res.

[cit249] Tamm C. , Mycotoxine in Human and Animal Health, Pathatox Publishers, Forest Park South, 1977, pp. 209–228

[cit250] Wadsworth Jr W. S. , Organic Reactions, 2005, pp. 73–253

[cit251] Zheng X.-Y., Zhang H.-L., Luo Q., Zhu J. (2011). Biomed Res. Int..

[cit252] Hoffmann N. (1994). Tetrahedron: Asymmetry.

[cit253] Huang Y., Minnaard A. J., Feringa B. L. (2012). Org. Biomol. Chem..

[cit254] Timple J. M. V., Magalhães L. G., Souza Rezende K. C., Pereira A. C., Cunha W. R., Andrade M. L., Silva E., Mortensen O. V., Fontana A. C. K. (2013). J. Nat. Prod..

[cit255] Marcotullio M. C., Pelosi A., Curini M. (2014). Molecules.

[cit256] Patel D. K., Patel K. (2022). Pharmacol. Res. – Mod. Chin. Med..

[cit257] Gielen M., Willem R., Dalil H., de Vos D., Kuiper C. M., Peters G. J. (1998). Met. Based. Drugs.

[cit258] Xiao Z., Li L., Li Y., Zhou W., Cheng J., Liu F., Zheng P., Zhang Y., Che Y. (2014). Cell Death Dis..

[cit259] Bhat V., Welin E. R., Guo X., Stoltz B. M. (2017). Chem. Rev..

[cit260] Shabeer S., Asad S., Jamal A., Ali A. (2022). Toxins.

[cit261] Benkerroum N. (2020). Int. J. Environ. Res. Public Health.

[cit262] Betina V. (1992). Folia Microbiol..

[cit263] De Vry J., Horváth E., Schreiber R. (2001). Eur. J. Pharmacol..

[cit264] Halliday H. (2008). J. Perinatol..

[cit265] Rosen M. J. and Kunjappu J. T., Surfactants and Interfacial Phenomena, John Wiley & Sons, Inc., 4th edn, 2012, pp. 1–38

[cit266] Neugebauer J. M. , in Methods in Enzymology, ed M. P. Deutscher, Academic Press, 1990, vol. 182, pp. 239–253 10.1016/0076-6879(90)82020-3 2314239

[cit267] Vecino X., Cruz J. M., Moldes A. B., Rodrigues L. R. (2017). Crit. Rev. Biotechnol..

[cit268] Hill R. L. (2008). Handbook of Detergents, Part E: Applications.

[cit269] Castro M. J. L., Ojeda C., Cirelli A. F. (2014). Environ. Chem. Lett..

[cit270] Lawrence M. J. (1994). Chem. Soc. Rev..

[cit271] Hellgren A.-C., Weissenborn P., Holmberg K. (1999). Prog. Org. Coat..

[cit272] Swarup S., Schoff C. K. (1993). Prog. Org. Coat..

[cit273] Scott M. J., Jones M. N. (2000). Biochim. Biophys. Acta, Biomembr..

[cit274] Patil H. V., Badgujar N. P., Suresh, Kulkarni R. D., Nagaraj K. (2025). Mater. Today Commun..

[cit275] Nagtode V. S., Cardoza C., Yasin H. K. A., Mali S. N., Tambe S. M., Roy P., Singh K., Goel A., Amin P. D., Thorat B. R., Cruz J. N., Pratap A. P. (2023). ACS Omega.

[cit276] Yue X., Queneau Y. (2022). ChemSusChem.

[cit277] OECD, Test No. 301: Ready Biodegradability, OECD Guidelines for the Testing of Chemicals, Paris, 1992

[cit278] Dey P., Dey P., Hoque M. B., Baria B., Rahman Md. M., Shovon S., Das D. (2025). Text. Res. J..

[cit279] Pizzicato B., Pacifico S., Cayuela D., Mijas G., Riba-Moliner M. (2023). Molecules.

[cit280] Geissler S. , in Handbook of natural colorants, ed T. Bechtold and R. Mussak, John Wiley & Sons, 2009, pp. 367–384

[cit281] Saska J., Li Z., Otsuki A. L., Wei J., Fettinger J. C., Mascal M. (2019). Angew. Chem..

[cit282] Franck J., Dymond E. G. (1926). Trans. Faraday Soc..

[cit283] Verhoeven J. W. (1996). Pure Appl. Chem..

[cit284] Nisticò R. (2020). Polym. Test..

[cit285] De With G. , Polymer Coatings, A Guide to Chemistry, Characterization, and Selected Applications, Wiley-VCH Verlag GmbH & Co. KGaA, 2018, pp. 1–17

[cit286] Kausar A. (2018). J. Macromol. Sci., Part A: Pure Appl. Chem..

[cit287] Dresel W. , Lubricants and lubrication, ed T. Mang and W. Dresel, John Wiley & Sons, 2nd edn, 2007, pp. 63–87

[cit288] Duncan R. (2003). Nat. Rev. Drug Discovery.

[cit289] Haag R., Kratz F. (2006). Angew. Chem., Int. Ed..

[cit290] Williams C. K., Hillmyer M. A. (2008). Polym. Rev..

[cit291] Veith C., Diot-Néant F., Miller S. A., Allais F. (2020). Polym. Chem..

[cit292] Satoh K. (2015). Polym. J..

[cit293] Fouilloux H., Thomas C. M. (2021). Macromol. Rapid Commun..

[cit294] Wang J.-S., Matyjaszewski K. (1995). J. Am. Chem. Soc..

[cit295] Kato M., Kamigaito M., Sawamoto M., Higashimura T. (1995). Macromolecules.

[cit296] Harrisson S., Whitfield R., Anastasaki A., Matyjaszewski K. (2025). Nature Reviews Methods Primers.

[cit297] Moad G., Rizzardo E., Thang S. H. (2008). Polymer.

[cit298] Rizzardo E. , Moad G. and Thang S. H., Encycl. Polym. Sci. Technol., 2009

[cit299] Szwarc M. (1956). Nature.

[cit300] Nicolas J., Guillaneuf Y., Lefay C., Bertin D., Gigmes D., Charleux B. (2013). Prog. Polym. Sci..

[cit301] Mosnáček J., Matyjaszewski K. (2008). Macromolecules.

[cit302] Moreno M., Goikoetxea M., de la Cal J. C., Barandiaran M. J. (2014). J. Polym. Sci. A Polym. Chem..

[cit303] Agarwal S. , Jin Q. and Maji S., Biobased Monomers, Polymers, and Materials, American Chemical Society, 2012, vol. 1105, pp. 197–212

[cit304] Mousa M., Bergenudd H., Kron A. L., Malmström E. (2021). Macromolecules.

[cit305] Sollka L., Lienkamp K. (2021). Macromol. Rapid Commun..

[cit306] Quintens G., Vrijsen J. H., Adriaensens P., Vanderzande D., Junkers T. (2019). Polym. Chem..

[cit307] Poskonin V. V., Badovskaya L. A., Seredinov M. V., Kovardakov V. A. (1997). Russ. J. Org. Chem..

[cit308] Poskonin V. V., Yakovlev D. N., Kovardakov V. A., Badovskaya L. A. (1999). Russ. J. Org. Chem..

[cit309] Jensma A., Jaeger J. T., Hermens J. G. H., Freese T., Meijer J. T., Tarozo R., Sneep J. L., Elders N., van den Berg K., Feringa B. L. (2026). Chem. – Eur. J..

[cit310] Nuyken O., Pask S. D. (2013). Polymers.

[cit311] Penczek S. , Kubisa P. and Matyjaszewski K., Cationic Ring-Opening Polymerization, Springer, 1st edn, 1985

[cit312] Ruckel E. R., Schuerch C. (1966). J. Org. Chem..

[cit313] Penczek S. (1979). Makromol. Chem..

[cit314] Andrews R. J. and Grulke E. A., in Polymer Handbook, ed J. Brandrup, E. H. Immergut and E. A. Grulke, Wiley & Sons Ltd, 4th edn, 1999, pp. 193–278

[cit315] Olabisi O. and Adewale K., Handbook of thermoplastics, CRC press, 2nd edn, 2016

[cit316] Guo Q. , Thermosets: structure, properties, and applications, Woodhead Publishing, 2017

[cit317] Hoevelmann C. H. , Niemeier M., Corten C., Garcia Castro I., Schaefer B., Lucas F., Schaefer C. G., Laerbusch M., Licht U. and Schumacher K.-H., EP 4 559 942 A1, 2025

[cit318] Lepage M. L. P. , Feringa B. L., Hermens J. G. H., Van den Berg K. J. and Elders N., WO 2024/126829 A1, 2024

[cit319] Feringa B. L. , Hermens J. G. H., Van den Berg K. J. and Van Gemert R., WO 2021/259819 A1, 2021

[cit320] Lepage M. L. P. , Feringa B. L., Hermens J. G. H., Van den Berg K. J. and Elders N., WO 2024/126832 A1, 2024

[cit321] Shukla V., Bajpai M., Singh D. K., Singh M., Shukla R. (2004). Pigm. Res. Technol..

[cit322] Moens E. K. C., De Smit K., Marien Y. W., Trigilio A. D., Van Steenberge P. H. M., Van Geem K. M., Dubois J.-L., D’hooge D. R. (2020). Polymers.

[cit323] Lepage M. L., Alachouzos G., Hermens J. G. H., Elders N., van den Berg K. J., Feringa B. L. (2023). J. Am. Chem. Soc..

[cit324] Lepage M. L. P. , Feringa B. L., Hermens J. G. H., Van den Berg K. J. and Elders N., WO 2024/126828 A1, 2024

[cit325] Mehravar S., Ballard N., Tomovska R., Asua J. M. (2019). Ind. Eng. Chem. Res..

[cit326] Kabbour M. and Luque R., Biomass, Biofuels, Biochemicals: Recent Advances in Development of Platform Chemicals, Elsevier Inc., 2019, pp. 283–297

[cit327] Sun Z., Fridrich B., De Santi A., Elangovan S., Barta K. (2018). Chem. Rev..

[cit328] Hu R., Zhan J., Zhao Y., Xu X., Luo G., Fan J., Clark J. H., Zhang S. (2023). Green Chemistry.

[cit329] Van Putten R. J., Van Der Waal J. C., De Jong E., Rasrendra C. B., Heeres H. J., De Vries J. G. (2013). Chem. Rev..

[cit330] Cai C. M., Zhang T., Kumar R., Wyman C. E. (2014). J. Chem. Technol. Biotechnol..

[cit331] Hermens J. G. H., Jensma A., Feringa B. L. (2022). Angew. Chem., Int. Ed..

[cit332] Freese T., Meijer J. T., Miola M., Pescarmona P. P., Feringa B. L. (2024). Sustainable Energy Fuels.

[cit333] de Jong J. C. , PhD thesis, Rijksuniversiteit Groningen, 1984

[cit334] Jansen J. F. G. A., Feringa B. L. (1989). Tetrahedron Lett..

[cit335] Kulkarni R. D., Chaudhari M. E., Mishra S. (2013). Pigm. Res. Technol..

[cit336] Kronberg L., Vartiaine T. (1988). Mutat. Res., Genet. Toxicol..

[cit337] LaLonde R. T., Bu L., Henwood A., Fiumano J., Zhang L. (1997). Chem. Res. Toxicol..

[cit338] Clark N. W. E., Chipman J. K. (1995). Toxicol. Lett..

[cit339] LaLonde R. T., Cook G. P., Perakyla H., Bu L. (1991). J. Chem. Soc., Perkin Trans. 1.

[cit340] Komulainen H., Kosma V. M., Vaittinen S. L., Vartiainen T., Kaliste-Korhonen E., Lötjönen S., Tuominen R. K., Tuomisto J. (1997). J. Natl. Cancer Inst..

[cit341] Vaittinen S. L., Komulainen H., Kosma V. M., Julkunen A., Mäki-Paakkanen J., Jansson K., Vartiainen T., Tuomisto J. (1995). Food Chem. Toxicol..

[cit342] Lalonde R. T., Cook G. P., Perakyla H., Dence C. W. (1991). Chem. Res. Toxicol.

[cit343] Komulainen H., Huuskonen H., Kosma V. M., Lötjönen S., Vartiainen T. (1994). Arch. Toxicol..

[cit344] Huuskonen H., Venäläinen R., Komulainen H. (2003). Birth Defects Res. B Dev. Reprod. Toxicol..

[cit345] Api A. M., Belsito D., Biserta S., Botelho D., Bruze M., Burton G. A., Buschmann J., Cancellieri M. A., Dagli M. L., Date M., Dekant W., Deodhar C., Fryer A. D., Gadhia S., Jones L., Joshi K., Lapczynski A., Lavelle M., Liebler D. C., Na M., O’brien D., Patel A., Penning T. M., Ritacco G., Rodriguez-Ropero F., Romine J., Sadekar N., Salvito D., Schultz T. W., Siddiqi F., Sipes I. G., Sullivan G., Thakkar Y., Tokura Y., Tsang S. (2020). Food Chem. Toxicol..

[cit346] Husain A., Khan S. A., Iram F., Iqbal M. A., Asif M. (2019). Eur. J. Med. Chem..

[cit347] Mills F. D., Mills F. D. (1978). J. Agric. Food Chem..

[cit348] Maki-Paakkanen J. , Mutation Research. Section on Environmental Mutagenesis and Related Subjects, 1992, vol. 271, pp. 174–174

[cit349] Ishiguro Y., Dence C. W., Lalonde R. T., Santodonato J. (1987). Environ. Toxicol. Chem.

[cit350] Lalonde R. T., Leo H. R. (1994). Chem. Res. Toxicol.

[cit351] Li X., Hiramoto K., Yoshida M., Kato T., Kikugawa K. (1998). Food Chem. Toxicol..

[cit352] LaLonde R. T., Perakyla H., Cook G. P., Dence C. W. (1990). Environ. Toxicol. Chem..

[cit353] Heiskanen K., Lindström-Seppö P., Haataja L., Vaittinen S. L., Vartiainen T., Komulainen H. (1995). Toxicology.

[cit354] Api A. M., Belsito D., Botelho D., Bruze M., Burton G. A., Cancellieri M. A., Chon H., Dagli M. L., Dekant W., Deodhar C., Fryer A. D., Jones L., Joshi K., Kumar M., Lapczynski A., Lavelle M., Lee I., Liebler D. C., Moustakas H., Muldoon J., Penning T. M., Ritacco G., Romine J., Sadekar N., Schultz T. W., Selechnik D., Siddiqi F., Sipes I. G., Sullivan G., Thakkar Y., Tokura Y. (2024). Food Chem. Toxicol..

[cit355] Melnick R. L., Boorman G. A., Dellarco V. (1997). J. Natl. Cancer Inst..

[cit356] Jansson K., Mäki-Paakkanen J., Vaittinen S. L., Vartiainen T., Komulainen H., Tuomisto J. (1993). Mutat. Res., Genet. Toxicol..

[cit357] Wright J. M., Schwartz J., Vartiainen T., Mäki-Paakkanen J., Altshul L., Harrington J. J., Dockery D. W. (2002). Environ. Health Perspect.

[cit358] Kronberg L., Holmbom B., Reunanen M., Tlkkanen L. (1988). Environ. Sci. Technol.

[cit359] Jansson K., Hyttinen J. M. T. (1994). Mutat. Res., Genet. Toxicol..

[cit360] Haataja L., Vartiainen T., Lampelo S., Lötjönen S., Tuomisto J. (1991). Toxicol. Lett..

[cit361] Meier J. R., Knohl R. B., Coleman W. E., Ringhand H. P., Munch J. W., Kaylor W. H., Streicher R. P., Kopfler F. C. (1987). Mutat. Res., Genet. Toxicol..

[cit362] Teramoto S., Takahashi K. L., Kikuta M., Kobayashi H. (1999). Congenit. Anom..

[cit363] Teramoto S., Shimizu N., Hojo H., Kobayashi H. (1999). Congenit. Anom..

[cit364] Risto L., Vartiainen T., Komulainen H. (1993). Toxicol. Lett..

[cit365] Mäki-Paakkanen J., Jansson K. (1995). Mutat. Res., Genet. Toxicol..

[cit366] Wang S., Tian D., Zheng W., Jiang S., Wang X., Andersen M. E., Zheng Y., He G., Qu W. (2013). Environ. Sci. Technol..

[cit367] Nishikawa A., Furukawa F., Lee I.-S., Kasahara K.-I., Tanakamaru Z.-Y., Nakamura H., Miyauchi M., Kinae N., Hirose M. (1999). Cancer Res..

[cit368] Hakulinen P., Mäki-Paakkanen J., Naarala J., Kronberg L., Komulainen H. (2004). Toxicol. Lett..

[cit369] Woodruff N. W., Durant J. L., Donhoffner L. L., Penman B. W., Crespi C. L. (2001). Mutat. Res., Genet. Toxicol. Environ. Mutagen..

[cit370] Holme J. A., Haddeland U., Haug K., Brunborg G. (1999). Mutat. Res., Genet. Toxicol. Environ. Mutagen..

[cit371] Nawrocki J., Andrzejewski P., Kronberg L., Jeleń H. (1997). J. Chromatogr. A.

[cit372] Hakulinen P., Yamamoto A., Koyama N., Kumita W., Yasui M., Honma M. (2011). Mutat. Res., Genet. Toxicol. Environ. Mutagen..

[cit373] Nunn J. W., Chipman J. K. (1994). Mutat. Res., Genet. Toxicol..

[cit374] Meier J. R., Monarca S., Patterson K. S., Villarini M., Daniel F. B., Moretti M., Pasquini R. (1996). Toxicology.

[cit375] Steffensen I. L., Paulsen J. E., Engeset D., Kronberg L., Alexander J. (1999). Pharmacol. Toxicol..

[cit376] Jansson K., Hyttinen J. M. T., Niittykoski M., Mäki-Paakkanen J. (1995). Environ. Mol. Mutagen..

[cit377] Laaksonen M., Mäki-Paakkanen J., Komulainen H. (2001). Arch. Toxicol..

[cit378] Hirose A., Nishikawa A., Kinae N., Hasegawa R. (1999). Rev. Environ. Health.

[cit379] Komulainen H., Tuominen R. K., Kosma V. M., Huuskonen H. (2000). Environ. Toxicol. Pharmacol..

[cit380] Dahlen T., Hauck T., Wein M., Schwab W. (2001). J. Biosci. Bioeng..

[cit381] Aquilina G., Bampidis V., De M., Bastos L., Guido Costa L., Flachowsky G., Gralak A., Hogstrand C., Leng L., López-Puente S., Martelli G., Mayo B., Ramos F., Renshaw D., Rychen G., Saarela M., Sejrsen K., Van Beelen P., Wallace R. J., Westendorf J. (2014). EFSA Journal.

[cit382] Yuan J., Liu H., Zhou L. H., Zou Y. L., Lu W. Q. (2006). Mutat. Res., Genet. Toxicol. Environ. Mutagen..

[cit383] Nishikawa A., Kinae N., Furukawa F., Mitsui M., Enami T., Hasegawa T., Takahashi M. (1994). Cancer Lett..

[cit384] Meier J. R., Blazak W. F., Knohl R. B. (1987). Environ. Mol. Mutagen..

[cit385] Munter T., Le Curieux F., Sjöholm R., Kronberg L. (1998). Chem. Res. Toxicol..

[cit386] Teramoto S., Takahashi K., Kikuta M., Kobayashi H. (1998). J. Toxicol. Environ. Health A.

[cit387] Ramos I., Lloveras M., Solans X., Huici A., Messeguer A. (2000). Environ. Toxicol. Chem.

[cit388] Api A. M., Bartlett A., Belsito D., Botelho D., Bruze M., Bryant-Friedrich A., Burton G. A., Cancellieri M. A., Chon H., Cronin M., Crotty S., Dagli M. L., Dekant W., Deodhar C., Farrell K., Fryer A. D., Jones L., Joshi K., Lapczynski A., Laskin D. L., Lavelle M., Lee I., Moustakas H., Muldoon J., Penning T. M., Piersma A. H., Ritacco G., Sadekar N., Schember I., Schultz T. W., Siddiqi F., Sipes I. G., Sullivan G., Thakkar Y. (2025). Food Chem. Toxicol..

[cit389] Komulainen H., Vaittinen S.-L., Vartiainen T., Lötjönen S., Paronen P., Tuomisto J. (1992). Pharmacol. Toxicol..

[cit390] Brunborg G., Holme J. A., Søderlund E. J., Hongslo J. K., Vartiainen T., Lötjönen S., Becher G. (1991). Mutat. Res., Genet. Toxicol..

[cit391] Mäki-Paakkanen J., Hakulinen P. (2008). Toxicology in Vitro.

[cit392] Munter T., Kronberg L., Sjöholm R. (1996). Chem. Res. Toxicol..

[cit393] Daniel F. B., Stober J. A., Olson G. R., Galloway S. M. (1991). Environ. Mol. Mutagen..

[cit394] Munter T., Le Curieux F., Sjöholm R., Kronberg L. (1999). Chem. Res. Toxicol..

[cit395] Domènech X., Ayllón J. A., Peral J., Rieradevall J. (2002). Environ. Sci. Technol..

[cit396] Hunt R. G., Franklin W. E. (1996). Int. J. Life Cycle Assess..

[cit397] Hellweg S., Benetto E., Huijbregts M. A. J., Verones F., Wood R. (2023). Nat. Rev. Earth Environ..

[cit398] Lankey R. L., Anastas P. T. (2002). Ind. Eng. Chem. Res..

[cit399] Finnveden G., Hauschild M. Z., Ekvall T., Guinée J., Heijungs R., Hellweg S., Koehler A., Pennington D., Suh S. (2009). J. Environ. Manage..

[cit400] Dreyer L. C., Hauschild M. Z., Schierbeck J. (2006). Int. J. Life Cycle Assess..

[cit401] Pennington D. W., Potting J., Finnveden G., Lindeijer E., Jolliet O., Rydberg T., Rebitzer G. (2004). Environ. Int..

[cit402] Curran M. A., Opin Curr (2013). Chem. Eng..

[cit403] Jessop P. G., MacDonald A. R. (2023). Green Chem..

[cit404] Ahmad N., Hosseini T., Zhang L. (2025). Chem. Eng. Res. Des..

[cit405] Chatterjee S., Dutta I., Lum Y., Lai Z., Huang K. W. (2021). Energy Environ. Sci..

[cit406] Spurgeon J. M., Theaker N., Phipps C. A., Uttarwar S. S., Grapperhaus C. A. (2022). ACS Sustainable Chem. Eng..

[cit407] Circolide | Green, high-performance building blocks for the future of materials, https://circolide.com/, (accessed 24 September 2026)

[cit408] 5-Hydroxy-2[5*H*]-furanone—Chemical Information Search, https://www.chemicalbook.com/ProductList_En.aspx?cbn=CB9435528, (accessed 24 September 2026)

[cit409] 5-HYDROXY-2,5-DIHYDROFURAN-2-ONE (CAS 14032-66-7), https://fluorochem.co.uk/product/F500261/, (accessed 24 September 2026)

[cit410] 14032-66-7 | 5-Hydroxyfuran-2(5*H*)-one | Alcohols | Ambeed.com, https://www.ambeed.com/products/14032-66-7.html, (accessed 24 September 2026)

[cit411] EnamineStore, https://enaminestore.com/catalog/EN300-94708, (accessed 24 September 2026)

[cit412] 14032-66-7|5-Hydroxyfuran-2(5*H*)-one|BLD Pharm, https://www.bldpharm.com/products/14032-66-7.html?BD=BD292906, (accessed 25 September 2026)

[cit413] Coatings Market Size, Share And Trends Report, 2026- 2033, https://www.grandviewresearch.com/industry-analysis/coatings-market-report, (accessed 24 September 2026)

[cit414] RoadToBio | Circular Bio-based Europe Joint Undertaking (CBE JU), https://www.cbe.europa.eu/projects/roadtobio, (accessed 24 September 2026)

[cit415] Roadmap for the Chemical Industry in Europe towards a Bioeconomy, https://cordis.europa.eu/project/id/745623/reporting, (accessed 24 September 2026)

[cit416] Annatelli M., Sanchez-Velandia J. E., Mazzi G., Pandeirada S. V., Giannakoudakis D., Rautiainen S., Esposito A., Thiyagarajan S., Richel A., Triantafyllidis K. S., Robert T., Guigo N., Sousa A. F., Garcia-Verdugo E., Arico F. (2024). Green Chemistry.

[cit417] Thoma C., Konnerth J., Sailer-Kronlachner W., Solt P., Rosenau T., van Herwijnen H. W. G. (2020). ChemSusChem.

[cit418] Biobased Succinic Acid Market Size, Share & Forecast 2026–2034, https://www.intellectualmarketinsights.com/report/biobased-succinic-acid-market/imi-008566, (accessed 22 September 2026)

[cit419] Árvai C., Medgyesi Z., Lui M. Y., Mika L. T. (2024). Adv. Synth. Catal..

