# Supplementary material for: 5-Hydroxy-2(5H)-furanone (HFO) – a versatile and bio-based platform chemical
Source: Chem Soc Rev. 2026 Oct 26. Online ahead of print. doi: 10.1039/d6cs00842a (PMC13704320; doi:10.1039/d6cs00842a)
Supplement: Supplementary file 1 [file CS-OLF-D6CS00842A-s001.pdf]

Supplementary Information (SI) for Chemical Society Reviews.
This journal is © The Royal Society of Chemistry

# Supporting information

## 5-Hydroxy-2(5H)-furanone (HFO) – a versatile and bio-based platform chemical

*Jelmer T. Meijer[a], Magdalena M. Kleybolte[a], Andries Jensma[a], Jeya P. Kaniraj[b], Thomas Freese*[a,b], Ben L. Feringa*[a]*

**Contents**

1. Equations for E-factor and carbon yield ........ 2
   1.1 E-factors ........ 2
   1.2 Carbon yield ........ 2
2. Heterogeneous catalytic oxidation ........ 3
3. Electrochemical oxidation ........ 4
4. Photochemical oxidation ........ 5
5. Maleic acid (MA) ........ 6
6. Maleic anhydride (MAnh) ........ 7
7. Gamma butyrolactone (GBL) ........ 8
8. THF ........ 9
9. 1,4-butanediol (BDO) ........ 10
10. γ-aminobutyric acid (GABA) ........ 11
11. 2-pyrrolidone ........ 12
12. Succinic acid (SA) ........ 13
13. References ........ 14

## 1. Equations for E-factor and carbon yield

### 1.1 E-factors

Calculations were performed based on the reported reaction yields. Solvents and water were not included in the E-factor calculations because no reliable solvent recycling data were available.

$$E\ Factor = \frac{mass\ waste}{mass\ products}$$

### 1.2 Carbon yield

The reaction has a theoretical maximum carbon yield of 80%, as HFO contains four carbon atoms, whereas furfural contains five.

$$Carbon\ yield\ (\%) = Molar\ yield\ of\ HFO(\%) * 0.8$$

## 2. Heterogeneous catalytic oxidation

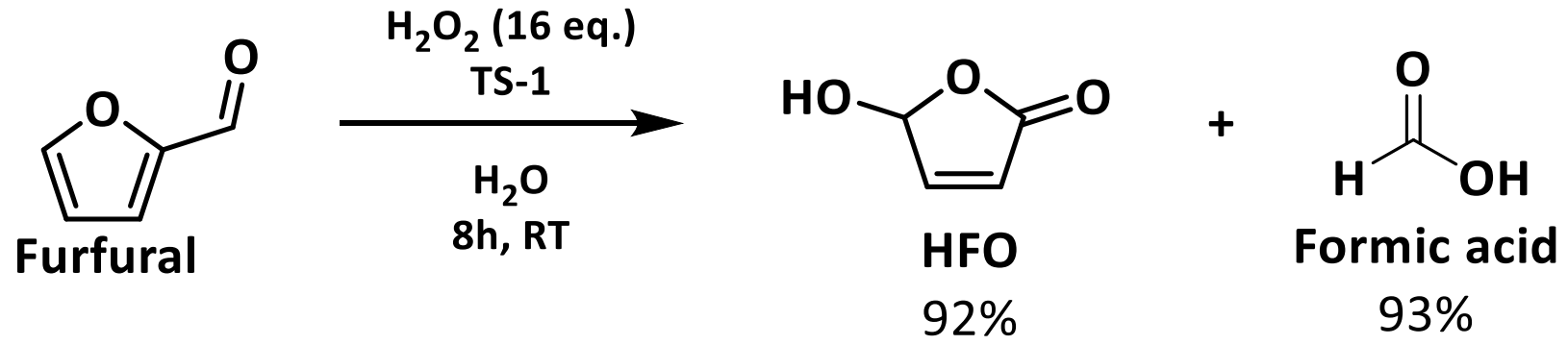


**Scheme 1:** Heterogeneous catalytic oxidation of furfural over TS-1 catalyst, using $H_2O_2$ as oxidant.[1]

| | |
|---|---|
| M(HFO): | 100.07 g/mol |
| M(Formic acid): | 46.03 g/mol |
| M($H_2O_2$): | 34.014 g/mol |
| M(MA): | 116.07 g/mol |
| M(Furfural): | 96.08 g/mol |

Heterogeneous catalytic oxidation of 1 mmol of furfural was reported over TS-1, using $H_2O_2$ as oxidant.[1] Complete conversion of furfural was achieved, affording HFO in 92% yield and maleic acid (MA) in 5% yield, while formic acid (93%) was formed as a by-product. A total of 16 mmol of $H_2O_2$ was added to the reaction, of which 3.1 mmol was consumed. For the E-factor calculation, only HFO was considered the desired product, while formic acid was regarded as a value-added by-product. MA was treated as a non-product stream resulting from the undesired overoxidation of HFO. In addition, the 8% unreacted furfural was regarded as waste in the E-factor calculation.

**Table 1:** Mass of products and waste generated in the heterogeneous catalytic oxidation of furfural to HFO.[1]

| Products | |
|---|---|
| 0.92 mmol HFO | 0.092 g |
| 0.93 mmol formic acid | 0.043 g |
| **Waste** | |
| 12.9 mmol $H_2O_2$ | 0.449 g |
| 0.05 mmol MA | 0.006 g |
| 0.08 mmol furfural | 0.008 g |

$$E\ Factor = \frac{mass\ waste}{mass\ products} = \frac{0.449 + 0.006 + 0.008}{0.092 + 0.043} = 3.4$$

$$Carbon\ yield\ (\%) = 92\% * 0.8 = 73.6\%$$

## 3. Electrochemical oxidation

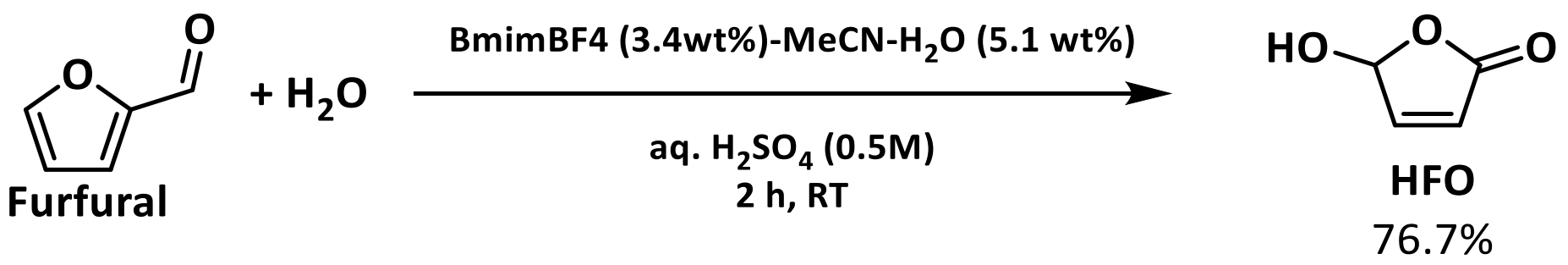


**Scheme 2:** Electrochemical oxidation of furfural over Meso-PDA.[2]

M(HFO): 100.07 g/mol

M(Furfural): 96.08 g/mol

3.4 wt% $BmimBF_4$ in MeCN gives 0.188 g $BmimBF_4$

Electrochemical oxidation of 0.5 mmol of furfural over spherical mesoporous polymer Meso-PDA afforded HFO at 76.7% conversion, corresponding to 0.384 mmol of HFO.[2] The anolyte consisted of $BmimBF_4$ (3.4 wt%), MeCN, and $H_2O$ (5.1 wt%). As the recovery and recycling of the $BmimBF_4$ electrolyte were not demonstrated, it was considered waste and was therefore included in the E-factor calculation. Only HFO was considered the desired product.

**Table 2:** Mass of products and waste generated in the electrochemical oxidation of furfural to HFO.[2]

| Products | |
|---|---|
| 0.384 mmol HFO | 0.038 g |
| **Waste** | |
| BMIMBF4 | 0.188 g |
| 0.116 mmol Furfural | 0.011 g |

$$E\ Factor = \frac{mass\ waste}{mass\ products} = \frac{0.188 + 0.011}{0.038} = 5.2$$

$$Carbon\ yield\ (\%) = 76.7 * 0.8 = 61.4$$

## 4. Photochemical oxidation

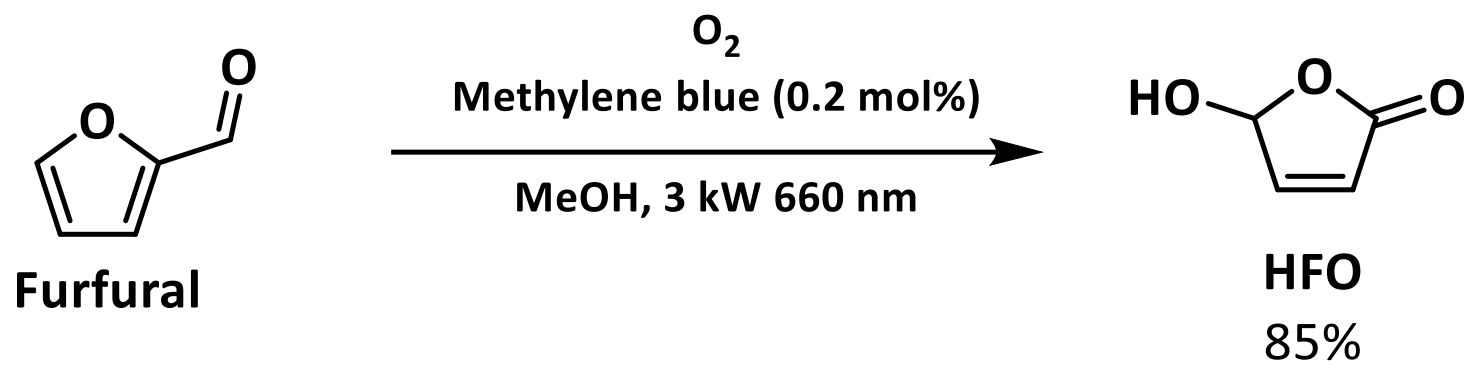


**Scheme 3:** Photochemical oxidation of furfural in a continuous photo-flow reactor.[3]

| | |
|---|---|
| M(HFO): | 100.07 g/mol |
| M(methyl formate): | 60.052 g/mol |
| M(Furfural): | 96.08 g/mol |
| M(methylene blue): | 319.85 g/mol |
| M($O_2$): | 32.00 g/mol |

Continuous-flow photooxidation of furfural was reported to achieve complete furfural conversion, affording HFO in 85% yield.[3] Accordingly, 1 mol of furfural produces 0.85 mol of HFO, while methyl formate is formed stoichiometrically alongside HFO as a value-added product. Methylene blue was employed as a photosensitizer at a loading of 0.2 mol% and was not reported to be recovered or recycled; it was therefore considered waste in the E-factor calculation. The remaining 15% of converted furfural that did not contribute to HFO formation was likewise considered waste. Accordingly, both the non-recovered photosensitizer and the unproductive fraction of furfural were included as waste streams in the E-factor calculation. A total of 1.2 equivalents of $O_2$ was supplied to the reactor, of which 0.85 equivalents were consumed in the formation of HFO. Therefore, the remaining 0.35 mol of $O_2$ was included as waste in the E-factor calculation.

**Table 3:** Mass of products and waste generated in the photochemical oxidation of furfural to HFO.[3]

| **Products** | |
|---|---|
| 0.85 mol HFO | 85.06 g |
| 0.85 mol methyl formate | 51.04 g |
| **Waste** | |
| 0.15 mol furfural | 14.41 g |
| 0.002 mol methylene blue | 0.64 g |
| 0.35 mol $O_2$ | 11.2 g |

$$E\ Factor = \frac{mass\ waste}{mass\ products} = \frac{14.41 + 0.64 + 11.2}{85.06 + 51.04} = 0.19$$

$$Carbon\ yield\ (\%) = 85\% * 0.8 = 68\%$$

## 5. Maleic acid (MA)

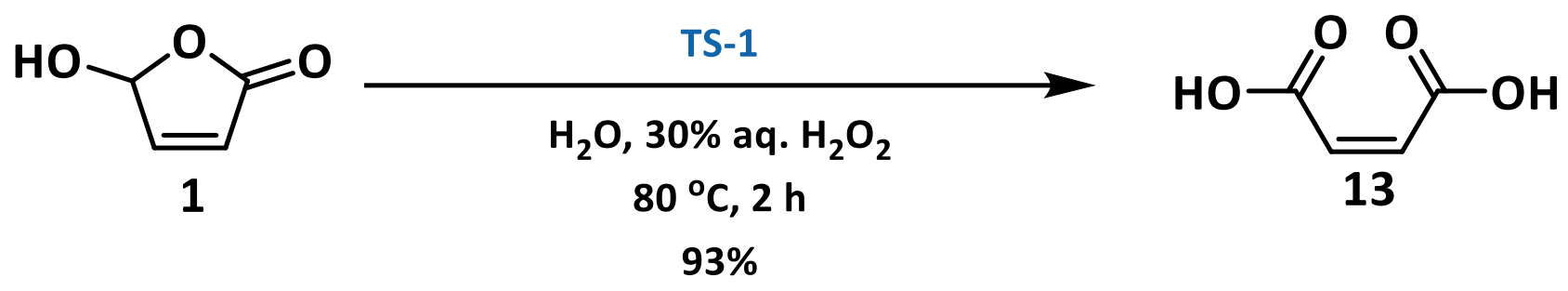

**Scheme 4:** Oxidation of HFO with TS-1 catalyst, using $H_2O_2$ as oxidant.[1]

M(HFO): 100.07 g/mol

M($H_2O_2$): 34.014 g/mol

M(MA): 116.07 g/mol

2 mL 30 % $H_2O_2$ gives 19.6 mmol $H_2O_2$.

Oxidation of 1 mmol of HFO was reported to afford maleic acid (MA) in 93% yield.[1] The remaining 7% of HFO was reported to form unidentified products and was therefore considered an unproductive fraction and included as waste in the E-factor calculation. An excess of 19.6 mmol of $H_2O_2$ was used in the reaction. Assuming a stoichiometric requirement of 1 mmol of $H_2O_2$ per mmol of HFO for conversion to MA, 1.0 mmol of $H_2O_2$ was considered consumed, while the remaining 18.6 mmol was included as waste in the E-factor calculation.

**Table 4:** Mass of products and waste generated in the oxidation of HFO to MA.[1]

| Products | |
|---|---|
| 0.93 mmol MA | 0.108 g |
| **Waste** | |
| 0.07 mmol HFO | 0.007 g |
| 18.6 mmol $H_2O_2$ | 0.633 g |

$$E\ Factor = \frac{mass\ waste}{mass\ products} = \frac{0.633 + 0.007}{0.108} = 5.9$$

## 6. Maleic anhydride (MAnh)

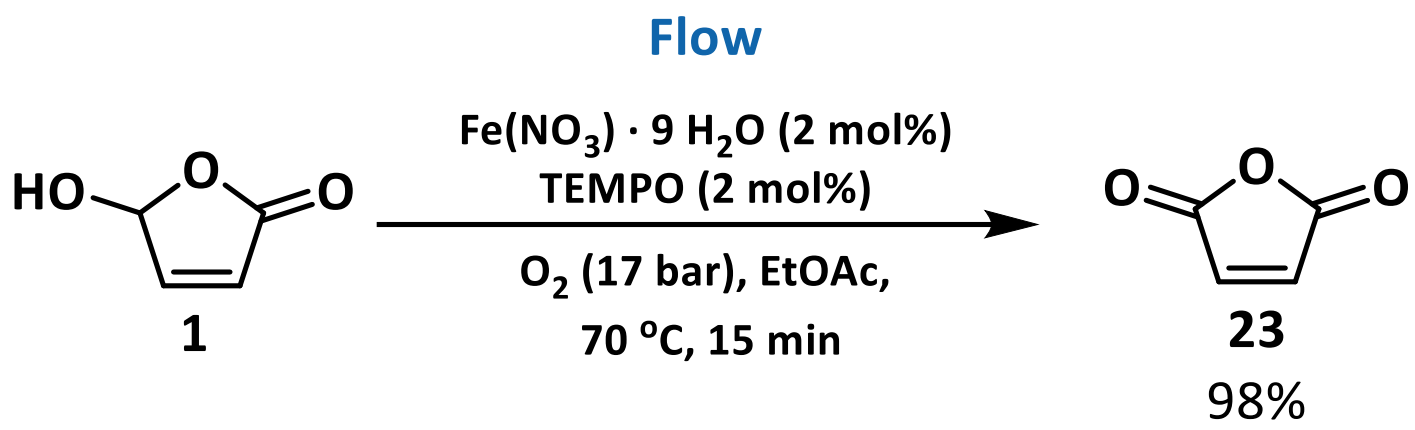


**Scheme 5:** Continuous flow production of MANh from HFO using oxygen.[4]

| | |
|---|---|
| M(HFO): | 100.07 g/mol |
| M(MAnh): | 98.1 g/mol |
| M($Fe(NO_3) \cdot 9\ H_2O$): | 403.99 g/mol |
| M(TEMPO): | 156.25 g/mol |

Continuous-flow production of maleic anhydride (MAnh) from HFO was reported with a 98% yield.[4] Accordingly, 1 mol of HFO produces 0.98 mol of MAnh, while the remaining 0.02 mol of HFO was considered an unproductive fraction and included as waste in the E-factor calculation. The process employed TEMPO (2 mol%) and $Fe(NO_3)_3 \cdot 9H_2O$ as catalysts. As no recovery or recycling of either catalyst was reported, both were considered waste and included in the E-factor calculation. $O_2$ was supplied at a pressure of 17 bar; however, the amount of $O_2$ used could not be quantified from the reported information. Therefore, $O_2$ consumption was not included in the E-factor calculation.

**Table 5:** Mass of products and waste generated in the oxidation of HFO to MA.[4]

| Products | |
|---|---|
| 0.98 mol MA | 96.14 g |
| **Waste** | |
| 0.02 mol HFO | 2 g |
| 0.02 mol TEMPO | 3.13 g |
| 0.02 mol $Fe(NO_3) \cdot 9\ H_2O$ | 8.08 g |

$$E\ Factor = \frac{mass\ waste}{mass\ products} = \frac{2 + 3.13 + 8.08}{96.14} = 0.14$$

## 7. Gamma butyrolactone (GBL)

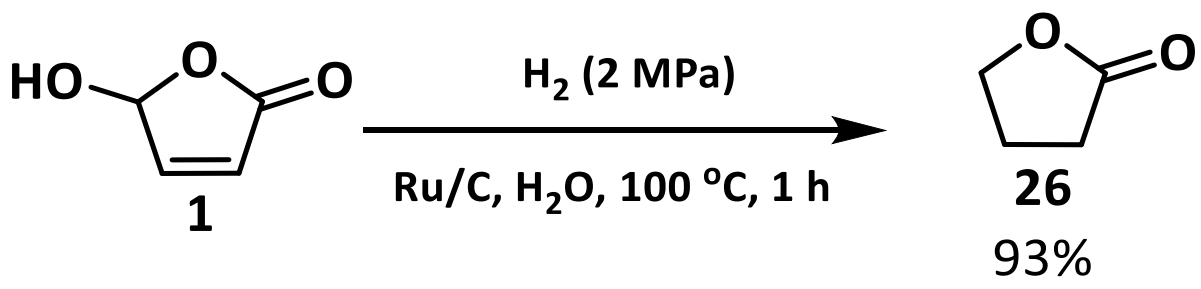

Scheme 6: Hydrogenation of HFO over Ru/C towards GBL.[1]

M(HFO): 100.07 g/mol

MW(GBL): 86.09 g/mol

Hydrogenation of 1 mmol of HFO was reported to afford γ-butyrolactone (GBL) in 93% yield, corresponding to 0.93 mmol of GBL.[1] The remaining 7% of HFO that did not form GBL was considered an unproductive fraction and included as waste in the E-factor calculation. The heterogeneous Ru/C catalyst was assumed to be recoverable and reusable over multiple reaction cycles and was therefore not considered waste in the E-factor calculation.

$H_2$ was supplied at a pressure of 2 MPa; however, the amount of $H_2$ used could not be quantified from the reported information. Therefore, $H_2$ consumption was not included in the E-factor calculation.

**Table 6:** Mass of products and waste generated in the hydrogenation of HFO over Ru/C to GBL.[1]

| Products | |
|---|---|
| 0.93 mmol GBL | 0.08 g |
| **Waste** | |
| 0.07 mmol HFO | 0.007 g |

$$E\ Factor = \frac{mass\ waste}{mass\ products} = \frac{0.007}{0.08} = 0.09$$

## 8. THF

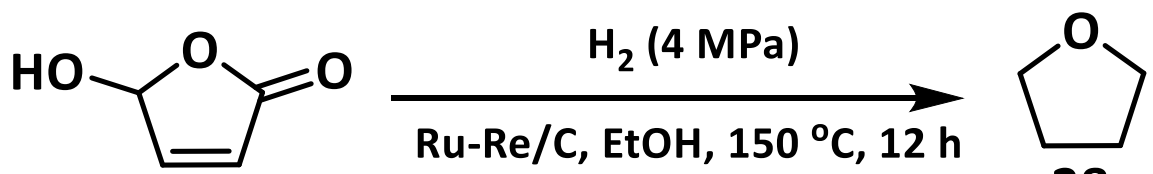

1 28 64%

**Scheme 7:** Hydrogenation of HFO over Ru-Re/C towards THF.[1]

M(HFO): 100.07 g/mol

M(THF): 72.11 g/mol

Hydrogenation of 1 mmol of HFO was reported to afford THF in 64% yield, corresponding to 0.64 mmol of THF.[1] The remaining 36% of HFO that did not form THF was considered an unproductive fraction and included as waste in the E-factor calculation. The heterogeneous Ru-Re/C catalyst was assumed to be recoverable and reusable over multiple reaction cycles and was therefore not considered waste in the E-factor calculation.

$H_2$ was supplied at a pressure of 4 MPa; however, the amount of $H_2$ used could not be quantified from the reported information. Therefore, $H_2$ consumption was not included in the E-factor calculation.

**Table 7:** Mass of products and waste generated in the hydrogenation of HFO over Ru-Re/C to THF.[1]

| **Products** | |
|---|---|
| 0.64 mmol THF | 0.046 g |
| **Waste** | |
| 0.36 mmol HFO | 0.036 g |

$$E\ Factor = \frac{mass\ waste}{mass\ products} = \frac{0.036}{0.046} = 0.78$$

## 9. 1,4-butanediol (BDO)

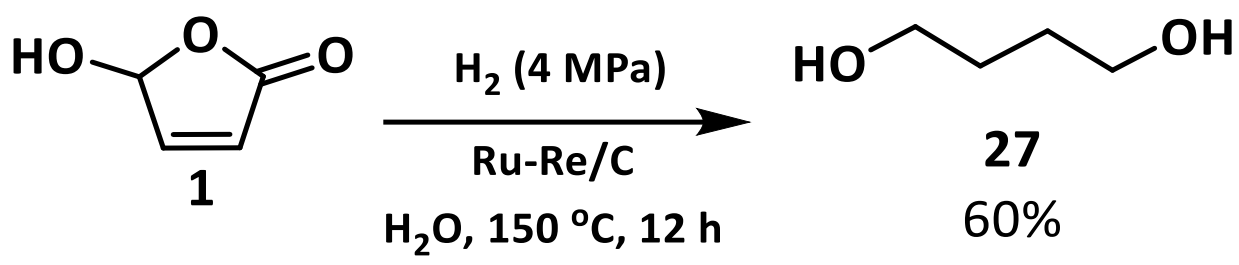


**Scheme 8:** Hydrogenation of HFO over Ru-Re/C towards BDO.[1]

M(HFO): 100.07 g/mol

MW(BDO): 90.12 g/mol

Hydrogenation of 1 mmol of HFO was reported to afford 1,4-butanediol (BDO) in 60% yield, corresponding to 0.6 mmol of BDO.[1] The remaining 40% of HFO that did not form BDO was considered an unproductive fraction and included as waste in the E-factor calculation. The heterogeneous Ru-Re/C catalyst was assumed to be recoverable and reusable over multiple reaction cycles and was therefore not considered waste in the E-factor calculation.

$H_2$ was supplied at a pressure of 4 MPa; however, the amount of $H_2$ used could not be quantified from the reported information. Therefore, $H_2$ consumption was not included in the E-factor calculation.

**Table 8:** Mass of products and waste generated in the hydrogenation of HFO over Ru-Re/C to BDO.[1]

| **Products** | |
|---|---|
| 0.6 mmol BDO | 0.054 g |
| **Waste** | |
| 0.4 mmol HFO | 0.040 g |

$$E\ Factor = \frac{mass\ waste}{mass\ products} = \frac{0.040}{0.054} = 0.74$$

## 10. γ-aminobutyric acid (GABA)

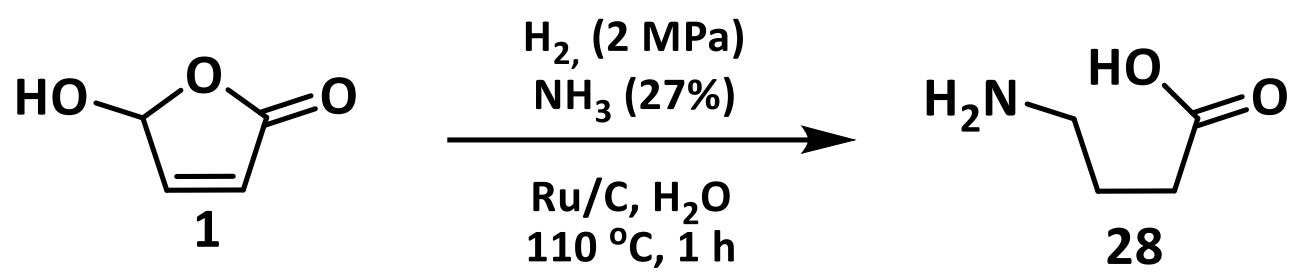


71%

**Scheme 9:** Reductive aminolysis of HFO over Ru/C towards GABA.[1]

| | |
|---|---|
| M(HFO): | 100.07 g/mol |
| M(GABA): | 103.12 g/mol |
| M($NH_3$): | 17.03 g/mol |

7.5 mL 27 % $NH_3$ gives 107 mmol $NH_3$

Reductive aminolysis of 5.56 mmol of HFO was reported to afford γ-aminobutyric acid (GABA) in 71% yield, corresponding to 3.95 mmol of GABA.[1] The remaining 29% of HFO that did not form GABA was considered an unproductive fraction and included as waste in the E-factor calculation. An excess of 107 mmol of $NH_3$ was supplied to the reaction. Assuming a stoichiometric requirement of 1 mmol of $NH_3$ per mmol of HFO for conversion to GABA, 3.95 mmol of $NH_3$ was considered consumed, while the remaining 103.5 mmol was included as waste in the E-factor calculation. The heterogeneous Ru/C catalyst was assumed to be recoverable and reusable over multiple reaction cycles and was therefore not considered waste in the E-factor calculation.

$H_2$ was supplied at a pressure of 2 MPa; however, the amount of $H_2$ used could not be quantified from the reported information. Therefore, $H_2$ consumption was not included in the E-factor calculation.

**Table 9:** Mass of products and waste generated in the reductive aminolysis of HFO over Ru/C to GABA.[1]

| | |
|---|---|
| **Products** | |
| 3.95 mmol GABA | 0.407 g |
| **Waste** | |
| 1.61 mmol HFO | 0.161 g |
| 103.05 mmol $NH_3$ | 1.755 g |

$$E\ Factor = \frac{mass\ waste}{mass\ products} = \frac{0.161 + 1.755}{0.407} = 4.7$$

## 11. 2-pyrrolidone

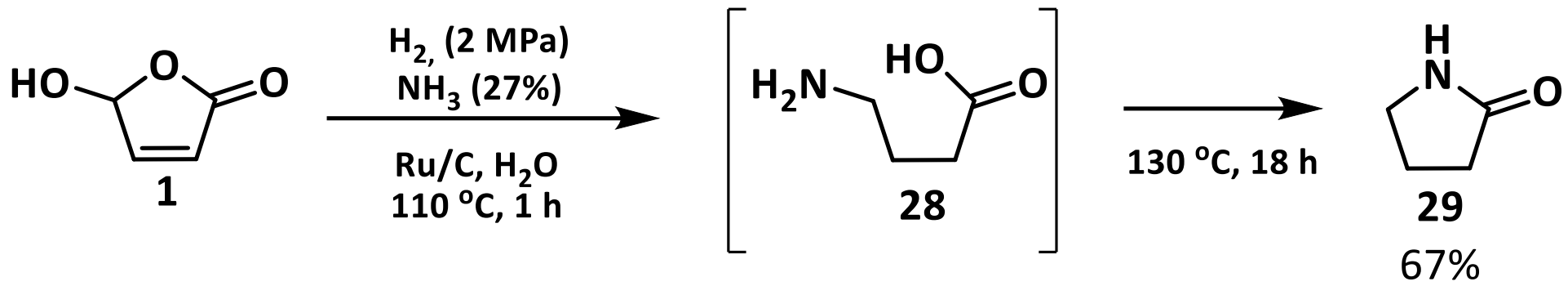

**Scheme 10:** Reductive aminolysis of HFO over Ru/C towards 2-pyrrolidone.[1]

M(HFO): 100.07 g/mol

M(2-pyrrolidone): 85.11 g/mol

M($NH_3$): 17.03 g/mol

7.5 mL 27 % $NH_3$ gives 107 mmol $NH_3$

Reductive aminolysis of 5.56 mmol of HFO was reported to afford 2-pyrrolidone in 67% yield, corresponding to 3.73 mmol of 2-pyrrolidone.[1] The remaining 33% of HFO that did not form 2-pyrrolidone was considered an unproductive fraction and included as waste in the E-factor calculation. An excess of 107 mmol of $NH_3$ was supplied to the reaction. Assuming a stoichiometric requirement of 1 mmol of $NH_3$ per mmol of HFO for conversion to 2-pyrrolidone, 3.73 mmol of $NH_3$ was considered consumed, while the remaining 103.27 mmol was included as waste in the E-factor calculation. The heterogeneous Ru/C catalyst was assumed to be recoverable and reusable over multiple reaction cycles and was therefore not considered waste in the E-factor calculation.

$H_2$ was supplied at a pressure of 2 MPa; however, the amount of $H_2$ used could not be quantified from the reported information. Therefore, $H_2$ consumption was not included in the E-factor calculation.

**Table 10:** Mass of products and waste generated in the reductive aminolysis of HFO over Ru/C to 2-pyrrolidone.[1]

| | |
|---|---|
| **Products** | |
| 3.73 mmol 2-pyrrolidone | 0.317 g |
| **Waste** | |
| 1.83 mmol HFO | 0.183 g |
| 103.27 mmol $NH_3$ | 1.759 g |

$$E\ Factor = \frac{mass\ waste}{mass\ products} = \frac{0.183 + 1.759}{0.317} = 6.13$$

## 12. Succinic acid (SA)

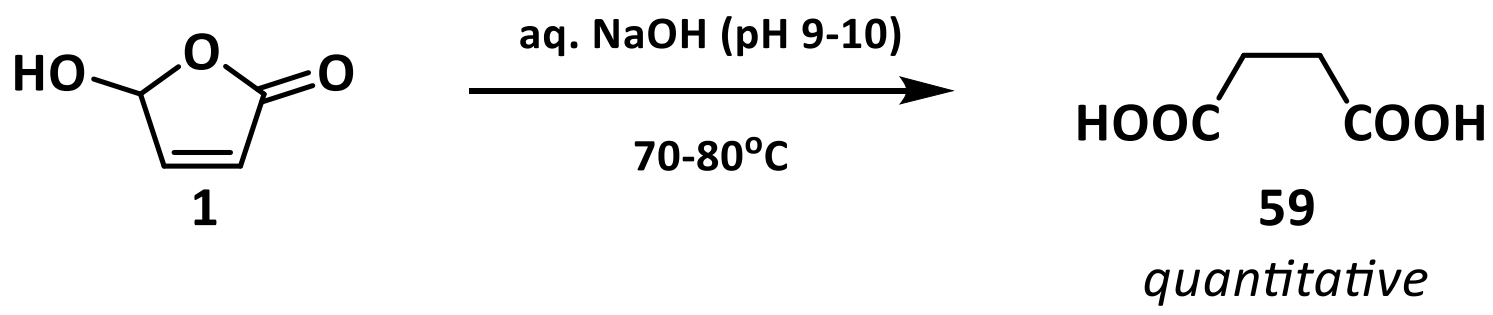

**Scheme 11:** Tautomeric transformation of HFO to SA in NaOH (pH 10) solution.[5]

M(HFO): 100.07 g/mol

M(SA): 85.11 g/mol

M(NaOH): 17.03 g/mol

8.6 mL NaOH (pH 10) gives 0.035 mg NaOH.

Tautomeric transformation of 1 mmol HFO to succinic acid (SA) was reported with complete conversion in NaOH solution.[5] The 8.6 mL NaOH solution (pH 10) contained 0.035 mg of NaOH, which was considered the only waste stream in this process.

**Table 11:** Mass of products and waste generated in the tautomeric transformation of HFO to SA in NaOH (pH 10) solution.[5]

| Products | |
|---|---|
| 1 mmol HFO | 100 mg |
| **Waste** | |
| NaOH | 0.035 g |

$$E\ Factor = \frac{mass\ waste}{mass\ products} = \frac{0.035}{100} = 0.00035$$

## 13. References


1. Palai, Y. N., Fukuoka, A. & Shrotri, A. Unlocking the Potential of 5-Hydroxy-2(5H)-furanone as a Platform for Bio-Based Four Carbon Chemicals. *ACS Catal.* **14**, 2545–2551 (2024).
2. Zhu, W. *et al.* Mechanistic insights into biomass-based furfural electrooxidation to 5-hydroxy-2(5H)-furanone over mesoporous polymers. *Chemical Communications* **61**, 6835–6838 (2025).
3. Edwards, M. D. *et al.* Process Intensification of the Continuous Synthesis of Bio-Derived Monomers for Sustainable Coatings Using a Taylor Vortex Flow Reactor. *Org. Process Res. Dev.* **28**, 1917–1928 (2024).
4. Mortier, J., Stevens, C. V. & Heugebaert, T. S. A. Efficient continuous flow oxidation of furfural to maleic anhydride using O2 as a green oxidant. *Green Chemistry* **27**, 5063–5072 (2025).
5. Poskonin, V. V. & Badovskaya, L. A. Unusual conversion of 5-hydroxy-2(5H)-furanone in aqueous solution. *Chem. Heterocycl. Compd. (N. Y).* **39**, 594–597 (2003).
