# Supplementary material for: 5-Hydroxy-2(5H)-furanone (HFO) – a versatile and bio-based platform chemical
Source: Chem Soc Rev. 2026 Oct 26;55(22):11855–913. doi: 10.1039/d6cs00842a (PMC13704320; doi:10.1039/d6cs00842a)
Supplement: Supplementary file 2 [file CS-055-D6CS00842A-s001.pdf]

Supplementary Information (SI) for Chemical Society Reviews.
This journal is © The Royal Society of Chemistry

Supporting information

5-Hydroxy-2(5H)-furanone (HFO) – a versatile and bio-based platform chemical

Jelmer T. Meijer[a], Magdalena M. Kleybolte[a], Andries Jensma[a], Jeya P. Kaniraj[b], Thomas Freese[a,b], Ben L. Feringa*[a]*

Contents

1. Equations for E-factor and carbon yield 2
   1.1 E-factors 2
   1.2 Carbon yield 2
2. Heterogeneous catalytic oxidation 3
3. Electrochemical oxidation 4
4. Photochemical oxidation 5
5. Maleic acid (MA) 6
6. Maleic anhydride (MAnh) 7
7. Gamma butyrolactone (GBL) 8
8. THF 9
9. 1,4-butanediol (BDO) 10
10. γ-aminobutyric acid (GABA) 11
11. 2-pyrrolidone 12
12. Succinic acid (SA) 13
13. References 14

1. Equations for E-factor and carbon yield

1.1 E-factors

Calculations were performed based on the reported reaction yields. Solvents and water were not included in the E-factor calculations because no reliable solvent recycling data were available.

$$E\ Factor = \frac{mass\ waste}{mass\ products}$$

1.2 Carbon yield

The reaction has a theoretical maximum carbon yield of 80%, as HFO contains four carbon atoms, whereas furfural contains five.

$$Carbon\ yield\ (\%) = Molar\ yield\ of\ HFO(\%) * 0.8$$

2. Heterogeneous catalytic oxidation

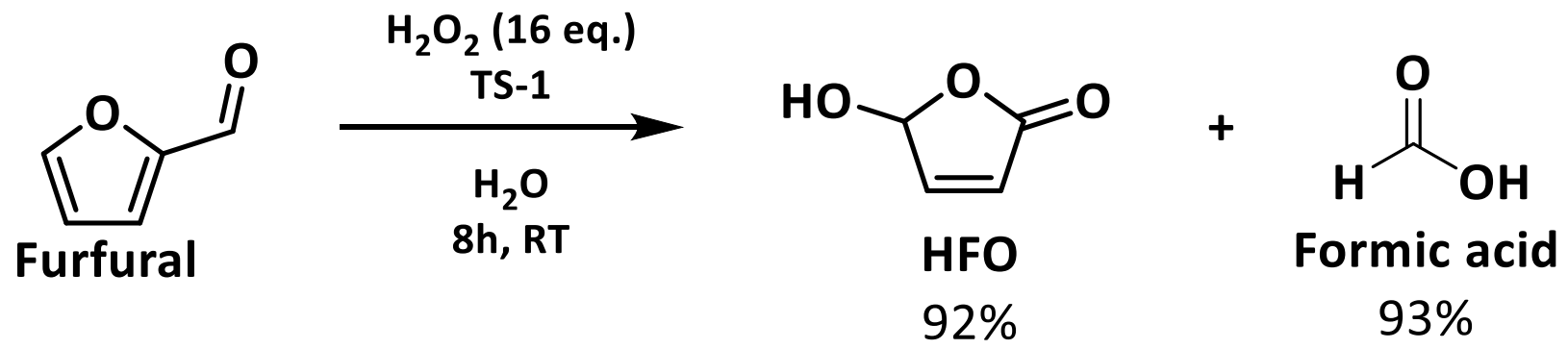

Scheme 1: Heterogeneous catalytic oxidation of furfural over TS-1 catalyst, using H_2O_2 as oxidant.[1]

M(HFO):	100.07 g/mol
M(Formic acid):	46.03 g/mol
M(H_2O_2):	34.014 g/mol
M(MA):	116.07 g/mol
M(Furfural):	96.08 g/mol

Heterogeneous catalytic oxidation of 1 mmol of furfural was reported over TS-1, using H_2O_2 as oxidant.[1] Complete conversion of furfural was achieved, affording HFO in 92% yield and maleic acid (MA) in 5% yield, while formic acid (93%) was formed as a by-product. A total of 16 mmol of H_2O_2 was added to the reaction, of which 3.1 mmol was consumed. For the E-factor calculation, only HFO was considered the desired product, while formic acid was regarded as a value-added by-product. MA was treated as a non-product stream resulting from the undesired overoxidation of HFO. In addition, the 8% unreacted furfural was regarded as waste in the E-factor calculation.

Table 1: Mass of products and waste generated in the heterogeneous catalytic oxidation of furfural to HFO.[1]

Products	
0.92 mmol HFO	0.092 g
0.93 mmol formic acid	0.043 g
**Waste**	
12.9 mmol H_2O_2	0.449 g
0.05 mmol MA	0.006 g
0.08 mmol furfural	0.008 g

$$E\ Factor = \frac{mass\ waste}{mass\ products} = \frac{0.449 + 0.006 + 0.008}{0.092 + 0.043} = 3.4$$

$$Carbon\ yield\ (\%) = 92\% * 0.8 = 73.6\%$$

3. Electrochemical oxidation

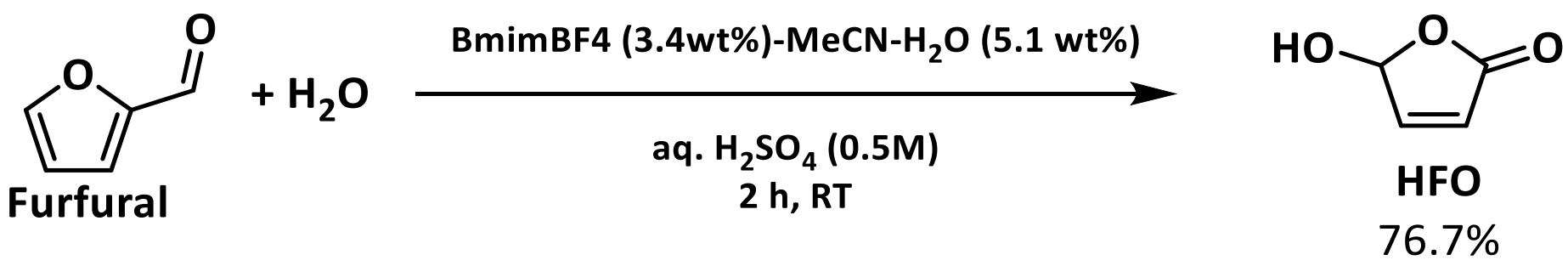

Scheme 2: Electrochemical oxidation of furfural over Meso-PDA.[2]

M(HFO): 100.07 g/mol

M(Furfural): 96.08 g/mol

3.4 wt% $BmimBF_4$ in MeCN gives 0.188 g $BmimBF_4$

Electrochemical oxidation of 0.5 mmol of furfural over spherical mesoporous polymer Meso-PDA afforded HFO at 76.7% conversion, corresponding to 0.384 mmol of HFO.[2] The anolyte consisted of $BmimBF_4$ (3.4 wt%), MeCN, and H_2O (5.1 wt%). As the recovery and recycling of the $BmimBF_4$ electrolyte were not demonstrated, it was considered waste and was therefore included in the E-factor calculation. Only HFO was considered the desired product.

Table 2: Mass of products and waste generated in the electrochemical oxidation of furfural to HFO.[2]

**Products**	
0.384 mmol HFO	0.038 g
**Waste**	
BMIMBF4	0.188 g
0.116 mmol Furfural	0.011 g

$$E\ Factor = \frac{mass\ waste}{mass\ products} = \frac{0.188 + 0.011}{0.038} = 5.2$$

$$Carbon\ yield\ (\%) = 76.7 * 0.8 = 61.4$$

4. Photochemical oxidation

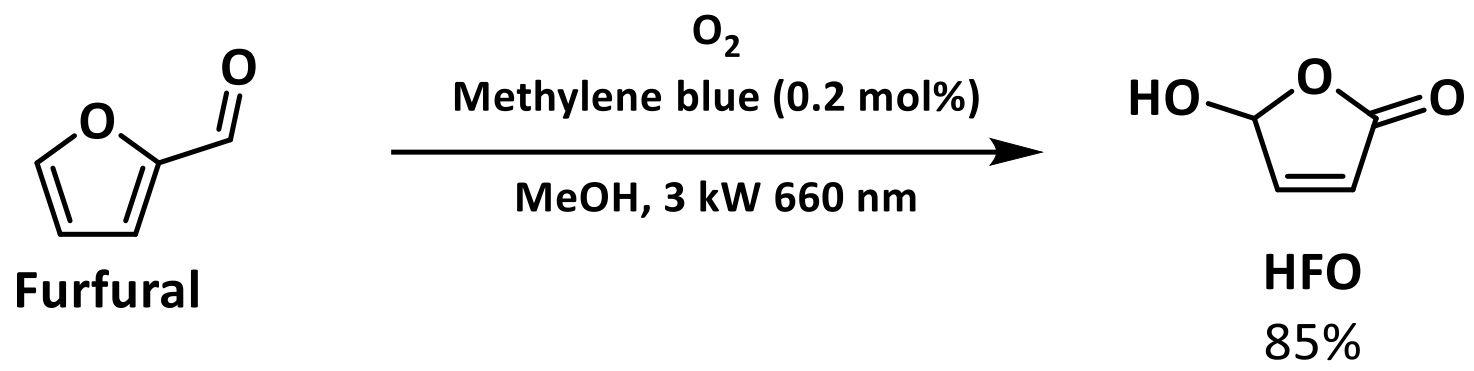

Scheme 3: Photochemical oxidation of furfural in a continuous photo-flow reactor.[3]

M(HFO): 100.07 g/mol

M(methyl formate): 60.052 g/mol

M(Furfural): 96.08 g/mol

M(methylene blue): 319.85 g/mol

M(O_2): 32.00 g/mol

Continuous-flow photooxidation of furfural was reported to achieve complete furfural conversion, affording HFO in 85% yield.[3] Accordingly, 1 mol of furfural produces 0.85 mol of HFO, while methyl formate is formed stoichiometrically alongside HFO as a value-added product. Methylene blue was employed as a photosensitizer at a loading of 0.2 mol% and was not reported to be recovered or recycled; it was therefore considered waste in the E-factor calculation. The remaining 15% of converted furfural that did not contribute to HFO formation was likewise considered waste. Accordingly, both the non-recovered photosensitizer and the unproductive fraction of furfural were included as waste streams in the E-factor calculation. A total of 1.2 equivalents of O_2 was supplied to the reactor, of which 0.85 equivalents were consumed in the formation of HFO. Therefore, the remaining 0.35 mol of O_2 was included as waste in the E-factor calculation.

Table 3: Mass of products and waste generated in the photochemical oxidation of furfural to HFO.[3]

**Products**	
0.85 mol HFO	85.06 g
0.85 mol methyl formate	51.04 g
**Waste**	
0.15 mol furfural	14.41 g
0.002 mol methylene blue	0.64 g
0.35 mol O_2	11.2 g

$$E\ Factor = \frac{mass\ waste}{mass\ products} = \frac{14.41 + 0.64 + 11.2}{85.06 + 51.04} = 0.19$$

$$Carbon\ yield\ (\%) = 85\% * 0.8 = 68\%$$

5. Maleic acid (MA)

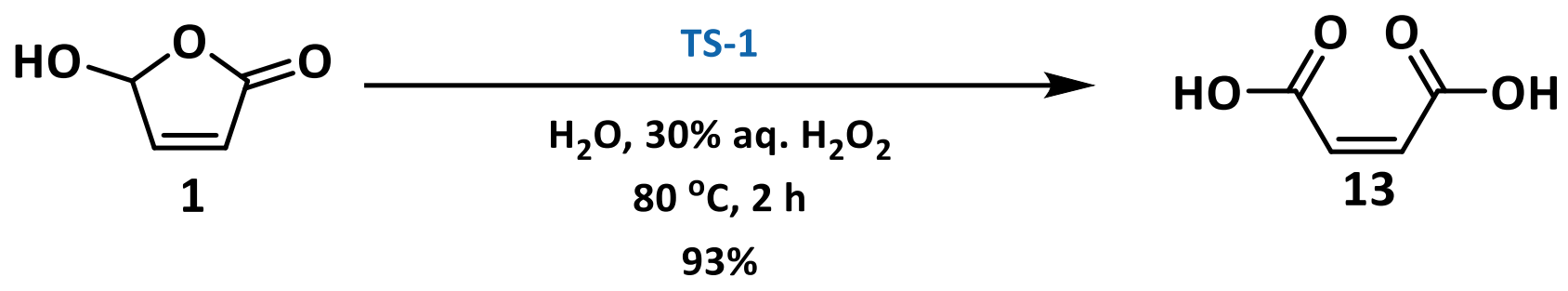

Scheme 4: Oxidation of HFO with TS-1 catalyst, using H_2O_2 as oxidant.[1]

M(HFO): 100.07 g/mol

M(H_2O_2): 34.014 g/mol

M(MA): 116.07 g/mol

2 mL 30 % H_2O_2 gives 19.6 mmol H_2O_2.

Oxidation of 1 mmol of HFO was reported to afford maleic acid (MA) in 93% yield.[1] The remaining 7% of HFO was reported to form unidentified products and was therefore considered an unproductive fraction and included as waste in the E-factor calculation. An excess of 19.6 mmol of H_2O_2 was used in the reaction. Assuming a stoichiometric requirement of 1 mmol of H_2O_2 per mmol of HFO for conversion to MA, 1.0 mmol of H_2O_2 was considered consumed, while the remaining 18.6 mmol was included as waste in the E-factor calculation.

Table 4: Mass of products and waste generated in the oxidation of HFO to MA.[1]

Products	
0.93 mmol MA	0.108 g
**Waste**	
0.07 mmol HFO	0.007 g
18.6 mmol H_2O_2	0.633 g

$$E\ Factor = \frac{mass\ waste}{mass\ products} = \frac{0.633 + 0.007}{0.108} = 5.9$$

6. Maleic anhydride (MAnh)

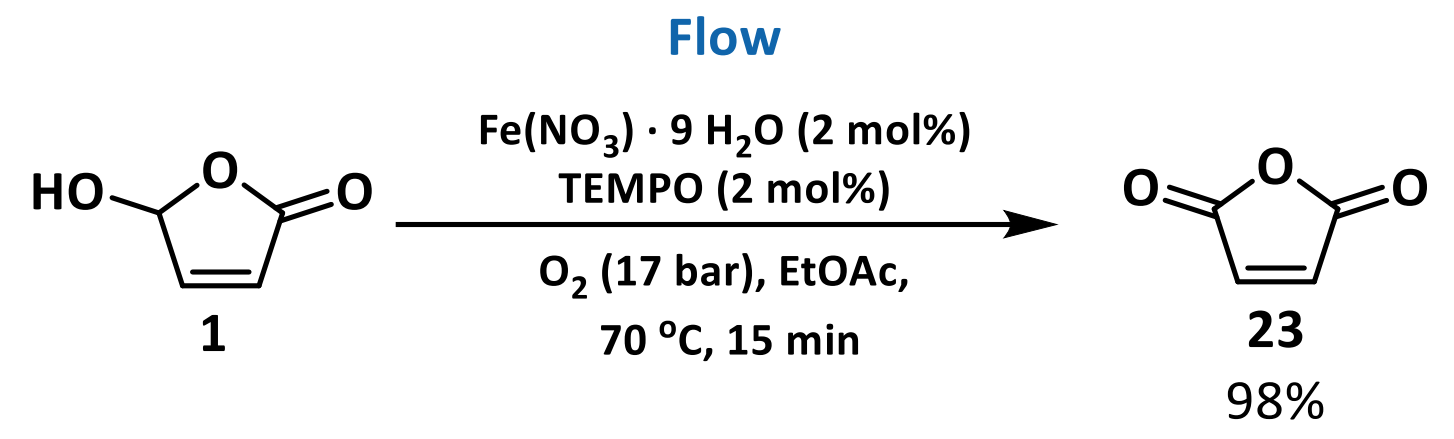

Scheme 5: Continuous flow production of MANh from HFO using oxygen.[4]

M(HFO):	100.07 g/mol
M(MAnh):	98.1 g/mol
M(Fe(NO$_3$) · 9 H$_2$O):	403.99 g/mol
M(TEMPO):	156.25 g/mol

Continuous-flow production of maleic anhydride (MAnh) from HFO was reported with a 98% yield.[4] Accordingly, 1 mol of HFO produces 0.98 mol of MAnh, while the remaining 0.02 mol of HFO was considered an unproductive fraction and included as waste in the E-factor calculation. The process employed TEMPO (2 mol%) and $Fe(NO_3)_3 \cdot 9H_2O$ as catalysts. As no recovery or recycling of either catalyst was reported, both were considered waste and included in the E-factor calculation. O_2 was supplied at a pressure of 17 bar; however, the amount of O_2 used could not be quantified from the reported information. Therefore, O_2 consumption was not included in the E-factor calculation.

Table 5: Mass of products and waste generated in the oxidation of HFO to MA.[4]

**Products**	
0.98 mol MA	96.14 g
**Waste**	
0.02 mol HFO	2 g
0.02 mol TEMPO	3.13 g
0.02 mol Fe(NO$_3$) · 9 H$_2$O	8.08 g

$$E\ Factor = \frac{mass\ waste}{mass\ products} = \frac{2 + 3.13 + 8.08}{96.14} = 0.14$$

7. Gamma butyrolactone (GBL)

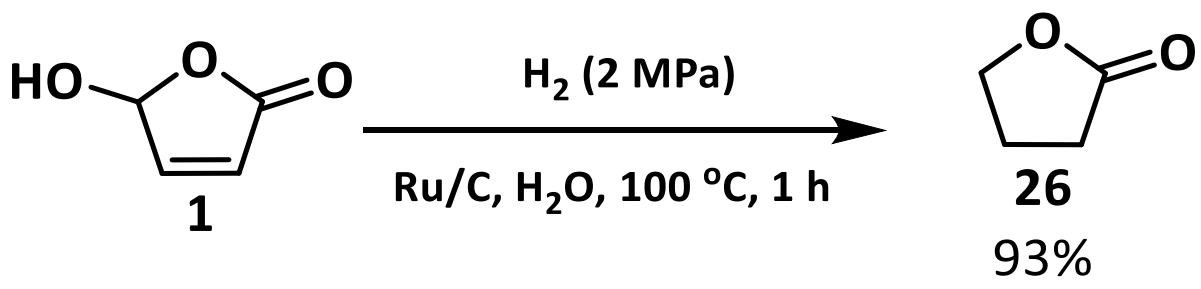

Scheme 6: Hydrogenation of HFO over Ru/C towards GBL.[1]

M(HFO): 100.07 g/mol

MW(GBL): 86.09 g/mol

Hydrogenation of 1 mmol of HFO was reported to afford γ-butyrolactone (GBL) in 93% yield, corresponding to 0.93 mmol of GBL.[1] The remaining 7% of HFO that did not form GBL was considered an unproductive fraction and included as waste in the E-factor calculation. The heterogeneous Ru/C catalyst was assumed to be recoverable and reusable over multiple reaction cycles and was therefore not considered waste in the E-factor calculation.

H_2 was supplied at a pressure of 2 MPa; however, the amount of H_2 used could not be quantified from the reported information. Therefore, H_2 consumption was not included in the E-factor calculation.

Table 6: Mass of products and waste generated in the hydrogenation of HFO over Ru/C to GBL.[1]

**Products**	
0.93 mmol GBL	0.08 g
**Waste**	
0.07 mmol HFO	0.007 g

$$E\ Factor = \frac{mass\ waste}{mass\ products} = \frac{0.007}{0.08} = 0.09$$

8. THF

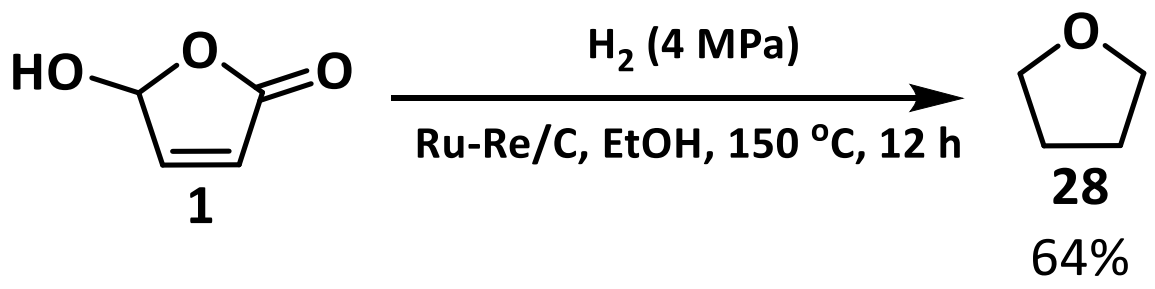

Scheme 7: Hydrogenation of HFO over Ru-Re/C towards THF.[1]

M(HFO): 100.07 g/mol

M(THF): 72.11 g/mol

Hydrogenation of 1 mmol of HFO was reported to afford THF in 64% yield, corresponding to 0.64 mmol of THF.[1] The remaining 36% of HFO that did not form THF was considered an unproductive fraction and included as waste in the E-factor calculation. The heterogeneous Ru-Re/C catalyst was assumed to be recoverable and reusable over multiple reaction cycles and was therefore not considered waste in the E-factor calculation.

H_2 was supplied at a pressure of 4 MPa; however, the amount of H_2 used could not be quantified from the reported information. Therefore, H_2 consumption was not included in the E-factor calculation.

Table 7: Mass of products and waste generated in the hydrogenation of HFO over Ru-Re/C to THF.[1]

**Products**	
0.64 mmol THF	0.046 g
**Waste**	
0.36 mmol HFO	0.036 g

$$E\ Factor = \frac{mass\ waste}{mass\ products} = \frac{0.036}{0.046} = 0.78$$

9. 1,4-butanediol (BDO)

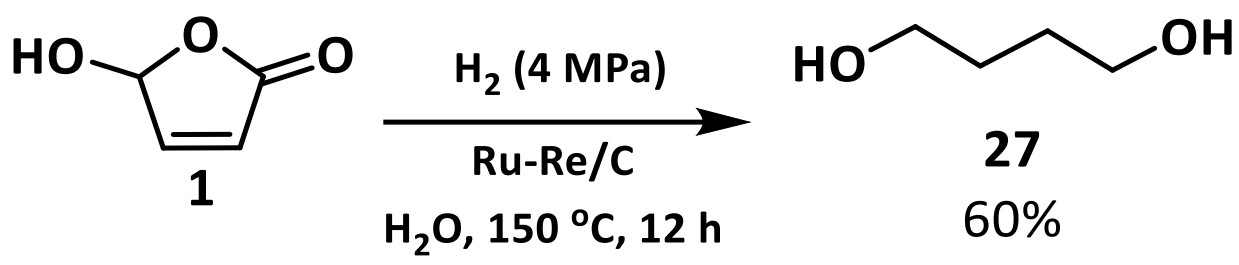

Scheme 8: Hydrogenation of HFO over Ru-Re/C towards BDO.[1]

M(HFO):	100.07 g/mol
MW(BDO):	90.12 g/mol

Hydrogenation of 1 mmol of HFO was reported to afford 1,4-butanediol (BDO) in 60% yield, corresponding to 0.6 mmol of BDO.[1] The remaining 40% of HFO that did not form BDO was considered an unproductive fraction and included as waste in the E-factor calculation. The heterogeneous Ru-Re/C catalyst was assumed to be recoverable and reusable over multiple reaction cycles and was therefore not considered waste in the E-factor calculation.

H_2 was supplied at a pressure of 4 MPa; however, the amount of H_2 used could not be quantified from the reported information. Therefore, H_2 consumption was not included in the E-factor calculation.

Table 8: Mass of products and waste generated in the hydrogenation of HFO over Ru-Re/C to BDO.[1]

**Products**	
0.6 mmol BDO	0.054 g
**Waste**	
0.4 mmol HFO	0.040 g

$$E\ Factor = \frac{mass\ waste}{mass\ products} = \frac{0.040}{0.054} = 0.74$$

10. γ-aminobutyric acid (GABA)

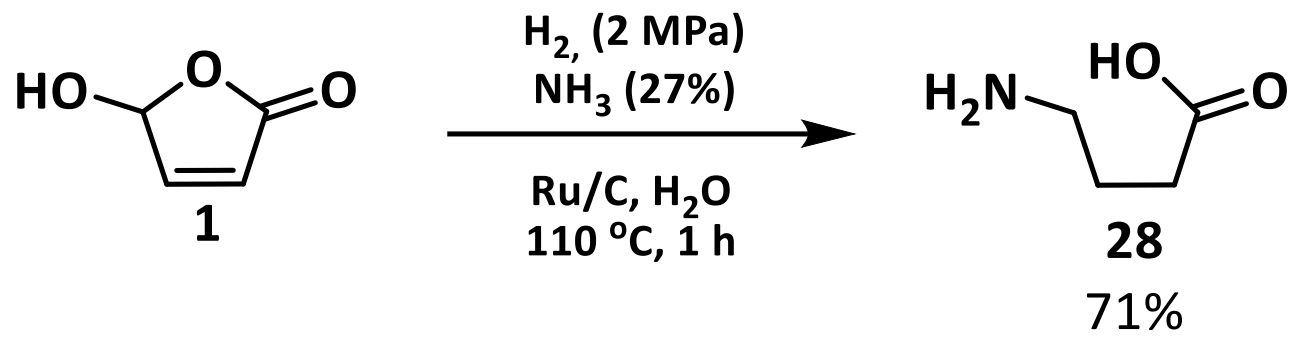

Scheme 9: Reductive aminolysis of HFO over Ru/C towards GABA.[1]

M(HFO): 100.07 g/mol

M(GABA): 103.12 g/mol

M(NH_3): 17.03 g/mol

7.5 mL 27 % NH_3 gives 107 mmol NH_3

Reductive aminolysis of 5.56 mmol of HFO was reported to afford γ-aminobutyric acid (GABA) in 71% yield, corresponding to 3.95 mmol of GABA.[1] The remaining 29% of HFO that did not form GABA was considered an unproductive fraction and included as waste in the E-factor calculation. An excess of 107 mmol of NH_3 was supplied to the reaction. Assuming a stoichiometric requirement of 1 mmol of NH_3 per mmol of HFO for conversion to GABA, 3.95 mmol of NH_3 was considered consumed, while the remaining 103.5 mmol was included as waste in the E-factor calculation. The heterogeneous Ru/C catalyst was assumed to be recoverable and reusable over multiple reaction cycles and was therefore not considered waste in the E-factor calculation.

H_2 was supplied at a pressure of 2 MPa; however, the amount of H_2 used could not be quantified from the reported information. Therefore, H_2 consumption was not included in the E-factor calculation.

Table 9: Mass of products and waste generated in the reductive aminolysis of HFO over Ru/C to GABA.[1]

Products	
3.95 mmol GABA	0.407 g
**Waste**	
1.61 mmol HFO	0.161 g
103.05 mmol NH_3	1.755 g

$$E\ Factor = \frac{mass\ waste}{mass\ products} = \frac{0.161 + 1.755}{0.407} = 4.7$$

11. 2-pyrrolidone

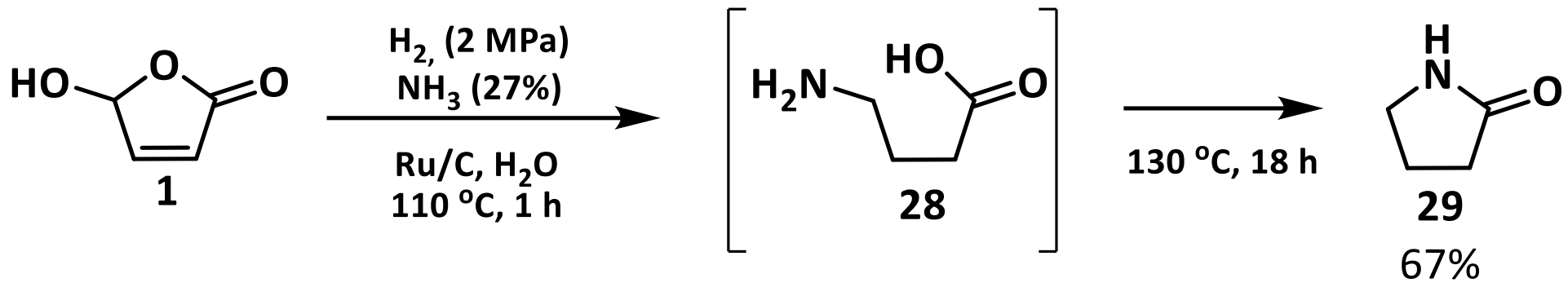

Scheme 10: Reductive aminolysis of HFO over Ru/C towards 2-pyrrolidone.[1]

M(HFO): 100.07 g/mol

M(2-pyrrolidone): 85.11 g/mol

M(NH_3): 17.03 g/mol

7.5 mL 27 % NH_3 gives 107 mmol NH_3

Reductive aminolysis of 5.56 mmol of HFO was reported to afford 2-pyrrolidone in 67% yield, corresponding to 3.73 mmol of 2-pyrrolidone.[1] The remaining 33% of HFO that did not form 2-pyrrolidone was considered an unproductive fraction and included as waste in the E-factor calculation. An excess of 107 mmol of NH_3 was supplied to the reaction. Assuming a stoichiometric requirement of 1 mmol of NH_3 per mmol of HFO for conversion to 2-pyrrolidone, 3.73 mmol of NH_3 was considered consumed, while the remaining 103.27 mmol was included as waste in the E-factor calculation. The heterogeneous Ru/C catalyst was assumed to be recoverable and reusable over multiple reaction cycles and was therefore not considered waste in the E-factor calculation.

H_2 was supplied at a pressure of 2 MPa; however, the amount of H_2 used could not be quantified from the reported information. Therefore, H_2 consumption was not included in the E-factor calculation.

Table 10: Mass of products and waste generated in the reductive aminolysis of HFO over Ru/C to 2-pyrrolidone.[1]

Products	
3.73 mmol 2-pyrrolidone	0.317 g
**Waste**	
1.83 mmol HFO	0.183 g
103.27 mmol NH_3	1.759 g

$$E\ Factor = \frac{mass\ waste}{mass\ products} = \frac{0.183 + 1.759}{0.317} = 6.13$$

12. Succinic acid (SA)

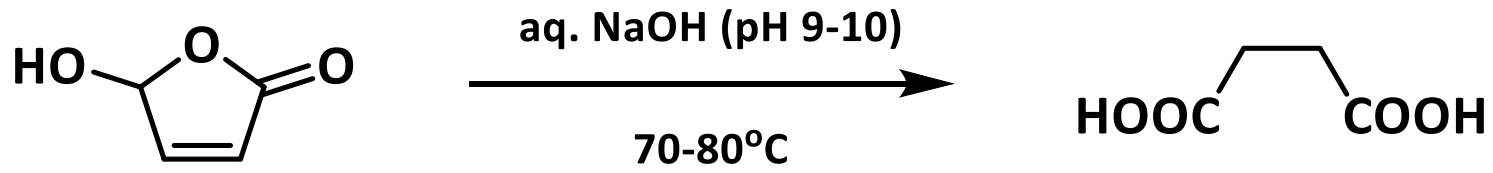

1 59

quantitative

Scheme 11: Tautomeric transformation of HFO to SA in NaOH (pH 10) solution.[5]

M(HFO): 100.07 g/mol

M(SA): 85.11 g/mol

M(NaOH): 17.03 g/mol

8.6 mL NaOH (pH 10) gives 0.035 mg NaOH.

Tautomeric transformation of 1 mmol HFO to succinic acid (SA) was reported with complete conversion in NaOH solution.[5] The 8.6 mL NaOH solution (pH 10) contained 0.035 mg of NaOH, which was considered the only waste stream in this process.

Table 11: Mass of products and waste generated in the tautomeric transformation of HFO to SA in NaOH (pH 10) solution.[5]

**Products**	
1 mmol HFO	100 mg
**Waste**	
NaOH	0.035 g

$$E\ Factor = \frac{mass\ waste}{mass\ products} = \frac{0.035}{100} = 0.00035$$

13. References

1. Palai, Y. N., Fukuoka, A. & Shrotri, A. Unlocking the Potential of 5-Hydroxy-2(5H)-furanone as a Platform for Bio-Based Four Carbon Chemicals. *ACS Catal.* **14**, 2545–2551 (2024).
2. Zhu, W. *et al.* Mechanistic insights into biomass-based furfural electrooxidation to 5-hydroxy-2(5H)-furanone over mesoporous polymers. *Chemical Communications* **61**, 6835–6838 (2025).
3. Edwards, M. D. *et al.* Process Intensification of the Continuous Synthesis of Bio-Derived Monomers for Sustainable Coatings Using a Taylor Vortex Flow Reactor. *Org. Process Res. Dev.* **28**, 1917–1928 (2024).
4. Mortier, J., Stevens, C. V. & Heugebaert, T. S. A. Efficient continuous flow oxidation of furfural to maleic anhydride using O2 as a green oxidant. *Green Chemistry* **27**, 5063–5072 (2025).
5. Poskonin, V. V. & Badovskaya, L. A. Unusual conversion of 5-hydroxy-2(5H)-furanone in aqueous solution. *Chem. Heterocycl. Compd. (N. Y).* **39**, 594–597 (2003).
